# Supplementary material for: Actin-Associated Proteins and Small Molecules Targeting the Actin Cytoskeleton
Source: Int J Mol Sci. 2022 Feb 14;23(4):2118. doi: 10.3390/ijms23042118 (PMC8880164; doi:10.3390/ijms23042118)
Supplement: Supplementary file 1 [file ijms-23-02118-s001.zip › Table S1 A list of actin-associated proteins.pdf]

Table S1. A list of actin-associated proteins.

Protein name	Gene name	Chromosome	Size, aa	Binding mode(s)*	Function	Disease	Actin Binding domain	UniProt	Human Protein Atlas	Year found**	Reference
Unconventional myosin-Ia, Brush border myosin I (BBM1), Myosin I heavy chain (MIHC)	MYO1A, MYHL	12	1043	Motor	Regulate movement of organelles along actin filaments.	Deafness.	ABD (571-593) within motor domain	https://www.uniprot.org/uniprot/Q9UBC5	https://www.proteinatlas.org/ENSG00000166866-MYO1A	1985	[1-5]
Unconventional myosin-Ib, MYH-1c, Myosin I alpha (MMIa)	MYO1B	2	1136	Motor	Regulate cell migration, neurite outgrowth and vesicular transport.	Expression is increased in cancers.	ABD (578-600) within motor domain	https://www.uniprot.org/uniprot/O43795	https://www.proteinatlas.org/ENSG00000128641-MYO1B	1994	[4, 6]
Unconventional myosin-Ic, Myosin I beta (MMIb)	MYO1C	17	1063	Motor	Regulate transforming growth factor-β-signaling and fibrosis in podocytes.	Impaired visual function.	ABD (608-630) within motor domain	https://www.uniprot.org/uniprot/O00159	https://www.proteinatlas.org/ENSG00000197879-MYO1C	1994	[7-9]
Unconventional myosin-Id	MYO1D, KIAA0727	17	1006	Motor	Regulate endosomal protein trafficking.	Regulate regeneration process after demyelination. Laterality defects.	ABD (572-594) within motor domain	https://www.uniprot.org/uniprot/O94832	https://www.proteinatlas.org/ENSG00000176658-MYO1D	1994	[10-12]
Unconventional myosin-Ie, Myosin-Ic	MYO1E, MYO1C	15	1108	Motor	Required for normal kidney function.	Focal segmental glomerulosclerosis.	ABD (581-591) within motor domain	https://www.uniprot.org/uniprot/Q12965	https://www.proteinatlas.org/ENSG00000157483-MYO1E	1994	[13-15]
Unconventional myosin-If, Myosin -Ie	MYO1F	19	1098	Motor	Regulate neutrophil migration and immune signaling.	Thyroid cancer.	ABD (579-589) within motor domain	https://www.uniprot.org/uniprot/O00160	https://www.proteinatlas.org/ENSG00000142347-MYO1F	1997	[15, 16]
Unconventional myosin-Ig, Minor histocompatibility antigen HA-2 (mHag-HA-2)	MYO1G, HA2	7	1018	Motor	Regulate T-cell migration.	Acute Lymphoblastic Leukemia.	ABD (584-606) within motor domain	https://www.uniprot.org/uniprot/B0I1T2	https://www.proteinatlas.org/ENSG00000136286-MYO1G	2001	[5, 17]
Unconventional myosin-Ih, Myosin-1H	MYO1H	12	1032	Motor	Regulate CO2 sensitivity and respiratory control.	Congenital central hypoventilation.	ABD (578-600) within motor domain	https://www.uniprot.org/uniprot/Q8N1T3	https://www.proteinatlas.org/ENSG00000174527-MYO1H	2001	[5, 18]
Myosin-1, Myosin heavy chain 1, Myosin heavy chain 2x (MyHC-2x), Myosin heavy chain IIx/d (MyHC-IIx/d), Myosin heavy chain, skeletal muscle, adult 1	MYH1	17	1939	Motor	Muscle contraction.	Myopathies.	ABD (659-681, 761-775) within motor domain	https://www.uniprot.org/uniprot/P12882	https://www.proteinatlas.org/ENSG00000109061-MYH1	1977	[19]
Myosin-2, Myosin heavy chain 2, yosin heavy chain 2a (MyHC-2a, MyHC-IIa), Myosin heavy chain, skeletal muscle, adult 2	MYH2, MYHSA2	17	1941	Motor	Muscle contraction.	Myopathies.	ABD (661-683, 763-777) within motor domain	https://www.uniprot.org/uniprot/Q9UKX2	https://www.proteinatlas.org/ENSG00000125414-MYH2	1977	[19, 20]
Myosin-3, Muscle embryonic myosin heavy chain, Myosin heavy chain 3, Myosin heavy chain, fast skeletal muscle, embryonic, SMHCE	MYH3	17	1940	Motor	Muscle contraction.	Myopathies. Freeman-Sheldon syndrome and Sheldon-Hall syndrome.	ABD (656-678, 758-772) within motor domain	https://www.uniprot.org/uniprot/P11055	https://www.proteinatlas.org/ENSG00000109063-MYH3	1986	[21-23]
Myosin-4, Myosin heavy chain 2b (MyHC-2b), Myosin heavy chain, skeletal muscle, fetal	MYH4	17	1939	Motor	Muscle contraction.	Myopathies.	ABD (659-681, 761-775) within motor domain	https://www.uniprot.org/uniprot/Q9Y623	https://www.proteinatlas.org/ENSG00000264424-MYH4	1979	[24]
Myosin-6, Myosin heavy chain 6, Myosin heavy chain, cardiac muscle alpha isoform (MyHC-alpha)	MYH6, MYHCA	14	1939	Motor	Muscle contraction.	Cardiomyopathy. Sick sinus syndrome.	ABD (657-679, 759-773) within motor domain	https://www.uniprot.org/uniprot/P13533	https://www.proteinatlas.org/ENSG00000197616-MYH6	1989	[25, 26]
Myosin-7, Myosin heavy chain 7, Myosin heavy chain slow isoform	MYH7, MYHCB	14	1935	Motor	Muscle contraction.	Cardiomyopathy.	ABD (655-677, 757-771) within motor domain	https://www.uniprot.org/uniprot/P12883	https://www.proteinatlas.org/ENSG00000092054-MYH7	1989	[25]

(MyHC-slow), Myosin heavy chain, cardiac muscle beta (MyHC-beta)											
Myosin-7B, Antigen MLAA-21, Myosin cardiac muscle beta chain, Myosin heavy chain 7B, cardiac muscle beta isoform, Slow A MYH14	MYH7B, KIAA1512	20	1983	Motor	Muscle contraction.	Cardiomyopathy.	ABD (704-726, 806-820) within motor domain	https://www.uniprot.org/uniprot/A7E2Y1	https://www.proteinatlas.org/ENSG00000078814-MYH7B	2001	[27]
Myosin-8, Myosin heavy chain 8, Myosin heavy chain, skeletal muscle, perinatal (MyHC-perinatal)	MYH8	17	1937	Motor	Muscle contraction.	Myopathies.	ABD (658-680, 760-774) within motor domain	https://www.uniprot.org/uniprot/P13535	https://www.proteinatlas.org/ENSG00000133020-MYH8	1983	[28, 29]
Myosin-9, Myosin heavy chain 9, Cellular myosin heavy chain, type A, Myosin heavy chain, non-muscle IIa, Non-muscle myosin heavy chain A (NMMHC-A), Non-muscle myosin heavy chain IIa (NMMHC II-a)	MYH9	22	1960	Motor	Regulate cytokinesis, cell shape, and migration.	Alport syndrome, Cataract, Deafness.	ABD (654-676) within motor domain	https://www.uniprot.org/uniprot/P35579	https://www.proteinatlas.org/ENSG00000100345-MYH9	1983	[28-30]
Myosin-10, Cellular myosin heavy chain, type B, Myosin heavy chain 10, Myosin heavy chain, non-muscle IIb, Non-muscle myosin heavy chain B (NMMHC-B), Non-muscle myosin heavy chain IIb (NMMHC II-b)	MYH10	17	1976	Motor	Regulate cytokinesis, cell shape, and migration.	Intellectual disability, microcephaly, and feeding difficulties as well as cerebral atrophy.	ABD (661-683) within motor domain	https://www.uniprot.org/uniprot/P35580	https://www.proteinatlas.org/ENSG00000133026-MYH10	1983	[28, 31]
Myosin-11, Myosin heavy chain 11, Myosin heavy chain, smooth muscle isoform, SMMHC	MYH11, KIAA0866	16	1972	Motor	Muscle contraction.	Acute myeloid leukemia, aortic aneurysm.	ABD (661-683, 762-776) within motor domain	https://www.uniprot.org/uniprot/P35749	https://www.proteinatlas.org/ENSG00000133392-MYH11	1993	[32]
Myosin-13, Myosin heavy chain 13, Myosin heavy chain, skeletal muscle, extraocular (MyHC-EO), Myosin heavy chain, skeletal muscle, laryngeal (MyHC-IIL), Superfast myosin	MYH13	17	1938	Motor	Muscle contraction.		ABD (659-681, 761-775) within motor domain	https://www.uniprot.org/uniprot/Q9UKX3	https://www.proteinatlas.org/ENSG00000006788-MYH13	1998	[33]
Myosin-14, Myosin heavy chain 14, Myosin heavy chain, non-muscle IIc, Non-muscle myosin heavy chain IIc (NMHC II-C)	MYH14, KIAA2034, FP17425	19	1995	Motor	Regulate cytokinesis, cell shape, and migration.	Deafness, neuropathy.	ABD (678-700) within motor domain	https://www.uniprot.org/uniprot/Q7Z406	https://www.proteinatlas.org/ENSG00000105357-MYH14	2003	[34-36]
Myosin-15, Myosin heavy chain 15	MYH15, KIAA1000	3	1946	Motor	Muscle contraction.	Deafness. Amyotrophic lateral sclerosis.	ABD (667-689, 769-783) within motor domain	https://www.uniprot.org/uniprot/Q9Y2K3	https://www.proteinatlas.org/ENSG00000144821-MYH15	2004	[37, 38]
Myosin-IIIa	MYO3A	10	1616	Motor	Regulate auditory hair bundle.	Deafness. Bardet-Biedl syndrome.	ABD (934-956) within motor domain	https://www.uniprot.org/uniprot/Q8NEV4	https://www.proteinatlas.org/ENSG00000095777-MYO3A	1985	[1, 39, 40]
Myosin-IIIb	MYO3B	2	1341	Motor	Regulate auditory hair bundle.	Deafness. Bardet-Biedl syndrome.	ABD (939-961) within motor domain	https://www.uniprot.org/uniprot/Q8WXR4	https://www.proteinatlas.org/ENSG00000071909-MYO3B	2002	[41]

Unconventional myosin-Va, Dilute myosin heavy chain, non-muscle, Myosin heavy chain 12, Myosin-12, Myoxin	MYO5A, MYH12	15	1855	Motor	Transport melanosome and vesicles.	Griscelli syndrome type 1 and Elejalde syndrome.	ABD (643-665) within motor domain	https://www.uniprot.org/uniprot/Q9Y4I1	https://www.proteinatlas.org/ENSG00000197535-MYO5A	1992	[42, 43]
Unconventional myosin-Vb	MYO5B, KIAA1119	18	1848	Motor	Regulate vesicle transport and trafficking.	Microvillus inclusion disease.	ABD (640-662) within motor domain	https://www.uniprot.org/uniprot/Q9ULV0	https://www.proteinatlas.org/ENSG00000167306-MYO5B	1996	[44]
Unconventional myosin-Vc	MYO5C	15	1742	Motor	Regulate transferrin trafficking.	Diabetic retinopathy.	ABD (632-654) within motor domain	https://www.uniprot.org/uniprot/Q9NQX4	https://www.proteinatlas.org/ENSG00000128833-MYO5C	2002	[45]
Unconventional myosin-VI	MYO6, KIAA0389	6	1294	Motor	Reverse-direction motor protein that moves towards the minus-end of actin filament. Vesicular membrane trafficking and cell migration.	Deafness.	ABD (665-672) within motor domain	https://www.uniprot.org/uniprot/Q9UM54	https://www.proteinatlas.org/ENSG00000196586-MYO6	1994	[10]
Unconventional myosin-VIIa	MYO7A, USH1B	11	2215	Motor	Regulate migration of retinal pigment epithelial melanosomes and phagosomes.	Usher syndrome type III (USH1B).	ABD (632-639) within motor domain	https://www.uniprot.org/uniprot/Q13402	https://www.proteinatlas.org/ENSG00000137474-MYO7A	1994	[10, 46]
Unconventional myosin-VIIb	MYO7B	2	2116	Motor	Regulate microvilli organization.		ABD (637-659) within motor domain	https://www.uniprot.org/uniprot/Q6PIF6	https://www.proteinatlas.org/ENSG00000169994-MYO7B	1994	[10]
Unconventional myosin-IXa	MYO9A, MYR7	15	2548	Motor	Regulate motor neuron axon guidance.	Bardet-Biedl Syndrome. Congenital myasthenic syndrom.	ABD (898-920) within motor domain	https://www.uniprot.org/uniprot/B2RTY4	https://www.proteinatlas.org/ENSG00000066933-MYO9A	1994	[10, 47]
Unconventional myosin-IXb	MYO9B, MYR5	19	2157	Motor	Regulate cell migration through RHOA.	Celiac disease, lcerative colitis and Crohn's disease.	ABD (844-855) within motor domain	https://www.uniprot.org/uniprot/Q13459	https://www.proteinatlas.org/ENSG00000099331-MYO9B	1994	[10]
Unconventional myosin-X	MYO10, KIAA0799	5	2058	Motor	Mediatescargo transport. Regulate cell shape, cell spreading and cell adhesion.	Cancer metastasis and pathogen infection.	ABD (619-641) within motor domain	https://www.uniprot.org/uniprot/Q9HD67	https://www.proteinatlas.org/ENSG00000145555-MYO10	2000	[48-50]
Unconventional myosin-XV, Unconventional myosin-15	MYO15A, MYO15	17	3530	Motor	Transport vesicle.	Deafness	ABD (1792-1799) within motor domain	https://www.uniprot.org/uniprot/Q9UKN7	https://www.proteinatlas.org/ENSG00000091536-MYO15A	1998	[51-53]
Unconventional myosin-XVI, Neuronal tyrosine-phosphorylated phosphoinositide-3-kinase adapter 3	MYO16, KIAA0865, NYAP3, MYO16B	13	1858	Motor	Transport vesicle.	Neurological disorders.	Motor domain (401-1145)	https://www.uniprot.org/uniprot/Q9Y6X6	https://www.proteinatlas.org/ENSG00000041515-MYO16	2001	[54, 55]
Unconventional myosin-XVIIIa, Molecule associated with JAK3 N-terminus (MAJN), Myosin containing a PDZ domain, Surfactant protein receptor SP-R210 (SP-R210)	MYO18A, CD245, KIAA0216, MYSPDZ	17	2054	Motor	Intracellular trafficking.	Myopathies.	Motor domain (405-1185)	https://www.uniprot.org/uniprot/Q92614	https://www.proteinatlas.org/ENSG00000196535-MYO18A	2000	[56, 57]
Unconventional myosin-XVIIIb	MYO18B	22	2567	Motor	Intracellular trafficking.Regulate tumor development and progression.	Myopathies.	Motor domain (571-1333) within motor domain	https://www.uniprot.org/uniprot/Q8IUG5	https://www.proteinatlas.org/ENSG00000133454-MYO18B	2000	[56, 57]
Unconventional myosin-XIX, Myosin head domain-containing protein 1	MYO19, MYOHD1	17	970	Motor	Regulate mitochondrial transport or positioning.		ABD (602-624) within motor domain	https://www.uniprot.org/uniprot/Q96H55	https://www.proteinatlas.org/ENSG00000278259-MYO19	2009	[58]
Myosin light chain kinase, smooth muscle (MLCK, smMLCK), Kinase-related protein (KRP), Telokin	MYLK, MLCK, MLCK1, MYLK1	3	1914		Regulate smooth muscle contraction via phosphorylation of myosin light chains.	Aortic aneurysm, familial thoracic 7 (AAT7), Megacystis-microcolon-intestinal hypoperistalsis syndrome (MMIHS)	DFRxxL motifs of N-terminal	https://www.uniprot.org/uniprot/Q15746	https://www.proteinatlas.org/ENSG00000065534-MYLK	1984	[59-62]
Actin, alpha skeletal muscle, Alpha-actin-1	ACTA1	1	377	Polymerization	Cell shape change, movement, cytokinesis, contraction,signaling.	Skeletal myopathy.	Pointed and barbed ends PDB: 2PBD,	https://www.uniprot.org/uniprot/P68133	https://www.proteinatlas.org/ENSG00000143632-ACTA1	1942	[63-65]

							4PKH, 7CCC				
Actin, alpha cardiac muscle 1, Alpha-cardiac actin	ACTC1, ACTC	15	377	Polymerization	Cell shape change, adhesion, movement, cytokinesis, contraction,signaling.	Cardiomyopathy, dilated 1R (CMD1R), Cardiomyopathy, familial hypertrophic 11 (CMH11), Atrial septal defect 5 (ASD5).	Pointed and barbed ends PDB: 7LRG	https://www.uniprot.org/uniprot/P68032	https://www.proteinatlas.org/ENSG00000159251-ACTC1	1949	[63-66]
Actin, aortic smooth muscle, Alpha-actin-2	ACTA2, ACTSA, ACTVS	10	377	Polymerization	Cell shape change, adhesion, movement, cytokinesis, contraction,signaling	Aortic aneurysm, familial thoracic 6 (AAT6), Moyamoya disease 5 (MYMY5), Multisystemic smooth muscle dysfunction syndrome (MSMDS).	Pointed and barbed ends	https://www.uniprot.org/uniprot/P62736	https://www.proteinatlas.org/ENSG00000107796-ACTA2	1986	[63-65, 67]
Actin, cytoplasmic 1, Actin Bata	ACTB	7	375	Polymerization	Cell shape change, adhesion, movement, cytokinesis, contraction,signaling	Dystonia, juvenile-onset (DJO), Baraitser-Winter syndrome 1 (BRWS1).	Pointed and barbed ends	https://www.uniprot.org/uniprot/P60709	https://www.proteinatlas.org/ENSG00000075624-ACTB	1949	[63-65, 68]
Actin, cytoplasmic 2, Gamma-actin	ACTG1, ACTG	17	375	Polymerization	Cell shape change, adhesion, movement, cytokinesis, contraction,signaling	Deafness, autosomal dominant, 20 (DFNA20), Baraitser-Winter syndrome 2 (BRWS2).	Pointed and barbed ends	https://www.uniprot.org/uniprot/P63261	https://www.proteinatlas.org/ENSG00000184009-ACTG1	1976	[63-65, 69, 70]
Actin, gamma-enteric smooth muscle, Alpha-actin-3, Gamma-2-actin, Smooth muscle gamma-actin	ACTG2, ACTA3, ACTL3, ACTSG	2	376	Polymerization	Cell shape change, adhesion, movement, cytokinesis, contraction,signaling	Visceral myopathy (VSCM).	Pointed and barbed ends	https://www.uniprot.org/uniprot/P63267	https://www.proteinatlas.org/ENSG00000163017-ACTG2	1981	[63-65, 71]
Thymosin beta-4, T beta-4, Fx	TMSB4X, TB4X, THYB4, TMSB4	X	44	Monomer binding	Inhibit actin polymerization	Chronic kidney disease, acute stroke, coronary heart disease, Progressive Neurodegenerative Diseases.	WH2 PDB: 2A3Z	https://www.uniprot.org/uniprot/P62328	https://www.proteinatlas.org/ENSG00000205542-TMSB4X	1978	[72-74]
Thymosin beta-4, Y-chromosomal	TMSB4Y, TB4Y	Y	44	Monomer binding	Inhibit actin polymerization. Regulate cellular morphology and proliferation.	Laryngeal, breast, prostate cancer.	WH2	https://www.uniprot.org/uniprot/O14604	https://www.proteinatlas.org/ENSG00000154620-TMSB4Y	1991	[72-75]
Thymosin beta-10	TMSB10, PTMB10, THYB10	2	44	Monomer binding	Inhibit actin polymerization. Regulate cell proliferation, migration, and invasion.	Sjögren's syndrome, papillary thyroid carcinoma, hepatocellular carcinoma, neuroblastoma, ovarian, pancreatic, melanoma, renal cell carcinoma, gastric, breast, lung, and thyroid cancers.	WH2	https://www.uniprot.org/uniprot/P63313	https://www.proteinatlas.org/ENSG00000034510-TMSB10	1983	[73, 74, 76]
Thymosin beta-15A, NB thymosin beta, Thymosin-like protein 8	TMSB15A, TMSL8, T MSNB	X	45	Monomer binding	Inhibit actin polymerization	Breast, colon, prostate cancers; Uterine leiomyoma; paediatric aplastic anaemia.	WH2	https://www.uniprot.org/uniprot/P0CG34	https://www.proteinatlas.org/ENSG00000158164-TMSB15A	1996	[73, 74, 77]
Thymosin beta-15B	TMSB15B	X	45	Monomer binding	Inhibit actin polymerization	Colon, prostate cancers, glioblastoma.	WH2	https://www.uniprot.org/uniprot/P0CG35	https://www.proteinatlas.org/ENSG00000158427-TMSB15B	1996	[73, 74, 77]
Profilin-1, Profilin I	PFN1	17	140	Monomer binding	At high concentrations, profilin prevents the polymerization of actin, whereas it enhances it at low concentrations. By binding to PIP2, it inhibits the formation of IP3 and DG. Bind to proline-rich proteins.	Amyotrophic lateral sclerosis (ALS), coronary artery disease, Fragile X syndrome (FXS), Glioblastoma, breast cancer, renal cell carcinoma (RCC), bladder cancer, pancreatic cancer, vascular hyperpermeability, hypertension/vascular hypertrophy, adipose tissue inflammation, atherosclerosis.	FH1, FH2 PDB: 2PBD	https://www.uniprot.org/uniprot/P07737	https://www.proteinatlas.org/ENSG00000108518-PFN1	1977	[78-80]
Profilin-2, Profilin II	PFN2	3	140	Monomer binding	At high concentrations, profilin prevents the polymerization of actin, whereas it enhances it at low concentrations. By binding to PIP2, it inhibits the formation of IP3 and DG.	Allergies, cardiovascular disease, Amyotrophic lateral sclerosis (ALS), Huntington's disease, Alzheimer's disease, diabetes, and lung, breast, and colorectal cancers, spinal muscular atrophy.	FH1, FH2 PDB: 1D1J	https://www.uniprot.org/uniprot/P35080	https://www.proteinatlas.org/ENSG00000070087-PFN2	1993	[80, 81]

Profilin-3, Profilin III	PFN3	5	137	Monomer binding	Filament elongation	Glioma, type II globozoospermia.	FH1, FH2	https://www.uniprot.org/uniprot/P60673	https://www.proteinatlas.org/ENSG00000196570-PFN3	2002	[80, 82]
Formin-1, Limb deformity protein homolog	FMN1, FMN, LD	15	1419	Nucleation, polymerization, bundling, Severing	Cell movement, shape change	X-linked Alport syndrome, autism spectrum disorder, schizophrenia, Cenani–Lenz-like non-syndromic, hearing loss, congenital anomalies of the kidney and urinary, melanoma, pancreatic, prostate cancer.	FH2 PDB: 1UX5	https://www.uniprot.org/uniprot/Q68DA7	https://www.proteinatlas.org/ENSG00000248905-FMN1	1990	[83, 84]
Formin-2	FMN2	1	1722	Nucleation, polymerization, bundling	Cell movement, shape change	Intellectual disability and neurodevelopmental disorders, premature ovarian failure and infertility, Alzheimer disease, colorectal, brain, pancreatic, glioma and testicular cancer.	FH2	https://www.uniprot.org/uniprot/Q9NZ56	https://www.proteinatlas.org/ENSG00000155816-FMN2	2000	[84, 85]
Formin-like protein 1, CLL-associated antigen KW-13, Leukocyte formin	FMNL1, C17orf1, C17orf1B, FMNL, FRL1	17	1100	Nucleation, polymerization, severing	Cell movement, shape change	Osteocarcinoma, lymphoma, leukemia, brain, head and neck, lung, ovarian, pancreatic, gastric cancer, renal carcinoma.	FH2	https://www.uniprot.org/uniprot/O95466	https://www.proteinatlas.org/ENSG00000184922-FMNL1	2003	[86-88]
Formin-like protein 2, Formin homology 2 domain-containing protein 2	FMNL2, FHOD2, KIAA1902	2	1086	Nucleation, polymerization	Cell movement, shape change, Proliferation, regulate Golji architecture.	Adenomyosis, mental retardation, POEMS syndrome, Crohn's disease, brain, colorectal, ovarian, gastric cancer, gallbladder carcinoma, hepatocellular carcinoma, melanoma, early-onset stroke, glaucoma.	FH2	https://www.uniprot.org/uniprot/Q96PY5	https://www.proteinatlas.org/ENSG00000157827-FMNL2	2003	[86, 87, 89]
Formin-like protein 3, Formin homology 2 domain-containing protein 3, WW domain-binding protein 3	FMNL3, FHOD3, FRL2, KIAA2014, WBP3	12	1028	Nucleation, polymerization	Cell movement, shape change, Proliferation, regulate Golji architecture.	Gastric, pancreatic, colorectal, head and neck, ovarian, prostate cancer, dermatophyma, nasopharyngeal carcinoma, peripheral neurodegenerative disorder.	FH2	https://www.uniprot.org/uniprot/Q8IVF7	https://www.proteinatlas.org/ENSG00000161791-FMNL3	2003	[86, 87, 89]
FH1/FH2 domain-containing protein 1, Formin homolog overexpressed in spleen 1, Formin homology 2 domain-containing protein 1	FHOD1, FHOS, FHOS1	16	1164	Polymerization	Organize actin filaments	Gastric, brain, breast, head and neck, renal cancer, dermatophyma, lung, oral squamous carcinoma, impaired cardiac.	FH2	https://www.uniprot.org/uniprot/Q9Y613	https://www.proteinatlas.org/ENSG00000135723-FHOD1	1999	[90, 91]
FH1/FH2 domain-containing protein 3, Formactin-2, Formin homolog overexpressed in spleen 2	FHOD3, FHOS2, KIAA1695	18	1422	Polymerization	Organize actin filaments	Brain, ovarian, thyroid cancer, leukemia, hypertrophic and dilated cardiomyopathies, type I diabetes, periodontal disease.	FH2	https://www.uniprot.org/uniprot/Q2V2M9	https://www.proteinatlas.org/ENSG00000134775-FHOD3	2004	[92, 93]
Disheveled-associated activator of morphogenesis 1	DAAM1, KIAA0666	14	1078	Nucleation, polymerization	Cell movement, shape change	Brain, breast, colorectal, esophagus, osteocarcinoma, dermatophyma, congenital heart defects, anomalies of the kidney and urinary tract, cerebral palsy.	FH2 PDB: 2Z6E	https://www.uniprot.org/uniprot/Q9Y4D1	https://www.proteinatlas.org/ENSG00000100592-DAAM1	2001	[94]
Dishevelled associated activator of morphogenesis 2	DAAM2, KIAA0381	6	1068	Nucleation, polymerization	Cell movement, shape change	Brain, Cervix, colorectal, lung cancer, renal carcinoma, hepatocellular carcinoma, schizophrenia, Guillain–Barre syndrome, diffuse pulmonary ossification, diabetic nephropathy, osteoporosis, melanoma.	FH2	https://www.uniprot.org/uniprot/Q86T65	https://www.proteinatlas.org/ENSG00000146122-DAAM2	2003	[95]
Protein diaphanous homolog 1,	DIAPH1, DIAP1	5	1272	Nucleation	Actin polymerization. Regulate spindle	Deafness, autosomal dominant 1	FH2, FH1-FH2 domain	https://www.uniprot.org/uniprot/O60610	https://www.proteinatlas.org/ENSG0	1997	[96-98]

Diaphanous-related formin-1, mammalian Diaphanous-related formin (mDia1)					formation and cell division.	(DFNA1), Seizures, cortical blindness, and microcephaly syndrome (SCBMS), neuroinflammation and neurodegenerative diseases, brain, breast, colorectal, esophagus, head and neck, ovarian cancer, glioma, leukemia, dermatophyma, Mendelian disorder, thrombocytopenia, Alzheimer disease, diabetes-associated nephropathy, ischemic stroke, Moyamoya disease.			0000131504-DIAPH1		
Protein diaphanous homolog 2, Diaphanous-related formin-2	DIAPH2, DIA	X	1101	Nucleation	Actin polymerization. Regulate formation of lamellipodia and filopodia.	Premature ovarian failure 2A, breast, colorectal, head and neck, renal, lung cancer; age-related macular degeneration, premature ovarian failure.	FH2	https://www.uniprot.org/uniprot/O60879	https://www.proteinatlas.org/ENSG00000147202-DIAPH2	1998	[98, 99]
Protein diaphanous homolog 3, Diaphanous-related formin-3	DIAPH3, DIAP3	13	1193	Nucleation	Actin polymerization. Regulate cytokinesis and microtubule dynamics.	Hepatocellular carcinoma, pancreatic, brain, breast, colorectal, head and neck, lung, ovarian, prostate, auditory neuropathy spectrum disorders, neurodevelopmental disorders, infantile spasm, epileptic encephalopathy.	FH2	https://www.uniprot.org/uniprot/Q9NSV4	https://www.proteinatlas.org/ENSG00000139734-DIAPH3	2004	[98, 100-102]
Inverted formin-2, HBEBP2-binding protein C	INF2, C14orf151, C14orf173	14	1249	Severing	Essential for proper placentation. Accelerates actin polymerization and depolymerization.	Neurodegeneration, Neuropathy, Focal segmental glomeruloesclerosis (FSGS), Charcot–Marie–Tooth (CMT) disease, intellectual disability and neurodevelopmental disorders, brain, breast, prostate, colorectal, thyoid cancer, thrombocytopenia, auditory nerve damage, Focal segmental glomerulosclerosis (FSGS), steroid-resistant nephrotic syndrome.	WH2, FH2	https://www.uniprot.org/uniprot/Q27J81	https://www.proteinatlas.org/ENSG00000203485-INF2		[103-105]
Protein cordon-bleu	COBL, KIAA0633	7	1261	Nucleation	Crucial for neuromorphogenesis processes	Acute lymphoblastic leukemia, Alzheimer's disease, pulmonary neuroendocrine carcinomas, lung cancer, lung squamous cell carcinoma, type 1 diabetes, head and neck squamous cell carcinoma, Huntington's disease, renal cell carcinoma.	Three WH2 PDB: 4JHD	https://www.uniprot.org/uniprot/O75128	https://www.proteinatlas.org/ENSG00000106078-COBL	1995	[106-108]
Junction-mediating and -regulatory protein	JMY	5	988	Nucleation	A regulator of both transcription and actin polymerization	Cardiovascular Disease, ankylosing spondylitis, glioblastoma, pancreatic cancer.	Tandem WH2	https://www.uniprot.org/uniprot/Q8N9B5	https://www.proteinatlas.org/ENSG00000152409-JMY	1999	[109]
Leiomodin-1, 64 kDa autoantigen 1D, 64 kDa autoantigen 1D3, 64 kDa autoantigen D1, Leiomodin, muscle form, Smooth muscle leiomodin (SM-Lmod), Thyroid-associated ophthalmopathy autoantigen	LMOD1	1	600	Nucleation	Bind along F-actin, next to the pointed end. Involved in sarcomere assembly and organization.	Visceral myopathy, megacystis microcolon intestinal hypoperistalsis syndrome.	ABS1, ABS2 (LRR domain), ABS3 (WH2 domain)	https://www.uniprot.org/uniprot/P29536	https://www.proteinatlas.org/ENSG00000163431-LMOD1	2001	[110, 111]

Leiomodin-2, Cardiac leiomodin (C-LMOD), Leiomodin	LMOD2	7	547	Nucleation	Bind along F-actin, next to the pointed end. Involved in sarcomere assembly and organization.	Neonatal dilated cardiomyopathy.	ABS1, ABS2 (LRR domain), ABS3 (WH2 domain)	https://www.uniprot.org/uniprot/Q6P5Q4	https://www.proteinatlas.org/ENSG00000170807-LMOD2	2001	[110, 111]
Leiomodin-3, Leiomodin, fetal form	LMOD3	3	560	Nucleation	Bind along F-actin, next to the pointed end. Involved in sarcomere assembly and organization.	Nemaline myopathy	ABS1, ABS2 (LRR domain), ABS3 (WH2 domain)	https://www.uniprot.org/uniprot/Q0VAK6	https://www.proteinatlas.org/ENSG00000163380-LMOD3	2012	[111, 112]
Arp2/3 complex (Actin-related protein 2, Actin-related protein 3, Actin-related protein 3B, Actin-related protein 2/3 complex subunit 1A, Actin-related protein 2/3 complex subunit 1B, Actin-related protein 2/3 complex subunit 2, Actin-related protein 2/3 complex subunit 3, Actin-related protein 2/3 complex subunit 4, Actin-related protein 2/3 complex subunit 5)	ACTR2, ACTR3, ACTR3B, ARPC1A, ARPC1B, ARPC2, ARPC3, ARPC4, ARPC5	2 2 7 7 7 2 12 3 1	394 418 418 370 372 300 178 168 151	Nucleation, branching, capping	Generates branched actin networks. Regulate actin-based cell motility.	Lung, breast, gliomas, gastric, pancreatic and colorectal cancers, psoriasis-like disease, psoriasis-like disease, intervertebral disc and cartilage degeneration, chronic obstructive pulmonary disease, Hirschsprung disease.		https://www.uniprot.org/uniprot/P61160 https://www.uniprot.org/uniprot/P61158 https://www.uniprot.org/uniprot/Q92747 https://www.uniprot.org/uniprot/O15143 https://www.uniprot.org/uniprot/Q9P1U1 https://www.uniprot.org/uniprot/O15144 https://www.uniprot.org/uniprot/O15145 https://www.uniprot.org/uniprot/P59998 https://www.uniprot.org/uniprot/O15511	https://www.proteinatlas.org/ENSG00000138071-ACTR2 https://www.proteinatlas.org/ENSG00000115091-ACTR3 https://www.proteinatlas.org/ENSG00000133627-ACTR3B https://www.proteinatlas.org/ENSG00000241685-ARPC1A https://www.proteinatlas.org/ENSG00000130429-ARPC1B https://www.proteinatlas.org/ENSG00000163466-ARPC2 https://www.proteinatlas.org/ENSG00000111229-ARPC3 https://www.proteinatlas.org/ENSG00000241553-ARPC4 https://www.proteinatlas.org/ENSG00000162704-ARPC5	1997	[108, 113-116]
Actin-related protein 3C, Actin-related protein 11	ACTR3C, ARP11	7	210	Capping	Decreases the formation of the Arp1 assemblies. Cap pointed end.			https://www.uniprot.org/uniprot/Q9C0K3	https://www.proteinatlas.org/ENSG00000106526-ACTR3C	1999	[117, 118]
Wiskott-Aldrich syndrome protein, WASp	WAS, IMD2	X	502	Polymerization, cross-linking	Effector protein for Rho-type GTPases, interaction with the Arp2/3 complex. Promote actin polymerization in the nucleus.	Wiskott-Aldrich syndrome, Thrombocytopenia 1, Neutropenia, severe congenital, X-linked (XLN).	WH1, WH2	https://www.uniprot.org/uniprot/P42768	https://www.proteinatlas.org/ENSG00000015285-WAS	1996	[119]
Wiskott-Aldrich syndrome protein family member 1 (WASP family protein member 1), Protein WAVE-1, Verprolin homology domain-containing protein 1	WASF1, KIAA0269, SCAR1, WAVE1	6	559	Monomer binding	The WAVE complex regulates actin filament reorganization via its interaction with the Arp2/3 complex. It restricts actin network extension at the leading edge.	Alzheimer's disease, acute myeloid leukemia, oral squamous cell carcinoma, breast, prostate, ovarian, melanoma cancer.	WH2	https://www.uniprot.org/uniprot/Q92558	https://www.proteinatlas.org/ENSG00000112290-WASF1	1998	[120, 121]
WAS protein family member 2, Verprolin homology domain-containing protein 2	WASF2, WAVE2	1	498	Monomer binding	The WAVE complex regulates actin filament reorganization via its interaction with the Arp2/3 complex. It promotes actin network extension at the leading edge.	Lung, liver, pancreatic, prostate, colorectal and breast, cervical cancers, left-sided obstructive heart defects, osteosarcoma, melanoma, Parkinson's disease.	WH2	https://www.uniprot.org/uniprot/Q9Y6W5	https://www.proteinatlas.org/ENSG00000158195-WASF2	1999	[121, 122]
Neural Wiskott-Aldrich syndrome protein, N-WASP	WASL	7	505	Monomer binding, Depolymerization	Stimulating the actin-nucleating activity of the Arp2/3 complex; bind with CDC42, involved in the extension and maintenance of the formation filopodia.	Parkinson's disease, Wiskott-Aldrich syndrome, hepatocellular carcinoma, polycystic kidney disease, esophageal cancer, gastric cancer, proteinuria kidney disease, breast cancer.	WH1, WH2	https://www.uniprot.org/uniprot/O00401	https://www.proteinatlas.org/ENSG00000106299-WASL	1996	[123]
WAS/WASL-interacting protein family member 1, Protein PRPL-2, Wiskott-Aldrich syndrome protein-	WIPF1, WASPIP, WIP	2	503	Monomer binding	Activation of N-WASP; activator of Arp2/3.	Wiskott-Aldrich syndrome 2.	WH2	https://www.uniprot.org/uniprot/O43516	https://www.proteinatlas.org/ENSG00000115935-WIPF1	2001	[124]

interacting protein											
WAS/WASL-interacting protein family member 2, WASP-interacting protein-related protein, WIP- and CR16-homologous protein, WIP-related protein	WIPF2, WICH, WIRE	17	440	Stabilization, Bunding	Cooperate with WASP and N-WASP to induce mobilization and reorganization of the actin filament system.	Parkinson's disease, hepatocellular carcinoma, breast cancer.	WH2	https://www.uniprot.org/uniprot/Q8TF74	https://www.proteinatlas.org/ENSG00000171475-WIPF2	2002	[125-127]
WAS/WASL-interacting protein family member 3, Corticosteroids and regional expression protein 16 homolog	WIPF3, CR16	7	483	Monomer binding	Cooperate with N-WASP to regulate actin polymerization.	Abdominal aortic aneurysm.	WH2	https://www.uniprot.org/uniprot/A6NGB9	https://www.proteinatlas.org/ENSG00000122574-WIPF3	2001	[128]
Tropomodulin 1, Erythrocyte tropomodulin	TMOD1, D9S57E, ETMOD, TMOD	9	359	Capping	Capping the pointed end. Interaction with tropomyosin stabilizes thin filaments in cardiac myocytes.	Breast cancer, glaucomatous retina, neurological diseases, diabetic kidney disease, chronic obstructive pulmonary disease, lung adenocarcinoma, congenital myopathy, meningiomas, acute lymphoblastic leukemia, ulcerative colitis (UC), Duchenne muscular dystrophy (DMD)	ABS1, ABS2	https://www.uniprot.org/uniprot/P28289	https://www.proteinatlas.org/ENSG00000136842-TMOD1	1987	[129-132]
Tropomodulin 2, Neuronal tropomodulin	TMOD2, NTMOD	15	351	Capping	Capping the pointed end. Regulate behavior, learning, memory, and synaptic plasticity.	Neurological diseases, Idiopathic pulmonary fibrosis (IPF), first-episode schizophrenia.	ABS1, ABS2	https://www.uniprot.org/uniprot/Q9NZR1	https://www.proteinatlas.org/ENSG00000128872-TMOD2	1996	[132-135]
Tropomodulin 3, Ubiquitous tropomodulin	TMOD3, UTMOD	15	352	Capping	Capping the pointed end. Regulate cell morphology, motility, and division.	Liver cancer, neurological diseases.	ABS1, ABS2	https://www.uniprot.org/uniprot/Q9NYL9	https://www.proteinatlas.org/ENSG00000138594-TMOD3	2000	[135-137]
Tropomodulin 4, Skeletal muscle tropomodulin	TMOD4, Sk-Tmod	1	345	Capping	Capping the pointed end. Skeletal muscle development.	Neurological diseases, myocardial infarction, early-onset dyslipidemia, primary auditory neurons degeneration (PAND).	ABS1, ABS2	https://www.uniprot.org/uniprot/Q9NZQ9	https://www.proteinatlas.org/ENSG00000163157-TMOD4	1999	[135, 136, 138, 139]
Adseverin,<br><br>Scinderin	SCIN, KIAA1905	7	715	Severing, Capping, Nucleation	F-actin depolymerization. Regulate cell proliferation. Gelsolin superfamily protein.	periodontal disease, bladder cancer, hepatocellular carcinoma, gastric cancer, Colorectal Cancer, breast cancer, acute myeloid leukemia, prostate cancer, melanoma, multiple sclerosis, head and neck cancer.	Gelsolin-like domain	https://www.uniprot.org/uniprot/Q9Y6U3	https://www.proteinatlas.org/ENSG00000006747-SCIN	1990	[140]
Advillin, p92	AVIL	12	819	Bunding	Regulate morphogenesis of neuronal cells. Gelsolin superfamily protein	Steroid-resistant nephrotic syndrome, glioblastoma.	Headpiece domain	https://www.uniprot.org/uniprot/O75366	https://www.proteinatlas.org/ENSG00000135407-AVIL	1998	[141, 142]
Supervillin, Archvillin, p205/p250	SVIL	10	2214	Bunding	Regulate actin dynamics. Gelsolin superfamily protein.	Liver cancer, myopathy, prostate cancer, Lymphangioleiomyomatosis.	Gelsolin-like domain	https://www.uniprot.org/uniprot/O95425	https://www.proteinatlas.org/ENSG00000197321-SVIL	1997	[143]
Twinfilin-1, protein A6, protein tyrosine kinase 9	TWF1, PTK9	12	350	Monomer binding, Severing	Inhibit actin polymerization. Regulate motility.	Ocular coloboma, pancreatic cancer, breast cancer, lung adenocarcinoma, hepatocellular carcinoma, coronary artery disease.	ADF-H	https://www.uniprot.org/uniprot/Q12792	https://www.proteinatlas.org/ENSG00000151239-TWF1	1998	[144]
Twinfilin-2, A6-related protein, Twinfilin-1-like protein	TWF2, PTK9L, MSTP011	3	349	Monomer binding	Inhibit actin polymerization by sequestering actin monomers and capping filament barbed ends.	Pancreatic cancer.	ADF-H	https://www.uniprot.org/uniprot/Q6IBS0	https://www.proteinatlas.org/ENSG00000247596-TWF2	2003	[145, 146]
Drebrin-like protein, Cervical SH3P7, Cervical mucin-associated	DBNL, CMAP, SH3P7	7	430	Depolymerization	Reorganize the actin cytoskeleton.		ADF-H	https://www.uniprot.org/uniprot/Q9UJU6	https://www.proteinatlas.org/ENSG00000136279-DBNL	1999	[147, 148]

protein, Drebrin-F, HPK1-interacting protein of 55 kDa											
Adenylyl cyclase-associated protein 1	CAP1, CAP	1	475	Monomer binding	Rapid actin filament depolymerization.	Head and neck squamous cell carcinomas. Chronic kidney disease.	C-CAP	https://www.uniprot.org/uniprot/Q01518	https://www.proteinatlas.org/ENSG00000131236-CAP1	1992	[149-152]
Adenylyl cyclase-associated protein 2	CAP2	6	477	Monomer binding	Blocking G-actin, rapid actin filament depolymerization.	Gliomas.	C-CAP	https://www.uniprot.org/uniprot/P40123	https://www.proteinatlas.org/ENSG00000112186-CAP2	1994	[153]
Erythroid spectrin Spectrin (Spectrin alpha chain, erythrocytic 1, Erythroid alpha-spectrin; Spectrin beta chain, erythrocytic; Beta-I spectrin)	SPTA1, SPTA; SPTB1	1<br>14	2419<br>2137	Bundling, Anchoring, Nucleation	Maintains cell membrane integrity and its mechanical properties. involve in cell adhesion and spreading, form lamellipodia, and also participate in morphogenetic processes.	Duchenne Muscular Dystrophy, Hereditary Spherocytosis, hereditary elliptocytosis, hereditary pyropoikilocytosis,	CH1, CH2	https://www.uniprot.org/uniprot/P02549 https://www.uniprot.org/uniprot/P11277	https://www.proteinatlas.org/ENSG00000163554-SPTA1 https://www.proteinatlas.org/ENSG00000070182-SPTB	1975	[154-158]
Non-erythrocytic spectrin, fodrin (Spectrin alpha chain, non-erythrocytic 1, Alpha-II spectrin, Fodrin alpha chain, Spectrin, non-erythroid alpha subunit; Spectrin beta chain, non-erythrocytic 1; Beta-II spectrin; Fodrin beta chain; Spectrin, non-erythroid beta chain 1)	SPTAN1, NEAS, SPTA2; SPTBN1, SPTB2	9<br>2	2472<br>2364	Bundling, Anchoring, Nucleation	Maintenance of structural integrity in mammalian cells, which is necessary for proper cell function.	Moyamoya disease (MMD), Colorectal, gastric, lung, breast, prostate, ovatian cancers, cutaneous, soft tissue tumors, non-Hodgkin lymphoma, acute lymphocytic leukemia.	CH1, CH2	https://www.uniprot.org/uniprot/Q13813 https://www.uniprot.org/uniprot/Q01082	https://www.proteinatlas.org/ENSG00000115306-SPTBN1	1981	[157, 159, 160]
Ezrin, Cytovillin, Villin-2, p81	EZR, VIL2	6	586	Anchoring	Crosslinker of actin with membrane; stimulate the actin polymerization.	Breast, lung and prostate cancers, oral squamous cell carcinomas (OSCCS).	C-terminal ABD	https://www.uniprot.org/uniprot/P15311	https://www.proteinatlas.org/ENSG00000092820-EZR	1983	[161-163]
Radixin	RDX	11	583	Anchoring, Capping	Capping the barbed end; inhibit the actin polymerization.	Prostate cancer.	C-terminal ABD	https://www.uniprot.org/uniprot/P35241	https://www.proteinatlas.org/ENSG00000137710-RDX	1989	[163]
Moesin, Membrane-organizing extension spike protein	MSN	X	577	Anchoring	Crosslinker of actin with membrane.	Oral squamous cell carcinomas (OSCCS).	C-terminal ABD	https://www.uniprot.org/uniprot/P26038	https://www.proteinatlas.org/ENSG00000147065-MSN	1988	[164]
Merlin, Moesin-ezrin-radixin-like protein, Neurofibromin-2, Schwannomerlin, Schwannomin	NF2, SCH	22	595	Anchoring, Stabilization	Tumor suppressor.	Neurofibromatosis type 2 (NF2), multiple nervous system tumors, vestibular and spinal schwannomas, meningiomas and ependymomas, mesotheliomas, breast, prostate, colorectal, hepatic, clear cell renal cell carcinoma, and melanomas.	N-terminal ABD (178-367)	https://www.uniprot.org/uniprot/P35240	https://www.proteinatlas.org/ENSG00000186575-NF2	1993	[165-168]
Protein phosphatase Slingshot homolog 1, SSH-like protein 1	SSH1, KIAA1298, SSH1L	12	1049	Stabilization, Bundling	Dephosphorylate and activate cofilin.	Overexpressed in some cancers. Disseminated superficial actinic porokeratosis.	PH-like domain	https://www.uniprot.org/uniprot/Q8WYL5	https://www.proteinatlas.org/ENSG00000084112-SSH1	2002	[169-172]
Coronin-1A, Coronin-like protein A, Tryptophan aspartate-containing coat protein	CORO1A, CORO1	16	461	Stabilization, Bundling	Disassemble actin filament branches. Regulate phagocytosis, locomotion, and cytokinesis.	Immunodeficiency 8 (IMD8), severe combined immunodeficiency (SCID), systemic lupus erythematosus (SLE), multiple sclerosis (MS), neurocognitive and behavioral abnormal, pathogenic infection.	N- and C-terminal	https://www.uniprot.org/uniprot/P31146	https://www.proteinatlas.org/ENSG00000102879-CORO1A	1991	[173-175]
Coronin-1B, Coronin-2	CORO1B	11	489	Stabilization, Bundling	Controls actin networks at classical lamellipodia.		N-terminal (Arg30 is crucial)	https://www.uniprot.org/uniprot/Q9BR76	https://www.proteinatlas.org/ENSG00000172725-CORO1B	1998	[175-177]
Coronin 1C, Coronin-3, hCRNN4	CORO1C, HCRNN4	12	474	Stabilization, Bundling	Regulate cell migration and metastasis.	Gastric, colorectal, breast cancers, lung squamous cell carcinoma, renal cell cancer, Diffuse Gliomas, Hepatocellular Carcinoma (HCC).	C-terminal	https://www.uniprot.org/uniprot/Q9ULV4	https://www.proteinatlas.org/ENSG00000110880-CORO1C	1998	[175, 176, 178-180]

Coronin-2A, IR10, WD repeat-containing protein 2, Coronin-4	CORO2A, IR10, WDR2	9	525	Stabilization, Bundling	Regulate focal adhesion turnover. Bind to the promoter of NCoR target gene.	Inflammatory.		https://www.uniprot.org/uniprot/Q92828	https://www.proteinatlas.org/ENSG00000106789-CORO2A	1996	[175, 181]
Coronin-2B, Coronin-like protein C (Clipin-C), Protein FC96, Coronin-5	CORO2B	15	480	Stabilization, Bundling	Limiting the speed of actin polymerization.	Diabetic nephropathy (DN).	N-terminal	https://www.uniprot.org/uniprot/Q9UQ03	https://www.proteinatlas.org/ENSG00000103647-CORO2B	1999	[175, 182, 183]
Coronin-6, Coronin-like protein E (Clipin-E)	CORO6	17	472	Anchoring	Anchor acetylcholine receptors to actin.	Congenital myasthenic syndrome (CMS).	N-terminal	https://www.uniprot.org/uniprot/Q6QEF8	https://www.proteinatlas.org/ENSG00000167549-CORO6	2014	[175, 184]
Coronin-7 (Crn7), 70 kDa WD repeat tumor rejection antigen homolog	CORO7	16	925	Stabilization	Facilitate vesicular trafficking. Regulate Golgi structure.	Obesity.		https://www.uniprot.org/uniprot/P57737	https://www.proteinatlas.org/ENSG00000262246-CORO7	2004	[175, 185, 186]
Src substrate cortactin, Amplaxin, Oncogene EMS1	CTTN, EMS1	11	550	Stabilization	Stabilize new filament branch points. directly activate Arp2/3, form microspikes.	Head and neck squamous cell carcinoma (HNSCC), oral squamous cell carcinoma, lung squamous cell carcinoma, gliosarcoma, breast cancer, colorectal cancer and melanoma, leukemia, cardiovascular diseases (CVDs), cerebral cavernous malformations (CCM), inflammatory Bowel diseases, acute lung injury, Alzheimer's disease (AD), hypogonadatropic hypogonadism (HH), myositis.	6.5 tandem repeats	https://www.uniprot.org/uniprot/Q14247	https://www.proteinatlas.org/ENSG00000085733-CTTN	1991	[187, 188]
Hematopoietic lineage cell-specific protein, Hematopoietic cell-specific LYN substrate 1, LckBP1, p75	HCLS1, HS1	3	486	Stabilization	Stabilizing newly formed branched actin networks.	Chronic lymphocytic leukemia (CLL), Systemic lupus erythematosus (SLE), severe congenital neutropenia (SCN), Alzheimer's disease (AD).	Repetitive tandem repeats and the coiled-coil (CC) domain	https://www.uniprot.org/uniprot/P14317	https://www.proteinatlas.org/ENSG00000180353-HCLS1	1989	[189, 190]
F-actin-capping protein subunit alpha-1, CapZ alpha-1	CAPZA1	1	286	Capping	Blocking the exchange of subunits at barbed end.	Gastric cancer, asthma, chronic obstructive pulmonary disease (COPD), pancreatic cancer.		https://www.uniprot.org/uniprot/P52907	https://www.proteinatlas.org/ENSG00000116489-CAPZA1	1989	[191]
F-actin-capping protein subunit alpha-2, CapZ alpha-2	CAPZA2	7	286	Capping	Blocking the exchange of subunits.	Non-syndromic neurodevelopmental disorder in children.		https://www.uniprot.org/uniprot/P47755	https://www.proteinatlas.org/ENSG00000198898-CAPZA2	1986	[192]
F-actin-capping protein subunit alpha-3, CapZ alpha-3	CAPZA3, CAPPA3, GSG3	12	299	Capping	Controlling actin polymerization during spermiogenesis.	Male infertility		https://www.uniprot.org/uniprot/Q96KX2	https://www.proteinatlas.org/ENSG00000177938-CAPZA3	2002	[193]
F-actin-capping protein subunit beta, CapZ beta	CAPZB	1	277	Capping	Blocks actin polymerizationand depolymerization at the fast growing (barbed) filament ends.	Alzheimer's disease; ovarian cancer; sporadic amyotrophic lateral sclerosis and frontotemporal lobar degeneration comorbidity (ALS/FTD).		https://www.uniprot.org/uniprot/P47756	https://www.proteinatlas.org/ENSG00000077549-CAPZB	1995	[194]
Destrin, Actin-depolymerizing factor (ADF)	DSTN, ACTDP, DSN	20	165	Severing	Actin depolymerizing. Sever actin filaments.	Lung adenocarcinoma, Alzheimer's disease and ischemic kidney disease.	ADF-H	https://www.uniprot.org/uniprot/P60981	https://www.proteinatlas.org/ENSG00000125868-DSTN	1980	[195, 196]
Gelsolin, AGEL, Actin-depolymerizing factor	GSN	9	782	Capping, Severing, Nucleation	Prevents further monomer binding and eventually depolymerizes actin filament; promote the polymerization of monomers into filaments (nucleation) as well as sever filaments.	Cancer, amyloidosis, rheumatoid arthritis, AD, autoimmune diseases, chronic kidney disease, diabetes type 2.	G1, G2 and G4	https://www.uniprot.org/uniprot/P06396	https://www.proteinatlas.org/ENSG00000148180-GSN	1979	[197]
Villin-1	VIL1, VIL	2	827	Capping, Severing, Nucleation, Bundling	Actin nucleation, actin filament bundling, actin filament capping and severing. Gelsolin superfamily protein.	Biliary atresia.	Core fragment in N terminal; headpiece domain in C terminal	https://www.uniprot.org/uniprot/P09327	https://www.proteinatlas.org/ENSG00000127831-VIL1	1979	[198, 199]

Alpha-actinin-1, Alpha-actinin cytoskeletal isoform, F-actin cross-linking protein, Non- muscle alpha-actinin-1	ACTN1	14	892	Bundling	Anchor actin to a variety of intracellular structures.	Bleeding disorder, platelet-type 15 (BDPLT15), congenital macrothrombocytopenia.	CH1, CH2	https://www.uniprot.org/uniprot/P12814	https://www.proteinatlas.org/ENSG00000072110-ACTN1	1978	[200-204]
Alpha-actinin-2, Alpha-actinin skeletal muscle isoform 2	ACTN2	1	894	Bundling	Anchor actin to a variety of intracellular structures.	Cardiomyopathy, familial hypertrophic 23, with or without left ventricular non-compaction (CMH23); Cardiomyopathy, dilated 1AA, with or without left ventricular non-compaction (CMD1AA); Myopathy, congenital, with structured cores and Z-line abnormalities (MYOCOZ); Myopathy, distal, 6, adult onset, autosomal dominant (MPD6).	CH1, CH2	https://www.uniprot.org/uniprot/P35609	https://www.proteinatlas.org/ENSG00000077522-ACTN2	1964	[204-206]
Alpha-actinin-3, Alpha-actinin skeletal muscle isoform 3	ACTN3	11	901	Bundling	Skeletal muscle growth.	Sarcopenia, bone loss.	CH1, CH2	https://www.uniprot.org/uniprot/Q08043	https://www.proteinatlas.org/ENSG00000248746-ACTN3	1992	[206, 207]
Alpha-actinin-4, Non- muscle alpha-actinin 4	ACTN4	19	911	Bundling	Regulate cell motility and invasion.	Focal segmental glomerulosclerosis 1 (FSGS1).	CH1, CH2	https://www.uniprot.org/uniprot/O43707	https://www.proteinatlas.org/ENSG00000130402-ACTN4	1998	[208-210]
Cofilin-1	CFL1, CFL	11	166	Severing	pH-sensitive F-actin depolymerizing activity.	Alzheimer disease; Parkinson's disease; ischemic kidney disease; colorectal cancer; Dent's disease; urothelial cancer; autoinflammatory disease and macrothrombocytopenia; neurodegenerative disease.	ADF-H	https://www.uniprot.org/uniprot/P23528	https://www.proteinatlas.org/ENSG00000172757-CFL1	1984	[211]
Cofilin-2	CFL2, NEM7	14	166	Severing	Controls reversibly actin polymerization and depolymerization in a Ph-sensitive manner. Its F-actin depolymerization activity is regulated by association with CSPR3.	Human muscle disorder; Duchenne muscular dystrophy; nemaline myopathy 7.	ADF-H	https://www.uniprot.org/uniprot/Q9Y281	https://www.proteinatlas.org/ENSG00000165410-CFL2	2001	[212]
Dematin, Dematin actin-binding protein, Erythrocyte membrane Protein band 4.9	DMTN, DMT, EPB49	8	405	Bundling, Stabilization, Anchoring	Induces F-actin bundles formation and stabilization; attaches the spectrin-actin network to the erythrocytic plasma membrane.	Autosomal dominant Marie Unna hereditary hypotrichosis disease; prostate cancer.	N-terminal core domain and the C-terminal headpiece domain	https://www.uniprot.org/uniprot/Q08495	https://www.proteinatlas.org/ENSG00000158856-DMTN	1985	[213]
Filamin-A, Actin-binding protein 280, Endothelial actin-binding protein, Filamin-1, Non- muscle filamin	FLNA, FLN, FLN1	X	2647	Cross-linking, Scaffolding	Scaffolding	Periventricular nodular heterotopia 1 (PVNH1), Otopalatodigital syndrome 2 (OPD2), Frontometaphyseal dysplasia 1 (FMD1), Frontometaphyseal dysplasia 1 (FMD1), Intestinal pseudoobstruction, neuronal, chronic idiopathic, X-linked (IPOX), FG syndrome 2 (FGS2), Terminal osseous dysplasia (TOD), Cardiac valvular dysplasia, X-linked (CVD1), Cardiac valvular dysplasia, X-linked (CVD1)	N-terminal ABD (CH1, CH2), R10	https://www.uniprot.org/uniprot/P21333	https://www.proteinatlas.org/ENSG00000196924-FLNA	1975	[214-218]
Filamin-B, ABP-278, ABP-280 homolog, Actin-binding-like protein, Beta-filamin, Filamin homolog 1, Thyroid autoantigen, Truncated	FLNB, FH1, FLN1L, TABP, TAP	3	2602	Cross-linking, Scaffolding	Scaffolding	Spondylocarpotarsal synostosis (SCT), Larsen syndrome (LS), atelosteogenesis (AO), boomerang dysplasia (BD), and isolated congenital talipes equinovarus.	N-terminal ABD (CH1, CH2)	https://www.uniprot.org/uniprot/O75369	https://www.proteinatlas.org/ENSG00000136068-FLNB	1998	[219-221]

actin-binding protein											
Filamin-C, ABP-280-like protein, Actin-binding-like protein, Filamin-2, Gamma-filamin	FLNC, ABPL, FLN2	7	2725	Cross-linking, Scaffolding	Cross-links F-actin in the Z-disc.	Myopathies, Distal and Myofibrillar Skeletal Myopathy, Cardiomyopathy, Limb-girdle muscular dystrophy.	N-terminal ABD (CH1, CH2)	https://www.uniprot.org/uniprot/Q14315	https://www.proteinatlas.org/ENSG00000128591-FLNC	1998	[222-224]
Macrophage-capping protein, Actin regulatory protein CAP-G	CAPG, AFCP, MCP	2	348	Capping	Block the barbed ends of actin filament.	Breast cancer, bladder cancer, Atherosclerosis, Malignant mesothelioma, ovarian carcinoma, clear cell renal cell carcinoma (ccRCC), lung adenocarcinoma, oral squamous-cell carcinoma, carotid atherosclerosis, prostate cancer, Rheumatoidarthritis, osteoarthritis.	Gelsolin-like 1, Gelsolin-like 2, Gelsolin-like 3	https://www.uniprot.org/uniprot/P40121	https://www.proteinatlas.org/ENSG00000042493-CAPG	1990	[225]
DNA-binding protein SATB1, Special AT-rich sequence-binding protein 1	SATB1	3	763	Scaffolding	Interact with nuclear F-actin.	Liver fibrosis		https://www.uniprot.org/uniprot/Q01826	https://www.proteinatlas.org/ENSG00000182568-SATB1	2014	[226]
Calponin-1, Basic calponin, Calponin H1, smooth muscle	CNN1	19	297	Cross-linking, polymerization	Inhibit the actomyosin Mg-ATPase activity. Specific to smooth muscle cells.	Hepatocellular carcinoma, renal angiomyolipoma, papillary carcinomas, basal cell-like breast carcinoma, metastatic basal cell carcinomas, and prostate cancer, fibrosarcoma, leiomyosarcoma, synovial sarcoma and osteosarcoma.	CH, two actin-binding sites in the middle region.	https://www.uniprot.org/uniprot/P51911	https://www.proteinatlas.org/ENSG00000130176-CNN1	1986	[227-229]
Calponin-2, Calponin H2, smooth muscle, Neutral calponin	CNN2	19	309	Cross-linking, polymerization	Regulate multiple actin cytoskeleton-based functions.	Prostate and breast cancer.	CH, two actin-binding sites in the middle region.	https://www.uniprot.org/uniprot/Q99439	https://www.proteinatlas.org/ENSG00000064666-CNN2	1993	[229-231]
Calponin-3, Calponin, acidic isoform	CNN3	1	329	Cross-linking, polymerization	Participates in actin cytoskeleton-based activities in embryonic development and myogenesis.	Seizure	CH, two actin-binding sites in the middle region.	https://www.uniprot.org/uniprot/Q15417	https://www.proteinatlas.org/ENSG00000117519-CNN3	1994	[229, 232]
Transgelin, 22 kDa actin-binding protein, Protein WS3-10, Smooth muscle protein 22-alpha	TAGLN, SM22, WS3-10	11	201	Bundling	Gelation and stabilization. Organization of the actin cytoskeleton, cell migration and response to stress.	Breast, prostate and colon cancers.	Actin-binding motif (ABM)	https://www.uniprot.org/uniprot/Q01995	https://www.proteinatlas.org/ENSG00000149591-TAGLN	1987	[233-235]
Transgelin-2, Epididymis tissue protein Li 7e, SM22-alpha homolog	TAGLN2, KIAA0120	1	199	Polymerization, Bundling	Blocks Arp2/3-nucleated actin branching.	Gliomas, infertility, lupus erythematosus, bladder, colorectal, hepatocellular and lung cancers, maxillary sinus squamous cell carcinoma, uterine cervical squamous cell carcinoma, Asthma.	Actin-binding motif (ABM)	https://www.uniprot.org/uniprot/P37802	https://www.proteinatlas.org/ENSG00000158710-TAGLN2	1994	[236-239]
Transgelin 3, Neuronal protein 22 (NP22)	TAGLN3, NP25	3	199	Bundling	Regulate actin stabilization and actomyosin contractility.		Actin-binding motif (ABM)	https://www.uniprot.org/uniprot/Q9UI15	https://www.proteinatlas.org/ENSG00000144834-TAGLN3	1994	[240, 241]
Nesprin-1, Enaptin, KASH domain-containing protein 1 (KASH1, Myne-1), Synaptic nuclear envelope protein 1, Synaptic nuclear envelope protein 1 (Syne-1)	SYNE1, C6orf98, KIAA0796, KIAA1262, KIAA1756, MYNE1	6	8797	Anchoring	Anchor the F-actin cytoskeleton to the nuclear envelope.	Autosomal recessive cerebellar ataxia (ARCA), schizophrenia, Disrupted-In-Schizophrenia 1 (DISC1), autism spectrum disorder (ASD), Emery Dreifuss muscular dystrophy (EDMD), Cerebellar ataxia accompanying motor neuron disease (juvenile-onset amyotrophic lateral sclerosis mimic), cardiac disease, arthrogryposis multiplex congenita (AMC), breast, lung, ovarian, pancreatic, head	ABD (1-289)	https://www.uniprot.org/uniprot/Q8NF91	https://www.proteinatlas.org/ENSG00000131018-SYNE1	2001	[242, 243]

						and neck and colorectal cancers.					
Nesprin-2, KASH domain-containing protein 2 (KASH2), Nucleus and actin connecting element protein (NUANCE, Synaptic nuclear envelope protein 2 (Syne-2)	SYNE2, KIAA1011, NUA	14	6885	Anchoring	Anchor the F-actin cytoskeleton to the nuclear envelope.	Emery Dreifuss muscular dystrophy (EDMD), breast, lung, ovarian, pancreatic, head and neck and colorectal cancers.	ABD (1-286)	https://www.uniprot.org/uniprot/Q8WXH0	https://www.proteinatlas.org/ENSG00000054654-SYNE2	2001	[242, 243]
Brain-specific angiogenesis inhibitor 1-associated protein 2-like protein 1, Insulin receptor tyrosine kinase substrate	BAIAP2L1, IRTKS	7	511	Bundling	Induce actin microspikes.	Ovarian cancer, lung adenocarcinoma, bladder cancer, clear cell renal cell carcinoma, pancreatic cancer, lung cancer, prostate cancer, spitzoid melanoma, gastric cancer, rheumatoid arthritis, hepatocellular carcinoma.	IRSp53/MIM homology domain (IMD, 1-249	https://www.uniprot.org/uniprot/Q9UHR4	https://www.proteinatlas.org/ENSG00000006453-BAIAP2L1	2007	[244]
Fascin, 55 kDa actin-bundling protein, Singed-like protein, p55	FSCN1, FAN1, HSN, SNL	7	493	Bundling	Organizes F-actin into parallel bundles. Promote metastasis.	Colorectal cancer, ovarian cancer, renal cell carcinoma, prostate cancer, adrenocortical carcinoma, thyroid carcinoma, squamous cell carcinoma, cervical carcinoma, pituitary adenoma, breast cancer, non-small cell lung cancer, esophageal cancer, hepatocellular carcinoma, gastric cancer, bladder cancer, pancreatic cancer, nasopharyngeal carcinoma, adrenocortical cancer, metastatic melanoma, chondrosarcoma.	Actin-binding sites (ABS) 1-3.	https://www.uniprot.org/uniprot/Q16658	https://www.proteinatlas.org/ENSG00000075618-FSCN1	1975	[245, 246]
Fascin-2, Retinal fascin	FSCN2,	17	492	Bundling	Play a pivotal role in photoreceptor cell-specific events, such as disk morphogenesis.	Retinopathy, Hearing Loss, autosomal dominant retinitis pigmentosa.	ABS	https://www.uniprot.org/uniprot/O14926	https://www.proteinatlas.org/ENSG00000186765-FSCN2	2000	[247]
Fascin-3, Testis fascin	FSCN3	7	498	Bundling	Testis-specific.	Nonobstructive azoospermia.		https://www.uniprot.org/uniprot/Q9NQT6	https://www.proteinatlas.org/ENSG00000106328-FSCN3	2002	[248]
LIM domain and actin-binding protein 1, Epithelial protein lost in neoplasm	LIMA1, EPLIN, SREBP3	12	759	Bundling, Stabilization	Formation of stress fibers. Inhibit membrane ruffling.	Oral cancer, breast cancer, prostate cancer, squamous cell carcinoma of head and neck (SCCHN), lung cancer, oesophageal cancer, ovarian cancer, colorectal cancer (CRC), gastric cancer.	Actin binding region	https://www.uniprot.org/uniprot/Q9UHB6	https://www.proteinatlas.org/ENSG00000050405-LIMA1	1999	[249]
Protein flightless-1 homolog	FLII, FLIL	17	1269	Monomer binding, Capping, Severing	Regulate actin dynamics. Gelsolin superfamily protein.	OVA-Induced Atopic Dermatitis Skin-Like Disease, epidermolysis bullosa acquisita (EBA), prostate cancer, epithelial ovarian cancer, breast cancer, endometrial cancer, alcoholic liver disease, Ulcerative colitis, psoriasiform dermatitis, cutaneous leishmaniasis, Systemic lupus erythematosus (SLE), melanoma.	gelsolin-like domain	https://www.uniprot.org/uniprot/Q13045	https://www.proteinatlas.org/ENSG00000177731-FLII	1993	[250]
Four and a half LIM domains protein 3 (FHL-3), Skeletal muscle LIM-protein 2 (SLIM-2)	FHL3, SLIM2	1	280	?	Inhibit alpha-actinin-mediated actin bundling. Enhance cell spreading and stress fiber disassembly.	Pancreatic, gastric breast cancers, glioma.	LIM domains	https://www.uniprot.org/uniprot/Q13643	https://www.proteinatlas.org/ENSG00000183386-FHL3	2003	[251-253]

WD repeat-containing protein 1, Actin-interacting protein 1 (AIP1, NORI-1)	WDR1	4	606	Depolymerization	Enhances the filament disassembly activity of cofilin and restricts cofilin localization to cortical actin patches. Regulate cytokinesis and migration.	Gout, pancreatitis, and primary glioblastoma.		https://www.uniprot.org/uniprot/O75083	https://www.proteinatlas.org/ENSG00000071127-WDR1	1999	[254-256]
Plastin-1, Intestine-specific plastin (I-plastin), fimbrin	PLS1	3	629	Bundling	Required for stereocilia formation.	Deafness, autosomal dominant, 76 (DFNA76).	CH (108-380, 381-625)	https://www.uniprot.org/uniprot/Q14651	https://www.proteinatlas.org/ENSG00000120756-PLS1	1980	[257, 258]
Plastin-2, Lymphocyte cytosolic protein 1 (LCP-1), L-plastin, LC64P	LCP1, PLS2	13	627	Bundling	Regulate T-cell activation.	B-cell non-Hodgkin lymphomas, coloboma, melanoma, breast, prostate, colorectal cancer.	CH (106-379, 380-624)	https://www.uniprot.org/uniprot/P13796	https://www.proteinatlas.org/ENSG00000136167-LCP1	1990	[259-261]
Plastin-3, T-plastin	PLS3	X	630	Bundling	Regulate actin-based cellular processes. Regulate bone development.	Acute myeloid leukemia (AML), Sézary syndrome (SS), Osteoporosis, osteoarthritis, colorectal, prostate, breast, gastric, and lung cancer, thoracic aortic dissection, Spinal muscular atrophy (SMA), ataxia, amyotrophic lateral sclerosis (ALS), and Charcot–Marie–Tooth (CMT), Infection and pathogen entry.	CH (1069-382, 383-627)	https://www.uniprot.org/uniprot/P13797	https://www.proteinatlas.org/ENSG00000102024-PLS3	1988	[262, 263]
KICSTOR complex protein kaptin, Actin-associated protein 2E4	KPTN, 2E4	19	436	?	Localized in lamellipodia.	Deafness, KPTN-related syndrome.	Bind to F-actin column	https://www.uniprot.org/uniprot/Q9Y664	https://www.proteinatlas.org/ENSG00000118162-KPTN	1999	[264]
TRIO and F-actin-binding protein, Protein Tara, Trio-associated repeat on actin	TRIOBP, KIAA1662, TARA	22	2365	Stabilization	Regulate cell spreading and contraction.	Deafness, autosomal recessive 28 (DFNB28), Schizophrenia, gastric, rectal, pancreatic and brain cancer.	R1 motif	https://www.uniprot.org/uniprot/Q9H2D6	https://www.proteinatlas.org/ENSG00000100106-TRIOBP	2001	[265]
Tyrosine-protein kinase ABL1, Abelson murine leukemia viral oncogene homolog 1, Abelson tyrosine-protein kinase 1, Proto-oncogene c-Abl, p150	ABL1, ABL, JTK7	9	1130	Bundling	Membrane ruffling, cell spreading, cell migration, and neurite extension in response to growth factor and extracellular matrix signals.	Alzheimer's Disease, Parkinson's disease (PD), Lewy body dementia, neuro-inflammation,breast, prostate and renal cancer, leukemia.	C-terminal	https://www.uniprot.org/uniprot/P00519	https://www.proteinatlas.org/ENSG00000097007-ABL1	2001	[266-268]
Myristoylated alanine-rich C-kinase substrate (MARCKS), Protein kinase C substrate, 80 kDa protein, light chain (80K-L protein, PKCSL)	MARCKS, MACS, PRKCSL	6	332	Cross-linking	Major substrate of PKC. Regulate actin dynamics.	Chronic obstructive pulmonary disease, asthma, lung cancer, and acute lung injury/acute respiratory distress syndrome.	Phosphorylation site domain (PSD), effector domain (ED)	https://www.uniprot.org/uniprot/P29966	https://www.proteinatlas.org/ENSG00000277443-MARCKS	1991	[269-274]
MARCKS-related protein, MARCKS-like protein 1, Macrophage myristoylated alanine-rich C kinase substrate (Mac-MARCKS)	MARCKSL1, MLP, MRP	1	195	Bundling, Stabilization	Regulate actin dynamics.. Regulate spine formation in the central amygdala.	Anxiety in mice.	Effector domain (ED)	https://www.uniprot.org/uniprot/P49006	https://www.proteinatlas.org/ENSG00000175130-MARCKSL1	2000	[272, 274, 275]
Neurabin-1, Protein phosphatase 1 regulatory subunit 9A, Neural tissue-specific F-actin-binding protein I	PPP1R9A, KIAA1222	7	1098	Cross-linking	Regulate cell morphology. Inhibit protein phosphatase 1-alpha activity.	Prostate cancer, schizophrenia and bipolar disorder, Hepatosplenic T-cell lymphoma (HSTL), Huntington disease, squamous cell carcinoma of head and neck (SCCHN), papillary thyroid cancer, breast cancer, restless legs syndrome (RLS).	ABD (1-144)	https://www.uniprot.org/uniprot/Q9ULJ8	https://www.proteinatlas.org/ENSG00000158528-PPP1R9A	1997	[276, 277]
Neurabin-2, Neurabin-II, Protein	PPP1R9B, PPP1R6	17	817	Cross-linking	Scaffolding	Alzheimer's disease, hypertension,	ABD (1-154, 164-283)	https://www.uniprot.org/uniprot/Q96SB3	https://www.proteinatlas.org/ENSG0	1998	[278-280]

phosphatase 1 regulatory subunit 9B, Spinophilin						hepatocellular carcinoma, Parkinson disease, breast, colorectal, lung, head and neck, colon, prostate cancers.			0000108819-PPP1R9B		
Protein spire homolog 1 (Spir-1)	SPIRE1, KIAA1135, SPIR1	18	756	Nucleation	Mediate asymmetric spindle positioning by assembling an actin network. Caps the pointed end.		WH2	https://www.uniprot.org/uniprot/Q08AE8	https://www.proteinatlas.org/ENSG00000134278-SPIRE1	1999	[281, 282]
Protein spire homolog 2 (Spir-2)	SPIRE2, KIAA1832, SPIR2	16	714	Nucleation	Mediate asymmetric spindle positioning by assembling an actin network.		WH2	https://www.uniprot.org/uniprot/Q8WWL2	https://www.proteinatlas.org/ENSG00000204991-SPIRE2	2004	[283, 284]
Drebrin, Developmentally-regulated brain protein	DBN1, D0S117E	5	649	Stabilization	The stability, dynamics, and organizations of actin structures in neuronal cells.	Alzheimer's disease, hereditary and acquired glomerulopathies, Down Syndrome, Seizures and Suspected Encephalitis, prostate cancer, lung adenocarcinoma,.glaucoma.	ADF-H	https://www.uniprot.org/uniprot/Q16643	https://www.proteinatlas.org/ENSG00000113758-DBN1	1993	[285-290]
Tropomyosin alpha-1 chain, Alpha-tropomyosin, Tropomyosin-1	TPM1, C15orf13, TMSA	15	284	Stabilization	Regulate contractile systems and cytoskeleton, modulates actin-myosin interaction.	Hypertrophic cardiomyopathy (HCM), Dilated cardiomyopathy (DCM), left ventricular noncompaction (LVNC), arrthythmogenic right ventricular cardiomyopathy.	ABS	https://www.uniprot.org/uniprot/P09493	https://www.proteinatlas.org/ENSG00000140416-TPM1	1946	[291-296]
Tropomyosin beta chain, Beta-tropomyosin, Tropomyosin-2	TPM2, TMSB	9	284	Stabilization	Regulates contractile systems and cytoskeleton, modulates actin-myosin interaction.	Myopathies and distal arthrogryposis.	ABS	https://www.uniprot.org/uniprot/P07951	https://www.proteinatlas.org/ENSG00000198467-TPM2	1988	[293-297]
Tropomyosin alpha-3 chain, Gamma-tropomyosin, Tropomyosin-3, Tropomyosin-5 (Htm5)	TPM3	1	285	Stabilization	Regulates contractile systems and cytoskeleton, modulates actin-myosin interaction.	Myopathies and distal arthrogryposis.	ABS	https://www.uniprot.org/uniprot/P06753	https://www.proteinatlas.org/ENSG00000143549-TPM3	1988	[293-298]
Tropomyosin alpha-4 chain, TM30p1, Tropomyosin-4	TPM4	19	248	Stabilization	Regulates contractile systems and cytoskeleton, modulates actin-myosin interaction.	Macrothrombocytopenia, Muscle diseases.	ABS	https://www.uniprot.org/uniprot/P67936	https://www.proteinatlas.org/ENSG00000167460-TPM4	1988	[293-297, 299]
Xin actin-binding repeat-containing protein 1, Cardiomyopathy-associated protein 1	XIRP1, CMYA1, XIN	3	1843	Stablization	Required for cardiac development and cardiac function. Protects actin filaments from depolymerization.	Cardiomyopathy, arrhythmias, heart disease, intercalated disc (ICD).	Xin-repeats	https://www.uniprot.org/uniprot/Q702N8	https://www.proteinatlas.org/ENSG00000168334-XIRP1	2004	[300, 301]
Xin actin-binding repeat-containing 2, Beta-xin, Cardiomyopathy-associated protein 3, Xeplin	XIRP2, CMYA3	2	3374	Stablization	Required for long-term maintenance of hair cell stereocilia.	Hearing loss	Xin-repeats	https://www.uniprot.org/uniprot/A4UGR9	https://www.proteinatlas.org/ENSG00000163092-XIRP2	2004	[300, 302]
Microtubule-actin cross-linking factor 1, isoforms 1/2/3/5, 620 kDa actin-binding protein (ABP620), Actin cross-linking family protein 7, Macrophin-1, Trabeculin-alpha	MACF1, ABP620, ACF7, KIAA0465, KIAA1251	1	7388	Cross-linking	Modulate actin and microtubule cytoskeletal networks.	Spectraplakinopathy type I, schizophrenia, Parkinson's disease (PD), breast, lung, colorectal, liver cancer, gliomas and glioblastoma, renal cell carcinoma, steoporosis. Lissencephaly 9 with complex brainstem malformation (LIS9)	CH1, CH2	https://www.uniprot.org/uniprot/Q9UPN3	https://www.proteinatlas.org/ENSG00000127603-MACF1	1995	[303-306]
Allograft inflammatory factor 1 (AIF-1), Ionized calcium-binding adapter molecule 1, Protein G1	AIF1, G1, IBA1	6	147	Polymerization, Bundling	Enhance the actin-bundling activity of LCP1. Promote membrane ruffling and the phagocytosis of macrophages. Involved in Rac and calcium signaling pathways.	Experimental autoimmune neuritis.		https://www.uniprot.org/uniprot/P55008	https://www.proteinatlas.org/ENSG00000204472-AIF1	2001	[307-309]
Allograft inflammatory factor 1-like, Ionized calcium-binding adapter molecule 2	AIF1L, C9orf58, IBA2	9	150	Bundling	Regulates actomyosin contractility and filopodial extensions.	Breast cancer.		https://www.uniprot.org/uniprot/Q9BQI0	https://www.proteinatlas.org/ENSG00000126878-AIF1L	2008	[310, 311]

EF-hand domain-containing protein D1, EF-hand domain-containing protein 1, Swiprosin-2	EFHD1, SWS2	2	239	Bundling	Bind to β-actin in the mitochondrial matrix.	Colorectal cancer		https://www.uniprot.org/uniprot/Q9BUP0#sequences	https://www.proteinatlas.org/ENSG00000115468-EFHD1	2013	[312, 313]
EF-hand domain-containing protein D2 (EFhd2), Swiprosin-1	EFHD2, SWS1	1	240	Bundling	Regulate cell spreading and migration.	Lung adenocarcinoma, acute myeloid leukemia (AML), idiopathic cardiomyopathy, Schizophrenia, Parkinson's disease (PD), Alzheimer's disease (AD), Huntington's disease (HD), amyotrophic lateral sclerosis (ALS).		https://www.uniprot.org/uniprot/Q96C19#sequences	https://www.proteinatlas.org/ENSG00000142634-EFHD2	2013	[312, 314]
CD2-associated protein, Adapter protein CMS, Cas ligand with multiple SH3 domains	CD2AP	6	639	Capping, Anchoring	Actin barbed-end capping protein. May anchor the podocyte slit diaphragm to the actin cytoskeleton in renal glomerolus.	Focal segmental glomerulosclerosis 3 (FSGS3), Congenital nephrotic syndromes, Alzheimer's Disease.	C-terminal	https://www.uniprot.org/uniprot/Q9Y5K6	https://www.proteinatlas.org/ENSG00000198087-CD2AP	2002	[315-317]
Coactosin-like protein	COTL1, CLP	16	142	Polymerization	Regulate lamellipodia dynamics in part by protecting F-actin from cofilin-mediated disassembly.	Rheumatoid arthritis	ADF-H	https://www.uniprot.org/uniprot/Q14019	https://www.proteinatlas.org/ENSG00000103187-COTL1	2001	[318, 319]
Epidermal growth factor receptor kinase substrate 8	EPS8	12	822	Capping, Bundling	Cap barbed end. Regulate EGFR transduction, actin dynamics, cell cycle regulation and cell proliferation.	Head and neck squamous cell carcinomas (HNSCCs), deafness, papillary thyroid carcinoma (PTC), breast, colon, cervical, ovarian cancers, pituitary tumor, pancreatic ductal adenocarcinoma (PDAC), oral squamous cell carcinoma (OSCC), esophageal squamous cell carcinoma (ESCC), acute lymphocytic leukemia (ALL), chronic myeloid leukemia (CML), acute myeloid leukemia (AML), multiple myeloma (MM), non-small cell lung cancer (NSCLC).	C-terminal effector domain	https://www.uniprot.org/uniprot/Q12929	https://www.proteinatlas.org/ENSG00000151491-EPS8	2004	[320-322]
Epidermal growth factor receptor kinase substrate 8-like protein 1 (EPS8-like protein 1), Epidermal growth factor receptor pathway substrate 8-related protein 1 (EPS8-related protein 1)	EPS8L1, DRC3, EPS8R1	19	723	Scaffolding	Stimulate guanine exchange activity of SOS1 and remodel the actin cytoskeleton.		C-terminal effector domain	https://www.uniprot.org/uniprot/Q8TE68	https://www.proteinatlas.org/ENSG00000131037-EPS8L1	2004	[323]
Epidermal growth factor receptor kinase substrate 8-like protein 2 (EPS8-like protein 2), Epidermal growth factor receptor pathway substrate 8-related protein 2 (EPS8-related protein 2)	EPS8L2, EPS8R2	11	715	Scaffolding	Stimulate guanine exchange activity of SOS1 and remodel the actin cytoskeleton. Required for stereocilia maintenance in adult hair cells.	Childhood onset autosomal recessive progressive hearing loss。	C-terminal effector domain	https://www.uniprot.org/uniprot/Q9H6S3	https://www.proteinatlas.org/ENSG00000177106-EPS8L2	2004	[323, 324]
Signal transducing adapter molecule 2, EGFR-Associated protein with SH3 and TAM	STAM2, EAST		468	Anchoring	Involved in the EGFR-regulated reorganization of the actin cytoskeleton and may be part of a link between cytoskeleton and		Shown with Chicken protein, N-terminal	Chicken: https://www.uniprot.org/uniprot/O93436 Human: https://www.uniprot.org/uniprot/O75886		1998	[325]

domains, EAST					endocytic machinery.						
Phosphatase and actin regulator 1	PHACTR1, KIAA1733, RPEL1	6	580	Monomer binding	Involved in stress fiber assembly, motility and invasiveness	Parkinson's disease, prostate cancer and myocardial infarction. Developmental and epileptic encephalopathy 70 (DEE70).	RPEL-repeat domain	https://www.uniprot.org/uniprot/Q9C0D0	https://www.proteinatlas.org/ENSG00000112137-PHACTR1	2004	[326, 327]
Phosphatase and actin regulator 2	PHACTR2, C6orf56, KIAA0680	6	634	Monomer binding	Involved in stress fiber assembly, motility and invasiveness	Parkinson's disease, tongue squamous cell carcinoma, breast cancer, lung adenocarcinoma, esophageal squamous cell carcinoma,	RPEL-repeat domain	https://www.uniprot.org/uniprot/O75167	https://www.proteinatlas.org/ENSG00000112419-PHACTR2	2004	[328]
Phosphatase and actin regulator 3, Scaffold-associated PP1-inhibiting protein (Scapinin)	PHACTR3, C20orf101, SCAPIN1	20	559	Monomer binding	Involved in stress fiber assembly, motility and invasiveness	Lung carcinoma, colorectal cancer, non-small-cell lung cancer.	RPEL-repeat domain	https://www.uniprot.org/uniprot/Q96KR7	https://www.proteinatlas.org/ENSG00000087495-PHACTR3	2004	[254-256]
Phosphatase and actin regulator 4	PHACTR4	1	702	Monomer binding	Involved in stress fiber assembly, motility and invasiveness	Hepatocellular carcinoma.	RPEL-repeat domain	https://www.uniprot.org/uniprot/Q8IZ21	https://www.proteinatlas.org/ENSG00000204138-PHACTR4	2004	[257, 258]
Brain-specific angiogenesis inhibitor 1-associated protein 2 (BAI-associated protein 2, BAI1-associated protein 2, Protein BAP2 Alternative), as ligand-associated factor 3 (FLAF3), Insulin receptor substrate p53/p58 (IRS-58, IRSp53/58), Insulin receptor substrate protein of 53 kDa (RSp53)	BAIAP2	17	552	Bundling	Induce filopodia and the formation of tightly packed parallel F-actin bundles.	Schizophrenia, autism spectrum disorders (ASDs) and attention deficit/hyperactivity disorder (ADHD).	IRSp53/MIM homology domain (IMD)	https://www.uniprot.org/uniprot/Q9UQB8	https://www.proteinatlas.org/ENSG00000175866-BAIAP2	1999	[259-261]
Huntingtin-interacting protein 1 (HIP-1)	HIP1	7	1037	Anchoring, Scaffolding	Involved in both endocytosis and regulate actin cytoskeleton.	Huntington's disease, chronic myelomonocytic leukemia.	Talin-HIP1/R/Sla2p actin-tethering C-terminal homology (THATCH) domain	https://www.uniprot.org/uniprot/O00291	https://www.proteinatlas.org/ENSG00000127946-HIP1	1999	[262, 263]
Huntingtin-interacting protein 1-related protein (HIP1-related protein), Huntingtin-interacting protein 12 (HIP-12)	HIP1R, HIP12, KIAA0655	12	1068	Anchoring, Scaffolding	Link membrane attachment and clathrin-coated vesicle formation with actin dynamics.	Huntington's disease	Talin-HIP1/R/Sla2p actin-tethering C-terminal homology (THATCH) domain	https://www.uniprot.org/uniprot/O75146	https://www.proteinatlas.org/ENSG00000130787-HIP1R	1999	[264]
Actin filament-associated protein 1, 110 kDa actin filament-associated protein (AFAP-110)	AFAP1, AFAP	4	730	Cross-linking, Scaffolding	Act as an adaptor protein that links signaling molecules to actin filaments. Serve as a platform for the construction of larger signaling complexes. Serve as an activator of Src family kinases in response to cellular signals that alter its conformation. Effect actin organization,	Glaucoma	C-terminal ABD	https://www.uniprot.org/uniprot/Q8N556	https://www.proteinatlas.org/ENSG00000196526-AFAP1	1993	[265]
Proline-serine-threonine phosphatase interacting protein 2 (PEST phosphatase-interacting protein 2), MAYP	PSTPIP2	18	344	Bundling	Participate in macrophage activation, neutrophil migration, cytokine production, and osteoclast differentiation.	Innate immune diseases and autoinflammatory diseases (AIDs), chronic recurrent multifocal osteomyelitis (CRMO)	F-BAR domain	https://www.uniprot.org/uniprot/Q9H939	https://www.proteinatlas.org/ENSG00000152229-PSTPIP2	1998	[266-268]

Protein Shroom1, Apical protein 2	SHROOM1, APXL2, KIAA1960	5	852	Anchoring, Scaffolding	Regulate gamma-tubulin distribution and microtubule architecture. Regulate myosin activity.		754–953 of mouse Shrm	https://www.uniprot.org/uniprot/Q2M3G4	https://www.proteinatlas.org/ENSG00000164403-SHROOM1	1999	[269-274]
Protein Shroom2, Apical-like protein, Protein APXL	SHROOM2, APXL	X	1616	Stabilization, Scaffolding	Provide a link between the plasma membrane and the cortical cytoskeleton at the tight junction. Regulate melanosome biogenesis and localization in the retinal pigment epithelium.	Nasopharyngeal carcinoma, colorectal cancer, Neural tube defects (NTDs).	ASD1 domain (513-880)	https://www.uniprot.org/uniprot/Q13796	https://www.proteinatlas.org/ENSG00000146950-SHROOM2	2006	[272, 274, 275]
Protein Shroom3, Shroom-related protein (hShrmL)	SHROOM3, KIAA1481, SHRML	4	1996	Scaffolding	Regulate cell shape changes in the neuroepithelium during neural tube closure.	Anencephaly, death at birth, infant with spina bifida.	ASD1 domain (928-1030)	https://www.uniprot.org/uniprot/Q8TF72	https://www.proteinatlas.org/ENSG00000138771-SHROOM3	2006	[276, 277]
Protein Shroom4, Second homolog of apical protein	SHROOM4, KIAA1202, SHAP	X	1493	Scaffolding	Induces the robust formation of the actin-based structures.	X-linked mental retardation, Dent disease.		https://www.uniprot.org/uniprot/Q9ULL8	https://www.proteinatlas.org/ENSG00000158352-SHROOM4	2007	[278-280]
Alpha-adducin, Erythrocyte adducin subunit alpha	ADD1, ADDA	4	737	Capping, Bundling	Recruite spectrin to actin filaments, bundling actin filaments and capping the barbed ends of actin filaments. Stabilize membrane cytoskeleton and cell-cell junctions.	Amyotrophic Lateral Sclerosis (ALS), ovarian cancer, non-cardia gastric cancer, colorectal cancer, lung cancer, small cell lung cancer, coronary heart disease, renal failure.	MARCKS-related domain	https://www.uniprot.org/uniprot/P35611	https://www.proteinatlas.org/ENSG00000087274-ADD1	1986	[281, 282]
Beta-adducin, Erythrocyte adducin subunit beta	ADD2, ADDB	2	726	Capping, Bundling	Recruite spectrin to actin filaments, bundling actin filaments and capping the barbed ends of actin filaments. Stabilize membrane cytoskeleton and cell-cell junctions.	Basal cell carcinoma and squamous cell carcinoma.	MARCKS-related domain	https://www.uniprot.org/uniprot/P35612	https://www.proteinatlas.org/ENSG00000075340-ADD2	1986	[283, 284]
Gamma-adducin, Adducin-like protein 70	ADD3, ADDL	10	706	Capping, Bundling	Recruite spectrin to actin filaments, bundling actin filaments and capping the barbed ends of actin filaments. Stabilize membrane cytoskeleton and cell-cell junctions.	Small cell lung cancer, murine breast tumor, non-small cell lung cancer, colorectal cancer, glioblastoma, T-lymphoblastic leukemia.	MARCKS-related domain	https://www.uniprot.org/uniprot/Q9UEY8	https://www.proteinatlas.org/ENSG00000148700-ADD3	1995	[285-289]
Anillin	ANLN	7	1124	Bundling	Organize the cortical actomyosin cytoskeleton in syncytial structures, involved in cell division.	Lung, Breast, pancreatic, colorectal, liver, bladder, urothelial, renal, nasopharyngeal, ovarian, hormone resistant prostate cancer.	ABD	https://www.uniprot.org/uniprot/Q9NQW6	https://www.proteinatlas.org/ENSG00000011426-ANLN	1995	[110, 111]
Espin, Autosomal recessive deafness type 36 protein, Ectoplasmic specialization protein	ESPN, DFNB36, LP2654	1	854	Bundling	Connect to hair cell stereocilia and microvillar specializations of sensory cells in the inner ear.	Melanoma	Actin-bundling module (ABM)	https://www.uniprot.org/uniprot/B1AK53	https://www.proteinatlas.org/ENSG00000187017-ESPN	1996	[110, 111]
Transcription activator BRG1, ATP-dependent helicase SMARCA4, BRG1-associated factor 190A (BAF190A), Mitotic growth and transcription activator, Protein BRG-1, Protein brahma homolog 1SWI/SNF related, matrix-associated actin-dependent regulator of chromatin subfamily A, member 4, SNF2-beta	SMARCA4, BAF190A, BRG1, SNF2B, SNF2L4	19	1647	Capping	Induce the formation of thick actin filament bundles resembling stress-fibers. Change the morphology and alterations in actin cytoskeletal organization.	Non-small cell lung carcinomas, lung adenocarcinoma, lung large-cell carcinoma, lung squamous cell carcinoma, prostate, breast, pancreas, colon cancers.	C-terminal	https://www.uniprot.org/uniprot/P51532	https://www.proteinatlas.org/ENSG00000127616-SMARCA4	2002	[111, 112]
Palladin, SIH002, Sarcoma antigen NY-SAR-77	PALLD, CGI-151, KIAA0992	4	1383	Bundling, Scaffolding	Regulate cell morphology, motility, cell adhesion.	Myocardial infarction (MI), pancreatic ductal adenocarcinoma (PDA), colorectal	Immunoglobulin-like domain (Ig-like domain 3)	https://www.uniprot.org/uniprot/Q8WX93	https://www.proteinatlas.org/ENSG00000129116-PALLD	2000	[291-296]

						cancer, pancreatic cancer, breast cancer.					
Myotilin, 57 kDa cytoskeletal protein, Myofibrillar titin-like Ig domains protein, Titin immunoglobulin domain protein	MYOT, TTID	5	498	Bundling	Regulate myofibril assembly and stability at the Z lines in muscle cells.	Late onset autosomal dominant distal limb girdle muscular dystrophy, spheroid body myopathy, myofibrillar myopathy.	C-terminal Ig-like domains	https://www.uniprot.org/uniprot/Q9UBF9	https://www.proteinatlas.org/ENSG00000120729-MYOT	2003	[293-297]
Myopalladin, 145 kDa sarcomeric protein	MYPN, MYOP	10	1320	Bundling, Scaffolding	Component of Z-lines.	Dilated cardiomyopathy, Familial restrictive cardiomyopathy (FRCM).	Ig-like domain	https://www.uniprot.org/uniprot/Q86TC9	https://www.proteinatlas.org/ENSG00000138347-MYPN	2001	[293-298]
Protein Abitram, Actin-binding transcription modulator, Protein Simiate	ABITRAM, C9orf6, FAM206A	9	181	Monomer binding, Capping, F-actin binding	Involved in filopodia dynamics and arborization of neurons.	Fragile X syndrome.		https://www.uniprot.org/uniprot/Q9NX38	https://www.proteinatlas.org/ENSG00000119328-ABITRAM	2014	[293-297, 299]
Myosin phosphatase Rho-interacting protein (M-RIP), Rho-interacting protein 3 (RIP3), p116Rip	MPRIP, KIAA0864, MRIP, RHOIP3	17	1025	Bundling	Disrupt stress fibers and promote formation of dendrite-like extensions. Disassemble the actomyosin-based cytoskeleton.	Lung adenocarcinoma, lung cancer, myeloproliferative neoplasms.	N-terminal (1-382)	https://www.uniprot.org/uniprot/Q6WCQ1	https://www.proteinatlas.org/ENSG00000133030-MPRIP	2003	[300, 301]
Alpha-parvin, Actopaxin, Calponin-like integrin-linked kinase-binding protein (CH-ILKBP), Matrix-remodeling-associated protein 2	PARVA, MXRA2	11	372	Scaffolding	Involved in integrin-mediated cell adhesion.	Lobular breast carcinoma, diabetic nephropathy.	CH1, CH2	https://www.uniprot.org/uniprot/Q9NVD7	https://www.proteinatlas.org/ENSG00000197702-PARVA	2000	[300, 302]
Caldesmon (CDM)	CALD1, CAD, CDM	7	793	Stabilization	Capable of stabilizing actin filaments against actin-severing proteins, inhibiting actomyosin ATPase activity, and inhibiting Arp2/3-mediated actin polymerization. Involved in smooth muscle contraction, cell motility and secretion.	Atherosclerosis, restenosis, glioma, Gastrointestinal stromal tumor, Ovarian adult granulosa cell tumor, Epithelioid pleural mesothelioma, Oral cavity squamous cell carcinoma, colorectal cancer, bladder cancer, Melanoma, Leiomyosarcoma, Fibroxanthoma.	653-686, 768-793	https://www.uniprot.org/uniprot/Q05682	https://www.proteinatlas.org/ENSG00000122786-CALD1	1981	[303-306]
Switch-associated protein 70 (SWAP-70)	SWAP70, KIAA0640	11	585	Bundling	Alter the actin organization and lamellipodial morphology.	Prostate cancer	ABD	https://www.uniprot.org/uniprot/Q9UH65	https://www.proteinatlas.org/ENSG00000133789-SWAP70	2003	[307-309]
Afadin, ALL1-fused gene from chromosome 6 protein (Protein AF-6), Afadin adherens junction formation factor	AFDN, AF6, MLLT4	6	1824	Scaffolding	Enhance the formation of adherens and tight junctions. Effect on cell adhesion, polarization, migration, differentiation, and survival.	Breast cancer, Parkinson's Disease.	C-terminal (1631-1829)	https://www.uniprot.org/uniprot/P55196	https://www.proteinatlas.org/ENSG00000130396-AFDN	1997	[329, 330]
Fructose-bisphosphate aldolase A, Lung cancer antigen NY-LU-1, Muscle-type aldolase	ALDOA, ALDA	16	364	Scaffolding	Promote lung cancer metastasis by interacting with gamma-actin. Work with cofilin keeping the polymerization and depolymerization of actin filaments in an orderly dynamic equilibrium.	Renal clear cell, hepatocellular and lung squamous cell carcinomas.		https://www.uniprot.org/uniprot/P04075	https://www.proteinatlas.org/ENSG00000149925-ALDOA	2019	[331-333]
Angiogenin, Ribonuclease 5 (RNase 5)	ANG, RNASE5	14	147	Polymerization	Induce angiogenesis by activating vessel endothelial and smooth muscle cells. Regulate cell migration, invasion, proliferation, and formation of tubular structures.	Amyotrophic lateral sclerosis (ALS), Parkinson's disease (PD), Alzheimer disease (AD), inflammatory bowel disease, peripheral artery disease (PAD), cardiovascular disease, colonic adenocarcinomas, gastric cancers, pancreatic cancers, breast cancer, head and neck squamous carcinoma, acute myeloid leukemia, advanced myelodysplasic		https://www.uniprot.org/uniprot/P03950	https://www.proteinatlas.org/ENSG00000214274-ANG	1993	[334-338]

						syndrome, arthritis, preterm delivery, hemangioendothelioma, chronic kidney disease, ischaemic heart disease, sickle cell disease, thalassemia, non-Hodgkin's lymphoma and Hodgkin's disease, inflammatory bowel disease (IBD), rheumatoid arthritis, overweight and obesity, proliferative diabetic retinopathy, proliferative vitreopathy					
Dystrophin	DMD	X	3685	Stablization	Link the actin cytoskeleton to the dystroglycan complex in the plasma membrane. Bind to the intracellular actin network to link the cytoskeleton to dystrophin glycoprotein complex.	Duchenne and Becker muscular dystrophies (DMD and BMD), X-linked dilated cardiomyopathy,	CH1 (15-119), CH2 (134-240)	https://www.uniprot.org/uniprot/P11532	https://www.proteinatlas.org/ENSG00000198947-DMD	1987	[339-344]
Dystroglycan 1, Dystrophin-associated glycoprotein 1, Dystroglycan	DAG1	3	895	Bundling	Maintain sarcolemmal integrity.	Muscular dystrophy-dystroglycanopathy limb-girdle C9 (MDDGC9), Muscular dystrophy-dystroglycanopathy congenital with brain and eye anomalies A9 (MDDGA9)	The cytoplasmic tail	https://www.uniprot.org/uniprot/Q14118	https://www.proteinatlas.org/ENSG00000173402-DAG1	2003	[345]
Vasodilator-stimulated phosphoprotein	VASP	19	380	Polymerization, Bundling	Stimulate actin polymerization by promoting the transfer of profilin-bound actin monomers onto the barbed end of growing F-actin.	Listeria monocytogenes	Enabled/VASP homology (EVH2) contains a G-actin-binding site (GAB), an F-actin-binding site (FAB).	https://www.uniprot.org/uniprot/P50552	https://www.proteinatlas.org/ENSG00000125753-VASP	1992	[346-348]
Protein enabled homolog	ENAH, MENA	1	591	Polymerization, Bundling	Regulate actin remodeling. Involved in carcinoma cell invasion and metastasis.	Carcinaoma.	EVH2 contains GAB and FAB.	https://www.uniprot.org/uniprot/Q8N8S7	https://www.proteinatlas.org/ENSG00000154380-ENAH	1996	[348, 349]
Ena/VASP-like protein, Ena/vasodilator-stimulated phosphoprotein-like protein	EVL, RNB6	14	416	Polymerization, Bundling	Implicate in the regulation of axon guidance, platelet aggregation, cell motility, and cell adhesion.	Breast cancer	EVH2 contains GAB and FAB.	https://www.uniprot.org/uniprot/Q9UI08	https://www.proteinatlas.org/ENSG00000196405-EVL	1997	[348, 350-352]
Kelch-like protein 1	KLHL1, KIAA1490	13	748	Scaffolding	Bind to F-actin. Modulate neuronal structure and function.	Neurodegenerative diseases.	C-terminal Kelch β-propeller region	https://www.uniprot.org/uniprot/Q9NR64	https://www.proteinatlas.org/ENSG00000150361-KLHL1	2000	[353, 354]
Kelch-like protein 17, Actinfilin	KLHL17, AF	1	642	Scaffolding	Bind to F-actin. Form circular puncta in dendritic spines and are surrounded by or adjacent to F-actin.	Infantile spasms and autism.	C-terminal Kelch domain (289 – 641)	https://www.uniprot.org/uniprot/Q6TDP4	https://www.proteinatlas.org/ENSG00000187961-KLHL17	2002	[355, 356]
Protein 4.1 (P4.1), Erythrocyte membrane protein band 4.1, 4.1R, Band 4.1, EPB4.1	EPB41, E41P	1	864	Anchoring, Cross-linking	Involved in cytoskeletal rearrangements, intracellular transport and signal transduction.	Hereditary elliptocytosis (HE), bradycardia and/or Long QT syndrome,	Spectrin-actin-binding domain (SABD, 615-713)	https://www.uniprot.org/uniprot/P11171	https://www.proteinatlas.org/ENSG00000159023-EPB41	1979	[357-360]
Band 4.1-like protein 2, Erythrocyte membrane protein band 4.1-like 2, Generally expressed protein 4.1 (4.1G)	EPB41L2	6	1005	Anchoring, Cross-linking	Regulates cell adhesion, spreading, and migration.		Spectrin-actin-binding domain (SABD, 611-676)	https://www.uniprot.org/uniprot/O43491	https://www.proteinatlas.org/ENSG00000079819-EPB41L2	1998	[359, 361, 362]
Band 4.1-like protein 1, Erythrocyte membrane protein band 4.1-like 1, Neuronal protein 4.1, 4.1N.	EPB41L1	20	881	Anchoring, Cross-linking	Regulate stability and plasticity of neuronal membrane.	Mental retardation, autosomal dominant 11 (MRD11).	Spectrin-actin-binding domain (SABD, 483-541)	https://www.uniprot.org/uniprot/Q9H4G0	https://www.proteinatlas.org/ENSG00000088367-EPB41L1	1999	[359, 363]
Band 4.1-like protein 3 (4.1B), Differentially	EPB41L3, DAL1, KIAA0987	18	1087	Anchoring, Cross-linking	Regulate cytoskeletal organization and a number of	Esophageal squamous cell carcinoma (ESCC), lung	Spectrin-actin-binding domain	https://www.uniprot.org/uniprot/Q9Y2J2	https://www.proteinatlas.org/ENSG00000082397-EPB41L3	2000	[362, 364] [359, 360]

expressed in adenocarcinoma of the lung protein 1 (DAL-1), Erythrocyte membrane protein band 4.1-like 3					processes through multiple interactions.	adenocarcinoma, meningiomas, breast cancer, ovarian cancer, prostate cancer, cervical cancer, gastric cancer, intestinal carcinoma, colorectal cancer, hepatocellular carcinoma and pancreatic carcinoma, esophageal carcinoma, renal clear cell carcinoma (RCCC).	(SABD, 514-860)				
Talin-1	TLN1, KIAA1027, TLN	9	2541	Cross-linking, Anchoring	Stabilize focal adhesion, involve in cellular mechanotransduction, provide a connection between the cytoskeleton and the ECM.	Myelodysplastic syndromes, prostate cancer, colon cancer, hepatocellular carcinoma, breast cancer, endometrioid carcinoma, glioblastoma, oral squamous cell carcinoma.	Actin-binding sites 1-3	https://www.uniprot.org/uniprot/Q9Y490	https://www.proteinatlas.org/ENSG00000137076-TLN1	1982	[365-369]
Talin-2	TLN2, KIAA0320	15	2542	Cross-linking, Anchoring	Stabilize focal adhesion, involve in cellular mechanotransduction, provide a connection between the cytoskeleton and the ECM.	Breast cancer, hepatocellular carcinoma.	Actin-binding sites 1-3	https://www.uniprot.org/uniprot/Q9Y4G6	https://www.proteinatlas.org/ENSG00000171914-TLN2	2001	[367-372]
Talin rod domain-containing protein 1, Mesoderm development candidate 1	TLNRD1, MESDC1	15	362	Bundling	Enhance filopodia formation and cell migration.		Four-helix domain.	https://www.uniprot.org/uniprot/Q9H1K6	https://www.proteinatlas.org/ENSG00000140406-TLNRD1	2021	[373]
Transient receptor potential cation channel subfamily V member 4 (TrpV4), Osm-9-like TRP channel 4 (OTRPC4), Transient receptor potential protein 12 (TRP12), Vanilloid receptor-like channel 2, Vanilloid receptor-like protein 2 (VRL-2), Vanilloid receptor-related osmotically-activated channel (VR-OAC)	TRPV4, VRL2, VROAC	12	871	Anchoring	Regulate cell stiffness, accelerate actin dynamics and downregulates cytoskeleton-associated proteins in the cell cortex.	Epithelial cancer, metastatic breast cancer, autism, depression, brain edema, neural birth defect, thermal hyperalgesia, scapuloperoneal spinal muscular atrophy, Charcot–Marie–Tooth disease type 2C and skeletal dysplasia, Helicobacter pylori infection, hypoglycemia, alcohol addiction, or cerebrovascular disorders.	C terminal region	https://www.uniprot.org/uniprot/Q9HBA0	https://www.proteinatlas.org/ENSG00000111199-TRPV4	2007	[374-378]
Transient receptor potential cation channel subfamily V member 2 (TrpV2), Osm-9-like TRP channel 2 (OTRPC2), Vanilloid receptor-like protein 1 (VRL-1)	TRPV2, VRL	17	764	Anchoring	Regulate submembranous actin cytoskeleton influencing different cellular functions such as cell adhesion and cell spreading. Involve in neurite initiation, extension and branching events.	Hepatic cirrhosis-induced hyponatremia, breast cancer, esophageal squamous cell carcinoma (ESCC), hepatocarcinoma, glioblastoma malign, multiple myeloma, gastric cancer, melanoma, urothelial cancer, prostate cancer, acute lymphoblastic leukemia.	C terminal region	https://www.uniprot.org/uniprot/Q9Y5S1	https://www.proteinatlas.org/ENSG00000187688-TRPV2	2017	[379-381]
Aquaporin-2, AQP-2.	AQP2	12	271	Anchoring	Regulate water homeostasis.	Diabetes insipidus, nephrogenic, autosomal (ANDI).		https://www.uniprot.org/uniprot/P41181	https://www.proteinatlas.org/ENSG00000167580-AQP2	2004	[376, 382, 383]
Cystic fibrosis transmembrane conductance regulator	CFTR	7	1480	Anchoring	Regulate epithelial ion and water transport and fluid homeostasis.	Cystic fibrosis.		https://www.uniprot.org/uniprot/Q20BH0	https://www.proteinatlas.org/ENSG00000001626-CFTR	2002	[376, 384]
Chloride channel protein 2, ClC-2	CLCN2	3	898	Anchoring	Regulate cell volume, membrane potential stabilization, and signal transduction.	Epilepsy, idiopathic generalized 11 (EIG11), Juvenile absence epilepsy 2 (JAE2), Juvenile myoclonic epilepsy 8 (EJM8), Leukoencephalopa		https://www.uniprot.org/uniprot/P51788	https://www.proteinatlas.org/ENSG00000114859-CLCN2	2000	[376, 385]

						thy with ataxia (LKPAT), Hyperaldosteronism, familial, 2 (HALD2).					
H(+)/Cl(-) exchange transporter 3, ClC-3	CLCN3	4	818	Anchoring	Short isoform interacts with actin. Mediate the exchange of chloride ions against protons.	Neuronal ceroid lipofuscinosis (NCL).	C-terminal	https://www.uniprot.org/uniprot/P51790	https://www.proteinatlas.org/ENSG00000109572-CLCN3	2007	[376, 383, 386]
Chloride intracellular channel protein 1, NCC27	CLIC1, NCC27	6	241	Anchoring	Involved in regulation of the cell cycle.	Over expression is corelated with tumors.		https://www.uniprot.org/uniprot/O00299	https://www.proteinatlas.org/ENSG00000213719-CLIC1	2007	[376, 387]
Chloride intracellular channel protein 5	CLIC5	6	410	Anchoring	Regulate formation of stereocilia, podocyte, and lens suture in the eye.	Deafness, autosomal recessive, 103 (DFNB103).		https://www.uniprot.org/uniprot/Q9NZA1	https://www.proteinatlas.org/ENSG00000112782-CLIC5	2007	[376, 387]
Amiloride-sensitive sodium channel subunit alpha	SCNN1A, SCNN1	12	669	Anchoring	Regulate electrolyte, blood pressure, and airway surface liquid homeostasis.	Pseudohypoaldosteronism 1, autosomal recessive (PHA1B), Bronchiectasis with or without elevated sweat chloride 2 (BESC2), Liddle syndrome 3 (LIDLS3).	C-terminal	https://www.uniprot.org/uniprot/P37088	https://www.proteinatlas.org/ENSG00000111319-SCNN1A	1996	[376, 388, 389]
Calcium-activated potassium channel subunit alpha-1, MaxiK	KCNMA1, KCNMA, SLO	10	1236	Anchoring	Regulate repolarization of the membrane potential.	Paroxysmal nonkinesigenic dyskinesia, 3, with or without generalized epilepsy (PNKD3), Cerebellar atrophy, developmental delay, and seizures (CADEDS), Liang-Wang syndrome (LIWAS).		https://www.uniprot.org/uniprot/Q12791	https://www.proteinatlas.org/search/KCNMA1	2005	[376, 390]
Voltage-dependent anion-selective channel protein 1, VDAC-1	VDAC1, VDAC	5	283	Anchoring	Regulate cell volume regulation and apoptosis.	Cancer, neurodegenerative disease, muscular dystrophy.		https://www.uniprot.org/uniprot/P21796	https://www.proteinatlas.org/ENSG00000213585-VDAC1	2001	[376, 383, 391, 392]
Voltage-dependent L-type calcium channel subunit beta-1, CAB1	CACNB1, CACNLB1	17	598	Anchoring	Regulate the activity of L-type calcium channels			https://www.uniprot.org/uniprot/Q02641	https://www.proteinatlas.org/ENSG00000067191-CACNB1	2015	[393]
Nexilin F-actin binding protein, Nelin	NEXN	1	675	Polymerization, Cross-linking	Stimulate cell migration and adhesion. Maintenan Z line and sarcomere integrity.	Coronary artery disease, congenital heart disease, atrial septal defect, hypertrophic cardiomyopathy.	Central ABD	https://www.uniprot.org/uniprot/Q0ZGT2	https://www.proteinatlas.org/ENSG00000162614-NEXN	1998	[394-396]
Actin binding Rho-activating protein, Striated muscle activator of Rho-dependent signaling (STARS)	ABRA	8	381	Anchoring	Specifically expressed in cardiac and skeletal muscle cells. Bind to the I-band of the sarcomere. Act as a mechanosensor that translates skeletal muscle-specific stimuli into intracellular signals to promote serum-response factor -dependent gene transcription.	Cardiac hypertrophy and myopathy, ageing, type 2 diabetic muscle, skeletal muscle hypertrophy and atrophy.	199-299, 300-381	https://www.uniprot.org/uniprot/Q8N0Z2	https://www.proteinatlas.org/ENSG00000174429-ABRA	2002	[397, 398]
Actin-binding LIM protein 1 (abLIM-1), Actin-binding LIM protein family member 1, Actin-binding double zinc finger protein, LIMAB1, Limatin	ABLIM1, ABLIM, KIAA0059, LIMAB1	10	778	Scaffolding	Connect actin filaments and cytoplasmic targets.	Nasopharyngeal Carcinoma, Hepatocellular Carcinoma, Myotonic dystrophy type 1 (DM1).	dematin-like domain	https://www.uniprot.org/uniprot/O14639	https://www.proteinatlas.org/ENSG00000099204-ABLIM1	1997	[399, 400]
Actin-binding LIM protein 2 (abLIM-2), Actin-binding LIM protein family member 2	ABLIM2, KIAA1808	4	611	Scaffolding	Enhance STARS-dependent activation of serum-response factor.	Breast carcinogenesis, periodontitis, Alzheimer's Disease.	villin domain	https://www.uniprot.org/uniprot/Q6H8Q1	https://www.proteinatlas.org/ENSG00000163995-ABLIM2	2005	[401, 402]
Actin-binding LIM protein 3 (abLIM-3), Actin-binding LIM protein family member 3	ABLIM3, KIAA0843	5	683	Scaffolding	Enhance STARS-dependent activation of serum-response factor.Involved in anchoring LIM domain-binding components of adherens junctions to circumferential actin bundles.		villin domain	https://www.uniprot.org/uniprot/O94929	https://www.proteinatlas.org/ENSG00000173210-ABLIM3	2006	[402-404]
Tight junction protein ZO-1, Tight junction protein 1, Zona	TJP1, ZO1	15	1748	Anchoring, Scaffolding	Link tight junction transmembrane proteins such as claudins and	Inflammatory bowel disease, Kawasaki disease, Parkinson's	1151-1371	https://www.uniprot.org/uniprot/Q07157	https://www.proteinatlas.org/ENSG00000104067-TJP1	1986	[405, 406]

occludens protein 1, Zonula occludens protein 1					occludin to the actin cytoskeleton.	disease, liver cancer, squamous cell carcinoma, Bowen's disease, celiac disease, gastrointestinal stromal tumor, endometrial carcinoma.					
Tight junction protein ZO-2, Zonula occludens protein 2	TJP2, ZO2	9	1190	Anchoring, Scaffolding	Link tight junction transmembrane proteins such as claudins and occludin to the actin cytoskeleton.	Familial hypercholanemia (FHCA), Cholestasis, progressive familial intrahepatic, 4 (PFIC4).		https://www.uniprot.org/uniprot/Q9UDY2	https://www.proteinatlas.org/ENSG00000119139-TJP2	1994	[407, 408]
Tight junction protein ZO-3, onula occludens protein 3	TJP3, ZO3	19	919	Anchoring, Scaffolding	Link tight junction transmembrane proteins such as claudins and occludin to the actin cytoskeleton.			https://www.uniprot.org/uniprot/O95049	https://www.proteinatlas.org/search/TJP3	1998	[408, 409]
Occludin	OCLN	5	522	Anchoring, Scaffolding	Transmembrane protein that forms and regulate tight junction. Maintain blood-brain barrier.	Pseudo-TORCH syndrome 1 (PTORCH1)		https://www.uniprot.org/uniprot/Q16625	https://www.proteinatlas.org/ENSG00000197822-OCLN	1993	[408, 410]
LIM and SH3 domain protein 1 (LASP1), Metastatic lymph node gene 50 protein (MLN 50)	LASP1, MLN50	17	261	Scaffolding	Enhance cancer cell migration and cell invasion. Involved in the differentiation and development of neurons. Involve in vesicular secretion.	Gastric carcinoma (GC), bladder cancer, esophageal squamous cell carcinoma, Breast carcinoma, Colorectal carcinoma, Ovarian carcinoma, Hepatocellular carcinoma, Renal cell carcinoma, Prostate carcinoma (PC), Medulloblastoma, Nasopharyngeal carcinoma (NC), Non-small lung cancer, Lung Adenocarcinoma, Choriocarcinoma, Gall bladder carcinoma, Thyroid carcinoma, Pancreatic carcinoma.	Nebulin-like repeat (NR1, NR2)	https://www.uniprot.org/uniprot/Q14847	https://www.proteinatlas.org/ENSG00000002834-LASP1	1998	[411, 412]
Nebulette, Actin-binding Z-disk protein	NEBL, C10orf113, LNEBL	10	1014	Bundling	Enhances cancer cell migration but reduces cell invasion. Involve in cell spreading	Colorectal cancer, non-small cell lung cancer (NSCLC).	Nebulin-like repeat (NR1, NR2, NR3)	https://www.uniprot.org/uniprot/O76041	https://www.proteinatlas.org/ENSG00000078114-NEBL	1995	[412-414]
Mitotic interactor and substrate of PLK1, Mitotic spindle positioning protein	MISP, C19orf21	19	679	Bundling	Effect on cellular morphology by inducing stress fiber-like thick filaments or filopodial formations. Ensure correct NuMA polarization and spindle orientation.		C-terminal	https://www.uniprot.org/uniprot/Q8IVT2	https://www.proteinatlas.org/ENSG0000099812-MISP	2013	[415-418]
Actin-histidine N-methyltransferase, Protein-L-histidine N-tele-methyltransferase, SET domain-containing protein 3 (hSETD3)	SETD3, C14orf154	14	594	Polymerization	Mediate histidine methylation on β-actin, and accelerate the assembly of actin filaments.	Muscle loading and hypertrophy	SET domain (94 – 314)	https://www.uniprot.org/uniprot/Q86TU7	https://www.proteinatlas.org/ENSG00000183576-SETD3	2018	[419, 420]
Synaptopodin	SYNPO, KIAA1029	5	929	Scaffolding	Regulate the integrity of the podocyte actin cytoskeleton and for the regulation of podocyte cell migration.	Alport syndrome (AS), eosinophilic esophagitis, autism, schizophrenia.		https://www.uniprot.org/uniprot/Q8N3V7	https://www.proteinatlas.org/ENSG00000171992-SYNPO	1997	[421-423]
Synaptopodin-2, Genethonin-2, Myopodin	SYNPO2	4	1093	Bundling	Participate in signaling pathways between the Z-disc and the nucleus.	Urothelial cancer, prostate cancer, bladder cancer.	410-563	https://www.uniprot.org/uniprot/Q9UMS6	https://www.proteinatlas.org/ENSG00000172403-SYNPO2	2001	[422, 424]
Synaptopodin 2-like protein	SYNPO2L	10	977	Bundling	Required for cardiac and skeletal muscle development.	Atrial fibrillation, myofibrillar myopathies (MFMs), cardiomyopathy and contractile dysfunction.		https://www.uniprot.org/uniprot/Q9H987	https://www.proteinatlas.org/ENSG00000166317-SYNPO2L	2009	[422, 425]
Smoothelin	SMTN	22	917	Bundling, Scaffolding	Implicated in cell contraction and mediate the interaction between actin filaments and other components of the cytoskeleton.	Atherosclerosis and restenosis, cardiac hypertrophy, essential hypertension, cerebral infarction, smooth muscle hamartoma,	Multiple domains	https://www.uniprot.org/uniprot/P53814	https://www.proteinatlas.org/ENSG00000183963-SMTN	1996	[426-429]

						prostate cancer, glomus tumor, urinary bladder carcinoma, colorectal adeno-carcinoma, smooth muscle myopathy.					
Smoothelin-like protein 2	SMTNL2	17	461	Stabilizatio n	Regulate actin dynamics during epithelial morphogenesis and the developmental control of the cellular cortex.	Rheumatoid arthritis, diabetes, middle cerebral artery occlusion, non-small cell lung cancer, Brachmann-Cornelia de Lange syndrome.		https://www.unipr ot.org/uniprot/Q2T AL5	https://www.protei natlas.org/ENSG0 0000188176-SMTNL2	2021	[430]
Myc box-dependent-interacting protein 1, Amphiphysin II, Amphiphysin-like protein, Box-dependent myc-interacting protein 1, Bridging integrator 1	BIN1, AMPHL	2	593	Stabilizatio n	Involved in endocytosis, actin cytoskeletal organization, transcription, and stress responses.	Alzheimer's disease, heart failure (HF), malignant arrhythmia, breast, colon, prostate and lung cancers, hepatocarcinoma, neuroblastoma, centronuclear myopathy (CNM) and myotonic dystrophy (DM), ventricular arrhythmia	Bin/Amphi physin/Rvs (BAR) domain	https://www.unipr ot.org/uniprot/O00 499	https://www.protei natlas.org/ENSG0 0000136717-BIN1	1997	[431-433]
Catenin alpha-1, Alpha E-catenin, Cadherin-associated protein, Renal carcinoma antigen NY-REN-13	CTNNA1	5	906	Bundling, Anchoring	Guide the establishment of classical epithelial cell polarity and contribute to the control of migration, growth, and differentiation.	Gastrointestinal cancer, colon cancer, dilated cardiomyopathy, cardiac injury, hereditary diffuse gastric cancer (HDGC), invasive lobular breast cancer (LBC), colitis.	C-terminal	https://www.unipr ot.org/uniprot/P35 221	https://www.protei natlas.org/ENSG0 0000044115-CTNNA1	1995	[434]
EH domain-binding protein 1	EHBP1, KIAA0903, NACSIN	2	1231	Anchoring	Link endosomes to the actin cytoskeleton.	Prostate cancer, colorectal cancer, pulmonary arterial hypertension, rectal cancer.	Calponin homology (CH) domain	https://www.unipr ot.org/uniprot/Q8N DI1	https://www.protei natlas.org/ENSG0 0000115504-EHBP1	2004	[435, 436]
Ras GTPase-activating-like protein IQGAP1, p195	IQGAP1, KIAA0051	15	1657	Cross-linking	Regulate mitogen-activated protein kinase (MAPK) signaling, Ca2+/calmodulin signaling, cell-cell adhesion, β-catenin-mediated transcription and microbial invasion.	Colorectal cancer, glioma, lung cancer, head and neck squamous cell, astrocytoma, breast cancer, gastric cancer, ovarian cancer, metastatic melanoma.	Calponin homology (CH) domain	https://www.unipr ot.org/uniprot/P46 940	https://www.protei natlas.org/ENSG0 0000140575-IQGAP1	1997	[437-440]
Ras GTPase-activating-like protein IQGAP2	IQGAP2	5	1575	Cross-linking	Predicted to bind to F-actin due to sequence similarity.	Carcinoma.	Calponin homology (CH) domain	https://www.unipr ot.org/uniprot/Q13 576	https://www.protei natlas.org/ENSG0 0000145703-IQGAP2	1996	[440, 441]
Ras GTPase-activating-like protein IQGAP3	IQGAP3	1	1631	Cross-linking	Link the activation of Rac1 and Cdc42 with the cytoskeletal architectures during neuronal morphogenesis.	Colorectal cancer, ovarian cancer, lung cancer, gastric cancer, colorectal cancer, hepatocellular carcinoma, breast cancer, clear cell renal cell carcinoma, bladder cancer, pancreatic cancer.	Calponin homology (CH) domain	https://www.unipr ot.org/uniprot/Q86 VI3	https://www.protei natlas.org/ENSG0 0000183856-IQGAP3	2007	[439, 440, 442, 443]
LIM domain-binding protein 3, Protein cypher, Z-band alternatively spliced PDZ-motif protein	LDB3, KIAA0613, ZASP	10	727	Monomer binding, F-actin binding	Effect on the core structure of the Z-discs in skeletal muscle.	Markesbery disease, myofibrillar myopathies (MFM), hypertrophic cardiomyopathy (HCM), dilated cardiomyopathy (DCM), arrhythmogenic right ventricular cardiomyopathy (ARVC), left ventricular noncompaction (LVNC).	ABD between PDZ and LIM	https://www.unipr ot.org/uniprot/O75 112	https://www.protei natlas.org/ENSG0 0000122367-LDB3	2014	[444-446]
Myocardin-related transcription factor A (MRTF-A), MKL/myocardi n-like protein 1, Megakaryoblas tic leukemia 1 protein, Megakaryocyti c acute leukemia protein	MRTFA, KIAA1438, MAL, MKL1	22	931	Monomer binding	Transcription coactivator that associates with the serum response factor.	Acute megakaryoblastic leukemia, Immunodeficiency 66 (IMD66)	RPEL motif	https://www.unipr ot.org/uniprot/Q96 9V6	https://www.protei natlas.org/ENSG0 0000196588-MRTFA	2003	[447]
Protein MTSS 1, Metastasis suppressor YGL-1, Metastasis suppressor	MTSS1, KIAA0429, MIM	8	755	Monomer binding, Bundling, Scaffolding	Regulate cell morphology, motility, metastasis. Act as a scaffold protein that interacts with	Prostate cancer, breast cancer, acute myeloid leukemia, pancreatic cancer, intrahepatic	WH2 domain, IRSp53/MI M domain	https://www.unipr ot.org/uniprot/O43 312	https://www.protei natlas.org/ENSG0 0000170873-MTSS1	2003	[448-450]

protein 1, Missing in metastasis protein					Rac, actin and actin-associated proteins to modulate lamellipodia formation.	cholangiocarcinoma, pancreatic ductal adenocarcinoma, bladder uroepithelium cell carcinoma, hepatocellular carcinoma, colorectal cancer, esophageal cancer, tongue squamous cellular carcinoma, gastric cancer, melanomas.					
Rab effector MyRIP, Exophilin-8, Myosin-VIIa- and Rab-interacting protein, Synaptotagmin-like protein lacking C2 domains C (SlaC2-c)	MYRIP, SLAC2C	3	859	Anchoring	Regulate Weibel-Palade body trafficking and exocytosis.	Left ventricular hypertrophy, hepatocellular carcinoma.	Slac2-a (400-590) and Slac2-c (670-856)	https://www.uniprot.org/uniprot/Q8NFW9	https://www.proteinatlas.org/ENSG00000170011-MYRIP	2002	[451-453]
Nebulin	NEB	2	6669	Nucleation, Stabilization	Form composite thin filaments in the skeletal muscle sarcomere.	Core-rod myopathy, distal myopathy	Nebulin repeats	https://www.uniprot.org/uniprot/P20929	https://www.proteinatlas.org/ENSG00000183091-NEB	1991	[454-457]
Nebulin-related-anchoring protein (N-RAP)	NRAP	10	1730	Anchoring	Act as an organizing center for the initial recruitment and assembly of sarcomeric actin filaments and Z-discs.	Dilated cardiomyopathy (DCM),	Nebulin repeats	https://www.uniprot.org/uniprot/Q86VF7	https://www.proteinatlas.org/ENSG00000197893-NRAP	1997	[456, 458]
Vinculin, Metavinculin (MV)	VCL	10	1134	Capping, Anchoring	Interact with F-actin both in recruitment of actin filaments to the growing focal adhesions and also in capping of actin filaments to prevent actin polymerization.	Chagas cardiomyopathy, dilated cardiomyopathy, hypertrophic cardiomyopathy, left ventricular assist device (LVAD).	Vinculin tail domain (Vt)	https://www.uniprot.org/25niport/P18206	https://www.proteinatlas.org/ENSG00000035403-VCL	1981	[459-462]
Cysteine and glycine-rich protein 3, Cardiac LIM protein, Cysteine-rich protein 3 (CRP3), LIM domain protein, cardiac, Muscle LIM protein	CSRP3, CLP, MLP	11	194	Bundling	Facilitate filopodia formation and increasing growth cone motility. Act as a mechanical stress sensor, entering in nuclei and modulating gene expression.	Dilated cardiomyopathy, hypertrophic cardiomyopathy, ischemic cardiomyopathy, Nemaline myopathy (NM), neuromuscular disorder facioscapulohumeral muscular dystrophy (FSHD).	LIM motif	https://www.uniprot.org/uniprot/P50461	https://www.proteinatlas.org/ENSG00000129170-CSRP3	1996	[463-465]
Utrophin, Dystrophin-related protein 1 (DRP-1)	UTRN, DMDL, DRP1	6	3433	Anchoring	Perform 'spacer' or 'shock absorber' role as dystrophin in mature muscle tissues.	Duchenne muscular dystrophy (DMD), Becher muscular dystrophy.	CH1(31-135), CH2(150-255)	https://www.uniprot.org/uniprot/P46939	https://www.proteinatlas.org/ENSG00000152818-UTRN	1994	[342, 466, 467]
Growth arrest-specific 7 (GAS-7)	GAS7, KIAA0394	17	476	Polymerization, Cross-linking	Mediate reorganization of microfilaments and induce the formation of extended cellular processes.	Glaucoma, Alzheimer disease, schizophrenia.	C terminal	https://www.uniprot.org/uniprot/O60861	https://www.proteinatlas.org/ENSG00000007237-GAS7	2002	[468]
Calicin	CCIN	9	588	F-actin binding	Target of calicin at the subacrosomal space of round spermatids, and that its ability to form homomultimers contributes to the formation of a rigid calyx.			https://www.uniprot.org/uniprot/Q13939	https://www.proteinatlas.org/ENSG00000185972-CCIN	2000	[469]
Cyclin-dependent kinase 5 activator 1 (CDK5 activator 1), Cyclin dependent kinase 5 regulatory subunit 1, TPKII regulatory subunit	CDK5R1, CDK5R, NCK5A	17	307	Scaffolding	Involved in dendritic spine morphogenesis and circadian clock.	Hepatocellular carcinoma, Alzheimer's disease, non-syndromic intellectual disability, prostate cancer, high-functioning autistic, Parkinson's disease.		https://www.uniprot.org/uniprot/Q15078	https://www.proteinatlas.org/ENSG00000176749-CDK5R1	2011	[470]
Ermin, Juxtanodin (JN)	ERMN, KIAA1189	2	284		Induce the formation of numerous cell protrusions and a pronounced change in cell morphology.	Multiple Sclerosis (MS)	265-284	https://www.uniprot.org/uniprot/Q8TAM6	https://www.proteinatlas.org/ENSG00000136541-ERMN	2005	[471-473]
FK506-binding protein 15 (FKBP-15), 133 kDa FK506-binding protein (133 kDa FKBP,	FKBP15, KIAA0674	9	1219		Involve in the transport of early endosomes at the level of transition between microfilament-based and	Inflammatory bowel disease (IBD).		https://www.uniprot.org/uniprot/Q5T1M5	https://www.proteinatlas.org/ENSG00000119321-FKBP15	2009	[474]

FKBP-133), WASP- and FKBP-like protein (WAFL)					microtubule-based movement.						
Growth arrest-specific protein 2 (GAS-2)	GAS2	11	313	Cross-linking	Cross-link microtubule and actin cytoskeletons.	Liver cancer, leukemia, recurrent colorectal cancer, prostate cancer, breast cancer, lung adenocarcinoma.	CH (34-156)	https://www.uniprot.org/uniprot/O43903	https://www.proteinatlas.org/ENSG00000148935-GAS2	1992	[475, 476]
GAS2-like protein 1, GAS2-related protein on chromosome 22, Growth arrest-specific protein 2-like 1	GAS2L1, GAR22	22	681	Cross-linking	Cross-link microtubule and actin cytoskeletons.	Acute myeloid leukemia, meningioma.	CH (27-148)	https://www.uniprot.org/uniprot/Q99501	https://www.proteinatlas.org/ENSG00000185340-GAS2L1	2003	[476, 477]
GAS2-like protein 2, GAS2-related protein on chromosome 17, Growth arrest-specific 2-like 2	GAS2L2, GAR17	17	880	Cross-linking	Cross-link microtubule and actin cytoskeletons.	Cancer with painful bone metastases, Ciliary dyskinesia, primary, 41 (CILD41).	CH (32-159)	https://www.uniprot.org/uniprot/Q8NHY3	https://www.proteinatlas.org/ENSG00000270765-GAS2L2	2003	[476, 477]
GAS2-like protein 3, Growth arrest-specific 2-like 3	GAS2L3	12	694	Cross-linking	Cross-link microtubule and actin cytoskeletons.	Glioma	CH (48-168)	https://www.uniprot.org/uniprot/Q86XJ1	https://www.proteinatlas.org/ENSG00000139354-GAS2L3	2011	[476, 478]
Kelch-like protein 2, Actin-binding protein Mayven	KLHL2	4	593	Scaffolding	Translocate along axonal processes and involved in the dynamic organization of the actin cytoskeleton in brain cells.	Renal cell carcinoma, acute myeloid leukemia (AML), ovarian cancer, familial hyperkalemic hypertension, acute peanut allergic reaction, septic-shock-associated acute kidney injury, multiple myeloma.	Kelch repeats	https://www.uniprot.org/uniprot/O95198	https://www.proteinatlas.org/ENSG00000109466-KLHL2	1999	[479]
Kelch-like protein 20, Kelch-like ECT2-interacting protein, Kelch-like protein X	KLHL20, KHLHX, KLEIP	1	609	Scaffolding	Involved in anterograde Golgi to endosome transport. Regulate corneal epithelial integrity.	Colon cancer	Kelch repeats	https://www.uniprot.org/uniprot/Q9Y2M5	https://www.proteinatlas.org/ENSG00000076321-KLHL20	2004	[480]
Actin-binding protein IPP, Intracisternal A particle-promoted polypeptide (IPP), Kelch-like protein 27	IPP, KLHL27	1	584	Anchoring	Regulate cell adhesion.	Breast cancer	Kelch repeats	https://www.uniprot.org/uniprot/Q9Y573	https://www.proteinatlas.org/ENSG00000197429-IPP	1999	[481, 482]
Protein ITPRID2, Cleavage signal-1 protein (CS-1), ITPR interacting domain-containing 2, Ki-ras-induced actin-interacting protein, Sperm-specific antigen 2	ITPRID2, CS1, KIAA1927, KRAP, SSFA2	2	1259	Scafolding	Regulate energy metabolism.	Colorectal cancer, pancreatic cancer, small cell lung cancer, obesity and diabetes, lung squamous cell cancer, chronic lymphocytic leukemia		https://www.uniprot.org/uniprot/P28290	https://www.proteinatlas.org/ENSG00000138434-ITPRID2	2004	[483, 484]
Melanophilin, Exophilin-3, Slp homolog lacking C2 domains a (SlaC2-a), Synaptotagmin-like protein 2a	MLPH, SLAC2A	2	600	Scafolding	Involved in in actin-based melanosome transport.	Rectal cancer, prostate cancer, allergic and inflammatory disease, Inherited diseases of pigmentation.	401-590	https://www.uniprot.org/uniprot/Q9BV36	https://www.proteinatlas.org/ENSG00000115648-MLPH	2002	[451]
Oligophrenin-1	OPHN1	X	802	Scafolding	Regulate Rho GTPase activity in platelets and induce a reorganization of actin.	Syndromic X-linked mental retardation with epilepsy, rostral ventricular enlargement and cerebellar hypoplasia, prostate cancer.	C-terminal	https://www.uniprot.org/uniprot/O60890	https://www.proteinatlas.org/ENSG00000079482-OPHN1	2003	[485, 486]
Pleckstrin homology domain-containing family H member 2	PLEKHH2, KIAA2028	2	1493	Stabilization	Slow down actin depolymerization.	Diabetic nephropathy, schizophrenia.	FERM domain	https://www.uniprot.org/uniprot/Q8IVE3	https://www.proteinatlas.org/ENSG00000152527-PLEKHH2	2012	[487]
SH2B adaptor protein 1, Pro-rich, PH and SH2 domain-containing signaling mediator (PSM), SH2 domain-containing protein 1B	SH2B1, KIAA1299, SH2B	16	756	Cross-linking	Regulate cultured cell morphology, motility and adhesion.	Obesity, leptin and insulin resistance, non-small cell lung cancer (NSCLC), esophageal cancer, gastric cancer, colorectal cancer, oropharyngeal cancer	150–200 and 615–670.	https://www.uniprot.org/uniprot/Q9NRF2	https://www.proteinatlas.org/ENSG00000178188-SH2B1	2009	[488, 489]

Annexin A1, Annexin I, Annexin-1, Calpactin II, Calpactin-2, Chromobindin-9, Lipocortin I, Phospholipase A2 inhibitory protein	ANXA1, ANX1, LPC1	9	346	Bundling, Anchoring	Mediate the Ca2+-dependent interaction between phagosomes and the actin cytoskeleton.	Hepatocellular carcinoma, lung cancer, melanoma colorectal cancer, and pancreatic cancer, esophageal cancer, prostate cancer, cervical cancer, B-cell non-Hodgkin's lymphomas, larynx cancer, nasopharyngeal carcinoma (NPC) and oral squamous cell carcinoma, multiple sclerosis, Alzheimer's disease, atherosclerosis, coronary artery disease, myocardial infarction, diabetic nephrophthy, stroke.	IRI motif	https://www.uniprot.org/uniprot/P04083	https://www.proteinatlas.org/ENSG00000135046-ANXA1	1986	[490-497]
Annexin A2, Annexin II, Annexin-2, Calpactin I heavy chain, Calpactin-1 heavy chain, Chromobindin-8, Lipocortin II, Placental anticoagulant protein IV (PAP-IV, Protein I), p36	ANXA2, ANX2, ANX2L4, CAL1H, LPC2D	15	339	Bundling, Anchoring	Maintain the plasticity of the dynamic membrane-associated actin cytoskeleton.	Breast cancer, glioblastoma, renal cell carcinoma, hepatocellular carcinoma, colorectal cancer, lung cancer, bacteria, fungus, virus infection, acute and chronic inflammatory disorder.	IRI motif	https://www.uniprot.org/uniprot/P07355	https://www.proteinatlas.org/ENSG00000182718-ANXA2	1984	[493, 498-500]
Annexin A5, Anchorin CII, Annexin V, Annexin-5, Calphobindin I (CBP-I), Endonexin II, Lipocortin V, Placental anticoagulant protein 4 (PP4), Placental anticoagulant protein I (PAP-I), Thromboplastin inhibitor, Vascular anticoagulant-alpha (VAC-alpha)	ANXA5, ANX5, ENX2, PP4	4	320	Anchoring		Hepatocarcinoma, breast cancer, cervical carcinoma, gastric cancer, nasopharyngeal carcinoma, colorectal cancer, pancreatic cancer, bladder cancer, prostate cancer, squamous cell carcinoma, glioma, sarcoma, thyroid cancer.		https://www.uniprot.org/uniprot/P08758	https://www.proteinatlas.org/ENSG00000164111-ANXA5	1988	[493, 501-503]
Annexin A6, 67 kDa calelectrin, Annexin VI, Annexin-6, Calphobindin-II (CPB-II), Chromobindin-20, Lipocortin VI, Protein III, p68, p70	ANXA6, ANX6	5	673	Anchoring	Regulate microfilament architecture, in response to calcium signals in neurites.	Breast cancer, esophageal adenocarcinoma.		https://www.uniprot.org/uniprot/P08133	https://www.proteinatlas.org/ENSG00000197043-ANXA6	1990	[493, 504, 505]
Annexin A8, Annexin VIII, Annexin-8, Vascular anticoagulant-beta, VAC-beta.	ANXA8, ANX8	10	327	Anchoring	Regulate late endosomes organization.	Gastric carcinoma.		https://www.uniprot.org/uniprot/P13928	https://www.proteinatlas.org/ENSG00000265190-ANXA8	2006	[506]
Girdin, Akt phosphorylation enhancer (APE), Coiled-coil domain containing protein 88A, G alpha-interacting vesicle-associated protein (GIV), Girders of actin filament, Hook-related protein 1 (HkRP1)	CCDC88A, APE, GRDN, KIAA1212	2	1871	Cross-linking, Bundling, Anchoring	Form actin bundles, and anchor the bundles to the plasma membrane at the cortical region of the cells. Facilitate lamellar protrusion, cell migration, adhesion, and invasion.	Pancreatic cancer, breast cancer, lung cancer, cervical carcinoma, colorectal cancer, gastric cancer, hepatocellular carcinoma, acute myocardial infarction, Alzheimer's disease (AD).	C-terminal 2	https://www.uniprot.org/uniprot/Q3V6T2	https://www.proteinatlas.org/ENSG00000115355-CCDC88A	2005	[507, 508]
Lymphocyte-specific protein 1, 47 kDa actin-binding protein, 52 kDa phosphoprotein (pp52), Lymphocyte-specific antigen WP34	LSP1, WP34	11	339	Bundling	Regulate focal adhesion dynamics, cell migration and phagocytosis.	Hodgkin's disease, breast cancer, Sjögren's syndrome, acute lung inflammation, rheumatoid arthritis, hepatocellular carcinoma, schizophrenia, carotid paragangliomas.	C-terminal basic domain	https://www.uniprot.org/uniprot/P33241	https://www.proteinatlas.org/ENSG00000130592-LSP1	1992	[509, 510]

[F-actin]-monooxygenase MICAL1, Molecule interacting with CasL protein 1 (MICAL1), NEDD9-interacting protein with calponin homology and LIM domains	MICAL1, MICAL, NICAL	6	1067	PT modification, Depolymerization	Directly oxidizes Met44 and Met47 of F-actin into methionine-R-sulfoxides resulting in F-actin disassembly.	Different cancers, diabetic nephropathy, blood brain barrier dysfunction, muscular dystrophy, liver disease, susceptibility to infection, epilepsy, Alzheimer's disease, aging, deafness, wide-ranging neurological disorders, skeletal abnormalities, and obesity.	FAD	https://www.uniprot.org/uniprot/Q8TDZ2	https://www.proteinatlas.org/ENSG00000135596-MICAL1	2010	[511-513]
[F-actin]-monooxygenase MICAL2, Molecule interacting with CasL protein 2 (MICAL-2)	MICAL2, KIAA0750, MICAL2PV1, MICAL2PV2	11	1124	PT modificatio, Depolymerization	Induce loss of actin stress fibers and the generation of actin-rich protrusions.	Colorectal, breast, gastric, prostate, bladder Cancer, lung adenocarcinoma, laryngeal squamous cell carcinoma.	FAD	https://www.uniprot.org/uniprot/O94851	https://www.proteinatlas.org/ENSG00000133816-MICAL2	2010	[511-515]
[F-actin]-monooxygenase MICAL3, Molecule interacting with CasL protein 3, MICAL-3.	MICAL3, KIAA0819, KIAA1364	22	2002	PT modificatio, Depolymerization	Directly oxidizes Met44 and Met47 of F-actin into methionine-R-sulfoxides resulting in F-actin disassembly.			https://www.uniprot.org/uniprot/Q7RTP6	https://www.proteinatlas.org/ENSG00000243156-MICAL3	2012	[512]
Methionine-R-sulfoxide reductase B1 (MsrB1), Selenoprotein X (SelX)	MSRB1, SEPX1	16	116	PT modificatio, Polymerization	Regulate actin assembly by reducing methionine (R)-sulfoxide mediated by MICALs on actin, thereby promoting polymerization.			https://www.uniprot.org/uniprot/Q9NZV6	https://www.proteinatlas.org/ENSG00000198736-MSRB1	2013	[516]
Methionine-R-sulfoxide reductase B2, mitochondrial (MsrB2)	MSRB2, CBS-1, MSRB	10	182	PT modification, Polymerization	Regulate actin assembly by reducing methionine (R)-sulfoxide mediated by MICALs on actin, thereby promoting polymerization.	Parkinson's disease, Alzheimer's disease.		https://www.uniprot.org/uniprot/Q9Y3D2	https://www.proteinatlas.org/ENSG00000148450-MSRB2	2020	[517]
N-alpha-acetyltransferase 80 (HsNAAA80) N-acetyltransferase 6, Protein fusion-2 (Protein fus-2)	NAA80, FUS2, NAT6	3	286	PT modification, Polymerization	Regulate actin assembly and cell motility.	Glioblastoma, low-grade gliomas (LGG), kidney cancers.		https://www.uniprot.org/uniprot/Q93015	https://www.proteinatlas.org/ENSG00000243477-NAA80	2018	[518-521]
Prolactin-inducible protein, gross cystic disease fluid protein 15, secretory actin-binding protein (SABP, gp17)	PIP, GCDFP15, GPIP4	7	146	Scaffolding	Regulate antitumor immunity and metastasis.	Lung cancer, breast cancer, Sjogren's Syndrome, metastatic carcinoma, mucinous carcinoma of skin, uterine leiomyomas.		https://www.uniprot.org/uniprot/P12273	https://www.proteinatlas.org/ENSG00000159763-PIP	1990	[522, 523]
Plectin (PCN, PLTN), Hemidesmosomal protein 1 (HD1), Plectin-1	PLEC, PLEC1	8	4684	Cross-linking	Connect intermediate filaments with microtubules and actin filaments. Form desmosomes and hemidesmosomes.	Pancreatic ductal carcinoma (PDAC), pancreatic, lung, esophageal, stomach, ovarian, and breast cancer, head and neck squamous cell carcinoma (HNSCC), oral squamous cell carcinoma (OSCC), hepatocellular carcinoma (HCC), epidermolysis bullosa simplex (EBS).	CH1 (179 – 282), CH2 (295 – 400)	https://www.uniprot.org/uniprot/Q15149	https://www.proteinatlas.org/ENSG00000178209-PLEC	1992	[524-526]
Shootin-1	SHTN1, KIAA1598	10	631	Cross-linking	Mediate actin filament retrograde flow and L1-CAM in axonal growth cones.	Age-related macular degeneration (AMD)	WH2	https://www.uniprot.org/uniprot/A0MZ66	https://www.proteinatlas.org/ENSG00000187164-SHTN1	2008	[527-529]
Angiomotin	AMOT, KIAA1071	X	1084	Anchoring, Scaffolding	Regulate tube formation and migration of endothelial cells and the regulation of tight junctions, polarity, and epithelial-mesenchymal transition in epithelial cells.	Breast cancer, osteosarcoma, prostate cancer, head and neck squamous cell carcinoma (HNSCC), hepatic carcinoma, renal cell cancer, ovarian cancer, lung cancer.	N-terminal	https://www.uniprot.org/uniprot/Q4VCS5	https://www.proteinatlas.org/ENSG00000126016-AMOT	2005	[530-532]
Anthrax toxin receptor 1, Tumor endothelial marker 8	ANTXR1, ATR, TEM8	2	564	Bundling, Anchoring	Mediate cell spreading by coupling extracellular	Gastric cancer, glioma, sickle cell disease, breast cancer, pancreatic cancer, colorectal	Cytosolicdomain	https://www.uniprot.org/uniprot/Q9H6X2	https://www.proteinatlas.org/ENSG00000169604-ANTXR1	2006	[533-536]

					ligands to the actin cytoskeleton.	cancer, hepatocellular carcinoma, growth retardation-alopecia-pseudoanodontia-optic atrophy (GAPO) syndrome, melanoma, breast, colon, and lung cancer, virus infection.					
CLIP-associating protein 1, Cytoplasmic linker-associated protein 1, Multiple asters homolog 1, Protein Orbit homolog 1 (hOrbit1)	CLASP1, KIAA0622, MAST1	2	1538	Cross-linking	Link plus ends of growing microtubules and actin filaments.	Prostate cancer.	Middle serine-arginine rich motif, dis1/TOG motif	https://www.uniprot.org/uniprot/Q7Z460	https://www.proteinatlas.org/ENSG00000074054-CLASP1	2007	[537]
CLIP-associating protein 2, Cytoplasmic linker-associated protein 2, Protein Orbit homolog 2 (hOrbit2)	CLASP2, KIAA0627	3	1294	Cross-linking	Link plus ends of growing microtubules and actin filaments.	Bladder tumor, endothelial inflammation and lung injury.	Middle serine-arginine rich motif, dis1/TOG motif	https://www.uniprot.org/uniprot/O75122	https://www.proteinatlas.org/ENSG00000163539-CLASP2	2007	[537]
Dixin, Coiled-coil protein DIX1 (Coiled-coil-DIX1), DIX domain-containing protein 1	DIXDC1, CCD1, KIAA1735	11	683	Cross-linking	Interact with gamma-tubulin. Regulate excitatory neuron dendrite development and synapse function in the cortex.	Autism spectrum disorders (ASDs), acute myeloid leukemia, cerebral ischemia/reperfusion injury, glioma, prostate cancer, gastric cancer, non-small-cell lung cancer, hepatocellular carcinoma, oral squamous cell carcinoma, colon cancer, retinoblastoma, pancreatic ductal adenocarcinoma, bladder cancer	127-300	https://www.uniprot.org/uniprot/Q155Q3	https://www.proteinatlas.org/ENSG00000150764-DIXDC1	2006	[538]
Dystonin, 230 kDa bullous pemphigoid antigen, 230/240 kDa bullous pemphigoid antigen, Bullous pemphigoid antigen 1 (BPA), Dystonia musculorum protein, Hemidesmosomal plaque protein	DST, BP230, BP240, BPAG1, DMH, DT, KIAA0728	6	7570	Cross-linking	Link intermediate filaments, actin and microtubule cytoskeleton networks.	Hereditary sensory and autonomic neuropathy type 6 (HSAN6), Parkinson's disease, schizophrenia, Dystonia musculorum	CH1 (35-138), CH2 (151-255)	https://www.uniprot.org/uniprot/Q03001	https://www.proteinatlas.org/ENSG00000151914-DST	1995	[539-541]
Protein FRG1, FSHD region gene 1 protein	FRG1	4	258	Bundling	Involved in muscle structure and integrity.	Prostate cancer, colorectal cancer, lung adenocarcinoma, Facioscapulohumeral muscular dystrophy (FSHD), gastric, colon and oral cavity tumor.	Fascin-like domain?	https://www.uniprot.org/uniprot/Q14331	https://www.proteinatlas.org/ENSG00000109536-FRG1	2010	[542]
Guanylate-binding protein 1, GTP-binding protein 1 (GBP-1, HuGBP-1), Guanine nucleotide-binding protein 1, Interferon-induced guanylate-binding protein 1	GBP1	1	592	Monomer binding, Capping	Hydrolyze GTP to GMP. Involvedd in anti-infection immune responses and anti-tumor activity.	Kaposi's Sarcoma-Associated Herpesvirus Virions, gastrointestinal diseases, Burkholderia thailandensis, non-small cell lung carcinoma, Ovarian Cancer, acute viral myocarditis, glioblastoma multiforme.		https://www.uniprot.org/uniprot/P32455	https://www.proteinatlas.org/ENSG00000117228-GBP1	2014	[543]
Kelch repeat and BTB domain-containing protein 13	KBTBD13	15	458		Involved in muscle relaxation. An adapter of a BCR (BTB-CUL3-RBX1) E3 ubiquitin ligase complex.	Nemaline myopathy, rod-core myopathy.		https://www.uniprot.org/uniprot/C9JR72	https://www.proteinatlas.org/ENSG00000234438-KBTBD13	2020	[544]
Fimbacin; Leucine zipper protein 1	LUZP1	1	1076	Cross-linking	Involved in actin-dependent centrosome to basal body conversion. Control cell division, migration and invasion.	Townes-Brocks Syndrome (TBS), glioma, colorectal cancer, cardiovascular malformations and cardiomyopathy,	400-500	https://www.uniprot.org/uniprot/Q86V48	https://www.proteinatlas.org/ENSG00000169641-LUZP1	2019	[545, 546]

						neural tube closure defect (NTD).					
Microtubule-associated protein 1A (MAP-1A), Proliferation-related protein p80	MAP1A, MAP1L	15	2803	Monomer binding, Bundling, Cross-linking	Link mictotubule and actin cytoskeleton. Regulate cellular morphology.	Parkinson's disease, Alzheimer's disease (AD), amyotrophic lateral sclerosis (ALS), autism spectrum disorder (ASD), attention deficit hyperactivity disorder (ADHD), bladder cancer, non-small cell lung cancer, prostate cancer, breast cancer.	C-terminal	https://www.uniprot.org/uniprot/P78559	https://www.proteinatlas.org/ENSG00000166963-MAP1A	1993	[547-549]
Microtubule-associated protein 1B (MAP-1B)	MAP1B	5	2468	Cross-linking	Induce the formation and maturation of dendritic spines by regulating actin cytoskeleton.	Chronic obstructive pulmonary disease to lung cancer, paraneoplastic neuropathies, glioblastoma, colorectal cancer, breast cancer, gastric cancer, urothelial carcinoma, renal cell carcinoma，sensorineural hearing loss, congenital microcephaly, Myelin-oligodendrocyte glycoprotein antibody-associated disease (MOGAD), Parkinson's disease (PD), Autoimmune Vestibulocerebellar Syndromes, Hirschsprung's Disease, MAP1B related syndrome, Amyotrophic lateral sclerosis (ALS), Alzheimer's disease.	C-terminal	https://www.uniprot.org/uniprot/P46821	https://www.proteinatlas.org/ENSG00000131711-MAP1B	1993	[549, 550]
Microtubule-associated protein 1S (MAP-1S), BPY2-interacting protein 1, Microtubule-associated protein 8, Variable charge Y chromosome 2-interacting protein 1 (VCY2IP-1)	MAP1S, BPY2IP1, C19orf5, MAP8, VCY2IP1	19	1059	Cross-linking	Regulate cell division and autophagy.	non-small cell lung cancer, hepatocellular carcinogenesis, clear cell renal cell carcinomas, Huntington's disease, Crohn's disease, Parkinson disease, Idiopathic pulmonary fibrosis (IPF), Autoimmune thyroid diseases (AITDs), breast cancer, colorectal cancer, prostate cancer, pancreatic ductal adenocarcinomas.	C-terminal	https://www.uniprot.org/uniprot/Q66K74	https://www.proteinatlas.org/ENSG00000130479-MAP1S	2005	[549, 551]
Microtubule-associated protein 2 (MAP-2)	MAP2	2	1827	Cross-linking	Promote neurite initiation and growth.	Huntington's Disease, oral cancer, gastric carcinoma, melanoma, breast cancer, pancreatic cancer, schizophrenia, Alzheimer's disease (AD), Hirschsprung's disease, prion disease.	C-terminal	https://www.uniprot.org/uniprot/P11137	https://www.proteinatlas.org/ENSG00000078018-MAP2	1978	[549, 552, 553]
Microtubule-associated protein 4 (MAP-4)	MAP4	3	1152	Cross-linking	Promote microtubule assembly.	Cardiac hypertrophy, fibrosis, heart failure, and hypoxic/ischemic related heart dysfunction, esophageal squamous cell carcinoma, ovarian cancer, bladder cancer, gastric cancer.	C-terminal	https://www.uniprot.org/uniprot/P27816	https://www.proteinatlas.org/ENSG00000047849-MAP4	2012	[549, 552, 554]
Microtubule-associated protein tau, Neurofibrillary tangle protein, Paired helical filament-tau (PHF-tau)	MAPT, MAPTL, MTBT1, TAU	17	758	Cross-linking	Promote morphological changes in the growth cone.	Alzheimer's disease (AD), frontotemporal dementia (FTD), Parkinson's disease (PD), prion diseases, amyotrophic lateral sclerosis (ALS), breast cancer, prostate	C-terminal	https://www.uniprot.org/uniprot/P10636	https://www.proteinatlas.org/ENSG00000186868-MAPT	1982	[549, 555-557]

						cancer, glioma, pediatric neuroblastoma, kidney clear cell carcinoma, lung carcinoinoma, pheochromocytoma/paraganglioma.					
Protein kinase C and casein kinase substrate in neurons protein 2, Syndapin-2, Syndapin-II (SdpII)	PACSIN2	22	486	Scaffolding	Regulate the morphogenesis and endocytosis of caveolae.	Diabetic kidney disease, virus infection, acute lymphoblastic leukemia.	F-BAR domain	https://www.uniprot.org/uniprot/Q9UNF0	https://www.proteinatlas.org/ENSG00000100266-PACSIN2	2014	[558, 559]
Pannexin-1	PANX1, MRS1	11	426	Anchoring, Scaffolding	Restrain human skin fibroblast motility, migration, and cell surface actin dynamics.	Renal ischemia/reperfusion injury, epilepsy, stroke, migraine with aura, chronic pain.	C-terminal	https://www.uniprot.org/uniprot/Q96RD7	https://www.proteinatlas.org/ENSG00000110218-PANX1	2010	[560, 561]
Refilin-A, Regulator of filamin protein A (RefilinA)	RFLNA, FAM101A	12	216	Bundling	Regulate lamellipodium protrusion dynamics.	Spondylocarpotarsal synostosis syndrome.		https://www.uniprot.org/uniprot/Q6ZTI6	https://www.proteinatlas.org/ENSG00000178882-RFLNA	2011	[562, 563]
Refilin-B, Regulator of filamin protein B (RefilinB)	RFLNB	17	214	Bundling	Regulate apical perinuclear Actin reorganization in early steps of epithelial mesenchymal transition (EMT).	Severe preeclampsia.		https://www.uniprot.org/uniprot/Q8N5W9	https://www.proteinatlas.org/ENSG00000183688-RFLNB	2011	[564]
SH3 and multiple ankyrin repeat domains protein 3 (Shank3), Proline-rich synapse-associated protein 2 (ProSAP2)	SHANK3, KIAA1650, PROSAP2, PSAP2	22	1731	Scaffolding	Regulate dendritic spine morphology in neurons and autism-linked phenotypes in vivo.	Parkinson disease, Phelan-McDermid syndrome, Alzheimer's disease, autism spectrum disorders (ASDs), schizophrenia (SCZ), a Rett syndrome-like phenotype, and intellectual disability (ID).	Shank/ProSAP N-terminal (SPN) domain	https://www.uniprot.org/uniprot/Q9BYB0	https://www.proteinatlas.org/ENSG00000251322-SHANK3	2021	[565]
Troponin I, slow skeletal muscle	TNNI1	1	187	Stabilization, Scaffolding	Regulate muscle contraction. Calcium-receptive protein for the Calcium -sensitive contraction in striated muscle.	Autosomal dominant proximal arthrogryposis, cardiomyopathy.	138-148	https://www.uniprot.org/uniprot/P19237	https://www.proteinatlas.org/ENSG00000159173-TNNI1	1965	[566-571]
Troponin I, fast skeletal muscle, Troponin I, fast-twitch isoform	TNNI2	11	182	?	Regulate muscle contraction. Calcium-receptive protein for the Calcium -sensitive contraction in striated muscle.	Arthrogryposis, distal, 2B1 (DA2B1).		https://www.uniprot.org/uniprot/P48788	https://www.proteinatlas.org/ENSG00000130598-TNNI2	1971	[572-576]
Troponin I, cardiac muscle, Cardiac troponin I	TNNI3, TNNC1	19	210	?	Regulate muscle contraction. Calcium-receptive protein for the Calcium -sensitive contraction in striated muscle.	Cardiomyopathy, familial hypertrophic 7 (CMH7), Cardiomyopathy, familial restrictive 1 (RCM1), Cardiomyopathy, dilated 2A (CMD2A), Cardiomyopathy, dilated 1FF (CMD1FF).		https://www.uniprot.org/uniprot/P19429	https://www.proteinatlas.org/ENSG00000129991-TNNI3	1971	[572-575, 577]
Troponin T, slow skeletal muscle, TnTs, Slow skeletal muscle troponin T, TnT.	TNNT1, TNT	19	278	?	Regulate muscle contraction. Calcium-receptive protein for the Calcium -sensitive contraction in striated muscle.	Nemaline myopathy 5 (NEM5).		https://www.uniprot.org/uniprot/P13805	https://www.proteinatlas.org/ENSG00000105048-TNNT1	1971	[572-575, 578, 579]
Troponin T, cardiac muscle (TnTc), Cardiac muscle troponin T (cTnT)	TNNT2	1	298	Stabilization, Scaffolding	Regulate muscle contraction. Calcium-receptive protein for the Calcium -sensitive contraction in striated muscle.	Dilated cardiomyopathy, hypertrophic cardiomyopathy, colorectal cancer, myotonic dystrophy type I, feline cardiomyopathy, myocardial diastolic dysfunction, sporadic hypertrophic cardiomyopathy.	C-terminal	https://www.uniprot.org/uniprot/P45379	https://www.proteinatlas.org/ENSG00000118194-TNNT2	1968	[568, 570, 571, 578, 580, 581]
Troponin T, fast skeletal muscle, TnTf, beta-TnTF, Fast skeletal muscle troponin T, fTnT.	TNNT3	11	269	?	Regulate muscle contraction. Calcium-receptive protein for the Calcium -sensitive contraction in striated muscle.	Arthrogryposis, distal, 2B2 (DA2B2).		https://www.uniprot.org/uniprot/P45378	https://www.proteinatlas.org/ENSG00000130595-TNNT3	1971	[572-575, 578, 582]
Troponin C, slow skeletal and cardiac muscles, TN-C.	TNNC1, TNNC	3	161	?	Regulate muscle contraction. Calcium-receptive protein for the Calcium -sensitive contraction in striated muscle.	Cardiomyopathy, dilated 1Z (CMD1Z), Cardiomyopathy, familial hypertrophic 13 (CMH13).		https://www.uniprot.org/uniprot/P63316	https://www.proteinatlas.org/ENSG00000114854-TNNC1	1971	[572-575, 583]

Troponin C, skeletal muscle	TNNC2	20	160	?	Regulate muscle contraction. Calcium-receptive protein for the Calcium -sensitive contraction in striated muscle.	Distal arthrogryposis (DA).		https://www.uniprot.org/uniprot/P02585	https://www.proteinatlas.org/ENSG00000101470-TNNC2	1971	[572-576, 584]
Tensin-1	TNS1, TNS	2	1735	Cross-linking, Capping	Maintain cellular structure and signal transduction and anchor actin filaments at the focal adhesion.	Cystic kidney diseases, colorectal cancer, acute myeloid leukemia, oral cleft, prostate cancer, kidney cancer, mitral valve prolapse (MVP), chronic obstructive lung disease (COPD), asthma with hay fever phenotype.	ABD Ia, ABD Ib, ABD II	https://www.uniprot.org/uniprot/Q9HBL0	https://www.proteinatlas.org/ENSG00000079308-TNS1	1986	[585-589]
Tensin-2, C1 domain-containing phosphatase and tensin homolog (C1-TEN), Tensin-like C1 domain-containing phosphatase	TNS2, KIAA1075, TENC1	12	1409	Anchoring	Permit Rho-mediated actomyosin contraction and remodeling of collagen fibers.	Pancreatic cancer, kidney cancer, glomerular disease, gastric cancer, sensitive nephrotic syndrome.	ABD Ia, ABD Ib, ABD II	https://www.uniprot.org/uniprot/Q63HR2	https://www.proteinatlas.org/ENSG00000111077-TNS2	2002	[355, 587, 589, 590]
Tensin-3, Tensin-like SH2 domain-containing protein 1, Tumor endothelial marker 6	TNS3, TEM6, TENS1, TPP	7	1445	Anchoring	Reorganize actin fiber and regulate cell migration.	Pancreatic cancer, melanoma, breast cancer, gastric cancer, colorectal cancer, hepatocellular carcinoma, lung adenocarcinoma, kidney cancer.	ABD Ia, ABD Ib, ABD II	https://www.uniprot.org/uniprot/Q68CZ2	https://www.proteinatlas.org/ENSG00000136205-TNS3	2004	[587, 589, 591]
Titin, Connectin, Rhabdomyosarcoma antigen MU-RMS-40.14	TTN	2	34350	Cross-linking	Contribute to muscle compliance, contraction, structural stability, and signaling.	Titinopathies, heart failure, ischemic and non-ischemic cardiomyopathy, dilated cardiomyopathy, tibialis muscular dystrophy, hypertrophic cardiomyopathy, centronuclear myopathy, Hereditary myopathies, Acquired titin diseases, Chronic Obstructive Pulmonay Disease (COPD), disuse atrophy.	N2A domain	https://www.uniprot.org/uniprot/Q8WZ42	https://www.proteinatlas.org/ENSG00000155657-TTN	1976	[592-596]
Dihydropyrimidinase-related protein 1, GRP-1, Collapsin response mediator protein 1, CRMP-1, Inactive dihydropyrimidinase, Unc-33-like phosphoprotein 3, ULIP-3.	CRMP1, DPYSL1, ULIP3	4	572	Scaffolding	Regulate remodeling of the cytoskeleton by dissociating FLNA from F-actin during the axon guidance process.	Schizophrenia, Epilepsy.		https://www.uniprot.org/uniprot/Q14194	https://www.proteinatlas.org/ENSG00000072832-CRMP1	2017	[597]
Dihydropyrimidinase-related protein 2 (DRP-2), Collapsin response mediator protein 2 (CRMP-2), N2A3, Unc-33-like phosphoprotein 2 (ULIP-2)	DPYSL2, CRMP2, ULIP2	8	572	Scaffolding	Promote microtubule assembly and Numb-mediated endocytosis	Alzheimer's disease (AD), Neurodegenerative disease, amyotrophic lateral sclerosis (ALS), Huntington's Disease, neuropathic pain, and Batten disease, Parkinson’s disease, lung cancer.		https://www.uniprot.org/uniprot/Q16555	https://www.proteinatlas.org/ENSG00000092964-DPYSL2	2005	[598]
Dihydropyrimidinase-related protein 3 (DRP-3), Collapsin response mediator protein 4 (CRMP-4), Unc-33-like phosphoprotein 1 (ULIP-1)	DPYSL3, CRMP4, DRP-3, ULIP, ULIP1	5	570	Bundling	Inhibit the cell migration and maintain rib-like actin-structures in lamellipodia. Promote dendritic growth and maturation.	Amyotrophic lateral sclerosis (ALS), Autism spectrum disorders (ASD), Parkinson's disease, Alzheimer's disease	C-terminal	https://www.uniprot.org/uniprot/Q14195	https://www.proteinatlas.org/ENSG00000113657-DPYSL3	2005	[599, 600]
Dihydropyrimidinase-related protein 5 (DRP-5), CRMP3-associated molecule (CRAM), Collapsin	DPYSL5, CRMP5, ULIP6	2	564	Scaffolding	Regulate growth cone development and neurite outgrowth.	Paraneoplastic neurological syndromes (PNS), Alzheimer's disease, small-cell lung cancer, thymoma.	C-terminal	https://www.uniprot.org/uniprot/Q9BPU6	https://www.proteinatlas.org/ENSG00000157851-DPYSL5	2016	[601]

response mediator protein 5 (CRMP-5), UNC33-like phosphoprotein 6 (ULIP-6)											
Cysteine and glycine-rich protein 2, Cysteine-rich protein 2 (CRP2), LIM domain only protein 5 (LMO-5), Smooth muscle cell LIM protein (SmLIM)	CSRP2, LMO5, SMLIM	12	193	Bunding	Contribute to the assembly and/or maintenance of the invadopodium actin backbone.	Colorectal cancer, acute lymphoblastic leukemia, hepatocellular carcinoma.		https://www.uniprot.org/uniprot/Q16527	https://www.proteinatlas.org/ENSG00000175183-CSRP2	2016	[602]
Epidermal growth factor receptor, Proto-oncogene c-ErbB-1, Receptor tyrosine-protein kinase erbB-1	EGFR, ERBB, ERBB1, HER1	7	1210	Anchoring	Regulate the internalization of the EGF/EGFR complex to the lysosomes.	Non-small cell lung cancer, glioblastoma, pulmonary fibrosis, chronic obstructive pulmonary disease (COPD), gastric cancer, hypertrophy and lysosomal disease, cardiovascular disease, head and neck cancer, colorectal cancer, epithelial ovarian cancer.	984-996	https://www.uniprot.org/uniprot/P00533	https://www.proteinatlas.org/ENSG00000146648-EGFR	1992	[603] [604]
ABI gene family member 3, New molecule including SH3, Nesh	ABI3, NESH	17	366	Stabilization	Regulate dendritic spine morphogenesis and synapse formation.	Alzheimer's disease and Nasu-Hakola disease.	N-terminal	https://www.uniprot.org/uniprot/Q9P2A4	https://www.proteinatlas.org/ENSG00000108798-ABI3	2012	[605, 606]
Neuroblast differentiation-associated protein AHNAK, Desmoyokin	AHNAK, PM227	11	5890	Bundling	Stabilize muscle contractility.	Pulmonary tumorigenesis	C-terminal	https://www.uniprot.org/uniprot/Q09666	https://www.proteinatlas.org/ENSG00000124942-AHNAK	2004	[607]
LIM domain-containing protein ajuba	AJUBA, JUB	14	538	Scaffolding	Scaffold many protein to regulate several cellular processes such migration and proliferation.	Hepatocellular carcinoma		https://www.uniprot.org/uniprot/Q96IF1	https://www.proteinatlas.org/ENSG00000129474-AJUBA	2003	[608-610]
Rho GTPase-activating protein 12, Rho-type GTPase-activating protein 12	ARHGAP12	10	846	Monomer binding	Regulate invadopodia formation and metastasis. ARHGAP12 and 32 subfamily interact with G-actin.	Carcinoma	RPEL-like motif	https://www.uniprot.org/uniprot/Q8IWW6	https://www.proteinatlas.org/ENSG00000165322-ARHGAP12	2019	[611]
Rho guanine nucleotide exchange factor 17, 164 kDa Rho-specific guanine-nucleotide exchange factor, p164-RhoGEF, Tumor endothelial marker 4	ARHGEF17, KIAA0337, TEM4	11	2063		Act as guanine nucleotide exchange factor for RhoA GTPases.	Intracranial Aneurysms	81-135	https://www.uniprot.org/uniprot/Q96PE2	https://www.proteinatlas.org/ENSG00000110237-ARHGEF17	2012	[612]
Apoptosis regulator Bcl-2	BCL2	18	239	Polymerization, Scaffolding	Inhibit cell adhesion, spreading, and motility. Suppresses apoptosis.	Chronic lymphatic leukemia.		https://www.uniprot.org/uniprot/P10415	https://www.proteinatlas.org/ENSG00000171791-BCL2	2010	[613]
Calcium/calmodulin-dependent protein kinase type II subunit beta, CaM kinase II subunit beta, CaMK-II subunit beta	CAMK2B, CAM2, CAMK2, CAMKB	7	666	Stabilization	Calcium/calmodulin-dependent protein kinase that regulate dendritic remodeling.	Mental retardation, autosomal dominant 54 (MRD54).	actin-binding/-stabilizing domain	https://www.uniprot.org/uniprot/Q13554	https://www.proteinatlas.org/ENSG00000058404-CAMK2B	1998	[614, 615]
T-complex protein 1 subunit epsilon, TCP-1-epsilon, CCT-epsilon T-complex protein 1 subunit theta, TCP-1-theta, CCT-theta, Renal carcinoma antigen NY-REN-15	CCT5, CCTE, KIAA0098 CCT8, C21orf112, CCTQ, KIAA0002	5<br>21	541<br>548	Folding of actin	Molecular chaperone complex involved in folding of actin and tubulin.	Neuropathy.		https://www.uniprot.org/uniprot/P48643<br>https://www.uniprot.org/uniprot/P50990	https://www.proteinatlas.org/ENSG00000150753-CCT5	2006	[616]
Prefoldin (Prefoldin subunit 1,	PFDN1, PFD1	5<br>1<br>X	122<br>154<br>197	Folding of actin	Target premature nascent actin to	Alzheimer's disease, Parkinson's	1-145	https://www.uniprot.org/uniprot/O60925	https://www.proteinatlas.org/ENSG0	1999	[617, 618]

Prefoldin subunit 2, Prefoldin subunit 3, Prefoldin subunit 4, Prefoldin subunit 5, Prefoldin subunit 6)	PFDN2, PFD2 PFDN3, VBP1 PFDN4, PFD4 PFDN5, PFD5 PFDN6, PFD6, HKE2	20<br>12<br>6	134<br>154<br>129		cytosolic chaperonin.	disease, and Huntington's disease.		https://www.uniprot.org/uniprot/Q9UHV9<br>https://www.uniprot.org/uniprot/P61758<br>https://www.uniprot.org/uniprot/Q9NQP4<br>https://www.uniprot.org/uniprot/Q99471<br>https://www.uniprot.org/uniprot/O15212	0000113068-PFDN1<br>https://www.proteinatlas.org/ENSG00000143256-PFDN2<br>https://www.proteinatlas.org/ENSG00000155959-VBP1<br>https://www.proteinatlas.org/ENSG00000101132-PFDN4<br>https://www.proteinatlas.org/ENSG00000123349-PFDN5<br>https://www.proteinatlas.org/ENSG00000204220-PFDN6		
Carcinoembryonic antigen-related cell adhesion molecule 1, Biliary glycoprotein 1, BGP-1, CD66a	CEACAM1, GP, BGP1	19	526	Anchoring, Scaffolding	Mediate homophilic cell adhesion.	Inflammatory bowel disease.	Cytoplasmic domain (453-526)	https://www.uniprot.org/uniprot/P13688	https://www.proteinatlas.org/ENSG00000079385-CEACAM1	2011	[619]
Cingulin	CGN, KIAA1319	1	1197	Anchoring	Regulate formation and regulation of the tight junction.	Disease related to tight junction: inflammatory bowel disease.	101-294	https://www.uniprot.org/uniprot/Q9P2M7	https://www.proteinatlas.org/ENSG00000143375-CGN	2001	[620]
Alpha-crystallin B chain, Heat shock protein beta-5, HspB5, Renal carcinoma antigen NY-REN-27, Rosenthal fiber component	CRYAB, CRYA2, HSPB5	11	175	Scaffolding	Regulate the cell cycle, and reducing caspase-mediated apoptosis.	Myopathy, myofibrillar, 2 (MFM2), Cataract 16, multiple types (CTRCT16), Myopathy, myofibrillar, fatal infantile hypertonic, alpha-B crystallin-related (MFMFIH-CRYAB), Cardiomyopathy, dilated 1II (CMD1II).	ABD	https://www.uniprot.org/uniprot/P02511	https://www.proteinatlas.org/ENSG00000109846-CRYAB	1992	[621-624]
DENN domain-containing protein 1C, Connecdenn 3, FAM31C	DENND1C, FAM31C	19	801		Act as guanine nucleotide exchange factor to activate RAB.		C-terminal motif (786-801)	https://www.uniprot.org/uniprot/Q8IV53	https://www.proteinatlas.org/ENSG00000205744-DENND1C	2012	[625]
ATP-dependent RNA helicase A, DEAH box protein 9, DExH-box helicase 9, Leukophysin (LKP), Nuclear DNA helicase II (NDH II), RNA helicase A	DHX9, DDX9, LKP, NDH2	1	1270		ATP-dependent nucleic acid helicase that regulates transcriptional activation.	Systemic lupus erythematosus (SLE) , Ewing's sarcoma family tumours (ESFTs)	C-terminal	https://www.uniprot.org/uniprot/Q08211	https://www.proteinatlas.org/ENSG00000135829-DHX9	2002	[626-628]
H1 Histone				Polymerization						1978	[629]
H2A-H2B histone dimer				Bundling						2011	[630, 631]
Deoxyribonuclease-1, Deoxyribonuclease I (DNase I), Dornase alfa	DNASE1, DNL1, DRNI	16	282	Depolymerization	Bind specifically to G-actin and blocks actin polymerization.	Systemic lupus erythematosus (SLE), Autoimmune diseases (AID).	PDB: 1ATN, 3W3D	https://www.uniprot.org/uniprot/P24855	https://www.proteinatlas.org/ENSG00000213918-DNASE1	1974	[631-633]
Dynamin-1	DNM1, DNM	9	864	Bundling	Involved in vesicular trafficking processes.	Developmental and epileptic encephalopathy 31 (DEE31)	399 and 444	https://www.uniprot.org/uniprot/Q05193	https://www.proteinatlas.org/ENSG00000106976-DNM1	2010	[634]
Dynamin-2	DNM2, DYN2	19	870	Bundling	Organize lamellipodial actin networks to orchestrate lamellar actomyosin. Involved in vesicular trafficking processes.	Myopathy, centronuclear, 1 (CNM1).		https://www.uniprot.org/uniprot/P50570	https://www.proteinatlas.org/ENSG00000079805-DNM2	2010	[634-636]
Elongation factor 1-alpha 1, EF-1-alpha-1, Elongation factor Tu, Eukaryotic elongation factor 1 A-1, Leukocyte receptor cluster member 7	EEF1A1, EEF1A, EF1A, LENG7	6	462	Bundling, Scaffolding	Regulate IFNG transcription in T-helper 1 cells.			https://www.uniprot.org/uniprot/P68104	https://www.proteinatlas.org/ENSG00000156508-EEF1A1	2002	[630, 637-640]
Elongation factor 2,	EEF2, EF2	19	858		Catalyze the GTP-dependent ribosomal translocation step during translation elongation.	Spinocerebellar ataxia 26 (SCA26).		https://www.uniprot.org/uniprot/P13639	https://www.proteinatlas.org/ENSG00000167658-EEF2	1994	[641, 642]
Cytoskeleton-associated protein 5,	CKAP5, KIAA0097(Xenopus	11	2032	Scaffolding	Regulate microtubule dynamics and		Demonstrated with Xenopus la	https://www.uniprot.org/uniprot/Q14008	https://www.proteinatlas.org/ENSG0	2019	[643]

Colonic and hepatic tumor overexpressed gene protein, Ch-TOG	laevis: XMAP215)				microtubule organization. TOG/XMAP215 family.		evis XMAP215.		0000175216-CKAP5		
Emerin	EMD, ED MD, STA	X	254	Capping	Stimulate actin polymerization in vitro by binding and stabilizing the pointed end.	Emery-Dreifuss muscular dystrophy 1, X-linked (EDMD1)		https://www.uniprot.org/uniprot/P50402	https://www.proteinatlas.org/ENSG00000102119-EMD	2004	[644]
F-box only protein 25	FBXO25, FBX25	8	367	Scaffolding	Interact with nuclear actin. Involved in the pathway protein ubiquitination.	X-linked mental retardation (XLMR).	N-terminal	https://www.uniprot.org/uniprot/Q8TCJ0	https://www.proteinatlas.org/ENSG00000147364-FBXO25	2010	[645]
FYVE, RhoGEF and PH domain-containing protein 4, Actin filament-binding protein frabin, FGD1-related F-actin-binding protein, Zinc finger FYVE domain-containing protein 6	FGD4, FRABP, ZFYVE6	12	766	Cross-linking	Capable of changing cell shape and activating c-Jun N-terminal kinase.	Charcot-Marie-Tooth disease 4H (CMT4H).	N-terminal	https://www.uniprot.org/uniprot/Q96M96	https://www.proteinatlas.org/ENSG00000139132-FGD4	1998	[646]
Flotillin-2, Epidermal surface antigen, ESA, Membrane component chromosome 17 surface marker 1	FLOT2, ESA1, M17S1	17	428	Scaffolding	Involved in formation of caveolae or caveolae-like vesicles.	Carcinoma.	SPFH domain (1-184)	https://www.uniprot.org/uniprot/Q14254	https://www.proteinatlas.org/ENSG00000132589-FLOT2	2007	[647]
Vitamin D-binding protein (DBP, VDB), Gc protein-derived macrophage activating factor (Gc-MAF, GcMAF), Gc-globulin, Group-specific component (Gc), Vitamin D-binding protein-macrophage activating factor (DBP-maf)	GC	4	474	Monomer binding, Depolymerization	Enhance clearance of fibrillar actin.	Osteoporosis, type 1 and type 2 diabetes, thyroid autoimmunity, inflammatory bowel disease, and chronic obstructive lung disease.	B and C domains	https://www.uniprot.org/uniprot/P02774	https://www.proteinatlas.org/ENSG00000145321-GC	1980	[648, 649]
Heat shock protein beta-7, HspB7, Cardiovascular heat shock protein, cvHsp	HSPB7, CVHSP	1	170	Monomer binding	HSPB7 binds G actin and inhibits actin polymerization to regulate actin thin filament length in cardiac muscle.	Cardiocmyopathy.		https://www.uniprot.org/uniprot/Q9UBY9	https://www.proteinatlas.org/ENSG00000173641-HSPB7	2017	[650]
Protein IMPACT, Imprinted and ancient gene protein homolog	IMPACT	18	320	Monomer binding	Involved in neuronal development, immune system regulation and the cell cycle.			https://www.uniprot.org/uniprot/Q9P2X3	https://www.proteinatlas.org/ENSG00000154059-IMPACT	2011	[651, 652]
Inner centromere protein,	INCENP	11	918	Scaffolding	Regulate cytokinesis and midzone MT stabilization following furrow ingression.	Graham Little-Piccardi-Lassueur syndrome, Breast cancer.		https://www.uniprot.org/uniprot/Q9NQS7	https://www.proteinatlas.org/ENSG00000149503-INCENP	2016	[653]
Chromatin-remodeling ATPase INO80,	INO80, INO80A, INOC1, KIAA1259	15	1556	Monomer binding (Nuclear actin)	Involved in transcriptional regulation, DNA replication and DNA repair.	Melanoma, non-small cell lung cancer, congenital heart disease.	N-terminal helical helicase-SANT-associated (HSA) domain. Demonstrated with Saccharomyces cerevisiae INO80.	https://www.uniprot.org/uniprot/Q9ULG1	https://www.proteinatlas.org/ENSG00000128908-INO80	2018	[654, 655]
Fermitin family homolog 2, Kindlin-2, Mitogen-inducible gene 2 protein (MIG-2), Pleckstrin homology domain-containing family C member 1 (PH domain-containing family C member 1)	FERMT2, KIND2, MIG2, PLEKHC1	14	680	Scaffolding	Regulate integrin outside-in signaling.	Late-onset Alzheimer's disease	F0 domain (1-105)	https://www.uniprot.org/uniprot/Q96AC1	https://www.proteinatlas.org/ENSG00000073712-FERMT2	2016	[656]

Inositol-trisphosphate 3-kinase A, Inositol 1,4,5-trisphosphate 3-kinase A, IP3 3-kinase A	ITPKA	15	461	Bundling	Organize actin networks.	Lung adenocarcinoma.	N-terminal	https://www.uniprot.org/uniprot/P23677	https://www.proteinatlas.org/ENSG00000137825-ITPKA	2009	[657, 658]
Calcium-activated potassium channel subunit alpha-1, BK channel, BKCA alpha, Calcium-activated potassium channel, subfamily M subunit alpha-1, K(VCA)alpha, KCa1.1, Maxi K channel (MaxiK), Slo-alpha, Slo1, Slowpoke homolog (Slo homolog, hSlo)	KCNMA1, KCNMA, SLO	10	1236	Anchoring	Regulate contraction of smooth muscle, tuning of hair cells in the cochlea, transmitter release, and innate immunity.	Paroxysmal nonkinesigenic dyskinesia, 3, with or without generalized epilepsy (PNKD3), Epilepsy, idiopathic generalized 16 (EIG16), Cerebellar atrophy, developmental delay, and seizures (CADEDS), Liang-Wang syndrome (LIWAS).	C-terminal (985-1108)	https://www.uniprot.org/uniprot/Q12791	https://www.proteinatlas.org/ENSG00000156113-KCNMA1	2008	[659]
Amiloride-sensitive sodium channel subunit alpha, Alpha-NaCH, Epithelial Na(+) channel subunit alpha (Alpha-ENaC, ENaCA), Nonvoltage-gated sodium channel 1 subunit alpha, SCNEA	SCNN1A, SCNN1	12	669	Anchoring	Regulate electrolyte and blood pressure homeostasis.	Pseudohypoaldosteronism 1, autosomal recessive (PHA1B), Bronchiectasis with or without elevated sweat chloride 2 (BESC2), Liddle syndrome 3 (LIDLS3).	C-terminal	https://www.uniprot.org/uniprot/P37088	https://www.proteinatlas.org/ENSG00000111319-SCNN1A	2006	[389]
Kinesin-like protein KIF18A, Marrow stromal KIF18A (MS-KIF18A)	KIF18A	11	898		Regulate chromosome movements.	Hepatocellular carcinoma (HCC), lung adenocarcinoma.		https://www.uniprot.org/uniprot/Q8NI77	https://www.proteinatlas.org/ENSG00000121621-KIF18A	2008	[660]
Serine/threonine-protein kinase LATS1, Large tumor suppressor homolog 1, WARTS protein kinase (h-warts)	LATS1, WARTS	6	1130	Scaffolding	Negatively regulate YAP1 in the Hippo signaling pathway. Modulate actin polymerization, cell migration and cell spreading.	Glicoma, many other cancers.		https://www.uniprot.org/uniprot/O95835	https://www.proteinatlas.org/ENSG00000131023-LATS1	2011	[661]
E3 ubiquitin-protein ligase MIB2, Mind bomb homolog 2, Novel zinc finger protein (Novelzin), Putative NF-kappa-B-activating protein 002N, RING-type E3 ubiquitin transferase MIB2, Skeletrophin, Zinc finger ZZ type with ankyrin repeat domain protein 1	MIB2, SKD, ZZANK1	1	1013	Monomer binding	Mediate ubiquitination of Delta receptors to regulate the Delta-mediated Notch signaling.	Ménétrier-like gastropathy.		https://www.uniprot.org/uniprot/Q96AX9	https://www.proteinatlas.org/ENSG00000197530-MIB2	2003	[662]
Myosin-binding protein C, cardiac-type, MyBP-C, C-protein, cardiac muscle isoform	MYBPC3	11	1274		Regulate muscle contraction.	Cardiomyopathy, familial hypertrophic 4 (CMH4), Cardiomyopathy, dilated 1MM (CMD1MM), Left ventricular non-compaction 10 (LVNC10).	N-terminal	https://www.uniprot.org/uniprot/Q14896	https://www.proteinatlas.org/ENSG00000134571-MYBPC3	1978	[663, 664]
Pleckstrin homology domain-containing family G member 2, PH domain-containing family G member 2, FLJ00018	PLEKHG2	19	1386	Polymerization	Regulate cell morphogenesis.	Leukodystrophy and acquired microcephaly with or without dystonia (LDAMD).	150-283, 465-1386	https://www.uniprot.org/uniprot/Q9H7P9	https://www.proteinatlas.org/ENSG00000090924-PLEKHG2	2013	[665]
Ras-related protein Rap-2a, RbBP-30	RAP2A	13	183	Scaffolding	Regulate cell migration and invasion.	Prostate cancer, breast cancer, colorectal cancer, bladder cancer, renal cell carcinoma, hepatocellular carcinoma,		https://www.uniprot.org/uniprot/P10114	https://www.proteinatlas.org/ENSG00000125249-RAP2A	1999	[666]

						nasopharyngeal carcinoma.					
AP-4 complex accessory subunit RUSC1, New molecule containing SH3 at the carboxy-terminus (Nesca), RUN and SH3 domain-containing protein 1	RUSC1, NESCA	1	902	Scaffolding	Function as an adapter involved in neuronal vesicular transport.		231-316	https://www.uniprot.org/uniprot/Q9BVN2	https://www.proteinatlas.org/ENSG00000160753-RUSC1	2012	[667]
NCK-interacting protein with SH3 domain, 54 kDa VacA-interacting protein, 54 kDa vimentin-interacting protein (VIP54), 90 kDa SH3 protein interacting with Nck, AF3p21, Dia-interacting protein 1 (DIP-1), Diaphanous protein-interacting protein, SH3 adapter protein SPIN90, WASP-interacting SH3-domain protein (WISH), Wiskott-Aldrich syndrome protein-interacting protein	NCKIPSD, AF3P21, SPIN90	3	722	Polymerization, Scaffolding	Involved in formation of branched actin networks and regulation of the actin cytoskeleton at the leading edge of cells.	Breast cancer, chromosomal aberration involving NCKIPSD/AF3p21.	582-722	https://www.uniprot.org/uniprot/Q9NZQ3	https://www.proteinatlas.org/ENSG00000213672-NCKIPSD	2007	[668]
Alpha-1-syntrophin, 59 kDa dystrophin-associated protein A1 acidic component 1, Pro-TGF-alpha cytoplasmic domain-interacting protein 1 (TACIP1), Syntrophin-1	SNTA1, SNT1	20	505	Scaffolding, Anchoring	Regulate intracellular localization and activity of various actin organizing signaling molecules.	Parkinson's disease (PD), long QT syndrome, breast cancer, neuromuscular junctions (NMJ), Duchenne muscular dystrophy (DMD), fukuyama muscular dystrophies (FMD), multisystemic disorder myotonic dystrophy type 1 (DM1), sudden infant death syndrome (SIDS).	274-315, 449-505	https://www.uniprot.org/uniprot/Q13424	https://www.proteinatlas.org/ENSG00000101400-SNTA1	2004	[669-671]
Beta-1-syntrophin, 59 kDa dystrophin-associated protein A1 basic component 1 (DAPA1B, BSYN2), Syntrophin-2, Tax interaction protein 43 (TIP-43)	SNTB1, SNT2B1	8	538	Scaffolding, Anchoring	Link various receptors to the actin cytoskeleton. Involved in synapse formation.	Duchenne muscular dystrophy (DMD)	PDZ domain	https://www.uniprot.org/uniprot/Q13884	https://www.proteinatlas.org/search/SNTB1	2004	[669, 670]
Beta-2-syntrophin, 59 kDa dystrophin-associated protein A1 basic component 2, Syntrophin-3 (SNT3), Syntrophin-like (SNTL)	SNTB2, D16S2531E, SNT2B2, SNTL	16	540	Scaffolding, Anchoring	Link various receptors to the actin cytoskeleton.	Neuromuscular junctions (NMJ), type-2 diabetes mellitus or non-insulin-dependent diabetes mellitus (NIDDM),	PDZ domain	https://www.uniprot.org/uniprot/Q13425	https://www.proteinatlas.org/ENSG00000168807-SNTB2	2004	[669, 670, 672]
Stomatin-like protein 2, mitochondrial (SLP-2), EPB72-like protein 2, Paraprotein target 7 (Paratarg-7)	STOML2, SLP2	9	356	Scaffolding, Anchoring	Involved in T cell activation by ensuring sustained TCR signaling.	Hepatocellular carcinoma, colorectal cancer, cervical cancer, head and neck squamous cell carcinoma, gastric adenocarcinoma, epithelial ovarian cancer, esophageal carcinoma, gallbladder cancer, non-small cell lung cancer, pancreatic cancer.		https://www.uniprot.org/uniprot/Q9UJZ1	https://www.proteinatlas.org/ENSG00000165283-STOML2	2008	[673]

Testin, TESS	TES	7	421	Scaffoldin	Involve in the cell adhesion, cell spreading and in the reorganization at the actin cytoskeleton.	Ovarian cancer, breast cancer, endometrial cancer, colorectal cancer, gastric cancer, lung cancer, prostate cancer, head and neck squamous cell cancer.	PET domain	https://www.uniprot.org/uniprot/Q9UGI8	https://www.proteinatlas.org/ENSG00000135269-TES	2003	[674, 675]
Tubby-related protein 1, Tubby-like protein 1	TULP1, TUBL1	6	542	Anchoring	Involve in protein trafficking through the connecting cilium into the outer segment of photoreceptor cells.	Retinal and cochlear degeneration, retinitis pigmentosa (RP), adult-onset obesity associated with insulin resistance.		https://www.uniprot.org/uniprot/O00294	https://www.proteinatlas.org/ENSG00000112041-TULP1	2005	[676]
Kelch-like protein 23	KLHL23	2	558	Bundling	Suppress F-actin, filopodium and lamellipodium formation.	Bladder urothelial carcinoma, hepatocellular carcinoma (HCC), pancreatic cancer, urothelial carcinoma.		https://www.uniprot.org/uniprot/Q8NBE8	https://www.proteinatlas.org/ENSG00000213160-KLHL23	2018	[677, 678]
Kelch-like ECH-associated protein 1, Cytosolic inhibitor of Nrf2 (INrf2), Kelch-like protein 19	KEAP1, INRF2, KIAA0132, KLHL19	19	624		Stabilize F-actin cytoskeleton structures and inhibit focal adhesion turnover.	Lung adenocarcinoma, large cell carcinoma, prostate carcinoma.	Double glycine repeat domain	https://www.uniprot.org/uniprot/Q14145	https://www.proteinatlas.org/ENSG00000079999-KEAP1	1999	[679-682]
Ectoderm-neural cortex protein 1(ENC-1), Kelch-like protein 37, Nuclear matrix protein NRP/B, p53-induced gene 10 protein	ENC1, KLHL37, NRPB, PIG10	5	589		Require for adipocyte differentiation.	Hairy cell leukemia (HCL), urothelial carcinoma.		https://www.uniprot.org/uniprot/O14682	https://www.proteinatlas.org/ENSG00000171617-ENC1	1997	[683, 684]
Protein S100-A4, Calvasculin, Metastasin, Placental calcium-binding protein, Protein Mts1, S100 calcium-binding protein A4	S100A4, CAPL, MTS1	1	101	Bundling, Scaffolding	Change in cell morphology, adhesion and migration.	Kidney fibrosis, liver fibrosis, pulmonary fibrosis and artery diseases, cardiac hypertrophy and fibrosis and rheumatoid arthritis, obesity, metastatic colorectal cancer, prostate cancer, brain tumors, breast, colon and lung carcinomas, Alzheimer's (AD) and Parkinson's diseases (PD), in cerebral ischemia, epilepsy, and schizophrenia, nervous system acute injuries.		https://www.uniprot.org/uniprot/P26447	https://www.proteinatlas.org/ENSG00000196154-S100A4	1993	[685]
Protein S100-A6, Calcyclin, Growth factor-inducible protein 2A9, MLN 4, Prolactin receptor-associated protein, PRA, S100 calcium-binding protein A6	S100A6, CACY	1	90	Monomer binding, F-actin binding	Regulate the cell cycle and morphology.	Pancreatic, gastric and prostate cancer, melanoma, non-small cell lung carcinoma, hepatocellular carcinoma, amyotrophic lateral sclerosis (ALS), Alzheimer's disease, urinary bladder urothelial carcinoma, ovarian cancer, acute coronary syndrome and myocardial infarction.		https://www.uniprot.org/uniprot/P06703	https://www.proteinatlas.org/ENSG00000197956-S100A6	2020	[686]
Tripartite motif-containing protein 3, Brain-expressed RING finger protein, RING finger protein 22, RING finger protein 97	TRIM3, BERP, RNF22, RNF97	11	744		Polyubiquitylates γ-actin to regulate synaptic γ-actin turnover and actin filament stability and thus form a transient inhibitory constraint on the expression of hippocampal synaptic plasticity.	Parkinson's disease, esophageal squamous cell carcinoma.		https://www.uniprot.org/uniprot/O75382	https://www.proteinatlas.org/ENSG00000110171-TRIM3	2015	[687]
Septin-9, MLL septin-like fusion protein MSF-A (MLL septin-like fusion protein), Ovarian/Breast septin (Ov/Br septin), Septin D1	SEPTIN9, KIAA0991, MSF, SEPT9	17	586	Cross-linking	Maintain the integrity of growing and contracting actin filaments.	Colorectal cancer, breast cancer, hematological tumors, head and neck squamous cell carcinoma, ovarian cancer, lung cancer, gastric cancer.	Basic domain (B-domain)	https://www.uniprot.org/uniprot/Q9UHD8	https://www.proteinatlas.org/ENSG00000184640-SEPTIN9	2014	[688-690]
Uveal autoantigen with coiled-coil domains and ankyrin	UACA			Capping	Cap barbed end. Act to spatially regulate the intracellular distribution of		Shown with bovine protein	Bovine: https://www.uniprot.org/uniprot/Q8HYY4 Human:	https://www.proteinatlas.org/ENSG00000137831-UACA	1996	[691, 692]

repeats protein, Beta-actin-binding protein, Beta cap73					isoactins, facilitate forward protrusion formation.			https://www.uniprot.org/uniprot/Q9BZF9			
Mitogen-activated protein kinase 3 (MAP kinase 3, MAPK3), ERT2, Extracellular signal-regulated kinase 1 (ERK-1), Insulin-stimulated MAP2 kinase, MAP kinase isoform p44 (p44-MAPK), Microtubule-associated protein 2 kinase, p44-ERK1	MAPK3, ERK1, PRKM3	16	379	Scaffolding	Provide a scaffold for ERK signaling complexes in both muscle and non-muscle cells.	Neurodevelopmental delay and autism, gastric cancer.		https://www.uniprot.org/uniprot/P27361	https://www.proteinatlas.org/ENSG00000102882-MAPK3	1997	[693, 694]
Harmonin, Antigen NY-CO-38/NY-CO-37, Autoimmune enteropathy-related antigen AIE-75, Protein PDZ-73, Renal carcinoma antigen NY-REN-3, Usher syndrome type-1C protein	USH1C, AIE75	11	552	Bundling, Scaffolding	Involve in the adaptation of mechanoelectrical transduction by sensory hair cells.	Usher syndrome 1C (USH1C), Deafness, autosomal recessive, 18A (DFNB18A).		https://www.uniprot.org/uniprot/Q9Y6N9	https://www.proteinatlas.org/ENSG00000006611-USH1C	2002	[695, 696]
Hexokinase-1, Brain form hexokinase, Hexokinase type I (HK I), Hexokinase-A	HK1	10	917	Capping	Mediate the initial step of glycolysis.	Hexokinase deficiency (HK deficiency), Neuropathy, hereditary motor and sensory, Russe type (HMSNR), Retinitis pigmentosa 79 (RP79), Neurodevelopmental disorder with visual defects and brain anomalies (NEDVIBA).		https://www.uniprot.org/uniprot/P19367	https://www.proteinatlas.org/ENSG00000156515-HK1	1999	[697]
Heat shock protein beta-1, HspB1, 28 kDa heat shock protein, Estrogen-regulated 24 kDa protein, Heat shock 27 kDa protein (HSP27), Stress-responsive protein 27 (SRP27)	HSPB1, HSP27, HSP28	7	205	Stabilization	A molecular chaperone involved in stress resistance and actin organization.	Charcot-Marie-Tooth disease 2F (CMT2F), Neuronopathy, distal hereditary motor, 2B (HMN2B).		https://www.uniprot.org/uniprot/P04792#names_and_taxonomy	https://www.proteinatlas.org/ENSG00000106211-HSPB1	2013	[698, 699]
Myelin basic protein, Myelin A1 protein, Myelin membrane encephalitogenic protein	MBP	18	304	Bundling, Polymerization	Create a cytosol to membrane signal caused by changes in interaction of the cytoskeleton with the membrane.	Rheumatoid arthritis, multiple sclerosis, demyelinating disease, brain cancer.		https://www.uniprot.org/uniprot/P02686	https://www.proteinatlas.org/ENSG00000197971-MBP	1984	[700, 701]
Neurocalcin-delta	NCALD	8	193		Control clathrin-coated vesicle traffic.	Alzheimer's disease, spinal muscular atrophy (SMA), diabetic nephropathy.		https://www.uniprot.org/uniprot/P61601	https://www.proteinatlas.org/ENSG00000104490-NCALD	2001	[702, 703]
Serine/threonine-protein kinase PAK 5, p21-activated kinase 5 (PAK-5), p21-activated kinase 7 (PAK7)	PAK5, KIAA1264, PAK7	20	719	Scaffolding	Communicate between the actin and MT networks during cellular responses to environmental conditions.	Colorectal, gastric, breast, lung, bladder, epithelial ovarian cancers, glioma, acute myeloid leukemia.		https://www.uniprot.org/uniprot/Q9P286	https://www.proteinatlas.org/ENSG00000101349-PAK5	2001	[704]
Neutrophil cytosol factor 1 (NCF-1), 47 kDa autosomal chronic granulomatous disease protein, 47 kDa neutrophil oxidase factor, NCF-47K, Neutrophil NADPH oxidase factor 1, Nox organizer 2, Nox-organizing protein 2, SH3	NCF1, NOXO2, SH3PXD1A	7	390	Scaffolding	Required for NADPH oxidase activation.	Granulomatous disease, chronic, cytochrome-b-positive 1, autosomal recessive (CGD1).		https://www.uniprot.org/uniprot/P14598	https://www.proteinatlas.org/ENSG00000158517-NCF1	2000	[705]

and PX domain-containing protein 1A, p47-phox											
Cellular tumor antigen p53, Antigen NY-CO-13, Phosphoprotein p53, Tumor suppressor p53	TP53, P53	17	393	Scaffolding	Serve to initiate cellular responses to a variety of stresses, particularly DNA damage and has the capacity to transactivate stress response genes.	Esophageal cancer (ESCR), Li-Fraumeni syndrome (LFS), Squamous cell carcinoma of the head and neck, Lung cancer, Papilloma of choroid plexus (CPP), Adrenocortical carcinoma (ADCC), Basal cell carcinoma 7 (BCC7), Bone marrow failure syndrome 5 (BMFS5).		https://www.uniprot.org/uniprot/P04637	https://www.proteinatlas.org/ENSG00000141510-TP53	1999	[706, 707]
Synapsin-1, Brain protein 4.1, Synapsin I	SYN1	X	705	Nucleation, Bundling, Anchoring	Regulate neurotransmitter release by cross-linking synaptic vesicles to the actin cytoskeleton.	Epilepsy X-linked, with variable learning disabilities and behavior disorders (XELBD).		https://www.uniprot.org/uniprot/P17600	https://www.proteinatlas.org/ENSG00000008056-SYN1	1987	[708, 709]
Synapsin-2, Synapsin II	SYN2	3	582	Nucleation, Bundling	Cross-link synaptic vesicles and actin filaments in the nerve terminal.	Schizophrenia (SCZD).		https://www.uniprot.org/uniprot/Q92777	https://www.proteinatlas.org/ENSG00000157152-SYN2	1994	[710, 711]
Proto-oncogene vav	VAV1, VAV	19	845		Correlate with their control on the G-proteins Rac and Rho and involve in the organization of cytoskeleton.	Pancreatic cancer, ell chronic lymphocytic leukemia (B-CLL), Diffuse large B-cell lymphoma (DLBCL), neuroblastoma, lung cancer, breast carcinoma, esophageal squamous cell carcinomas (ESCC), ovarian cancer, medulloblastomas.	N-terminal CH domain is responsible for localization to actin filaments in cells but direct interaction has never been demonstrated.	https://www.uniprot.org/uniprot/P15498	https://www.proteinatlas.org/ENSG00000141968-VAV1	1995	[712, 713]

*: See Figure 2.
**: As a gene or AAP in any vertebrate.

References for Table-S1

1. Albertson, D. G., Mapping muscle protein genes by in situ hybridization using biotin-labeled probes. *EMBO J* **1985,** 4, (10), 2493-8.
2. Citi, S.; Kendrick-Jones, J., Regulation in vitro of brush border myosin by light chain phosphorylation. *J Mol Biol* **1986,** 188, (3), 369-82.
3. Hoshimaru, M.; Nakanishi, S., Identification of a new type of mammalian myosin heavy chain by molecular cloning. Overlap of its mRNA with preprotachykinin B mRNA. *J Biol Chem* **1987,** 262, (30), 14625-32.
4. Mooseker, M. S.; Coleman, T. R., The 110-kD protein-calmodulin complex of the intestinal microvillus (brush border myosin I) is a mechanoenzyme. *The Journal of cell biology* **1989,** 108, (6), 2395-2400.
5. Berg, J. S.; Powell, B. C.; Cheney, R. E., A millennial myosin census. *Mol Biol Cell* **2001,** 12, (4), 780-94.
6. Williams, R.; Coluccio, L. M., Novel 130-kDa rat liver myosin-1 will translocate actin filaments. *Cell Motil Cytoskeleton* **1994,** 27, (1), 41-8.
7. Bement, W. M.; Wirth, J. A.; Mooseker, M. S., Cloning and mRNA expression of human unconventional myosin-IC. A homologue of amoeboid myosins-I with a single IQ motif and an SH3 domain. *Journal of molecular biology* **1994,** 243, (2), 356-363.
8. Arif, E.; Solanki, A. K.; Srivastava, P.; Rahman, B.; Tash, B. R.; Holzman, L. B.; Janech, M. G.; Martin, R.; Knolker, H. J.; Fitzgibbon, W. R.; Deng, P.; Budisavljevic, M. N.; Syn, W. K.; Wang, C.; Lipschutz, J. H.; Kwon, S. H.; Nihalani, D., The motor protein

Myo1c regulates transforming growth factor-beta-signaling and fibrosis in podocytes. *Kidney Int* **2019,** 96, (1), 139-158.
9. Solanki, A. K.; Biswal, M. R.; Walterhouse, S.; Martin, R.; Kondkar, A. A.; Knolker, H. J.; Rahman, B.; Arif, E.; Husain, S.; Montezuma, S. R.; Nihalani, D.; Lobo, G. P., Loss of Motor Protein MYO1C Causes Rhodopsin Mislocalization and Results in Impaired Visual Function. *Cells* **2021,** 10, (6).
10. Bement, W. M.; Hasson, T.; Wirth, J. A.; Cheney, R. E.; Mooseker, M. S., Identification and overlapping expression of multiple unconventional myosin genes in vertebrate cell types. *Proc Natl Acad Sci U S A* **1994,** 91, (24), 11767.
11. Yamazaki, R.; Baba, H.; Yamaguchi, Y., Unconventional Myosin ID is Involved in Remyelination After Cuprizone-Induced Demyelination. *Neurochem Res* **2018,** 43, (1), 195-204.
12. Alsafwani, R. S.; Nasser, K. K.; Shinawi, T.; Banaganapalli, B.; ElSokary, H. A.; Zaher, Z. F.; Shaik, N. A.; Abdelmohsen, G.; Al-Aama, J. Y.; Shapiro, A. J.; O, O. A.-R.; Elango, R.; Alahmadi, T., Novel MYO1D Missense Variant Identified Through Whole Exome Sequencing and Computational Biology Analysis Expands the Spectrum of Causal Genes of Laterality Defects. *Front Med (Lausanne)* **2021,** 8, 724826.
13. Bahler, M.; Kroschewski, R.; Stoffler, H. E.; Behrmann, T., Rat myr 4 defines a novel subclass of myosin I: identification, distribution, localization, and mapping of calmodulin-binding sites with differential calcium sensitivity. *J Cell Biol* **1994,** 126, (2), 375-89.
14. Mele, C.; Iatropoulos, P.; Donadelli, R.; Calabria, A.; Maranta, R.; Cassis, P.; Buelli, S.; Tomasoni, S.; Piras, R.; Krendel, M.; Bettoni, S.; Morigi, M.; Delledonne, M.; Pecoraro, C.; Abbate, I.; Capobianchi, M. R.; Hildebrandt, F.; Otto, E.; Schaefer, F.; Macciardi, F.; Ozaltin, F.; Emre, S.; Ibsirlioglu, T.; Benigni, A.; Remuzzi, G.; Noris, M.; PodoNet, C., MYO1E mutations and childhood familial focal segmental glomerulosclerosis. *N Engl J Med* **2011,** 365, (4), 295-306.
15. Navines-Ferrer, A.; Martin, M., Long-Tailed Unconventional Class I Myosins in Health and Disease. *Int J Mol Sci* **2020,** 21, (7).
16. Crozet, F.; el Amraoui, A.; Blanchard, S.; Lenoir, M.; Ripoll, C.; Vago, P.; Hamel, C.; Fizames, C.; Levi-Acobas, F.; Depetris, D.; Mattei, M. G.; Weil, D.; Pujol, R.; Petit, C., Cloning of the genes encoding two murine and human cochlear unconventional type I myosins. *Genomics* **1997,** 40, (2), 332-41.
17. Diaz-Valencia, J. D.; Estrada-Abreo, L. A.; Rodriguez-Cruz, L.; Salgado-Aguayo, A. R.; Patino-Lopez, G., Class I Myosins, molecular motors involved in cell migration and cancer. *Cell Adh Migr* **2022,** 16, (1), 1-12.
18. Spielmann, M.; Hernandez-Miranda, L. R.; Ceccherini, I.; Weese-Mayer, D. E.; Kragesteen, B. K.; Harabula, I.; Krawitz, P.; Birchmeier, C.; Leonard, N.; Mundlos, S., Mutations in MYO1H cause a recessive form of central hypoventilation with autonomic dysfunction. *J Med Genet* **2017,** 54, (11), 754-761.
19. Patrinou-Georgoulas, M.; John, H. A., The genes and mRNA coding for the heavy chains of chick embryonic skeletal myosin. *Cell* **1977,** 12, (2), 491-9.
20. Xu, W. M.; Gorman, P. A.; Rider, S. H.; Hedge, P. J.; Moore, G.; Prichard, C.; Sheer, D.; Solomon, E., Construction of a genetic map of human chromosome 17 by use of chromosome-mediated gene transfer. *Proc Natl Acad Sci U S A* **1988,** 85, (22), 8563-7.

21. Strehler, E. E.; Strehler-Page, M. A.; Perriard, J. C.; Periasamy, M.; Nadal-Ginard, B., Complete nucleotide and encoded amino acid sequence of a mammalian myosin heavy chain gene. Evidence against intron-dependent evolution of the rod. *J Mol Biol* **1986,** 190, (3), 291-317.
22. Toydemir, R. M.; Rutherford, A.; Whitby, F. G.; Jorde, L. B.; Carey, J. C.; Bamshad, M. J., Mutations in embryonic myosin heavy chain (MYH3) cause Freeman-Sheldon syndrome and Sheldon-Hall syndrome. *Nat Genet* **2006,** 38, (5), 561-5.
23. Pokrzywa, M.; Norum, M.; Lengqvist, J.; Ghobadpour, M.; Abdul-Hussein, S.; Moslemi, A. R.; Tajsharghi, H., Developmental MYH3 Myopathy Associated with Expression of Mutant Protein and Reduced Expression Levels of Embryonic MyHC. *PLoS One* **2015,** 10, (11), e0142094.
24. McDonald, B. L.; Dawkins, R. L.; Robinson, J., Myosin autoantibodies reacting with selective muscle fiber types. *Muscle Nerve* **1979,** 2, (1), 37-43.
25. Matsuoka, R.; Yoshida, M. C.; Kanda, N.; Kimura, M.; Ozasa, H.; Takao, A., Human cardiac myosin heavy chain gene mapped within chromosome region 14q11.2----q13. *Am J Med Genet* **1989,** 32, (2), 279-84.
26. Chen, J. H.; Wang, L. L.; Tao, L.; Qi, B.; Wang, Y.; Guo, Y. J.; Miao, L., Identification of MYH6 as the potential gene for human ischaemic cardiomyopathy. *J Cell Mol Med* **2021,** 25, (22), 10736-10746.
27. Chen, Z. Y.; Hasson, T.; Zhang, D. S.; Schwender, B. J.; Derfler, B. H.; Mooseker, M. S.; Corey, D. P., Myosin-VIIb, a novel unconventional myosin, is a constituent of microvilli in transporting epithelia. *Genomics* **2001,** 72, (3), 285-96.
28. Leinwand, L. A.; Saez, L.; McNally, E.; Nadal-Ginard, B., Isolation and characterization of human myosin heavy chain genes. *Proc Natl Acad Sci U S A* **1983,** 80, (12), 3716-20.
29. Catanzaro, D. F.; Morris, B. J., Human cardiac myosin heavy chain genes. Isolation of a genomic DNA clone and its characterization and of a second unique clone also present in the human genome. *Circ Res* **1986,** 59, (6), 655-62.
30. Fernandez-Prado, R.; Carriazo-Julio, S. M.; Torra, R.; Ortiz, A.; Perez-Gomez, M. V., MYH9-related disease: it does exist, may be more frequent than you think and requires specific therapy. *Clin Kidney J* **2019,** 12, (4), 488-493.
31. Takahashi, M.; Kawamoto, S.; Adelstein, R. S., Evidence for inserted sequences in the head region of nonmuscle myosin specific to the nervous system. Cloning of the cDNA encoding the myosin heavy chain-B isoform of vertebrate nonmuscle myosin. *J Biol Chem* **1992,** 267, (25), 17864-71.
32. Matsuoka, R.; Yoshida, M. C.; Furutani, Y.; Imamura, S.; Kanda, N.; Yanagisawa, M.; Masaki, T.; Takao, A., Human smooth muscle myosin heavy chain gene mapped to chromosomal region 16q12. *Am J Med Genet* **1993,** 46, (1), 61-7.
33. Winters, L. M.; Briggs, M. M.; Schachat, F., The human extraocular muscle myosin heavy chain gene (MYH13) maps to the cluster of fast and developmental myosin genes on chromosome 17. *Genomics* **1998,** 54, (1), 188-9.
34. Leal, A.; Endele, S.; Stengel, C.; Huehne, K.; Loetterle, J.; Barrantes, R.; Winterpacht, A.; Rautenstrauss, B., A novel myosin heavy chain gene in human chromosome 19q13.3. *Gene* **2003,** 312, 165-71.
35. Golomb, E.; Ma, X.; Jana, S. S.; Preston, Y. A.; Kawamoto, S.; Shoham, N. G.; Goldin, E.; Conti, M. A.; Sellers, J. R.; Adelstein, R. S., Identification and characterization of

nonmuscle myosin II-C, a new member of the myosin II family. *J Biol Chem* **2004,** 279, (4), 2800-8.
36. Liang, W.; Wang, L.; Song, X.; Gao, F.; Liu, P.; Lee, T. H.; Peng, K. A., Cochlear Nerve Canal Stenosis: Association With MYH14 and MYH9 Genes. *Ear Nose Throat J* **2021,** 100, (3_suppl), 343S-346S.
37. McGuigan, K.; Phillips, P. C.; Postlethwait, J. H., Evolution of sarcomeric myosin heavy chain genes: evidence from fish. *Mol Biol Evol* **2004,** 21, (6), 1042-56.
38. Kim, H.; Lim, J.; Bao, H.; Jiao, B.; Canon, S. M.; Epstein, M. P.; Xu, K.; Jiang, J.; Parameswaran, J.; Li, Y.; Moberg, K. H.; Landers, J. E.; Fournier, C.; Allen, E. G.; Glass, J. D.; Wingo, T. S.; Jin, P., Rare variants in MYH15 modify amyotrophic lateral sclerosis risk. *Hum Mol Genet* **2019,** 28, (14), 2309-2318.
39. Dose, A. C.; Burnside, B., Cloning and chromosomal localization of a human class III myosin. *Genomics* **2000,** 67, (3), 333-42.
40. Grati, M.; Yan, D.; Raval, M. H.; Walsh, T.; Ma, Q.; Chakchouk, I.; Kannan-Sundhari, A.; Mittal, R.; Masmoudi, S.; Blanton, S. H.; Tekin, M.; King, M. C.; Yengo, C. M.; Liu, X. Z., MYO3A Causes Human Dominant Deafness and Interacts with Protocadherin 15-CD2 Isoform. *Hum Mutat* **2016,** 37, (5), 481-7.
41. Dose, A. C.; Burnside, B., A class III myosin expressed in the retina is a potential candidate for Bardet-Biedl syndrome. *Genomics* **2002,** 79, (5), 621-4.
42. Sanders, G.; Lichte, B.; Meyer, H. E.; Kilimann, M. W., cDNA encoding the chicken ortholog of the mouse dilute gene product. Sequence comparison reveals a myosin I subfamily with conserved C-terminal domains. *FEBS Lett* **1992,** 311, (3), 295-8.
43. Van Gele, M.; Dynoodt, P.; Lambert, J., Griscelli syndrome: a model system to study vesicular trafficking. *Pigment Cell Melanoma Res* **2009,** 22, (3), 268-82.
44. Zhao, L. P.; Koslovsky, J. S.; Reinhard, J.; Bahler, M.; Witt, A. E.; Provance, D. W., Jr.; Mercer, J. A., Cloning and characterization of myr 6, an unconventional myosin of the dilute/myosin-V family. *Proc Natl Acad Sci U S A* **1996,** 93, (20), 10826-31.
45. Rodriguez, O. C.; Cheney, R. E., Human myosin-Vc is a novel class V myosin expressed in epithelial cells. *J Cell Sci* **2002,** 115, (Pt 5), 991-1004.
46. Hasson, T.; Heintzelman, M. B.; Santos-Sacchi, J.; Corey, D. P.; Mooseker, M. S., Expression in cochlea and retina of myosin VIIa, the gene product defective in Usher syndrome type 1B. *Proc Natl Acad Sci U S A* **1995,** 92, (21), 9815-9.
47. Gorman, S. W.; Haider, N. B.; Grieshammer, U.; Swiderski, R. E.; Kim, E.; Welch, J. W.; Searby, C.; Leng, S.; Carmi, R.; Sheffield, V. C.; Duhl, D. M., The cloning and developmental expression of unconventional myosin IXA (MYO9A) a gene in the Bardet-Biedl syndrome (BBS4) region at chromosome 15q22-q23. *Genomics* **1999,** 59, (2), 150-60.
48. Berg, J. S.; Derfler, B. H.; Pennisi, C. M.; Corey, D. P.; Cheney, R. E., Myosin-X, a novel myosin with pleckstrin homology domains, associates with regions of dynamic actin. *Journal of cell science* **2000,** 113 Pt 19, 3439-3451.
49. Yonezawa, S.; Kimura, A.; Koshiba, S.; Masaki, S.; Ono, T.; Hanai, A.; Sonta, S.; Kageyama, T.; Takahashi, T.; Moriyama, A., Mouse myosin X: molecular architecture and tissue expression as revealed by northern blot and in situ hybridization analyses. *Biochem Biophys Res Commun* **2000,** 271, (2), 526-33.
50. Courson, D. S.; Cheney, R. E., Myosin-X and disease. *Exp Cell Res* **2015,** 334, (1), 10-5.

51. Probst, F. J.; Fridell, R. A.; Raphael, Y.; Saunders, T. L.; Wang, A.; Liang, Y.; Morell, R. J.; Touchman, J. W.; Lyons, R. H.; Noben-Trauth, K.; Friedman, T. B.; Camper, S. A., Correction of deafness in shaker-2 mice by an unconventional myosin in a BAC transgene. *Science* **1998,** 280, (5368), 1444-7.
52. Wang, A.; Liang, Y.; Fridell, R. A.; Probst, F. J.; Wilcox, E. R.; Touchman, J. W.; Morton, C. C.; Morell, R. J.; Noben-Trauth, K.; Camper, S. A.; Friedman, T. B., Association of unconventional myosin MYO15 mutations with human nonsyndromic deafness DFNB3. *Science* **1998,** 280, (5368), 1447-51.
53. Wakabayashi, Y.; Takahashi, Y.; Kikkawa, Y.; Okano, H.; Mishima, Y.; Ushiki, T.; Yonekawa, H.; Kominami, R., A novel type of myosin encoded by the mouse deafness gene shaker-2. *Biochem Biophys Res Commun* **1998,** 248, (3), 655-9.
54. Patel, K. G.; Liu, C.; Cameron, P. L.; Cameron, R. S., Myr 8, a novel unconventional myosin expressed during brain development associates with the protein phosphatase catalytic subunits 1alpha and 1gamma1. *J Neurosci* **2001,** 21, (20), 7954-68.
55. Telek, E.; Kengyel, A.; Bugyi, B., Myosin XVI in the Nervous System. *Cells* **2020,** 9, (8).
56. Furusawa, T.; Ikawa, S.; Yanai, N.; Obinata, M., Isolation of a novel PDZ-containing myosin from hematopoietic supportive bone marrow stromal cell lines. *Biochem Biophys Res Commun* **2000,** 270, (1), 67-75.
57. Taft, M. H.; Latham, S. L., Myosin XVIII. *Adv Exp Med Biol* **2020,** 1239, 421-438.
58. Quintero, O. A.; DiVito, M. M.; Adikes, R. C.; Kortan, M. B.; Case, L. B.; Lier, A. J.; Panaretos, N. S.; Slater, S. Q.; Rengarajan, M.; Feliu, M.; Cheney, R. E., Human Myo19 is a novel myosin that associates with mitochondria. *Curr Biol* **2009,** 19, (23), 2008-13.
59. Sellers, J. R.; Pato, M. D., The binding of smooth muscle myosin light chain kinase and phosphatases to actin and myosin. *The Journal of biological chemistry* **1984,** 259, (12), 7740-7746.
60. Smith, L.; Stull, J. T., Myosin light chain kinase binding to actin filaments. *FEBS letters* **2000,** 480, (2-3), 298-300.
61. Smith, L.; Su, X.; Lin, P.; Zhi, G.; Stull, J. T., Identification of a novel actin binding motif in smooth muscle myosin light chain kinase. *The Journal of biological chemistry* **1999,** 274, (41), 29433-29438.
62. Hatch, V.; Zhi, G.; Smith, L.; Stull, J. T.; Craig, R.; Lehman, W., Myosin light chain kinase binding to a unique site on F-actin revealed by three-dimensional image reconstruction. *The Journal of cell biology* **2001,** 154, (3), 611-617.
63. Dugina, V. B.; Shagieva, G. S.; Kopnin, P. B., Biological Role of Actin Isoforms in Mammalian Cells. *Biochemistry (Mosc)* **2019,** 84, (6), 583-592.
64. Bugyi, B.; Kellermayer, M., The discovery of actin: "to see what everyone else has seen, and to think what nobody has thought". *J Muscle Res Cell Motil* **2020,** 41, (1), 3-9.
65. Parker, F.; Baboolal, T. G.; Peckham, M., Actin Mutations and Their Role in Disease. *Int J Mol Sci* **2020,** 21, (9).
66. Kovats, J., Myosin and actomyosin content of the heart-muscle. *Nature* **1949,** 163, (4146), 606.
67. Carroll, S. L.; Bergsma, D. J.; Schwartz, R. J., Structure and complete nucleotide sequence of the chicken alpha-smooth muscle (aortic) actin gene. An actin gene which produces multiple messenger RNAs. *J Biol Chem* **1986,** 261, (19), 8965-76.

68. Csapo, A., Adenosine triphosphatase activity; spontaneous dissociation and recombination of actomyosin in extracts from cross-striated and uterine muscle. *Nature* **1949,** 164, (4173), 702.
69. Whalen, R. G.; Butler-Browne, G. S.; Gros, F., Protein synthesis and actin heterogeneity in calf muscle cells in culture. *Proc Natl Acad Sci U S A* **1976,** 73, (6), 2018-22.
70. Garrels, J. I.; Gibson, W., Identification and characterization of multiple forms of actin. *Cell* **1976,** 9, (4 PT 2), 793-805.
71. Engel, J. N.; Gunning, P. W.; Kedes, L., Isolation and characterization of human actin genes. *Proc Natl Acad Sci U S A* **1981,** 78, (8), 4674-8.
72. Pazmiño, N. H.; Ihle, J. N.; McEwan, R. N.; Goldstein, A. L., Control of differentiation of thymocyte precursors in the bone marrow by thymic hormones. *Cancer Treat Rep* **1978,** 62, (11), 1749-1755.
73. Hoch, K.; Volk, D. E., Structures of Thymosin Proteins. *Vitam Horm* **2016,** 102, 1-24.
74. Dominari, A.; Hathaway Iii, D.; Pandav, K.; Matos, W.; Biswas, S.; Reddy, G.; Thevuthasan, S.; Khan, M. A.; Mathew, A.; Makkar, S. S.; Zaidi, M.; Fahem, M. M. M.; Beas, R.; Castaneda, V.; Paul, T.; Halpern, J.; Baralt, D., Thymosin alpha 1: A comprehensive review of the literature. *World J Virol* **2020,** 9, (5), 67-78.
75. Clauss, I. M.; Wathelet, M. G.; Szpirer, J.; Islam, M. Q.; Levan, G.; Szpirer, C.; Huez, G. A., Human thymosin-beta 4/6-26 gene is part of a multigene family composed of seven members located on seven different chromosomes. *Genomics* **1991,** 9, (1), 174-80.
76. Erickson-Viitanen, S.; Ruggieri, S.; Natalini, P.; Horecker, B. L., Thymosin beta 10, a new analog of thymosin beta 4 in mammalian tissues. *Archives of biochemistry and biophysics* **1983,** 225, (2), 407-413.
77. Bao, L.; Loda, M.; Janmey, P. A.; Stewart, R.; Anand-Apte, B.; Zetter, B. R., Thymosin beta 15: a novel regulator of tumor cell motility upregulated in metastatic prostate cancer. *Nat Med* **1996,** 2, (12), 1322-8.
78. Carlsson, L.; Nyström, L. E.; Sundkvist, I.; Markey, F.; Lindberg, U., Actin polymerizability is influenced by profilin, a low molecular weight protein in non-muscle cells. *Journal of molecular biology* **1977,** 115, (3), 465-483.
79. Krishnan, K.; Moens, P. D. J., Structure and functions of profilins. *Biophys Rev* **2009,** 1, (2), 71-81.
80. Murk, K.; Ornaghi, M.; Schiweck, J., Profilin Isoforms in Health and Disease - All the Same but Different. *Front Cell Dev Biol* **2021,** 9, 681122.
81. Honoré, B.; Madsen, P.; Andersen, A. H.; Leffers, H., Cloning and expression of a novel human profilin variant, profilin II. *FEBS letters* **1993,** 330, (2), 151-155.
82. Braun, A.; Aszódi, A.; Hellebrand, H.; Berna, A.; Fässler, R.; Brandau, O., Genomic organization of profilin-III and evidence for a transcript expressed exclusively in testis. *Gene* **2002,** 283, (1-2), 219-225.
83. Woychik, R. P.; Maas, R. L.; Zeller, R.; Vogt, T. F.; Leder, P., 'Formins': proteins deduced from the alternative transcripts of the limb deformity gene. *Nature* **1990,** 346, (6287), 850-853.
84. Young, K. G.; Copeland, J. W., Formins in cell signaling. *Biochim Biophys Acta* **2010,** 1803, (2), 183-90.
85. Leader, B.; Leder, P., Formin-2, a novel formin homology protein of the cappuccino subfamily, is highly expressed in the developing and adult central nervous system. *Mechanisms of development* **2000,** 93, (1-2), 221-231.

86. Katoh, M.; Katoh, M., Identification and characterization of human FMNL1, FMNL2 and FMNL3 genes in silico. *Int J Oncol* **2003,** 22, (5), 1161-8.
87. Bai, S. W.; Herrera-Abreu, M. T.; Rohn, J. L.; Racine, V.; Tajadura, V.; Suryavanshi, N.; Bechtel, S.; Wiemann, S.; Baum, B.; Ridley, A. J., Identification and characterization of a set of conserved and new regulators of cytoskeletal organization, cell morphology and migration. *BMC Biol* **2011,** 9, 54.
88. Calvo, V.; Izquierdo, M., Role of Actin Cytoskeleton Reorganization in Polarized Secretory Traffic at the Immunological Synapse. *Front Cell Dev Biol* **2021,** 9, 629097.
89. Kage, F.; Steffen, A.; Ellinger, A.; Ranftler, C.; Gehre, C.; Brakebusch, C.; Pavelka, M.; Stradal, T.; Rottner, K., FMNL2 and -3 regulate Golgi architecture and anterograde transport downstream of Cdc42. *Sci Rep* **2017,** 7, (1), 9791.
90. Westendorf, J. J.; Mernaugh, R.; Hiebert, S. W., Identification and characterization of a protein containing formin homology (FH1/FH2) domains. *Gene* **1999,** 232, (2), 173-82.
91. Takeya, R.; Sumimoto, H., Fhos, a mammalian formin, directly binds to F-actin via a region N-terminal to the FH1 domain and forms a homotypic complex via the FH2 domain to promote actin fiber formation. *J Cell Sci* **2003,** 116, (Pt 22), 4567-75.
92. Katoh, M.; Katoh, M., Identification and characterization of human FHOD3 gene in silico. *Int J Mol Med* **2004,** 13, (4), 615-20.
93. Kanaya, H.; Takeya, R.; Takeuchi, K.; Watanabe, N.; Jing, N.; Sumimoto, H., Fhos2, a novel formin-related actin-organizing protein, probably associates with the nestin intermediate filament. *Genes Cells* **2005,** 10, (7), 665-78.
94. Habas, R.; Kato, Y.; He, X., Wnt/Frizzled activation of Rho regulates vertebrate gastrulation and requires a novel Formin homology protein Daam1. *Cell* **2001,** 107, (7), 843-854.
95. Katoh, M.; Katoh, M., Identification and characterization of human DAAM2 gene in silico. *International journal of oncology* **2003,** 22, (4), 915-920.
96. Watanabe, N.; Madaule, P.; Reid, T.; Ishizaki, T.; Watanabe, G.; Kakizuka, A.; Saito, Y.; Nakao, K.; Jockusch, B. M.; Narumiya, S., p140mDia, a mammalian homolog of Drosophila diaphanous, is a target protein for Rho small GTPase and is a ligand for profilin. *The EMBO journal* **1997,** 16, (11), 3044-3056.
97. Ercan-Sencicek, A. G.; Jambi, S.; Franjic, D.; Nishimura, S.; Li, M.; El-Fishawy, P.; Morgan, T. M.; Sanders, S. J.; Bilguvar, K.; Suri, M.; Johnson, M. H.; Gupta, A. R.; Yuksel, Z.; Mane, S.; Grigorenko, E.; Picciotto, M.; Alberts, A. S.; Gunel, M.; Sestan, N.; State, M. W., Homozygous loss of DIAPH1 is a novel cause of microcephaly in humans. *Eur J Hum Genet* **2015,** 23, (2), 165-72.
98. Lin, Y. N.; Windhorst, S., Diaphanous-related formin 1 as a target for tumor therapy. *Biochem Soc Trans* **2016,** 44, (5), 1289-1293.
99. Bione, S.; Sala, C.; Manzini, C.; Arrigo, G.; Zuffardi, O.; Banfi, S.; Borsani, G.; Jonveaux, P.; Philippe, C.; Zuccotti, M.; Ballabio, A.; Toniolo, D., A human homologue of the Drosophila melanogaster diaphanous gene is disrupted in a patient with premature ovarian failure: evidence for conserved function in oogenesis and implications for human sterility. *Am J Hum Genet* **1998,** 62, (3), 533-41.
100. Katoh, M.; Katoh, M., Identification and characterization of human DIAPH3 gene in silico. *International journal of molecular medicine* **2004,** 13, (3), 473-478.

101. Stastna, J.; Pan, X.; Wang, H.; Kollmannsperger, A.; Kutscheidt, S.; Lohmann, V.; Grosse, R.; Fackler, O. T., Differing and isoform-specific roles for the formin DIAPH3 in plasma membrane blebbing and filopodia formation. *Cell Res* **2012,** 22, (4), 728-45.
102. Morley, S.; You, S.; Pollan, S.; Choi, J.; Zhou, B.; Hager, M. H.; Steadman, K.; Spinelli, C.; Rajendran, K.; Gertych, A.; Kim, J.; Adam, R. M.; Yang, W.; Krishnan, R.; Knudsen, B. S.; Di Vizio, D.; Freeman, M. R., Regulation of microtubule dynamics by DIAPH3 influences amoeboid tumor cell mechanics and sensitivity to taxanes. *Sci Rep* **2015,** 5, 12136.
103. Chhabra, E. S.; Higgs, H. N., INF2 Is a WASP homology 2 motif-containing formin that severs actin filaments and accelerates both polymerization and depolymerization. *J Biol Chem* **2006,** 281, (36), 26754-67.
104. Lamm, K. Y. B.; Johnson, M. L.; Baker Phillips, J.; Muntifering, M. B.; James, J. M.; Jones, H. N.; Redline, R. W.; Rokas, A.; Muglia, L. J., Inverted formin 2 regulates intracellular trafficking, placentation, and pregnancy outcome. *Elife* **2018,** 7.
105. Labat-de-Hoz, L.; Alonso, M. A., The formin INF2 in disease: progress from 10 years of research. *Cell Mol Life Sci* **2020,** 77, (22), 4581-4600.
106. Gasca, S.; Hill, D. P.; Klingensmith, J.; Rossant, J., Characterization of a gene trap insertion into a novel gene, cordon-bleu, expressed in axial structures of the gastrulating mouse embryo. *Dev Genet* **1995,** 17, (2), 141-154.
107. Renault, L.; Bugyi, B.; Carlier, M.-F., Spire and Cordon-bleu: multifunctional regulators of actin dynamics. *Trends in cell biology* **2008,** 18, (10), 494-504.
108. Dominguez, R., The WH2 Domain and Actin Nucleation: Necessary but Insufficient. *Trends Biochem Sci* **2016,** 41, (6), 478-490.
109. Shikama, N.; Lee, C. W.; France, S.; Delavaine, L.; Lyon, J.; Krstic-Demonacos, M.; La Thangue, N. B., A novel cofactor for p300 that regulates the p53 response. *Molecular cell* **1999,** 4, (3), 365-376.
110. Conley, C. A.; Fritz-Six, K. L.; Almenar-Queralt, A.; Fowler, V. M., Leiomodins: larger members of the tropomodulin (Tmod) gene family. *Genomics* **2001,** 73, (2), 127-139.
111. Tolkatchev, D.; Gregorio, C. C.; Kostyukova, A. S., The role of leiomodin in actin dynamics: a new road or a secret gate. *The FEBS journal* **2021**.
112. Nanda, V.; Miano, J. M., Leiomodin 1, a new serum response factor-dependent target gene expressed preferentially in differentiated smooth muscle cells. *The Journal of biological chemistry* **2012,** 287, (4), 2459-2467.
113. Mullins, R. D.; Stafford, W. F.; Pollard, T. D., Structure, subunit topology, and actin-binding activity of the Arp2/3 complex from Acanthamoeba. *J Cell Biol* **1997,** 136, (2), 331-43.
114. Welch, M. D.; Iwamatsu, A.; Mitchison, T. J., Actin polymerization is induced by Arp2/3 protein complex at the surface of Listeria monocytogenes. *Nature* **1997,** 385, (6613), 265-9.
115. Pollard, T. D.; Beltzner, C. C., Structure and function of the Arp2/3 complex. *Curr Opin Struct Biol* **2002,** 12, (6), 768-74.
116. Molinie, N.; Gautreau, A., The Arp2/3 Regulatory System and Its Deregulation in Cancer. *Physiological reviews* **2018,** 98, (1), 215-238.
117. Eckley, D. M.; Gill, S. R.; Melkonian, K. A.; Bingham, J. B.; Goodson, H. V.; Heuser, J. E.; Schroer, T. A., Analysis of dynactin subcomplexes reveals a novel actin-related

protein associated with the arp1 minifilament pointed end. *The Journal of cell biology* **1999,** 147, (2), 307-320.

118. Eckley, D. M.; Schroer, T. A., Interactions between the evolutionarily conserved, actin-related protein, Arp11, actin, and Arp1. *Molecular biology of the cell* **2003,** 14, (7), 2645-2654.

119. Symons, M.; Derry, J. M.; Karlak, B.; Jiang, S.; Lemahieu, V.; McCormick, F.; Francke, U.; Abo, A., Wiskott-Aldrich syndrome protein, a novel effector for the GTPase CDC42Hs, is implicated in actin polymerization. *Cell* **1996,** 84, (5), 723-734.

120. Miki, H.; Suetsugu, S.; Takenawa, T., WAVE, a novel WASP-family protein involved in actin reorganization induced by Rac. *The EMBO journal* **1998,** 17, (23), 6932-6941.

121. Tang, Q.; Schaks, M.; Koundinya, N.; Yang, C.; Pollard, L. W.; Svitkina, T. M.; Rottner, K.; Goode, B. L., WAVE1 and WAVE2 have distinct and overlapping roles in controlling actin assembly at the leading edge. *Molecular biology of the cell* **2020,** 31, (20), 2168-2178.

122. Suetsugu, S.; Miki, H.; Takenawa, T., Identification of two human WAVE/SCAR homologues as general actin regulatory molecules which associate with the Arp2/3 complex. *Biochemical and biophysical research communications* **1999,** 260, (1), 296-302.

123. Miki, H.; Miura, K.; Takenawa, T., N-WASP, a novel actin-depolymerizing protein, regulates the cortical cytoskeletal rearrangement in a PIP2-dependent manner downstream of tyrosine kinases. *The EMBO journal* **1996,** 15, (19), 5326-5335.

124. Martinez-Quiles, N.; Rohatgi, R.; Antón, I. M.; Medina, M.; Saville, S. P.; Miki, H.; Yamaguchi, H.; Takenawa, T.; Hartwig, J. H.; Geha, R. S.; Ramesh, N., WIP regulates N-WASP-mediated actin polymerization and filopodium formation. *Nature cell biology* **2001,** 3, (5), 484-491.

125. Kato, M.; Miki, H.; Kurita, S.; Endo, T.; Nakagawa, H.; Miyamoto, S.; Takenawa, T., WICH, a novel verprolin homology domain-containing protein that functions cooperatively with N-WASP in actin-microspike formation. *Biochemical and biophysical research communications* **2002,** 291, (1), 41-47.

126. Kato, M.; Takenawa, T., WICH, a member of WASP-interacting protein family, cross-links actin filaments. *Biochemical and biophysical research communications* **2005,** 328, (4), 1058-1066.

127. Aspenstrom, P., The WASP-binding protein WIRE has a role in the regulation of the actin filament system downstream of the platelet-derived growth factor receptor. *Exp Cell Res* **2002,** 279, (1), 21-33.

128. Ho, H. Y.; Rohatgi, R.; Ma, L.; Kirschner, M. W., CR16 forms a complex with N-WASP in brain and is a novel member of a conserved proline-rich actin-binding protein family. *Proceedings of the National Academy of Sciences of the United States of America* **2001,** 98, (20), 11306-11311.

129. Fowler, V. M., Identification and purification of a novel Mr 43,000 tropomyosin-binding protein from human erythrocyte membranes. *The Journal of biological chemistry* **1987,** 262, (26), 12792-12800.

130. Weber, A.; Pennise, C. R.; Babcock, G. G.; Fowler, V. M., Tropomodulin caps the pointed ends of actin filaments. *The Journal of cell biology* **1994,** 127, (6 Pt 1), 1627-1635.

131. Mudry, R. E.; Perry, C. N.; Richards, M.; Fowler, V. M.; Gregorio, C. C., The interaction of tropomodulin with tropomyosin stabilizes thin filaments in cardiac myocytes. *J Cell Biol* **2003,** 162, (6), 1057-68.
132. Bettinsoli, P.; Ferrari-Toninelli, G.; Bonini, S. A.; Guarienti, M.; Cangelosi, D.; Varesio, L.; Memo, M., Favorable prognostic role of tropomodulins in neuroblastoma. *Oncotarget* **2018,** 9, (43), 27092-27103.
133. Watakabe, A.; Kobayashi, R.; Helfman, D. M., N-tropomodulin: a novel isoform of tropomodulin identified as the major binding protein to brain tropomyosin. *Journal of cell science* **1996,** 109 (Pt 9), 2299-2310.
134. Cox, P. R.; Fowler, V.; Xu, B.; Sweatt, J. D.; Paylor, R.; Zoghbi, H. Y., Mice lacking Tropomodulin-2 show enhanced long-term potentiation, hyperactivity, and deficits in learning and memory. *Mol Cell Neurosci* **2003,** 23, (1), 1-12.
135. Fowler, V. M.; Dominguez, R., Tropomodulins and Leiomodins: Actin Pointed End Caps and Nucleators in Muscles. *Biophysical journal* **2017,** 112, (9), 1742-1760.
136. Cox, P. R.; Zoghbi, H. Y., Sequencing, expression analysis, and mapping of three unique human tropomodulin genes and their mouse orthologs. *Genomics* **2000,** 63, (1).
137. Stobdan, T.; Jain, P. P.; Xiong, M.; Bafna, V.; Yuan, J. X.; Haddad, G. G., Heterozygous Tropomodulin 3 mice have improved lung vascularization after chronic hypoxia. *Hum Mol Genet* **2021**.
138. Almenar-Queralt, A.; Lee, A.; Conley, C. A.; Ribas de Pouplana, L.; Fowler, V. M., Identification of a novel tropomodulin isoform, skeletal tropomodulin, that caps actin filament pointed ends in fast skeletal muscle. *J Biol Chem* **1999,** 274, (40), 28466-75.
139. Gokhin, D. S.; Ochala, J.; Domenighetti, A. A.; Fowler, V. M., Tropomodulin 1 directly controls thin filament length in both wild-type and tropomodulin 4-deficient skeletal muscle. *Development* **2015,** 142, (24), 4351-62.
140. Rodriguez Del Castillo, A.; Lemaire, S.; Tchakarov, L.; Jeyapragasan, M.; Doucet, J. P.; Vitale, M. L.; Trifaro, J. M., Chromaffin cell scinderin, a novel calcium-dependent actin filament-severing protein. *EMBO J* **1990,** 9, (1), 43-52.
141. Marks, P. W.; Arai, M.; Bandura, J. L.; Kwiatkowski, D. J., Advillin (p92): a new member of the gelsolin/villin family of actin regulatory proteins. *Journal of cell science* **1998,** 111 (Pt 15), 2129-2136.
142. Vermeulen, W.; Vanhaesebrouck, P.; Van Troys, M.; Verschueren, M.; Fant, F.; Goethals, M.; Ampe, C.; Martins, J. C.; Borremans, F. A. M., Solution structures of the C-terminal headpiece subdomains of human villin and advillin, evaluation of headpiece F-actin-binding requirements. *Protein science : a publication of the Protein Society* **2004,** 13, (5), 1276-1287.
143. Pestonjamasp, K. N.; Pope, R. K.; Wulfkuhle, J. D.; Luna, E. J., Supervillin (p205): A novel membrane-associated, F-actin-binding protein in the villin/gelsolin superfamily. *The Journal of cell biology* **1997,** 139, (5), 1255-1269.
144. Goode, B. L.; Drubin, D. G.; Lappalainen, P., Regulation of the cortical actin cytoskeleton in budding yeast by twinfilin, a ubiquitous actin monomer-sequestering protein. *J Cell Biol* **1998,** 142, (3), 723-33.
145. Vartiainen, M. K.; Sarkkinen, E. M.; Matilainen, T.; Salminen, M.; Lappalainen, P., Mammals have two twinfilin isoforms whose subcellular localizations and tissue distributions are differentially regulated. *J Biol Chem* **2003,** 278, (36), 34347-55.

146. Stritt, S.; Beck, S.; Becker, I. C.; Vögtle, T.; Hakala, M.; Heinze, K. G.; Du, X.; Bender, M.; Braun, A.; Lappalainen, P.; Nieswandt, B., Twinfilin 2a regulates platelet reactivity and turnover in mice. *Blood* **2017,** 130, (15), 1746-1756.
147. Larbolette, O.; Wollscheid, B.; Schweikert, J.; Nielsen, P. J.; Wienands, J., SH3P7 is a cytoskeleton adapter protein and is coupled to signal transduction from lymphocyte antigen receptors. *Molecular and cellular biology* **1999,** 19, (2), 1539-1546.
148. Kessels, M. M.; Engqvist-Goldstein, A. E.; Drubin, D. G., Association of mouse actin-binding protein 1 (mAbp1/SH3P7), an Src kinase target, with dynamic regions of the cortical actin cytoskeleton in response to Rac1 activation. *Molecular biology of the cell* **2000,** 11, (1), 393-412.
149. Gieselmann, R.; Mann, K., ASP-56, a new actin sequestering protein from pig platelets with homology to CAP, an adenylate cyclase-associated protein from yeast. *FEBS Lett* **1992,** 298, (2-3), 149-53.
150. Bertling, E.; Hotulainen, P.; Mattila, P. K.; Matilainen, T.; Salminen, M.; Lappalainen, P., Cyclase-associated protein 1 (CAP1) promotes cofilin-induced actin dynamics in mammalian nonmuscle cells. *Mol Biol Cell* **2004,** 15, (5), 2324-34.
151. Kakurina, G. V.; Kolegova, E. S.; Kondakova, I. V., Adenylyl Cyclase-Associated Protein 1: Structure, Regulation, and Participation in Cellular Processes. *Biochemistry. Biokhimiia* **2018,** 83, (1), 45-53.
152. Matviw, H.; Yu, G.; Young, D., Identification of a human cDNA encoding a protein that is structurally and functionally related to the yeast adenylyl cyclase-associated CAP proteins. *Molecular and cellular biology* **1992,** 12, (11), 5033-5040.
153. Yu, G.; Swiston, J.; Young, D., Comparison of human CAP and CAP2, homologs of the yeast adenylyl cyclase-associated proteins. *Journal of cell science* **1994,** 107 (Pt 6), 1671-1678.
154. Marchesi, V. T.; Steers, E., Jr., Selective solubilization of a protein component of the red cell membrane. *Science* **1968,** 159, (3811), 203-4.
155. Tilney, L. G.; Detmers, P., Actin in erythrocyte ghosts and its association with spectrin. Evidence for a nonfilamentous form of these two molecules in situ. *J Cell Biol* **1975,** 66, (3), 508-20.
156. Bennett, V., The molecular basis for membrane - cytoskeleton association in human erythrocytes. *J Cell Biochem* **1982,** 18, (1), 49-65.
157. Winkelmann, J. C.; Forget, B. G., Erythroid and nonerythroid spectrins. *Blood* **1993,** 81, (12), 3173-3185.
158. Machnicka, B.; Grochowalska, R.; Boguslawska, D. M.; Sikorski, A. F., The role of spectrin in cell adhesion and cell-cell contact. *Exp Biol Med (Maywood)* **2019,** 244, (15), 1303-1312.
159. Levine, J.; Willard, M., Fodrin: axonally transported polypeptides associated with the internal periphery of many cells. *The Journal of cell biology* **1981,** 90, (3), 631-642.
160. Sreeja, J. S.; John, R.; Dharmapal, D.; Nellikka, R. K.; Sengupta, S., A Fresh Look at the Structure, Regulation, and Functions of Fodrin. *Molecular and cellular biology* **2020,** 40, (17).
161. Turunen, O.; Wahlström, T.; Vaheri, A., Ezrin has a COOH-terminal actin-binding site that is conserved in the ezrin protein family. *The Journal of cell biology* **1994,** 126, (6), 1445-1453.

162. Bretscher, A., Purification of an 80,000-dalton protein that is a component of the isolated microvillus cytoskeleton, and its localization in nonmuscle cells. *The Journal of cell biology* **1983,** 97, (2), 425-432.
163. Tsukita, S.; Hieda, Y.; Tsukita, S., A new 82-kD barbed end-capping protein (radixin) localized in the cell-to-cell adherens junction: purification and characterization. *The Journal of cell biology* **1989,** 108, (6), 2369-2382.
164. Lankes, W.; Griesmacher, A.; Grünwald, J.; Schwartz-Albiez, R.; Keller, R., A heparin-binding protein involved in inhibition of smooth-muscle cell proliferation. *The Biochemical journal* **1988,** 251, (3), 831-842.
165. Trofatter, J. A.; MacCollin, M. M.; Rutter, J. L.; Murrell, J. R.; Duyao, M. P.; Parry, D. M.; Eldridge, R.; Kley, N.; Menon, A. G.; Pulaski, K., A novel moesin-, ezrin-, radixin-like gene is a candidate for the neurofibromatosis 2 tumor suppressor. *Cell* **1993,** 72, (5), 791-800.
166. Xu, H. M.; Gutmann, D. H., Merlin differentially associates with the microtubule and actin cytoskeleton. *J Neurosci Res* **1998,** 51, (3), 403-15.
167. James, M. F.; Manchanda, N.; Gonzalez-Agosti, C.; Hartwig, J. H.; Ramesh, V., The neurofibromatosis 2 protein product merlin selectively binds F-actin but not G-actin, and stabilizes the filaments through a lateral association. *The Biochemical journal* **2001,** 356, (Pt 2), 377-386.
168. Petrilli, A. M.; Fernández-Valle, C., Role of Merlin/NF2 inactivation in tumor biology. *Oncogene* **2016,** 35, (5), 537-548.
169. Niwa, R.; Nagata-Ohashi, K.; Takeichi, M.; Mizuno, K.; Uemura, T., Control of actin reorganization by Slingshot, a family of phosphatases that dephosphorylate ADF/cofilin. *Cell* **2002,** 108, (2), 233-46.
170. Yamamoto, M.; Nagata-Ohashi, K.; Ohta, Y.; Ohashi, K.; Mizuno, K., Identification of multiple actin-binding sites in cofilin-phosphatase Slingshot-1L. *FEBS Lett* **2006,** 580, (7), 1789-94.
171. Takahashi, K.; Okabe, H.; Kanno, S. I.; Nagai, T.; Mizuno, K., A pleckstrin homology-like domain is critical for F-actin binding and cofilin-phosphatase activity of Slingshot-1. *Biochem Biophys Res Commun* **2017,** 482, (4), 686-692.
172. Kurita, S.; Gunji, E.; Ohashi, K.; Mizuno, K., Actin filaments-stabilizing and -bundling activities of cofilin-phosphatase Slingshot-1. *Genes to cells : devoted to molecular & cellular mechanisms* **2007,** 12, (5), 663-676.
173. de Hostos, E. L.; Bradtke, B.; Lottspeich, F.; Guggenheim, R.; Gerisch, G., Coronin, an actin binding protein of Dictyostelium discoideum localized to cell surface projections, has sequence similarities to G protein beta subunits. *EMBO J* **1991,** 10, (13), 4097-104.
174. Suzuki, K.; Nishihata, J.; Arai, Y.; Honma, N.; Yamamoto, K.; Irimura, T.; Toyoshima, S., Molecular cloning of a novel actin-binding protein, p57, with a WD repeat and a leucine zipper motif. *FEBS letters* **1995,** 364, (3), 283-288.
175. Liu, X.; Gao, Y.; Lin, X.; Li, L.; Han, X.; Liu, J., The Coronin Family and Human Disease. *Current protein & peptide science* **2016,** 17, (6), 603-611.
176. Okumura, M.; Kung, C.; Wong, S.; Rodgers, M.; Thomas, M. L., Definition of family of coronin-related proteins conserved between humans and mice: close genetic linkage between coronin-2 and CD45-associated protein. *DNA and cell biology* **1998,** 17, (9), 779-787.

177. Werner, A.-C.; Weckbach, L. T.; Salvermoser, M.; Pitter, B.; Cao, J.; Maier-Begandt, D.; Forné, I.; Schnittler, H.-J.; Walzog, B.; Montanez, E., Coronin 1B Controls Endothelial Actin Dynamics at Cell-Cell Junctions and Is Required for Endothelial Network Assembly. *Frontiers in cell and developmental biology* **2020,** 8, 708.
178. Iizaka, M.; Han, H. J.; Akashi, H.; Furukawa, Y.; Nakajima, Y.; Sugano, S.; Ogawa, M.; Nakamura, Y., Isolation and chromosomal assignment of a novel human gene, CORO1C, homologous to coronin-like actin-binding proteins. *Cytogenetics and cell genetics* **2000,** 88, (3-4), 221-224.
179. Rosentreter, A.; Hofmann, A.; Xavier, C.-P.; Stumpf, M.; Noegel, A. A.; Clemen, C. S., Coronin 3 involvement in F-actin-dependent processes at the cell cortex. *Experimental cell research* **2007,** 313, (5), 878-895.
180. Cheng, X.; Wang, X.; Wu, Z.; Tan, S.; Zhu, T.; Ding, K., CORO1C expression is associated with poor survival rates in gastric cancer and promotes metastasis in vitro. *FEBS open bio* **2019,** 9, (6), 1097-1108.
181. Zaphiropoulos, P. G.; Toftgård, R., cDNA cloning of a novel WD repeat protein mapping to the 9q22.3 chromosomal region. *DNA and cell biology* **1996,** 15, (12), 1049-1056.
182. Nakamura, T.; Takeuchi, K.; Muraoka, S.; Takezoe, H.; Takahashi, N.; Mori, N., A neurally enriched coronin-like protein, ClipinC, is a novel candidate for an actin cytoskeleton-cortical membrane-linking protein. *The Journal of biological chemistry* **1999,** 274, (19), 13322-13327.
183. Chen, Y.; Xu, J.; Zhang, Y.; Ma, S.; Yi, W.; Liu, S.; Yu, X.; Wang, J.; Chen, Y., Coronin 2B regulates dendrite outgrowth by modulating actin dynamics. *FEBS letters* **2020,** 594, (18), 2975-2987.
184. Chen, Y.; Ip, F. C.; Shi, L.; Zhang, Z.; Tang, H.; Ng, Y. P.; Ye, W. C.; Fu, A. K.; Ip, N. Y., Coronin 6 regulates acetylcholine receptor clustering through modulating receptor anchorage to actin cytoskeleton. *J Neurosci* **2014,** 34, (7), 2413-21.
185. Bhattacharya, K.; Swaminathan, K.; Peche, V. S.; Clemen, C. S.; Knyphausen, P.; Lammers, M.; Noegel, A. A.; Rastetter, R. H., Novel Coronin7 interactions with Cdc42 and N-WASP regulate actin organization and Golgi morphology. *Scientific reports* **2016,** 6, 25411.
186. Rybakin, V.; Stumpf, M.; Schulze, A.; Majoul, I. V.; Noegel, A. A.; Hasse, A., Coronin 7, the mammalian POD-1 homologue, localizes to the Golgi apparatus. *FEBS letters* **2004,** 573, (1-3), 161-167.
187. Wu, H.; Reynolds, A. B.; Kanner, S. B.; Vines, R. R.; Parsons, J. T., Identification and characterization of a novel cytoskeleton-associated pp60src substrate. *Molecular and cellular biology* **1991,** 11, (10), 5113-5124.
188. Schnoor, M.; Stradal, T. E.; Rottner, K., Cortactin: Cell Functions of A Multifaceted Actin-Binding Protein. *Trends in cell biology* **2018,** 28, (2), 79-98.
189. Kitamura, D.; Kaneko, H.; Miyagoe, Y.; Ariyasu, T.; Watanabe, T., Isolation and characterization of a novel human gene expressed specifically in the cells of hematopoietic lineage. *Nucleic acids research* **1989,** 17, (22), 9367-9379.
190. Castro-Ochoa, K. F.; Guerrero-Fonseca, I. M.; Schnoor, M., Hematopoietic cell-specific lyn substrate (HCLS1 or HS1): A versatile actin-binding protein in leukocytes. *Journal of leukocyte biology* **2019,** 105, (5), 881-890.
191. Casella, J. F.; Casella, S. J.; Hollands, J. A.; Caldwell, J. E.; Cooper, J. A., Isolation and characterization of cDNA encoding the alpha subunit of Cap Z(36/32), an actin-capping

protein from the Z line of skeletal muscle. *Proceedings of the National Academy of Sciences of the United States of America* **1989,** 86, (15), 5800-5804.
192. Casella, J. F.; Maack, D. J.; Lin, S., Purification and initial characterization of a protein from skeletal muscle that caps the barbed ends of actin filaments. *The Journal of biological chemistry* **1986,** 261, (23), 10915-10921.
193. Miyagawa, Y.; Tanaka, H.; Iguchi, N.; Kitamura, K.; Nakamura, Y.; Takahashi, T.; Matsumiya, K.; Okuyama, A.; Nishimune, Y., Molecular cloning and characterization of the human orthologue of male germ cell-specific actin capping protein alpha3 (cpalpha3). *Molecular human reproduction* **2002,** 8, (6), 531-539.
194. Barron-Casella, E. A.; Torres, M. A.; Scherer, S. W.; Heng, H. H.; Tsui, L. C.; Casella, J. F., Sequence analysis and chromosomal localization of human Cap Z. Conserved residues within the actin-binding domain may link Cap Z to gelsolin/severin and profilin protein families. *The Journal of biological chemistry* **1995,** 270, (37), 21472-21479.
195. Bamburg, J. R.; Harris, H. E.; Weeds, A. G., Partial purification and characterization of an actin depolymerizing factor from brain. *FEBS letters* **1980,** 121, (1), 178-182.
196. Kanellos, G.; Frame, M. C., Cellular functions of the ADF/cofilin family at a glance. *J Cell Sci* **2016,** 129, (17), 3211-8.
197. Yin, H. L.; Stossel, T. P., Control of cytoplasmic actin gel-sol transformation by gelsolin, a calcium-dependent regulatory protein. *Nature* **1979,** 281, (5732), 583-6.
198. Bretscher, A.; Weber, K., Villin: the major microfilament-associated protein of the intestinal microvillus. *Proceedings of the National Academy of Sciences of the United States of America* **1979,** 76, (5), 2321-2325.
199. Khurana, S.; George, S. P., Regulation of cell structure and function by actin-binding proteins: villin's perspective. *FEBS Lett* **2008,** 582, (14), 2128-39.
200. Schook, W.; Ores, C.; Puszkin, S., Isolation and properties of brain alpha-actinin. *Biochem J* **1978,** 175, (1), 63-72.
201. Davison, M. D.; Critchley, D. R., alpha-Actinins and the DMD protein contain spectrin-like repeats. *Cell* **1988,** 52, (2), 159-160.
202. Youssoufian, H.; McAfee, M.; Kwiatkowski, D. J., Cloning and chromosomal localization of the human cytoskeletal alpha-actinin gene reveals linkage to the beta-spectrin gene. *American journal of human genetics* **1990,** 47, (1), 62-72.
203. Sjoblom, B.; Salmazo, A.; Djinovic-Carugo, K., Alpha-actinin structure and regulation. *Cell Mol Life Sci* **2008,** 65, (17), 2688-701.
204. Murphy, A. C.; Young, P. W., The actinin family of actin cross-linking proteins - a genetic perspective. *Cell Biosci* **2015,** 5, 49.
205. Ebashi, S.; Ebashi, F., A New Protein Factor Promoting Contraction of Actomyosin. *Nature* **1964,** 203, 645-6.
206. Beggs, A. H.; Byers, T. J.; Knoll, J. H.; Boyce, F. M.; Bruns, G. A.; Kunkel, L. M., Cloning and characterization of two human skeletal muscle alpha-actinin genes located on chromosomes 1 and 11. *The Journal of biological chemistry* **1992,** 267, (13), 9281-9288.
207. Pickering, C.; Kiely, J., ACTN3, Morbidity, and Healthy Aging. *Front Genet* **2018,** 9, 15.
208. Honda, K.; Yamada, T.; Endo, R.; Ino, Y.; Gotoh, M.; Tsuda, H.; Yamada, Y.; Chiba, H.; Hirohashi, S., Actinin-4, a novel actin-bundling protein associated with cell motility and cancer invasion. *The Journal of cell biology* **1998,** 140, (6), 1383-1393.

209. Feng, D.; DuMontier, C.; Pollak, M. R., The role of alpha-actinin-4 in human kidney disease. *Cell Biosci* **2015,** 5, 44.
210. Tentler, D.; Lomert, E.; Novitskaya, K.; Barlev, N. A., Role of ACTN4 in Tumorigenesis, Metastasis, and EMT. *Cells* **2019,** 8, (11).
211. Nishida, E.; Maekawa, S.; Sakai, H., Cofilin, a protein in porcine brain that binds to actin filaments and inhibits their interactions with myosin and tropomyosin. *Biochemistry* **1984,** 23, (22), 5307-13.
212. Aizawa, H.; Kishi, Y.; Iida, K.; Sameshima, M.; Yahara, I., Cofilin-2, a novel type of cofilin, is expressed specifically at aggregation stage of Dictyostelium discoideum development. *Genes Cells* **2001,** 6, (10), 913-21.
213. Siegel, D. L.; Branton, D., Partial purification and characterization of an actin-bundling protein, band 4.9, from human erythrocytes. *The Journal of cell biology* **1985,** 100, (3), 775-785.
214. Hartwig, J. H.; Stossel, T. P., Isolation and properties of actin, myosin, and a new actinbinding protein in rabbit alveolar macrophages. *J Biol Chem* **1975,** 250, (14), 5696-705.
215. Wang, K.; Ash, J. F.; Singer, S. J., Filamin, a new high-molecular-weight protein found in smooth muscle and non-muscle cells. *Proc Natl Acad Sci U S A* **1975,** 72, (11), 4483-6.
216. Nakamura, F.; Stossel, T. P.; Hartwig, J. H., The filamins: organizers of cell structure and function. *Cell Adh Migr* **2011,** 5, (2), 160-9.
217. Bandaru, S.; Ala, C.; Zhou, A. X.; Akyurek, L. M., Filamin A Regulates Cardiovascular Remodeling. *Int J Mol Sci* **2021,** 22, (12).
218. Zhou, J.; Kang, X.; An, H.; Lv, Y.; Liu, X., The function and pathogenic mechanism of filamin A. *Gene* **2021,** 784, 145575.
219. Xu, W.; Xie, Z.; Chung, D. W.; Davie, E. W., A novel human actin-binding protein homologue that binds to platelet glycoprotein Ibalpha. *Blood* **1998,** 92, (4), 1268-76.
220. Takafuta, T.; Wu, G.; Murphy, G. F.; Shapiro, S. S., Human beta-filamin is a new protein that interacts with the cytoplasmic tail of glycoprotein Ibalpha. *J Biol Chem* **1998,** 273, (28), 17531-8.
221. Robertson, S., FLNB Disorders. In *GeneReviews((R))*, Adam, M. P.; Ardinger, H. H.; Pagon, R. A.; Wallace, S. E.; Bean, L. J. H.; Mirzaa, G.; Amemiya, A., Eds. Seattle (WA), 1993.
222. Xie, Z.; Xu, W.; Davie, E. W.; Chung, D. W., Molecular cloning of human ABPL, an actin-binding protein homologue. *Biochem Biophys Res Commun* **1998,** 251, (3), 914-9.
223. Thompson, T. G.; Chan, Y. M.; Hack, A. A.; Brosius, M.; Rajala, M.; Lidov, H. G.; McNally, E. M.; Watkins, S.; Kunkel, L. M., Filamin 2 (FLN2): A muscle-specific sarcoglycan interacting protein. *J Cell Biol* **2000,** 148, (1), 115-26.
224. Mao, Z.; Nakamura, F., Structure and Function of Filamin C in the Muscle Z-Disc. *Int J Mol Sci* **2020,** 21, (8).
225. Yu, F. X.; Johnston, P. A.; Südhof, T. C.; Yin, H. L., gCap39, a calcium ion- and polyphosphoinositide-regulated actin capping protein. *Science (New York, N.Y.)* **1990,** 250, (4986), 1413-1415.
226. Grzanka, D.; Gagat, M.; Izdebska, M., Involvement of the SATB1/F-actin complex in chromatin reorganization during active cell death. *Int J Mol Med* **2014,** 33, (6), 1441-50.

227. Takahashi, K.; Hiwada, K.; Kokubu, T., Isolation and characterization of a 34,000-dalton calmodulin- and F-actin-binding protein from chicken gizzard smooth muscle. *Biochemical and biophysical research communications* **1986,** 141, (1), 20-26.
228. Leinweber, B.; Tang, J. X.; Stafford, W. F.; Chalovich, J. M., Calponin interaction with alpha-actinin-actin: evidence for a structural role for calponin. *Biophys J* **1999,** 77, (6), 3208-17.
229. Liu, R.; Jin, J. P., Calponin isoforms CNN1, CNN2 and CNN3: Regulators for actin cytoskeleton functions in smooth muscle and non-muscle cells. *Gene* **2016,** 585, (1), 143-153.
230. Strasser, P.; Gimona, M.; Moessler, H.; Herzog, M.; Small, J. V., Mammalian calponin. Identification and expression of genetic variants. *FEBS Lett* **1993,** 330, (1), 13-8.
231. Masuda, H.; Tanaka, K.; Takagi, M.; Ohgami, K.; Sakamaki, T.; Shibata, N.; Takahashi, K., Molecular cloning and characterization of human non-smooth muscle calponin. *Journal of biochemistry* **1996,** 120, (2), 415-424.
232. Applegate, D.; Feng, W.; Green, R. S.; Taubman, M. B., Cloning and expression of a novel acidic calponin isoform from rat aortic vascular smooth muscle. *The Journal of biological chemistry* **1994,** 269, (14), 10683-10690.
233. Lees-Miller, J. P.; Heeley, D. H.; Smillie, L. B.; Kay, C. M., Isolation and characterization of an abundant and novel 22-kDa protein (SM22) from chicken gizzard smooth muscle. *The Journal of biological chemistry* **1987,** 262, (7), 2988-2993.
234. Assinder, S. J.; Stanton, J.-A. L.; Prasad, P. D., Transgelin: an actin-binding protein and tumour suppressor. *The international journal of biochemistry & cell biology* **2009,** 41, (3), 482-486.
235. Thompson, O.; Moghraby, J. S.; Ayscough, K. R.; Winder, S. J., Depletion of the actin bundling protein SM22/transgelin increases actin dynamics and enhances the tumourigenic phenotypes of cells. *BMC cell biology* **2012,** 13, 1.
236. Kobayashi, R.; Kubota, T.; Hidaka, H., Purification, characterization, and partial sequence analysis of a new 25-kDa actin-binding protein from bovine aorta: a SM22 homolog. *Biochemical and biophysical research communications* **1994,** 198, (3), 1275-1280.
237. Kim, H.-R.; Kwon, M.-S.; Lee, S.; Mun, Y.; Lee, K.-S.; Kim, C.-H.; Na, B.-R.; Kim, B. N. R.; Piragyte, I.; Lee, H.-S.; Jun, Y.; Jin, M. S.; Hyun, Y.-M.; Jung, H. S.; Mun, J. Y.; Jun, C.-D., TAGLN2 polymerizes G-actin in a low ionic state but blocks Arp2/3-nucleated actin branching in physiological conditions. *Scientific reports* **2018,** 8, (1), 5503.
238. Liang, X.; Jin, Y.; Wang, H.; Meng, X.; Tan, Z.; Huang, T.; Fan, S., Transgelin 2 is required for embryo implantation by promoting actin polymerization. *FASEB journal : official publication of the Federation of American Societies for Experimental Biology* **2019,** 33, (4), 5667-5675.
239. Yin, L.-M.; Ulloa, L.; Yang, Y.-Q., Transgelin-2: Biochemical and Clinical Implications in Cancer and Asthma. *Trends in biochemical sciences* **2019,** 44, (10), 885-896.
240. Mori, K.; Muto, Y.; Kokuzawa, J.; Yoshioka, T.; Yoshimura, S.; Iwama, T.; Okano, Y.; Sakai, N., Neuronal protein NP25 interacts with F-actin. *Neuroscience research* **2004,** 48, (4), 439-446.
241. Ren, W. Z.; Ng, G. Y.; Wang, R. X.; Wu, P. H.; O'Dowd, B. F.; Osmond, D. H.; George, S. R.; Liew, C. C., The identification of NP25: a novel protein that is differentially

expressed by neuronal subpopulations. *Brain research. Molecular brain research* **1994,** 22, (1-4), 173-185.
242. Zhang, Q.; Skepper, J. N.; Yang, F.; Davies, J. D.; Hegyi, L.; Roberts, R. G.; Weissberg, P. L.; Ellis, J. A.; Shanahan, C. M., Nesprins: a novel family of spectrin-repeat-containing proteins that localize to the nuclear membrane in multiple tissues. *Journal of cell science* **2001,** 114, (Pt 24), 4485-4498.
243. Cartwright, S.; Karakesisoglou, I., Nesprins in health and disease. *Seminars in cell & developmental biology* **2014,** 29, 169-179.
244. Millard, T. H.; Dawson, J.; Machesky, L. M., Characterisation of IRTKS, a novel IRSp53/MIM family actin regulator with distinct filament bundling properties. *Journal of cell science* **2007,** 120, (Pt 9), 1663-1672.
245. Kane, R. E., Preparation and purification of polymerized actin from sea urchin egg extracts. *The Journal of cell biology* **1975,** 66, (2), 305-315.
246. Liu, H.; Zhang, Y.; Li, L.; Cao, J.; Guo, Y.; Wu, Y.; Gao, W., Fascin actin-bundling protein 1 in human cancer: promising biomarker or therapeutic target? *Mol Ther Oncolytics* **2021,** 20, 240-264.
247. Tubb, B. E.; Bardien-Kruger, S.; Kashork, C. D.; Shaffer, L. G.; Ramagli, L. S.; Xu, J.; Siciliano, M. J.; Bryan, J., Characterization of human retinal fascin gene (FSCN2) at 17q25: close physical linkage of fascin and cytoplasmic actin genes. *Genomics* **2000,** 65, (2), 146-156.
248. Tubb, B.; Mulholland, D. J.; Vogl, W.; Lan, Z.-J.; Niederberger, C.; Cooney, A.; Bryan, J., Testis fascin (FSCN3): a novel paralog of the actin-bundling protein fascin expressed specifically in the elongate spermatid head. *Experimental cell research* **2002,** 275, (1).
249. Maul, R. S.; Chang, D. D., EPLIN, epithelial protein lost in neoplasm. *Oncogene* **1999,** 18, (54), 7838-7841.
250. Campbell, H. D.; Schimansky, T.; Claudianos, C.; Ozsarac, N.; Kasprzak, A. B.; Cotsell, J. N.; Young, I. G.; de Couet, H. G.; Miklos, G. L., The Drosophila melanogaster flightless-I gene involved in gastrulation and muscle degeneration encodes gelsolin-like and leucine-rich repeat domains and is conserved in Caenorhabditis elegans and humans. *Proc Natl Acad Sci U S A* **1993,** 90, (23), 11386-90.
251. Coghill, I. D.; Brown, S.; Cottle, D. L.; McGrath, M. J.; Robinson, P. A.; Nandurkar, H. H.; Dyson, J. M.; Mitchell, C. A., FHL3 is an actin-binding protein that regulates alpha-actinin-mediated actin bundling: FHL3 localizes to actin stress fibers and enhances cell spreading and stress fiber disassembly. *The Journal of biological chemistry* **2003,** 278, (26), 24139-24152.
252. Morgan, M. J.; Madgwick, A. J., Slim defines a novel family of LIM-proteins expressed in skeletal muscle. *Biochemical and biophysical research communications* **1996,** 225, (2), 632-638.
253. Samson, T.; Smyth, N.; Janetzky, S.; Wendler, O.; Muller, J. M.; Schule, R.; von der Mark, H.; von der Mark, K.; Wixler, V., The LIM-only proteins FHL2 and FHL3 interact with alpha- and beta-subunits of the muscle alpha7beta1 integrin receptor. *J Biol Chem* **2004,** 279, (27), 28641-52.
254. Adler, H. J.; Winnicki, R. S.; Gong, T. W.; Lomax, M. I., A gene upregulated in the acoustically damaged chick basilar papilla encodes a novel WD40 repeat protein. *Genomics* **1999,** 56, (1), 59-69.

255. Rodal, A. A.; Tetreault, J. W.; Lappalainen, P.; Drubin, D. G.; Amberg, D. C., Aip1p interacts with cofilin to disassemble actin filaments. *The Journal of cell biology* **1999,** 145, (6), 1251-1264.
256. Samstag, Y.; Klemke, M., Ectopic expression of L-plastin in human tumor cells: diagnostic and therapeutic implications. *Adv Enzyme Regul* **2007,** 47, 118-126.
257. Bretscher, A.; Weber, K., Fimbrin, a new microfilament-associated protein present in microvilli and other cell surface structures. *J Cell Biol* **1980,** 86, (1), 335-40.
258. Lin, C. S.; Shen, W.; Chen, Z. P.; Tu, Y. H.; Matsudaira, P., Identification of I-plastin, a human fimbrin isoform expressed in intestine and kidney. *Molecular and cellular biology* **1994,** 14, (4), 2457-2467.
259. Zu, Y. L.; Shigesada, K.; Nishida, E.; Kubota, I.; Kohno, M.; Hanaoka, M.; Namba, Y., 65-kilodalton protein phosphorylated by interleukin 2 stimulation bears two putative actin-binding sites and two calcium-binding sites. *Biochemistry* **1990,** 29, (36), 8319-24.
260. Hamaguchi, H.; Yamada, M.; Noguchi, A.; Fujii, K.; Shibasaki, M.; Mukai, R.; Yabe, T.; Kondo, I., Genetic analysis of human lymphocyte proteins by two-dimensional gel electrophoresis: 2. Genetic polymorphism of lymphocyte cytosol 64K polypeptide. *Human genetics* **1982,** 60, (2), 176-180.
261. Schaffner-Reckinger, E.; Machado, R. A. C., The actin-bundling protein L-plastin-A double-edged sword: Beneficial for the immune response, maleficent in cancer. *Int Rev Cell Mol Biol* **2020,** 355, 109-154.
262. Lin, C. S.; Aebersold, R. H.; Kent, S. B.; Varma, M.; Leavitt, J., Molecular cloning and characterization of plastin, a human leukocyte protein expressed in transformed human fibroblasts. *Molecular and cellular biology* **1988,** 8, (11), 4659-4668.
263. Wolff, L.; Strathmann, E. A.; Müller, I.; Mählich, D.; Veltman, C.; Niehoff, A.; Wirth, B., Plastin 3 in health and disease: a matter of balance. *Cellular and molecular life sciences : CMLS* **2021,** 78, (13), 5275-5301.
264. Bearer, E. L.; Abraham, M. T., 2E4 (kaptin): a novel actin-associated protein from human blood platelets found in lamellipodia and the tips of the stereocilia of the inner ear. *European journal of cell biology* **1999,** 78, (2), 117-126.
265. Seipel, K.; O'Brien, S. P.; Iannotti, E.; Medley, Q. G.; Streuli, M., Tara, a novel F-actin binding protein, associates with the Trio guanine nucleotide exchange factor and regulates actin cytoskeletal organization. *Journal of cell science* **2001,** 114, (Pt 2), 389-399.
266. Wang, Y.; Miller, A. L.; Mooseker, M. S.; Koleske, A. J., The Abl-related gene (Arg) nonreceptor tyrosine kinase uses two F-actin-binding domains to bundle F-actin. *Proc Natl Acad Sci U S A* **2001,** 98, (26), 14865-70.
267. Schlatterer, S. D.; Acker, C. M.; Davies, P., c-Abl in neurodegenerative disease. *Journal of molecular neuroscience : MN* **2011,** 45, (3), 445-452.
268. Khatri, A.; Wang, J.; Pendergast, A. M., Multifunctional Abl kinases in health and disease. *Journal of cell science* **2016,** 129, (1).
269. Nairn, A. C.; Aderem, A., Calmodulin and protein kinase C cross-talk: the MARCKS protein is an actin filament and plasma membrane cross-linking protein regulated by protein kinase C phosphorylation and by calmodulin. *Ciba Found Symp* **1992,** 164, 145-54; discussion 154-61.

270. Hartwig, J. H.; Thelen, M.; Rosen, A.; Janmey, P. A.; Nairn, A. C.; Aderem, A., MARCKS is an actin filament crosslinking protein regulated by protein kinase C and calcium-calmodulin. *Nature* **1992,** 356, (6370), 618-622.
271. Tapp, H.; Al-Naggar, I. M.; Yarmola, E. G.; Harrison, A.; Shaw, G.; Edison, A. S.; Bubb, M. R., MARCKS is a natively unfolded protein with an inaccessible actin-binding site: evidence for long-range intramolecular interactions. *The Journal of biological chemistry* **2005,** 280, (11), 9946-9956.
272. El Amri, M.; Fitzgerald, U.; Schlosser, G., MARCKS and MARCKS-like proteins in development and regeneration. *Journal of biomedical science* **2018,** 25, (1), 43.
273. Sheats, M. K.; Yin, Q.; Fang, S.; Park, J.; Crews, A. L.; Parikh, I.; Dickson, B.; Adler, K. B., MARCKS and Lung Disease. *American journal of respiratory cell and molecular biology* **2019,** 60, (1), 16-27.
274. Chen, Z.; Zhang, W.; Selmi, C.; Ridgway, W. M.; Leung, P. S. C.; Zhang, F.; Gershwin, M. E., The myristoylated alanine-rich C-kinase substrates (MARCKS): A membrane-anchored mediator of the cell function. *Autoimmun Rev* **2021,** 20, (11), 102942.
275. Wohnsland, F.; Steinmetz, M. O.; Aebi, U.; Vergères, G., MARCKS-related protein binds to actin without significantly affecting actin polymerization or network structure. Myristoylated alanine-rich C kinase substrate. *Journal of structural biology* **2000,** 131, (3), 217-224.
276. Nakanishi, H.; Obaishi, H.; Satoh, A.; Wada, M.; Mandai, K.; Satoh, K.; Nishioka, H.; Matsuura, Y.; Mizoguchi, A.; Takai, Y., Neurabin: a novel neural tissue-specific actin filament-binding protein involved in neurite formation. *The Journal of cell biology* **1997,** 139, (4), 951-961.
277. Oliver, C. J.; Terry-Lorenzo, R. T.; Elliott, E.; Bloomer, W. A. C.; Li, S.; Brautigan, D. L.; Colbran, R. J.; Shenolikar, S., Targeting protein phosphatase 1 (PP1) to the actin cytoskeleton: the neurabin I/PP1 complex regulates cell morphology. *Molecular and cellular biology* **2002,** 22, (13), 4690-4701.
278. Satoh, A.; Nakanishi, H.; Obaishi, H.; Wada, M.; Takahashi, K.; Satoh, K.; Hirao, K.; Nishioka, H.; Hata, Y.; Mizoguchi, A.; Takai, Y., Neurabin-II/spinophilin. An actin filament-binding protein with one pdz domain localized at cadherin-based cell-cell adhesion sites. *J Biol Chem* **1998,** 273, (6), 3470-5.
279. Barnes, A. P.; Smith, F. D.; VanDongen, H. M.; VanDongen, A. M. J.; Milgram, S. L., The identification of a second actin-binding region in spinophilin/neurabin II. *Brain research. Molecular brain research* **2004,** 124, (2), 105-113.
280. Sarrouilhe, D.; di Tommaso, A.; Métayé, T.; Ladeveze, V., Spinophilin: from partners to functions. *Biochimie* **2006,** 88, (9), 1099-1113.
281. Wellington, A.; Emmons, S.; James, B.; Calley, J.; Grover, M.; Tolias, P.; Manseau, L., Spire contains actin binding domains and is related to ascidian posterior end mark-5. *Development* **1999,** 126, (23), 5267-74.
282. Otto, I. M.; Raabe, T.; Rennefahrt, U. E.; Bork, P.; Rapp, U. R.; Kerkhoff, E., The p150-Spir protein provides a link between c-Jun N-terminal kinase function and actin reorganization. *Current biology : CB* **2000,** 10, (6), 345-348.
283. Pleiser, S.; Rock, R.; Wellmann, J.; Gessler, M.; Kerkhoff, E., Expression patterns of the mouse Spir-2 actin nucleator. *Gene expression patterns : GEP* **2010,** 10, (7-8), 345-350.
284. Schumacher, N.; Borawski, J. M.; Leberfinger, C. B.; Gessler, M.; Kerkhoff, E., Overlapping expression pattern of the actin organizers Spir-1 and formin-2 in the

developing mouse nervous system and the adult brain. *Gene expression patterns : GEP* **2004,** 4, (3), 249-255.
285. Obata, K.; Shirao, T.; Kojima, N.; Tanaka, H., Molecular events associated with neural development. *Acta Neurochir Suppl (Wien)* **1987,** 41, 3-7.
286. de Hostos, E. L.; Bradtke, B.; Lottspeich, F.; Gerisch, G., Coactosin, a 17 kDa F-actin binding protein from Dictyostelium discoideum. *Cell Motil Cytoskeleton* **1993,** 26, (3), 181-91.
287. Shirao, T., The roles of microfilament-associated proteins, drebrins, in brain morphogenesis: a review. *Journal of biochemistry* **1995,** 117, (2), 231-236.
288. Ishikawa, R., Biochemistry of Drebrin and Its Binding to Actin Filaments. *Advances in experimental medicine and biology* **2017,** 1006, 37-47.
289. Asada, H.; Uyemura, K.; Shirao, T., Actin-binding protein, drebrin, accumulates in submembranous regions in parallel with neuronal differentiation. *Journal of neuroscience research* **1994,** 38, (2), 149-159.
290. Li, M. W.; Xiao, X.; Mruk, D. D.; Lam, Y. L.; Lee, W. M.; Lui, W. Y.; Bonanomi, M.; Silvestrini, B.; Cheng, C. Y., Actin-binding protein drebrin E is involved in junction dynamics during spermatogenesis. *Spermatogenesis* **2011,** 1, (2), 123-136.
291. Bailey, K., Tropomyosin: a new asymmetric protein component of muscle. *Nature* **1946,** 157, 368.
292. Liu, H. P.; Bretscher, A., Disruption of the single tropomyosin gene in yeast results in the disappearance of actin cables from the cytoskeleton. *Cell* **1989,** 57, (2), 233-42.
293. Geeves, M. A.; Hitchcock-DeGregori, S. E.; Gunning, P. W., A systematic nomenclature for mammalian tropomyosin isoforms. *Journal of muscle research and cell motility* **2015,** 36, (2), 147-153.
294. Hitchcock-DeGregori, S. E.; Barua, B., Tropomyosin Structure, Function, and Interactions: A Dynamic Regulator. *Sub-cellular biochemistry* **2017,** 82, 253-284.
295. Bai, F.; Wang, L.; Kawai, M., A study of tropomyosin's role in cardiac function and disease using thin-filament reconstituted myocardium. *Journal of muscle research and cell motility* **2013,** 34, (3-4), 295-310.
296. Matyushenko, A. M.; Levitsky, D. I., Molecular Mechanisms of Pathologies of Skeletal and Cardiac Muscles Caused by Point Mutations in the Tropomyosin Genes. *Biochemistry. Biokhimiia* **2020,** 85, (Suppl 1), S20-S33.
297. Clayton, L.; Reinach, F. C.; Chumbley, G. M.; MacLeod, A. R., Organization of the hTMnm gene. Implications for the evolution of muscle and non-muscle tropomyosins. *J Mol Biol* **1988,** 201, (3), 507-15.
298. Novy, R. E.; Lin, J. L.; Lin, C. S.; Lin, J. J., Human fibroblast tropomyosin isoforms: characterization of cDNA clones and analysis of tropomyosin isoform expression in human tissues and in normal and transformed cells. *Cell motility and the cytoskeleton* **1993,** 25, (3), 267-281.
299. Hardy, S.; Thézé, N.; Lepetit, D.; Allo, M. R.; Thiebaud, P., The Xenopus laevis TM-4 gene encodes non-muscle and cardiac tropomyosin isoforms through alternative splicing. *Gene* **1995,** 156, (2), 265-270.
300. Pacholsky, D.; Vakeel, P.; Himmel, M.; Lowe, T.; Stradal, T.; Rottner, K.; Furst, D. O.; van der Ven, P. F., Xin repeats define a novel actin-binding motif. *J Cell Sci* **2004,** 117, (Pt 22), 5257-68.

301. Wang, Q.; Lin, J. L.-C.; Erives, A. J.; Lin, C.-I.; Lin, J. J.-C., New insights into the roles of Xin repeat-containing proteins in cardiac development, function, and disease. *International review of cell and molecular biology* **2014,** 310.
302. Scheffer, D. I.; Zhang, D.-S.; Shen, J.; Indzhykulian, A.; Karavitaki, K. D.; Xu, Y. J.; Wang, Q.; Lin, J. J.-C.; Chen, Z.-Y.; Corey, D. P., XIRP2, an actin-binding protein essential for inner ear hair-cell stereocilia. *Cell reports* **2015,** 10, (11), 1811-1818.
303. Byers, T. J.; Beggs, A. H.; McNally, E. M.; Kunkel, L. M., Novel actin crosslinker superfamily member identified by a two step degenerate PCR procedure. *FEBS letters* **1995,** 368, (3), 500-504.
304. Leung, C. L.; Sun, D.; Zheng, M.; Knowles, D. R.; Liem, R. K., Microtubule actin cross-linking factor (MACF): a hybrid of dystonin and dystrophin that can interact with the actin and microtubule cytoskeletons. *J Cell Biol* **1999,** 147, (6), 1275-86.
305. Hu, L.; Su, P.; Li, R.; Yin, C.; Zhang, Y.; Shang, P.; Yang, T.; Qian, A., Isoforms, structures, and functions of versatile spectraplakin MACF1. *BMB reports* **2016,** 49, (1), 37-44.
306. Hu, L.; Xiao, Y.; Xiong, Z.; Zhao, F.; Yin, C.; Zhang, Y.; Su, P.; Li, D.; Chen, Z.; Ma, X.; Zhang, G.; Qian, A., MACF1, versatility in tissue-specific function and in human disease. *Seminars in cell & developmental biology* **2017,** 69, 3-8.
307. Sasaki, Y.; Ohsawa, K.; Kanazawa, H.; Kohsaka, S.; Imai, Y., Iba1 is an actin-cross-linking protein in macrophages/microglia. *Biochem Biophys Res Commun* **2001,** 286, (2), 292-7.
308. Autieri, M. V.; Kelemen, S. E.; Wendt, K. W., AIF-1 is an actin-polymerizing and Rac1-activating protein that promotes vascular smooth muscle cell migration. *Circulation research* **2003,** 92, (10), 1107-1114.
309. Utans, U.; Arceci, R. J.; Yamashita, Y.; Russell, M. E., Cloning and characterization of allograft inflammatory factor-1: a novel macrophage factor identified in rat cardiac allografts with chronic rejection. *The Journal of clinical investigation* **1995,** 95, (6), 2954-2962.
310. Schulze, J. O.; Quedenau, C.; Roske, Y.; Adam, T.; Schüler, H.; Behlke, J.; Turnbull, A. P.; Sievert, V.; Scheich, C.; Mueller, U.; Heinemann, U.; Büssow, K., Structural and functional characterization of human Iba proteins. *The FEBS journal* **2008,** 275, (18), 4627-4640.
311. Yasuda-Yamahara, M.; Rogg, M.; Yamahara, K.; Maier, J. I.; Huber, T. B.; Schell, C., AIF1L regulates actomyosin contractility and filopodial extensions in human podocytes. *PloS one* **2018,** 13, (7), e0200487.
312. Kwon, M.-S.; Park, K. R.; Kim, Y.-D.; Na, B.-R.; Kim, H.-R.; Choi, H.-J.; Piragyte, I.; Jeon, H.; Chung, K. H.; Song, W. K.; Eom, S. H.; Jun, C.-D., Swiprosin-1 is a novel actin bundling protein that regulates cell spreading and migration. *PloS one* **2013,** 8, (8), e71626.
313. Park, K. R.; Kwon, M.-S.; An, J. Y.; Lee, J.-G.; Youn, H.-S.; Lee, Y.; Kang, J. Y.; Kim, T. G.; Lim, J. J.; Park, J. S.; Lee, S. H.; Song, W. K.; Cheong, H.-K.; Jun, C.-D.; Eom, S. H., Structural implications of Ca-dependent actin-bundling function of human EFhd2/Swiprosin-1. *Scientific reports* **2016,** 6, 39095.
314. Kogias, G.; Kornhuber, J.; Reimer, D.; Mielenz, D.; Müller, C. P., Swiprosin-1/ EFhd2: from Immune Regulator to Personality and Brain Disorders. *Neurosignals* **2019,** 27, (S1).

315. Lehtonen, S.; Zhao, F.; Lehtonen, E., CD2-associated protein directly interacts with the actin cytoskeleton. *Am J Physiol Renal Physiol* **2002,** 283, (4), F734-43.
316. Tang, V. W.; Brieher, W. M., FSGS3/CD2AP is a barbed-end capping protein that stabilizes actin and strengthens adherens junctions. *The Journal of cell biology* **2013,** 203, (5), 815-833.
317. Wang, Y.; Brieher, W. M., CD2AP links actin to PI3 kinase activity to extend epithelial cell height and constrain cell area. *The Journal of cell biology* **2020,** 219, (1).
318. Provost, P.; Doucet, J.; Stock, A.; Gerisch, G.; Samuelsson, B.; Rådmark, O., Coactosin-like protein, a human F-actin-binding protein: critical role of lysine-75. *The Biochemical journal* **2001,** 359, (Pt 2), 255-263.
319. Kim, J.; Shapiro, M. J.; Bamidele, A. O.; Gurel, P.; Thapa, P.; Higgs, H. N.; Hedin, K. E.; Shapiro, V. S.; Billadeau, D. D., Coactosin-like 1 antagonizes cofilin to promote lamellipodial protrusion at the immune synapse. *PloS one* **2014,** 9, (1), e85090.
320. Disanza, A.; Carlier, M.-F.; Stradal, T. E. B.; Didry, D.; Frittoli, E.; Confalonieri, S.; Croce, A.; Wehland, J.; Di Fiore, P. P.; Scita, G., Eps8 controls actin-based motility by capping the barbed ends of actin filaments. *Nature cell biology* **2004,** 6, (12), 1180-1188.
321. Provenzano, C.; Gallo, R.; Carbone, R.; Di Fiore, P. P.; Falcone, G.; Castellani, L.; Alemà, S., Eps8, a tyrosine kinase substrate, is recruited to the cell cortex and dynamic F-actin upon cytoskeleton remodeling. *Experimental cell research* **1998,** 242, (1), 186-200.
322. Luo, K.; Zhang, L.; Liao, Y.; Zhou, H.; Yang, H.; Luo, M.; Qing, C., Effects and mechanisms of Eps8 on the biological behaviour of malignant tumours (Review). *Oncology reports* **2021,** 45, (3), 824-834.
323. Offenhäuser, N.; Borgonovo, A.; Disanza, A.; Romano, P.; Ponzanelli, I.; Iannolo, G.; Di Fiore, P. P.; Scita, G., The eps8 family of proteins links growth factor stimulation to actin reorganization generating functional redundancy in the Ras/Rac pathway. *Molecular biology of the cell* **2004,** 15, (1), 91-98.
324. Dahmani, M.; Ammar-Khodja, F.; Bonnet, C.; Lefèvre, G. M.; Hardelin, J.-P.; Ibrahim, H.; Mallek, Z.; Petit, C., EPS8L2 is a new causal gene for childhood onset autosomal recessive progressive hearing loss. *Orphanet journal of rare diseases* **2015,** 10, 96.
325. Lohi, O.; Lehto, V. P., EAST, a novel EGF receptor substrate, associates with focal adhesions and actin fibers. *FEBS letters* **1998,** 436, (3), 419-423.
326. Allen, P. B.; Greenfield, A. T.; Svenningsson, P.; Haspeslagh, D. C.; Greengard, P., Phactrs 1-4: A family of protein phosphatase 1 and actin regulatory proteins. *Proceedings of the National Academy of Sciences of the United States of America* **2004,** 101, (18), 7187-7192.
327. Wiezlak, M.; Diring, J.; Abella, J.; Mouilleron, S.; Way, M.; McDonald, N. Q.; Treisman, R., G-actin regulates the shuttling and PP1 binding of the RPEL protein Phactr1 to control actomyosin assembly. *Journal of cell science* **2012,** 125, (Pt 23), 5860-5872.
328. Allen, P. B.; Greenfield, A. T.; Svenningsson, P.; Haspeslagh, D. C.; Greengard, P., Phactrs 1-4: A family of protein phosphatase 1 and actin regulatory proteins. *Proc Natl Acad Sci U S A* **2004,** 101, (18), 7187-92.
329. Mandai, K.; Nakanishi, H.; Satoh, A.; Obaishi, H.; Wada, M.; Nishioka, H.; Itoh, M.; Mizoguchi, A.; Aoki, T.; Fujimoto, T.; Matsuda, Y.; Tsukita, S.; Takai, Y., Afadin: A novel actin filament-binding protein with one PDZ domain localized at cadherin-based cell-to-cell adherens junction. *The Journal of cell biology* **1997,** 139, (2), 517-528.

330. Sakakibara, S.; Maruo, T.; Miyata, M.; Mizutani, K.; Takai, Y., Requirement of the F-actin-binding activity of l-afadin for enhancing the formation of adherens and tight junctions. *Genes to cells : devoted to molecular & cellular mechanisms* **2018,** 23, (3), 185-199.
331. Tochio, T.; Tanaka, H.; Nakata, S.; Hosoya, H., Fructose-1,6-bisphosphate aldolase A is involved in HaCaT cell migration by inducing lamellipodia formation. *Journal of dermatological science* **2010,** 58, (2), 123-129.
332. Chang, Y. C.; Chiou, J.; Yang, Y. F.; Su, C. Y.; Lin, Y. F.; Yang, C. N.; Lu, P. J.; Huang, M. S.; Yang, C. J.; Hsiao, M., Therapeutic Targeting of Aldolase A Interactions Inhibits Lung Cancer Metastasis and Prolongs Survival. *Cancer Res* **2019,** 79, (18), 4754-4766.
333. Sun, J.; He, D.; Fu, Y.; Zhang, R.; Guo, H.; Wang, Z.; Wang, Y.; Gao, T.; Wei, Y.; Guo, Y.; Pang, Q.; Liu, Q., A novel lncRNA ARST represses glioma progression by inhibiting ALDOA-mediated actin cytoskeleton integrity. *Journal of experimental & clinical cancer research : CR* **2021,** 40, (1), 187.
334. Hu, G. F.; Strydom, D. J.; Fett, J. W.; Riordan, J. F.; Vallee, B. L., Actin is a binding protein for angiogenin. *Proceedings of the National Academy of Sciences of the United States of America* **1993,** 90, (4), 1217-1221.
335. Pyatibratov, M. G.; Kostyukova, A. S., New insights into the role of angiogenin in actin polymerization. *International review of cell and molecular biology* **2012,** 295, 175-198.
336. Gao, X.; Xu, Z., Mechanisms of action of angiogenin. *Acta biochimica et biophysica Sinica* **2008,** 40, (7), 619-624.
337. Strydom, D. J., The angiogenins. *Cellular and molecular life sciences : CMLS* **1998,** 54, (8), 811-824.
338. Tello-Montoliu, A.; Patel, J. V.; Lip, G. Y. H., Angiogenin: a review of the pathophysiology and potential clinical applications. *Journal of thrombosis and haemostasis : JTH* **2006,** 4, (9), 1864-1874.
339. Hammonds, R. G., Protein sequence of DMD gene is related to actin-binding domain of alpha-actinin. *Cell* **1987,** 51, (1), 1.
340. Hoffman, E. P.; Brown, R. H., Jr.; Kunkel, L. M., Dystrophin: the protein product of the Duchenne muscular dystrophy locus. *Cell* **1987,** 51, (6), 919-28.
341. Rybakova, I. N.; Amann, K. J.; Ervasti, J. M., A new model for the interaction of dystrophin with F-actin. *J Cell Biol* **1996,** 135, (3), 661-72.
342. Keep, N. H., Structural comparison of actin binding in utrophin and dystrophin. *Neurol Sci* **2000,** 21, (5 Suppl), S929-S937.
343. Muntoni, F.; Torelli, S.; Ferlini, A., Dystrophin and mutations: one gene, several proteins, multiple phenotypes. *Lancet Neurol* **2003,** 2, (12), 731-740.
344. Gao, Q. Q.; McNally, E. M., The Dystrophin Complex: Structure, Function, and Implications for Therapy. *Comprehensive Physiology* **2015,** 5, (3), 1223-1239.
345. Chen, Y.-J.; Spence, H. J.; Cameron, J. M.; Jess, T.; Ilsley, J. L.; Winder, S. J., Direct interaction of beta-dystroglycan with F-actin. *The Biochemical journal* **2003,** 375, (Pt 2), 329-337.
346. Reinhard, M.; Halbrügge, M.; Scheer, U.; Wiegand, C.; Jockusch, B. M.; Walter, U., The 46/50 kDa phosphoprotein VASP purified from human platelets is a novel protein associated with actin filaments and focal contacts. *The EMBO journal* **1992,** 11, (6), 2063-2070.

347. Holt, M. R.; Critchley, D. R.; Brindle, N. P., The focal adhesion phosphoprotein, VASP. *The international journal of biochemistry & cell biology* **1998,** 30, (3), 307-311.
348. Bear, J. E.; Gertler, F. B., Ena/VASP: towards resolving a pointed controversy at the barbed end. *J Cell Sci* **2009,** 122, (Pt 12), 1947-53.
349. Gertler, F. B.; Niebuhr, K.; Reinhard, M.; Wehland, J.; Soriano, P., Mena, a relative of VASP and Drosophila Enabled, is implicated in the control of microfilament dynamics. *Cell* **1996,** 87, (2), 227-39.
350. Ohta, S.; Mineta, T.; Kimoto, M.; Tabuchi, K., Differential display cloning of a novel rat cDNA (RNB6) that shows high expression in the neonatal brain revealed a member of Ena/VASP family. *Biochemical and biophysical research communications* **1997,** 237, (2), 307-312.
351. Bachmann, C.; Fischer, L.; Walter, U.; Reinhard, M., The EVH2 domain of the vasodilator-stimulated phosphoprotein mediates tetramerization, F-actin binding, and actin bundle formation. *The Journal of biological chemistry* **1999,** 274, (33), 23549-23557.
352. Wanner, S. J.; Danos, M. C.; Lohr, J. L.; Miller, J. R., Molecular cloning and expression of Ena/Vasp-like (Evl) during Xenopus development. *Gene expression patterns : GEP* **2005,** 5, (3), 423-428.
353. Nemes, J. P.; Benzow, K. A.; Moseley, M. L.; Ranum, L. P.; Koob, M. D., The SCA8 transcript is an antisense RNA to a brain-specific transcript encoding a novel actin-binding protein (KLHL1). *Human molecular genetics* **2000,** 9, (10), 1543-1551.
354. Aromolaran, K. A.; Benzow, K. A.; Cribbs, L. L.; Koob, M. D.; Piedras-Rentería, E. S., Elimination of the actin-binding domain in kelch-like 1 protein induces T-type calcium channel modulation only in the presence of action potential waveforms. *J Signal Transduct* **2012,** 2012, 505346.
355. Chen, Y.; Derin, R.; Petralia, R. S.; Li, M., Actinfilin, a brain-specific actin-binding protein in postsynaptic density. *The Journal of biological chemistry* **2002,** 277, (34), 30495-30501.
356. Hu, H.-T.; Huang, T.-N.; Hsueh, Y.-P., KLHL17/Actinfilin, a brain-specific gene associated with infantile spasms and autism, regulates dendritic spine enlargement. *Journal of biomedical science* **2020,** 27, (1), 103.
357. Ungewickell, E.; Bennett, P. M.; Calvert, R.; Ohanian, V.; Gratzer, W. B., In vitro formation of a complex between cytoskeletal proteins of the human erythrocyte. *Nature* **1979,** 280, (5725), 811-814.
358. Baines, A. J.; Bennett, P. M.; Carter, E. W.; Terracciano, C., Protein 4.1 and the control of ion channels. *Blood cells, molecules & diseases* **2009,** 42, (3), 211-215.
359. Baines, A. J.; Lu, H.-C.; Bennett, P. M., The Protein 4.1 family: hub proteins in animals for organizing membrane proteins. *Biochimica et biophysica acta* **2014,** 1838, (2), 605-619.
360. Yuan, X.; Piao, L.; Wang, L.; Han, X.; Zhuang, M.; Liu, Z., Pivotal roles of protein 4.1B/DAL-1, a FERM-domain containing protein, in tumor progression (Review). *International journal of oncology* **2019,** 55, (5), 979-987.
361. Parra, M.; Gascard, P.; Walensky, L. D.; Snyder, S. H.; Mohandas, N.; Conboy, J. G., Cloning and characterization of 4.1G (EPB41L2), a new member of the skeletal protein 4.1 (EPB41) gene family. *Genomics* **1998,** 49, (2), 298-306.

362. Gimm, J. A.; An, X.; Nunomura, W.; Mohandas, N., Functional characterization of spectrin-actin-binding domains in 4.1 family of proteins. *Biochemistry* **2002,** 41, (23), 7275-7282.
363. Walensky, L. D.; Blackshaw, S.; Liao, D.; Watkins, C. C.; Weier, H. U.; Parra, M.; Huganir, R. L.; Conboy, J. G.; Mohandas, N.; Snyder, S. H., A novel neuron-enriched homolog of the erythrocyte membrane cytoskeletal protein 4.1. *J Neurosci* **1999,** 19, (15), 6457-67.
364. Parra, M.; Gascard, P.; Walensky, L. D.; Gimm, J. A.; Blackshaw, S.; Chan, N.; Takakuwa, Y.; Berger, T.; Lee, G.; Chasis, J. A.; Snyder, S. H.; Mohandas, N.; Conboy, J. G., Molecular and functional characterization of protein 4.1B, a novel member of the protein 4.1 family with high level, focal expression in brain. *J Biol Chem* **2000,** 275, (5), 3247-55.
365. Collier, N. C.; Wang, K., Purification and properties of human platelet P235. A high molecular weight protein substrate of endogenous calcium-activated protease(s). *J Biol Chem* **1982,** 257, (12), 6937-43.
366. Burridge, K.; Connell, L., Talin: a cytoskeletal component concentrated in adhesion plaques and other sites of actin-membrane interaction. *Cell motility* **1983,** 3, (5-6), 405-417.
367. Haining, A. W.; Lieberthal, T. J.; Del Rio Hernandez, A., Talin: a mechanosensitive molecule in health and disease. *FASEB J* **2016,** 30, (6), 2073-85.
368. Goult, B. T.; Yan, J.; Schwartz, M. A., Talin as a mechanosensitive signaling hub. *J Cell Biol* **2018,** 217, (11), 3776-3784.
369. Malla, R. R.; Vempati, R. K., Talin: A Potential Drug Target for Cancer Therapy. *Curr Drug Metab* **2020,** 21, (1), 25-32.
370. McCann, R. O.; Craig, S. W., Functional genomic analysis reveals the utility of the I/LWEQ module as a predictor of protein:actin interaction. *Biochem Biophys Res Commun* **1999,** 266, (1), 135-40.
371. Monkley, S. J.; Pritchard, C. A.; Critchley, D. R., Analysis of the mammalian talin2 gene TLN2. *Biochem Biophys Res Commun* **2001,** 286, (5), 880-5.
372. Debrand, E.; El Jai, Y.; Spence, L.; Bate, N.; Praekelt, U.; Pritchard, C. A.; Monkley, S. J.; Critchley, D. R., Talin 2 is a large and complex gene encoding multiple transcripts and protein isoforms. *The FEBS journal* **2009,** 276, (6), 1610-1628.
373. Cowell, A. R.; Jacquemet, G.; Singh, A. K.; Varela, L.; Nylund, A. S.; Ammon, Y.-C.; Brown, D. G.; Akhmanova, A.; Ivaska, J.; Goult, B. T., Talin rod domain-containing protein 1 (TLNRD1) is a novel actin-bundling protein which promotes filopodia formation. *The Journal of cell biology* **2021,** 220, (9).
374. Ramadass, R.; Becker, D.; Jendrach, M.; Bereiter-Hahn, J., Spectrally and spatially resolved fluorescence lifetime imaging in living cells: TRPV4-microfilament interactions. *Arch Biochem Biophys* **2007,** 463, (1), 27-36.
375. Goswami, C.; Kuhn, J.; Heppenstall, P. A.; Hucho, T., Importance of non-selective cation channel TRPV4 interaction with cytoskeleton and their reciprocal regulations in cultured cells. *PloS one* **2010,** 5, (7), e11654.
376. Sasaki, S.; Yui, N.; Noda, Y., Actin directly interacts with different membrane channel proteins and influences channel activities: AQP2 as a model. *Biochim Biophys Acta* **2014,** 1838, (2), 514-20.

377. Lee, W. H.; Choong, L. Y.; Mon, N. N.; Lu, S.; Lin, Q.; Pang, B.; Yan, B.; Krishna, V. S. R.; Singh, H.; Tan, T. Z.; Thiery, J. P.; Lim, C. T.; Tan, P. B. O.; Johansson, M.; Harteneck, C.; Lim, Y. P., TRPV4 Regulates Breast Cancer Cell Extravasation, Stiffness and Actin Cortex. *Scientific reports* **2016,** 6, 27903.
378. Shibasaki, K., TRPV4 activation by thermal and mechanical stimuli in disease progression. *Laboratory investigation; a journal of technical methods and pathology* **2020,** 100, (2), 218-223.
379. Sugio, S.; Nagasawa, M.; Kojima, I.; Ishizaki, Y.; Shibasaki, K., Transient receptor potential vanilloid 2 activation by focal mechanical stimulation requires interaction with the actin cytoskeleton and enhances growth cone motility. *FASEB journal : official publication of the Federation of American Societies for Experimental Biology* **2017,** 31, (4), 1368-1381.
380. Santoni, G.; Amantini, C.; Maggi, F.; Marinelli, O.; Santoni, M.; Nabissi, M.; Morelli, M. B., The TRPV2 cation channels: from urothelial cancer invasiveness to glioblastoma multiforme interactome signature. *Laboratory investigation; a journal of technical methods and pathology* **2020,** 100, (2), 186-198.
381. Yadav, M.; Goswami, C., TRPV2 interacts with actin and reorganizes submembranous actin cytoskeleton. *Bioscience reports* **2020,** 40, (10).
382. Noda, Y.; Horikawa, S.; Katayama, Y.; Sasaki, S., Water channel aquaporin-2 directly binds to actin. *Biochem Biophys Res Commun* **2004,** 322, (3), 740-5.
383. Noda, Y.; Sasaki, S., Actin-binding channels. *Prog Brain Res* **2008,** 170, 551-7.
384. Chasan, B.; Geisse, N. A.; Pedatella, K.; Wooster, D. G.; Teintze, M.; Carattino, M. D.; Goldmann, W. H.; Cantiello, H. F., Evidence for direct interaction between actin and the cystic fibrosis transmembrane conductance regulator. *Eur Biophys J* **2002,** 30, (8), 617-24.
385. Ahmed, N.; Ramjeesingh, M.; Wong, S.; Varga, A.; Garami, E.; Bear, C. E., Chloride channel activity of ClC-2 is modified by the actin cytoskeleton. *Biochem J* **2000,** 352 Pt 3, 789-94.
386. McCloskey, D. T.; Doherty, L.; Dai, Y. P.; Miller, L.; Hume, J. R.; Yamboliev, I. A., Hypotonic activation of short ClC3 isoform is modulated by direct interaction between its cytosolic C-terminal tail and subcortical actin filaments. *J Biol Chem* **2007,** 282, (23), 16871-7.
387. Singh, H.; Cousin, M. A.; Ashley, R. H., Functional reconstitution of mammalian 'chloride intracellular channels' CLIC1, CLIC4 and CLIC5 reveals differential regulation by cytoskeletal actin. *FEBS J* **2007,** 274, (24), 6306-16.
388. Berdiev, B. K.; Prat, A. G.; Cantiello, H. F.; Ausiello, D. A.; Fuller, C. M.; Jovov, B.; Benos, D. J.; Ismailov, II, Regulation of epithelial sodium channels by short actin filaments. *J Biol Chem* **1996,** 271, (30), 17704-10.
389. Mazzochi, C.; Bubien, J. K.; Smith, P. R.; Benos, D. J., The carboxyl terminus of the alpha-subunit of the amiloride-sensitive epithelial sodium channel binds to F-actin. *J Biol Chem* **2006,** 281, (10), 6528-38.
390. Brainard, A. M.; Miller, A. J.; Martens, J. R.; England, S. K., Maxi-K channels localize to caveolae in human myometrium: a role for an actin-channel-caveolin complex in the regulation of myometrial smooth muscle K+ current. *Am J Physiol Cell Physiol* **2005,** 289, (1), C49-57.

391. Xu, X.; Forbes, J. G.; Colombini, M., Actin modulates the gating of Neurospora crassa VDAC. *J Membr Biol* **2001,** 180, (1), 73-81.
392. Roman, I.; Figys, J.; Steurs, G.; Zizi, M., Direct measurement of VDAC-actin interaction by surface plasmon resonance. *Biochim Biophys Acta* **2006,** 1758, (4), 479-86.
393. Stolting, G.; de Oliveira, R. C.; Guzman, R. E.; Miranda-Laferte, E.; Conrad, R.; Jordan, N.; Schmidt, S.; Hendriks, J.; Gensch, T.; Hidalgo, P., Direct interaction of CaVbeta with actin up-regulates L-type calcium currents in HL-1 cardiomyocytes. *J Biol Chem* **2015,** 290, (8), 4561-4572.
394. Ohtsuka, T.; Nakanishi, H.; Ikeda, W.; Satoh, A.; Momose, Y.; Nishioka, H.; Takai, Y., Nexilin: a novel actin filament-binding protein localized at cell-matrix adherens junction. *The Journal of cell biology* **1998,** 143, (5), 1227-1238.
395. Wang, W.; Zhang, W.; Han, Y.; Chen, J.; Wang, Y.; Zhang, Z.; Hui, R., NELIN, a new F-actin associated protein, stimulates HeLa cell migration and adhesion. *Biochemical and biophysical research communications* **2005,** 330, (4), 1127-1131.
396. Yang, F.; Zhou, L.; Wang, Q.; You, X.; Li, Y.; Zhao, Y.; Han, X.; Chang, Z.; He, X.; Cheng, C.; Wu, C.; Wang, W.-J.; Hu, F.-Y.; Zhao, T.; Li, Y.; Zhao, M.; Zheng, G.-Y.; Dong, J.; Fan, C.; Yang, J.; Meng, X.; Zhang, Y.; Zhu, X.; Xiong, J.; Tian, X.-L.; Cao, H., NEXN inhibits GATA4 and leads to atrial septal defects in mice and humans. *Cardiovascular research* **2014,** 103, (2), 228-237.
397. Arai, A.; Spencer, J. A.; Olson, E. N., STARS, a striated muscle activator of Rho signaling and serum response factor-dependent transcription. *The Journal of biological chemistry* **2002,** 277, (27), 24453-24459.
398. Lamon, S.; Wallace, M. A.; Russell, A. P., The STARS signaling pathway: a key regulator of skeletal muscle function. *Pflugers Archiv : European journal of physiology* **2014,** 466, (9), 1659-1671.
399. Roof, D. J.; Hayes, A.; Adamian, M.; Chishti, A. H.; Li, T., Molecular characterization of abLIM, a novel actin-binding and double zinc finger protein. *The Journal of cell biology* **1997,** 138, (3), 575-588.
400. Narahara, H.; Sakai, E.; Yamaguchi, Y.; Narahara, S.; Iwatake, M.; Okamoto, K.; Yoshida, N.; Tsukuba, T., Actin binding LIM 1 (abLIM1) negatively controls osteoclastogenesis by regulating cell migration and fusion. *Journal of cellular physiology* **2018,** 234, (1), 486-499.
401. Klimov, E.; Rud'ko, O.; Rakhmanaliev, E.; Sulimova, G., Genomic organisation and tissue specific expression of ABLIM2 gene in human, mouse and rat. *Biochimica et biophysica acta* **2005,** 1730, (1), 1-9.
402. Barrientos, T.; Frank, D.; Kuwahara, K.; Bezprozvannaya, S.; Pipes, G. C. T.; Bassel-Duby, R.; Richardson, J. A.; Katus, H. A.; Olson, E. N.; Frey, N., Two novel members of the ABLIM protein family, ABLIM-2 and -3, associate with STARS and directly bind F-actin. *The Journal of biological chemistry* **2007,** 282, (11), 8393-8403.
403. Krupp, M.; Weinmann, A.; Galle, P. R.; Teufel, A., Actin binding LIM protein 3 (abLIM3). *International journal of molecular medicine* **2006,** 17, (1), 129-133.
404. Matsuda, M.; Yamashita, J. K.; Tsukita, S.; Furuse, M., abLIM3 is a novel component of adherens junctions with actin-binding activity. *European journal of cell biology* **2010,** 89, (11), 807-816.

405. Stevenson, B. R.; Siliciano, J. D.; Mooseker, M. S.; Goodenough, D. A., Identification of ZO-1: a high molecular weight polypeptide associated with the tight junction (zonula occludens) in a variety of epithelia. *The Journal of cell biology* **1986,** 103, (3), 755-766.
406. González-Mariscal, L.; Quirós, M.; Díaz-Coránguez, M., ZO proteins and redox-dependent processes. *Antioxidants & redox signaling* **2011,** 15, (5), 1235-1253.
407. Jesaitis, L. A.; Goodenough, D. A., Molecular characterization and tissue distribution of ZO-2, a tight junction protein homologous to ZO-1 and the Drosophila discs-large tumor suppressor protein. *J Cell Biol* **1994,** 124, (6), 949-61.
408. Wittchen, E. S.; Haskins, J.; Stevenson, B. R., Protein interactions at the tight junction. Actin has multiple binding partners, and ZO-1 forms independent complexes with ZO-2 and ZO-3. *J Biol Chem* **1999,** 274, (49), 35179-85.
409. Haskins, J.; Gu, L.; Wittchen, E. S.; Hibbard, J.; Stevenson, B. R., ZO-3, a novel member of the MAGUK protein family found at the tight junction, interacts with ZO-1 and occludin. *J Cell Biol* **1998,** 141, (1), 199-208.
410. Furuse, M.; Hirase, T.; Itoh, M.; Nagafuchi, A.; Yonemura, S.; Tsukita, S.; Tsukita, S., Occludin: a novel integral membrane protein localizing at tight junctions. *J Cell Biol* **1993,** 123, (6 Pt 2), 1777-88.
411. Schreiber, V.; Moog-Lutz, C.; Régnier, C. H.; Chenard, M. P.; Boeuf, H.; Vonesch, J. L.; Tomasetto, C.; Rio, M. C., Lasp-1, a novel type of actin-binding protein accumulating in cell membrane extensions. *Molecular medicine (Cambridge, Mass.)* **1998,** 4, (10), 675-687.
412. Butt, E.; Raman, D., New Frontiers for the Cytoskeletal Protein LASP1. *Frontiers in oncology* **2018,** 8, 391.
413. Moncman, C. L.; Wang, K., Nebulette: a 107 kD nebulin-like protein in cardiac muscle. *Cell Motil Cytoskeleton* **1995,** 32, (3), 205-25.
414. Nakagawa, H.; Terasaki, A. G.; Suzuki, H.; Ohashi, K.; Miyamoto, S., Short-term retention of actin filament binding proteins on lamellipodial actin bundles. *FEBS letters* **2006,** 580, (13), 3223-3228.
415. Zhu, M.; Settele, F.; Kotak, S.; Sanchez-Pulido, L.; Ehret, L.; Ponting, C. P.; Gonczy, P.; Hoffmann, I., MISP is a novel Plk1 substrate required for proper spindle orientation and mitotic progression. *J Cell Biol* **2013,** 200, (6), 773-87.
416. Maier, B.; Kirsch, M.; Anderhub, S.; Zentgraf, H.; Krämer, A., The novel actin/focal adhesion-associated protein MISP is involved in mitotic spindle positioning in human cells. *Cell cycle (Georgetown, Tex.)* **2013,** 12, (9), 1457-1471.
417. Kumeta, M.; Gilmore, J. L.; Umeshima, H.; Ishikawa, M.; Kitajiri, S.; Horigome, T.; Kengaku, M.; Takeyasu, K., Caprice/MISP is a novel F-actin bundling protein critical for actin-based cytoskeletal reorganizations. *Genes Cells* **2014,** 19, (4), 338-49.
418. Maarof, N. D.; Kumeta, M.; Yoshimura, S. H., Modulation of actin-binding and -bundling activities of MISP/Caprice by multiple phosphorylation. *Biochemical and biophysical research communications* **2021,** 561, 128-135.
419. Kwiatkowski, S.; Seliga, A. K.; Vertommen, D.; Terreri, M.; Ishikawa, T.; Grabowska, I.; Tiebe, M.; Teleman, A. A.; Jagielski, A. K.; Veiga-da-Cunha, M.; Drozak, J., SETD3 protein is the actin-specific histidine N-methyltransferase. *Elife* **2018,** 7.
420. Wilkinson, A. W.; Diep, J.; Dai, S.; Liu, S.; Ooi, Y. S.; Song, D.; Li, T. M.; Horton, J. R.; Zhang, X.; Liu, C.; Trivedi, D. V.; Ruppel, K. M.; Vilches-Moure, J. G.; Casey, K. M.; Mak, J.; Cowan, T.; Elias, J. E.; Nagamine, C. M.; Spudich, J. A.; Cheng, X.; Carette, J.

E.; Gozani, O., SETD3 is an actin histidine methyltransferase that prevents primary dystocia. *Nature* **2019,** 565, (7739), 372-376.
421. Mundel, P.; Heid, H. W.; Mundel, T. M.; Krüger, M.; Reiser, J.; Kriz, W., Synaptopodin: an actin-associated protein in telencephalic dendrites and renal podocytes. *The Journal of cell biology* **1997,** 139, (1), 193-204.
422. Chalovich, J. M.; Schroeter, M. M., Synaptopodin family of natively unfolded, actin binding proteins: physical properties and potential biological functions. *Biophys Rev* **2010,** 2, (4), 181-189.
423. Yap, K.; Drakew, A.; Smilovic, D.; Rietsche, M.; Paul, M. H.; Vuksic, M.; Del Turco, D.; Deller, T., The actin-modulating protein synaptopodin mediates long-term survival of dendritic spines. *eLife* **2020,** 9.
424. Weins, A.; Schwarz, K.; Faul, C.; Barisoni, L.; Linke, W. A.; Mundel, P., Differentiation- and stress-dependent nuclear cytoplasmic redistribution of myopodin, a novel actin-bundling protein. *J Cell Biol* **2001,** 155, (3), 393-404.
425. Beqqali, A.; Monshouwer-Kloots, J.; Monteiro, R.; Welling, M.; Bakkers, J.; Ehler, E.; Verkleij, A.; Mummery, C.; Passier, R., CHAP is a newly identified Z-disc protein essential for heart and skeletal muscle function. *J Cell Sci* **2010,** 123, (Pt 7), 1141-50.
426. van der Loop, F. T.; Schaart, G.; Timmer, E. D.; Ramaekers, F. C.; van Eys, G. J., Smoothelin, a novel cytoskeletal protein specific for smooth muscle cells. *The Journal of cell biology* **1996,** 134, (2), 401-411.
427. Niessen, P.; Clément, S.; Fontao, L.; Chaponnier, C.; Teunissen, B.; Rensen, S.; van Eys, G.; Gabbiani, G., Biochemical evidence for interaction between smoothelin and filamentous actin. *Experimental cell research* **2004,** 292, (1), 170-178.
428. Murali, M.; MacDonald, J. A., Smoothelins and the Control of Muscle Contractility. *Advances in pharmacology (San Diego, Calif.)* **2018,** 81, 39-78.
429. Wang, L.; Nakamura, F., Identification of Filamin A Mechanobinding Partner I: Smoothelin Specifically Interacts with the Filamin A Mechanosensitive Domain 21. *Biochemistry* **2019**.
430. Hachimi, M.; Grabowski, C.; Campanario, S.; Herranz, G.; Baonza, G.; Serrador, J. M.; Gomez-Lopez, S.; Barea, M. D.; Bosch-Fortea, M.; Gilmour, D.; Bagnat, M.; Rodriguez-Fraticelli, A. E.; Martin-Belmonte, F., Smoothelin-like 2 Inhibits Coronin-1B to Stabilize the Apical Actin Cortex during Epithelial Morphogenesis. *Current biology : CB* **2021,** 31, (4).
431. Butler, M. H.; David, C.; Ochoa, G. C.; Freyberg, Z.; Daniell, L.; Grabs, D.; Cremona, O.; De Camilli, P., Amphiphysin II (SH3P9; BIN1), a member of the amphiphysin/Rvs family, is concentrated in the cortical cytomatrix of axon initial segments and nodes of ranvier in brain and around T tubules in skeletal muscle. *The Journal of cell biology* **1997,** 137, (6), 1355-1367.
432. Dräger, N. M.; Nachman, E.; Winterhoff, M.; Brühmann, S.; Shah, P.; Katsinelos, T.; Boulant, S.; Teleman, A. A.; Faix, J.; Jahn, T. R., Bin1 directly remodels actin dynamics through its BAR domain. *EMBO reports* **2017,** 18, (11), 2051-2066.
433. Prokic, I.; Cowling, B. S.; Laporte, J., Amphiphysin 2 (BIN1) in physiology and diseases. *Journal of molecular medicine (Berlin, Germany)* **2014,** 92, (5), 453-463.
434. Rimm, D. L.; Koslov, E. R.; Kebriaei, P.; Cianci, C. D.; Morrow, J. S., Alpha 1(E)-catenin is an actin-binding and -bundling protein mediating the attachment of F-actin to

the membrane adhesion complex. *Proceedings of the National Academy of Sciences of the United States of America* **1995,** 92, (19), 8813-8817.
435. Guilherme, A.; Soriano, N. A.; Bose, S.; Holik, J.; Bose, A.; Pomerleau, D. P.; Furcinitti, P.; Leszyk, J.; Corvera, S.; Czech, M. P., EHD2 and the novel EH domain binding protein EHBP1 couple endocytosis to the actin cytoskeleton. *The Journal of biological chemistry* **2004,** 279, (11), 10593-10605.
436. Rai, A.; Bleimling, N.; Vetter, I. R.; Goody, R. S., The mechanism of activation of the actin binding protein EHBP1 by Rab8 family members. *Nat Commun* **2020,** 11, (1), 4187.
437. Bashour, A. M.; Fullerton, A. T.; Hart, M. J.; Bloom, G. S., IQGAP1, a Rac- and Cdc42-binding protein, directly binds and cross-links microfilaments. *The Journal of cell biology* **1997,** 137, (7), 1555-1566.
438. Fukata, M.; Kuroda, S.; Fujii, K.; Nakamura, T.; Shoji, I.; Matsuura, Y.; Okawa, K.; Iwamatsu, A.; Kikuchi, A.; Kaibuchi, K., Regulation of cross-linking of actin filament by IQGAP1, a target for Cdc42. *The Journal of biological chemistry* **1997,** 272, (47), 29579-29583.
439. White, C. D.; Brown, M. D.; Sacks, D. B., IQGAPs in cancer: a family of scaffold proteins underlying tumorigenesis. *FEBS letters* **2009,** 583, (12), 1817-1824.
440. Watanabe, T.; Wang, S.; Kaibuchi, K., IQGAPs as Key Regulators of Actin-cytoskeleton Dynamics. *Cell Struct Funct* **2015,** 40, (2), 69-77.
441. Brill, S.; Li, S.; Lyman, C. W.; Church, D. M.; Wasmuth, J. J.; Weissbach, L.; Bernards, A.; Snijders, A. J., The Ras GTPase-activating-protein-related human protein IQGAP2 harbors a potential actin binding domain and interacts with calmodulin and Rho family GTPases. *Mol Cell Biol* **1996,** 16, (9), 4869-78.
442. Wang, S.; Watanabe, T.; Noritake, J.; Fukata, M.; Yoshimura, T.; Itoh, N.; Harada, T.; Nakagawa, M.; Matsuura, Y.; Arimura, N.; Kaibuchi, K., IQGAP3, a novel effector of Rac1 and Cdc42, regulates neurite outgrowth. *Journal of cell science* **2007,** 120, (Pt 4), 567-577.
443. Brandt, D. T.; Grosse, R., Get to grips: steering local actin dynamics with IQGAPs. *EMBO reports* **2007,** 8, (11), 1019-1023.
444. Lin, X.; Ruiz, J.; Bajraktari, I.; Ohman, R.; Banerjee, S.; Gribble, K.; Kaufman, J. D.; Wingfield, P. T.; Griggs, R. C.; Fischbeck, K. H.; Mankodi, A., Z-disc-associated, alternatively spliced, PDZ motif-containing protein (ZASP) mutations in the actin-binding domain cause disruption of skeletal muscle actin filaments in myofibrillar myopathy. *The Journal of biological chemistry* **2014,** 289, (19), 13615-13626.
445. Watts, N. R.; Zhuang, X.; Kaufman, J. D.; Palmer, I. W.; Dearborn, A. D.; Coscia, S.; Blech-Hermoni, Y.; Alfano, C.; Pastore, A.; Mankodi, A.; Wingfield, P. T., Expression and Purification of ZASP Subdomains and Clinically Important Isoforms: High-Affinity Binding to G-Actin. *Biochemistry* **2017,** 56, (14), 2061-2070.
446. Wadmore, K.; Azad, A. J.; Gehmlich, K., The Role of Z-disc Proteins in Myopathy and Cardiomyopathy. *International journal of molecular sciences* **2021,** 22, (6).
447. Miralles, F.; Posern, G.; Zaromytidou, A. I.; Treisman, R., Actin dynamics control SRF activity by regulation of its coactivator MAL. *Cell* **2003,** 113, (3), 329-42.
448. Mattila, P. K.; Salminen, M.; Yamashiro, T.; Lappalainen, P., Mouse MIM, a tissue-specific regulator of cytoskeletal dynamics, interacts with ATP-actin monomers through its C-terminal WH2 domain. *J Biol Chem* **2003,** 278, (10), 8452-9.

449. Woodings, J. A.; Sharp, S. J.; Machesky, L. M., MIM-B, a putative metastasis suppressor protein, binds to actin and to protein tyrosine phosphatase delta. *The Biochemical journal* **2003,** 371, (Pt 2), 463-471.
450. Loberg, R. D.; Neeley, C. K.; Adam-Day, L. L.; Fridman, Y.; St John, L. N.; Nixdorf, S.; Jackson, P.; Kalikin, L. M.; Pienta, K. J., Differential expression analysis of MIM (MTSS1) splice variants and a functional role of MIM in prostate cancer cell biology. *International journal of oncology* **2005,** 26, (6), 1699-1705.
451. Fukuda, M.; Kuroda, T. S., Slac2-c (synaptotagmin-like protein homologue lacking C2 domains-c), a novel linker protein that interacts with Rab27, myosin Va/VIIa, and actin. *The Journal of biological chemistry* **2002,** 277, (45), 43096-43103.
452. El-Amraoui, A.; Schonn, J. S.; Kussel-Andermann, P.; Blanchard, S.; Desnos, C.; Henry, J. P.; Wolfrum, U.; Darchen, F.; Petit, C., MyRIP, a novel Rab effector, enables myosin VIIa recruitment to retinal melanosomes. *EMBO Rep* **2002,** 3, (5), 463-70.
453. Kuroda, T. S.; Fukuda, M., Identification and biochemical analysis of Slac2-c/MyRIP as a Rab27A-, myosin Va/VIIa-, and actin-binding protein. *Methods in enzymology* **2005,** 403, 431-444.
454. Jin, J. P.; Wang, K., Nebulin as a giant actin-binding template protein in skeletal muscle sarcomere. Interaction of actin and cloned human nebulin fragments. *FEBS letters* **1991,** 281, (1-2), 93-96.
455. Chen, M. J.; Shih, C. L.; Wang, K., Nebulin as an actin zipper. A two-module nebulin fragment promotes actin nucleation and stabilizes actin filaments. *J Biol Chem* **1993,** 268, (27), 20327-34.
456. Pappas, C. T.; Bliss, K. T.; Zieseniss, A.; Gregorio, C. C., The Nebulin family: an actin support group. *Trends in cell biology* **2011,** 21, (1), 29-37.
457. Labeit, S.; Ottenheijm, C. A. C.; Granzier, H., Nebulin, a major player in muscle health and disease. *FASEB journal : official publication of the Federation of American Societies for Experimental Biology* **2011,** 25, (3), 822-829.
458. Luo, G.; Zhang, J. Q.; Nguyen, T. P.; Herrera, A. H.; Paterson, B.; Horowits, R., Complete cDNA sequence and tissue localization of N-RAP, a novel nebulin-related protein of striated muscle. *Cell motility and the cytoskeleton* **1997,** 38, (1), 75-90.
459. Sefton, B. M.; Hunter, T.; Ball, E. H.; Singer, S. J., Vinculin: a cytoskeletal target of the transforming protein of Rous sarcoma virus. *Cell* **1981,** 24, (1), 165-74.
460. Jockusch, B. M.; Isenberg, G., Interaction of alpha-actinin and vinculin with actin: opposite effects on filament network formation. *Proc Natl Acad Sci U S A* **1981,** 78, (5), 3005-9.
461. Zemljic-Harpf, A.; Manso, A. M.; Ross, R. S., Vinculin and talin: focus on the myocardium. *J Investig Med* **2009,** 57, (8), 849-55.
462. Golji, J.; Mofrad, M. R. K., The interaction of vinculin with actin. *PLoS computational biology* **2013,** 9, (4), e1002995.
463. Arber, S.; Caroni, P., Specificity of single LIM motifs in targeting and LIM/LIM interactions in situ. *Genes & development* **1996,** 10, (3), 289-300.
464. Levin, E.; Leibinger, M.; Gobrecht, P.; Hilla, A.; Andreadaki, A.; Fischer, D., Muscle LIM Protein Is Expressed in the Injured Adult CNS and Promotes Axon Regeneration. *Cell reports* **2019,** 26, (4).

465. Hoffmann, C.; Moreau, F.; Moes, M.; Luthold, C.; Dieterle, M.; Goretti, E.; Neumann, K.; Steinmetz, A.; Thomas, C., Human muscle LIM protein dimerizes along the actin cytoskeleton and cross-links actin filaments. *Mol Cell Biol* **2014,** 34, (16), 3053-65.
466. Kamakura, K.; Tadano, Y.; Kawai, M.; Ishiura, S.; Nakamura, R.; Miyamoto, K.; Nagata, N.; Sugita, H., Dystrophin-related protein is found in the central nervous system of mice at various developmental stages, especially at the postsynaptic membrane. *Journal of neuroscience research* **1994,** 37, (6), 728-734.
467. Winder, S. J.; Hemmings, L.; Maciver, S. K.; Bolton, S. J.; Tinsley, J. M.; Davies, K. E.; Critchley, D. R.; Kendrick-Jones, J., Utrophin actin binding domain: analysis of actin binding and cellular targeting. *Journal of cell science* **1995,** 108 (Pt 1), 63-71.
468. She, B.-R.; Liou, G.-G.; Lin-Chao, S., Association of the growth-arrest-specific protein Gas7 with F-actin induces reorganization of microfilaments and promotes membrane outgrowth. *Experimental cell research* **2002,** 273, (1), 34-44.
469. Lécuyer, C.; Dacheux, J. L.; Hermand, E.; Mazeman, E.; Rousseaux, J.; Rousseaux-Prévost, R., Actin-binding properties and colocalization with actin during spermiogenesis of mammalian sperm calicin. *Biology of reproduction* **2000,** 63, (6), 1801-1810.
470. Xu, J.; Tsutsumi, K.; Tokuraku, K.; Estes, K. A.; Hisanaga, S.-i.; Ikezu, T., Actin interaction and regulation of cyclin-dependent kinase 5/p35 complex activity. *Journal of neurochemistry* **2011,** 116, (2), 192-204.
471. Zhang, B.; Cao, Q.; Guo, A.; Chu, H.; Chan, Y. G.; Buschdorf, J. P.; Low, B. C.; Ling, E. A.; Liang, F., Juxtanodin: an oligodendroglial protein that promotes cellular arborization and 2',3'-cyclic nucleotide-3'-phosphodiesterase trafficking. *Proc Natl Acad Sci U S A* **2005,** 102, (32), 11527-32.
472. Brockschnieder, D.; Sabanay, H.; Riethmacher, D.; Peles, E., Ermin, a myelinating oligodendrocyte-specific protein that regulates cell morphology. *The Journal of neuroscience : the official journal of the Society for Neuroscience* **2006,** 26, (3), 757-762.
473. Ruskamo, S.; Chukhlieb, M.; Vahokoski, J.; Bhargav, S. P.; Liang, F.; Kursula, I.; Kursula, P., Juxtanodin is an intrinsically disordered F-actin-binding protein. *Scientific reports* **2012,** 2, 899.
474. Viklund, I.-M.; Aspenström, P.; Meas-Yedid, V.; Zhang, B.; Kopec, J.; Agren, D.; Schneider, G.; D'Amato, M.; Olivo-Marin, J.-C.; Sansonetti, P.; Van Nhieu, G. T.; Pettersson, S., WAFL, a new protein involved in regulation of early endocytic transport at the intersection of actin and microtubule dynamics. *Experimental cell research* **2009,** 315, (6), 1040-1052.
475. Brancolini, C.; Bottega, S.; Schneider, C., Gas2, a growth arrest-specific protein, is a component of the microfilament network system. *The Journal of cell biology* **1992,** 117, (6), 1251-1261.
476. Zhang, N.; Zhao, C.; Zhang, X.; Cui, X.; Zhao, Y.; Yang, J.; Gao, X., Growth arrest-specific 2 protein family: Structure and function. *Cell proliferation* **2021,** 54, (1), e12934.
477. Goriounov, D.; Leung, C. L.; Liem, R. K. H., Protein products of human Gas2-related genes on chromosomes 17 and 22 (hGAR17 and hGAR22) associate with both microfilaments and microtubules. *Journal of cell science* **2003,** 116, (Pt 6), 1045-1058.
478. Stroud, M. J.; Kammerer, R. A.; Ballestrem, C., Characterization of G2L3 (GAS2-like 3), a new microtubule- and actin-binding protein related to spectraplakins. *The Journal of biological chemistry* **2011,** 286, (28), 24987-24995.

479. Soltysik-Espanola, M.; Rogers, R. A.; Jiang, S.; Kim, T. A.; Gaedigk, R.; White, R. A.; Avraham, H.; Avraham, S., Characterization of Mayven, a novel actin-binding protein predominantly expressed in brain. *Molecular biology of the cell* **1999,** 10, (7), 2361-2375.
480. Hara, T.; Ishida, H.; Raziuddin, R.; Dorkhom, S.; Kamijo, K.; Miki, T., Novel kelch-like protein, KLEIP, is involved in actin assembly at cell-cell contact sites of Madin-Darby canine kidney cells. *Molecular biology of the cell* **2004,** 15, (3), 1172-1184.
481. Kim, I. F.; Mohammadi, E.; Huang, R. C., Isolation and characterization of IPP, a novel human gene encoding an actin-binding, kelch-like protein. *Gene* **1999,** 228, (1-2), 73-83.
482. Govindaraj, V.; Yaduvanshi, N. S.; Krishnamachar, H.; Rao, A. J., Expression of thyroid-stimulating hormone receptor, octamer-binding transcription factor 4, and intracisternal A particle-promoted polypeptide in human breast cancer tissues. *Horm Mol Biol Clin Investig* **2012,** 9, (3), 173-178.
483. Inokuchi, J.; Komiya, M.; Baba, I.; Naito, S.; Sasazuki, T.; Shirasawa, S., Deregulated expression of KRAP, a novel gene encoding actin-interacting protein, in human colon cancer cells. *Journal of human genetics* **2004,** 49, (1), 46-52.
484. Fujimoto, T.; Shirasawa, S., KRAS-induced actin-interacting protein: a potent target for obesity, diabetes and cancer. *Anticancer research* **2011,** 31, (7), 2413-2417.
485. Fauchereau, F.; Herbrand, U.; Chafey, P.; Eberth, A.; Koulakoff, A.; Vinet, M.-C.; Ahmadian, M. R.; Chelly, J.; Billuart, P., The RhoGAP activity of OPHN1, a new F-actin-binding protein, is negatively controlled by its amino-terminal domain. *Molecular and cellular neurosciences* **2003,** 23, (4), 574-586.
486. Bergmann, C.; Zerres, K.; Senderek, J.; Rudnik-Schoneborn, S.; Eggermann, T.; Häusler, M.; Mull, M.; Ramaekers, V. T., Oligophrenin 1 (OPHN1) gene mutation causes syndromic X-linked mental retardation with epilepsy, rostral ventricular enlargement and cerebellar hypoplasia. *Brain : a journal of neurology* **2003,** 126, (Pt 7), 1537-1544.
487. Perisic, L.; Lal, M.; Hulkko, J.; Hultenby, K.; Önfelt, B.; Sun, Y.; Dunér, F.; Patrakka, J.; Betsholtz, C.; Uhlen, M.; Brismar, H.; Tryggvason, K.; Wernerson, A.; Pikkarainen, T., Plekhh2, a novel podocyte protein downregulated in human focal segmental glomerulosclerosis, is involved in matrix adhesion and actin dynamics. *Kidney international* **2012,** 82, (10), 1071-1083.
488. Rider, L.; Tao, J.; Snyder, S.; Brinley, B.; Lu, J.; Diakonova, M., Adapter protein SH2B1beta cross-links actin filaments and regulates actin cytoskeleton. *Molecular endocrinology (Baltimore, Md.)* **2009,** 23, (7), 1065-1076.
489. Cheng, Y.; Duan, C.; Zhang, C., New perspective on SH2B1: An accelerator of cancer progression. *Biomedicine & pharmacotherapy = Biomedecine & pharmacotherapie* **2020,** 121, 109651.
490. Glenney, J., Two related but distinct forms of the Mr 36,000 tyrosine kinase substrate (calpactin) that interact with phospholipid and actin in a Ca2+-dependent manner. *Proc Natl Acad Sci U S A* **1986,** 83, (12), 4258-62.
491. Glenney, J. R.; Tack, B.; Powell, M. A., Calpactins: two distinct Ca++-regulated phospholipid- and actin-binding proteins isolated from lung and placenta. *The Journal of cell biology* **1987,** 104, (3), 503-511.
492. Geisow, M. J.; Walker, J. H.; Boustead, C.; Taylor, W., Annexins--new family of Ca2+-regulated-phospholipid binding protein. *Biosci Rep* **1987,** 7, (4), 289-98.
493. Hayes, M. J.; Rescher, U.; Gerke, V.; Moss, S. E., Annexin-actin interactions. *Traffic (Copenhagen, Denmark)* **2004,** 5, (8), 571-576.

494. de Jong, R.; Leoni, G.; Drechsler, M.; Soehnlein, O., The advantageous role of annexin A1 in cardiovascular disease. *Cell adhesion & migration* **2017,** 11, (3), 261-274.
495. Patel, D. M.; Ahmad, S. F.; Weiss, D. G.; Gerke, V.; Kuznetsov, S. A., Annexin A1 is a new functional linker between actin filaments and phagosomes during phagocytosis. *Journal of cell science* **2011,** 124, (Pt 4), 578-588.
496. Purvis, G. S. D.; Solito, E.; Thiemermann, C., Annexin-A1: Therapeutic Potential in Microvascular Disease. *Frontiers in immunology* **2019,** 10, 938.
497. Fu, Z.; Zhang, S.; Wang, B.; Huang, W.; Zheng, L.; Cheng, A., Annexin A1: A double-edged sword as novel cancer biomarker. *Clinica chimica acta; international journal of clinical chemistry* **2020,** 504, 36-42.
498. Gerke, V.; Weber, K., Identity of p36K phosphorylated upon Rous sarcoma virus transformation with a protein purified from brush borders; calcium-dependent binding to non-erythroid spectrin and F-actin. *The EMBO journal* **1984,** 3, (1), 227-233.
499. Hayes, M. J.; Shao, D.; Bailly, M.; Moss, S. E., Regulation of actin dynamics by annexin 2. *The EMBO journal* **2006,** 25, (9), 1816-1826.
500. Dallacasagrande, V.; Hajjar, K. A., Annexin A2 in Inflammation and Host Defense. *Cells* **2020,** 9, (6).
501. Pepinsky, R. B.; Tizard, R.; Mattaliano, R. J.; Sinclair, L. K.; Miller, G. T.; Browning, J. L.; Chow, E. P.; Burne, C.; Huang, K. S.; Pratt, D.; et al., Five distinct calcium and phospholipid binding proteins share homology with lipocortin I. *J Biol Chem* **1988,** 263, (22), 10799-811.
502. Tzima, E.; Trotter, P. J.; Orchard, M. A.; Walker, J. H., Annexin V relocates to the platelet cytoskeleton upon activation and binds to a specific isoform of actin. *European journal of biochemistry* **2000,** 267, (15), 4720-4730.
503. Peng, B.; Guo, C.; Guan, H.; Liu, S.; Sun, M.-Z., Annexin A5 as a potential marker in tumors. *Clinica chimica acta; international journal of clinical chemistry* **2014,** 427, 42-48.
504. Kobayashi, R.; Tashima, Y., Purification, biological properties and partial sequence analysis of 67-kDa calcimedin and its 34-kDa fragment from chicken gizzard. *European journal of biochemistry* **1990,** 188, (2), 447-453.
505. Strzelecka-Kiliszek, A.; Buszewska, M. E.; Podszywalow-Bartnicka, P.; Pikula, S.; Otulak, K.; Buchet, R.; Bandorowicz-Pikula, J., Calcium- and pH-dependent localization of annexin A6 isoforms in Balb/3T3 fibroblasts reflecting their potential participation in vesicular transport. *J Cell Biochem* **2008,** 104, (2), 418-34.
506. Goebeler, V.; Ruhe, D.; Gerke, V.; Rescher, U., Annexin A8 displays unique phospholipid and F-actin binding properties. *FEBS Lett* **2006,** 580, (10), 2430-4.
507. Enomoto, A.; Murakami, H.; Asai, N.; Morone, N.; Watanabe, T.; Kawai, K.; Murakumo, Y.; Usukura, J.; Kaibuchi, K.; Takahashi, M., Akt/PKB regulates actin organization and cell motility via Girdin/APE. *Dev Cell* **2005,** 9, (3), 389-402.
508. Gu, F.; Wang, L.; He, J.; Liu, X.; Zhang, H.; Li, W.; Fu, L.; Ma, Y., Girdin, an actin-binding protein, is critical for migration, adhesion, and invasion of human glioblastoma cells. *Journal of neurochemistry* **2014,** 131, (4), 457-469.
509. Jongstra-Bilen, J.; Janmey, P. A.; Hartwig, J. H.; Galea, S.; Jongstra, J., The lymphocyte-specific protein LSP1 binds to F-actin and to the cytoskeleton through its COOH-terminal basic domain. *The Journal of cell biology* **1992,** 118, (6), 1443-1453.

510. Schäringer, K.; Maxeiner, S.; Schalla, C.; Rütten, S.; Zenke, M.; Sechi, A., LSP1-myosin1e bimolecular complex regulates focal adhesion dynamics and cell migration. *FASEB journal : official publication of the Federation of American Societies for Experimental Biology* **2021,** 35, (2), e21268.
511. Hung, R. J.; Yazdani, U.; Yoon, J.; Wu, H.; Yang, T.; Gupta, N.; Huang, Z.; van Berkel, W. J.; Terman, J. R., Mical links semaphorins to F-actin disassembly. *Nature* **2010,** 463, (7282), 823-7.
512. Giridharan, S. S.; Rohn, J. L.; Naslavsky, N.; Caplan, S., Differential regulation of actin microfilaments by human MICAL proteins. *J Cell Sci* **2012,** 125, (Pt 3), 614-24.
513. Alto, L. T.; Terman, J. R., MICALs. *Current biology : CB* **2018,** 28, (9), R538-R541.
514. Suzuki, T.; Nakamoto, T.; Ogawa, S.; Seo, S.; Matsumura, T.; Tachibana, K.; Morimoto, C.; Hirai, H., MICAL, a novel CasL interacting molecule, associates with vimentin. *The Journal of biological chemistry* **2002,** 277, (17), 14933-14941.
515. Terman, J. R.; Mao, T.; Pasterkamp, R. J.; Yu, H.-H.; Kolodkin, A. L., MICALs, a family of conserved flavoprotein oxidoreductases, function in plexin-mediated axonal repulsion. *Cell* **2002,** 109, (7), 887-900.
516. Lee, B. C.; Péterfi, Z.; Hoffmann, F. W.; Moore, R. E.; Kaya, A.; Avanesov, A.; Tarrago, L.; Zhou, Y.; Weerapana, E.; Fomenko, D. E.; Hoffmann, P. R.; Gladyshev, V. N., MsrB1 and MICALs regulate actin assembly and macrophage function via reversible stereoselective methionine oxidation. *Molecular cell* **2013,** 51, (3), 397-404.
517. Bai, J.; Wioland, H.; Advedissian, T.; Cuvelier, F.; Romet-Lemonne, G.; Echard, A., Actin reduction by MsrB2 is a key component of the cytokinetic abscission checkpoint and prevents tetraploidy. *Proc Natl Acad Sci U S A* **2020,** 117, (8), 4169-4179.
518. Drazic, A.; Aksnes, H.; Marie, M.; Boczkowska, M.; Varland, S.; Timmerman, E.; Foyn, H.; Glomnes, N.; Rebowski, G.; Impens, F.; Gevaert, K.; Dominguez, R.; Arnesen, T., NAA80 is actin's N-terminal acetyltransferase and regulates cytoskeleton assembly and cell motility. *Proc Natl Acad Sci U S A* **2018,** 115, (17), 4399-4404.
519. Goris, M.; Magin, R. S.; Foyn, H.; Myklebust, L. M.; Varland, S.; Ree, R.; Drazic, A.; Bhambra, P.; Stove, S. I.; Baumann, M.; Haug, B. E.; Marmorstein, R.; Arnesen, T., Structural determinants and cellular environment define processed actin as the sole substrate of the N-terminal acetyltransferase NAA80. *Proc Natl Acad Sci U S A* **2018,** 115, (17), 4405-4410.
520. Rebowski, G.; Boczkowska, M.; Drazic, A.; Ree, R.; Goris, M.; Arnesen, T.; Dominguez, R., Mechanism of actin N-terminal acetylation. *Science advances* **2020,** 6, (15), eaay8793.
521. Koufaris, C.; Kirmizis, A., N-Terminal Acetyltransferases Are Cancer-Essential Genes Prevalently Upregulated in Tumours. *Cancers* **2020,** 12, (9).
522. Akiyama, K.; Kimura, H., Isolation of a new actin-binding protein from human seminal plasma. *Biochim Biophys Acta* **1990,** 1040, (2), 206-10.
523. Schenkels, L. C.; Schaller, J.; Walgreen-Weterings, E.; Schadee-Eestermans, I. L.; Veerman, E. C.; Nieuw Amerongen, A. V., Identity of human extra parotid glycoprotein (EP-GP) with secretory actin binding protein (SABP) and its biological properties. *Biological chemistry Hoppe-Seyler* **1994,** 375, (9), 609-615.
524. Seifert, G. J.; Lawson, D.; Wiche, G., Immunolocalization of the intermediate filament-associated protein plectin at focal contacts and actin stress fibers. *European journal of cell biology* **1992,** 59, (1), 138-147.

525. Fontao, L.; Geerts, D.; Kuikman, I.; Koster, J.; Kramer, D.; Sonnenberg, A., The interaction of plectin with actin: evidence for cross-linking of actin filaments by dimerization of the actin-binding domain of plectin. *Journal of cell science* **2001,** 114, (Pt 11), 2065-2076.
526. Perez, S. M.; Brinton, L. T.; Kelly, K. A., Plectin in Cancer: From Biomarker to Therapeutic Target. *Cells* **2021,** 10, (9).
527. Ergin, V.; Zheng, S., Putative Coiled-Coil Domain-Dependent Autoinhibition and Alternative Splicing Determine SHTN1's Actin-Binding Activity. *Journal of molecular biology* **2020,** 432, (14), 4154-4166.
528. Winiarczyk, M.; Kaarniranta, K.; Winiarczyk, S.; Adaszek, Ł.; Winiarczyk, D.; Mackiewicz, J., Tear film proteome in age-related macular degeneration. *Graefes Arch Clin Exp Ophthalmol* **2018,** 256, (6), 1127-1139.
529. Shimada, T.; Toriyama, M.; Uemura, K.; Kamiguchi, H.; Sugiura, T.; Watanabe, N.; Inagaki, N., Shootin1 interacts with actin retrograde flow and L1-CAM to promote axon outgrowth. *The Journal of cell biology* **2008,** 181, (5), 817-829.
530. Bratt, A.; Birot, O.; Sinha, I.; Veitonmäki, N.; Aase, K.; Ernkvist, M.; Holmgren, L., Angiomotin regulates endothelial cell-cell junctions and cell motility. *The Journal of biological chemistry* **2005,** 280, (41), 34859-34869.
531. Ernkvist, M.; Aase, K.; Ukomadu, C.; Wohlschlegel, J.; Blackman, R.; Veitonmäki, N.; Bratt, A.; Dutta, A.; Holmgren, L., p130-angiomotin associates to actin and controls endothelial cell shape. *The FEBS journal* **2006,** 273, (9), 2000-2011.
532. Lv, M.; Shen, Y.; Yang, J.; Li, S.; Wang, B.; Chen, Z.; Li, P.; Liu, P.; Yang, J., Angiomotin Family Members: Oncogenes or Tumor Suppressors? *International journal of biological sciences* **2017,** 13, (6), 772-781.
533. Werner, E.; Kowalczyk, A. P.; Faundez, V., Anthrax toxin receptor 1/tumor endothelium marker 8 mediates cell spreading by coupling extracellular ligands to the actin cytoskeleton. *The Journal of biological chemistry* **2006,** 281, (32), 23227-23236.
534. Garlick, K. M.; Batty, S.; Mogridge, J., Binding of filamentous actin to anthrax toxin receptor 1 decreases its association with protective antigen. *Biochemistry* **2012,** 51, (6), 1249-1256.
535. Stránecký, V.; Hoischen, A.; Hartmannová, H.; Zaki, M. S.; Chaudhary, A.; Zudaire, E.; Nosková, L.; Barešová, V.; Přistoupilová, A.; Hodaňová, K.; Sovová, J.; Hůlková, H.; Piherová, L.; Hehir-Kwa, J. Y.; de Silva, D.; Senanayake, M. P.; Farrag, S.; Zeman, J.; Martásek, P.; Baxová, A.; Afifi, H. H.; St Croix, B.; Brunner, H. G.; Temtamy, S.; Kmoch, S., Mutations in ANTXR1 cause GAPO syndrome. *American journal of human genetics* **2013,** 92, (5), 792-799.
536. Chaudhary, A.; Hilton, M. B.; Seaman, S.; Haines, D. C.; Stevenson, S.; Lemotte, P. K.; Tschantz, W. R.; Zhang, X. M.; Saha, S.; Fleming, T.; St Croix, B., TEM8/ANTXR1 blockade inhibits pathological angiogenesis and potentiates tumoricidal responses against multiple cancer types. *Cancer cell* **2012,** 21, (2), 212-226.
537. Tsvetkov, A. S.; Samsonov, A.; Akhmanova, A.; Galjart, N.; Popov, S. V., Microtubule-binding proteins CLASP1 and CLASP2 interact with actin filaments. *Cell motility and the cytoskeleton* **2007,** 64, (7), 519-530.
538. Wang, X.; Zheng, L.; Zeng, Z.; Zhou, G.; Chien, J.; Qian, C.; Vasmatzis, G.; Shridhar, V.; Chen, L.; Liu, W., DIXDC1 isoform, l-DIXDC1, is a novel filamentous actin-binding protein. *Biochemical and biophysical research communications* **2006,** 347, (1), 22-30.

539. Brown, A.; Bernier, G.; Mathieu, M.; Rossant, J.; Kothary, R., The mouse dystonia musculorum gene is a neural isoform of bullous pemphigoid antigen 1. *Nat Genet* **1995,** 10, (3), 301-6.
540. Dalpé, G.; Leclerc, N.; Vallée, A.; Messer, A.; Mathieu, M.; De Repentigny, Y.; Kothary, R., Dystonin is essential for maintaining neuronal cytoskeleton organization. *Molecular and cellular neurosciences* **1998,** 10, (5-6), 243-257.
541. Ferrier, A.; Boyer, J. G.; Kothary, R., Cellular and molecular biology of neuronal dystonin. *International review of cell and molecular biology* **2013,** 300.
542. Liu, Q.; Jones, T. I.; Tang, V. W.; Brieher, W. M.; Jones, P. L., Facioscapulohumeral muscular dystrophy region gene-1 (FRG-1) is an actin-bundling protein associated with muscle-attachment sites. *Journal of cell science* **2010,** 123, (Pt 7), 1116-1123.
543. Ostler, N.; Britzen-Laurent, N.; Liebl, A.; Naschberger, E.; Lochnit, G.; Ostler, M.; Forster, F.; Kunzelmann, P.; Ince, S.; Supper, V.; Praefcke, G. J. K.; Schubert, D. W.; Stockinger, H.; Herrmann, C.; Stürzl, M., Gamma interferon-induced guanylate binding protein 1 is a novel actin cytoskeleton remodeling factor. *Molecular and cellular biology* **2014,** 34, (2), 196-209.
544. de Winter, J. M.; Molenaar, J. P.; Yuen, M.; van der Pijl, R.; Shen, S.; Conijn, S.; van de Locht, M.; Willigenburg, M.; Bogaards, S. J.; van Kleef, E. S.; Lassche, S.; Persson, M.; Rassier, D. E.; Sztal, T. E.; Ruparelia, A. A.; Oorschot, V.; Ramm, G.; Hall, T. E.; Xiong, Z.; Johnson, C. N.; Li, F.; Kiss, B.; Lozano-Vidal, N.; Boon, R. A.; Marabita, M.; Nogara, L.; Blaauw, B.; Rodenburg, R. J.; Küsters, B.; Doorduin, J.; Beggs, A. H.; Granzier, H.; Campbell, K.; Ma, W.; Irving, T.; Malfatti, E.; Romero, N. B.; Bryson-Richardson, R. J.; van Engelen, B. G.; Voermans, N. C.; Ottenheijm, C. A., KBTBD13 is an actin-binding protein that modulates muscle kinetics. *The Journal of clinical investigation* **2020,** 130, (2), 754-767.
545. Wang, J.; Nakamura, F., Identification of Filamin A Mechanobinding Partner II: Fimbacin Is a Novel Actin Cross-Linking and Filamin A Binding Protein. *Biochemistry* **2019**.
546. Bozal-Basterra, L.; Gonzalez-Santamarta, M.; Muratore, V.; Martín-Martín, N.; Ercilla, A.; Rodríguez, J. A.; Carracedo, A.; Sutherland, J. D.; Barrio, R., LUZP1 Controls Cell Division, Migration and Invasion Through Regulation of the Actin Cytoskeleton. *Frontiers in cell and developmental biology* **2021,** 9, 624089.
547. Fujii, T., [Structure and function of mammalian brain microtubule-associated proteins]. *Yakugaku Zasshi* **1994,** 114, (7), 435-47.
548. Pedrotti, B.; Colombo, R.; Islam, K., Microtubule associated protein MAP1A is an actin-binding and crosslinking protein. *Cell Motil Cytoskeleton* **1994,** 29, (2), 110-6.
549. Mohan, R.; John, A., Microtubule-associated proteins as direct crosslinkers of actin filaments and microtubules. *IUBMB Life* **2015,** 67, (6), 395-403.
550. Fujii, T.; Watanabe, M.; Ogoma, Y.; Kondo, Y.; Arai, T., Microtubule-associated proteins, MAP 1A and MAP 1B, interact with F-actin in vitro. *Journal of biochemistry* **1993,** 114, (6), 827-829.
551. Orbán-Németh, Z.; Simader, H.; Badurek, S.; Tranciková, A.; Propst, F., Microtubule-associated protein 1S, a short and ubiquitously expressed member of the microtubule-associated protein 1 family. *The Journal of biological chemistry* **2005,** 280, (3), 2257-2265.

552. Griffith, L. M.; Pollard, T. D., Evidence for actin filament-microtubule interaction mediated by microtubule-associated proteins. *J Cell Biol* **1978,** 78, (3), 958-65.
553. Griffith, L. M.; Pollard, T. D., The interaction of actin filaments with microtubules and microtubule-associated proteins. *The Journal of biological chemistry* **1982,** 257, (15), 9143-9151.
554. Matsushima, K.; Tokuraku, K.; Hasan, M. R.; Kotani, S., Microtubule-associated protein 4 binds to actin filaments and modulates their properties. *Journal of biochemistry* **2012,** 151, (1).
555. Griffith, L. M.; Pollard, T. D., The interaction of actin filaments with microtubules and microtubule-associated proteins. *J Biol Chem* **1982,** 257, (15), 9143-51.
556. Pîrşcoveanu, D. F. V.; Pirici, I.; Tudorică, V.; Bălşeanu, T. A.; Albu, V. C.; Bondari, S.; Bumbea, A. M.; Pîrşcoveanu, M., Tau protein in neurodegenerative diseases - a review. *Romanian journal of morphology and embryology = Revue roumaine de morphologie et embryologie* **2017,** 58, (4), 1141-1150.
557. Papin, S.; Paganetti, P., Emerging Evidences for an Implication of the Neurodegeneration-Associated Protein TAU in Cancer. *Brain sciences* **2020,** 10, (11).
558. Ritter, B.; Modregger, J.; Paulsson, M.; Plomann, M., PACSIN 2, a novel member of the PACSIN family of cytoplasmic adapter proteins. *FEBS letters* **1999,** 454, (3), 356-362.
559. Kostan, J.; Salzer, U.; Orlova, A.; Törö, I.; Hodnik, V.; Senju, Y.; Zou, J.; Schreiner, C.; Steiner, J.; Meriläinen, J.; Nikki, M.; Virtanen, I.; Carugo, O.; Rappsilber, J.; Lappalainen, P.; Lehto, V.-P.; Anderluh, G.; Egelman, E. H.; Djinović-Carugo, K., Direct interaction of actin filaments with F-BAR protein pacsin2. *EMBO reports* **2014,** 15, (11), 1154-1162.
560. Bhalla-Gehi, R.; Penuela, S.; Churko, J. M.; Shao, Q.; Laird, D. W., Pannexin1 and pannexin3 delivery, cell surface dynamics, and cytoskeletal interactions. *The Journal of biological chemistry* **2010,** 285, (12), 9147-9160.
561. Boyce, A. K.; Wicki-Stordeur, L. E.; Swayne, L. A., Powerful partnership: crosstalk between pannexin 1 and the cytoskeleton. *Front Physiol* **2014,** 5, 27.
562. Gay, O.; Gilquin, B.; Pitaval, A.; Baudier, J., Refilins: A link between perinuclear actin bundle dynamics and mechanosensing signaling. *Bioarchitecture* **2011,** 1, (5), 245-249.
563. Gay, O.; Gilquin, B.; Assard, N.; Stuelsatz, P.; Delphin, C.; Lachuer, J.; Gidrol, X.; Baudier, J., Refilins are short-lived Actin-bundling proteins that regulate lamellipodium protrusion dynamics. *Biol Open* **2016,** 5, (10), 1351-1361.
564. Gay, O.; Gilquin, B.; Nakamura, F.; Jenkins, Z. A.; McCartney, R.; Krakow, D.; Deshiere, A.; Assard, N.; Hartwig, J. H.; Robertson, S. P.; Baudier, J., RefilinB (FAM101B) targets filamin A to organize perinuclear actin networks and regulates nuclear shape. *Proc Natl Acad Sci U S A* **2011,** 108, (28), 11464-9.
565. Salomaa, S. I.; Miihkinen, M.; Kremneva, E.; Paatero, I.; Lilja, J.; Jacquemet, G.; Vuorio, J.; Antenucci, L.; Kogan, K.; Hassani Nia, F.; Hollos, P.; Isomursu, A.; Vattulainen, I.; Coffey, E. T.; Kreienkamp, H.-J.; Lappalainen, P.; Ivaska, J., SHANK3 conformation regulates direct actin binding and crosstalk with Rap1 signaling. *Current biology : CB* **2021,** 31, (22).
566. Ebashi, S.; Kodama, A., A new protein factor promoting aggregation of tropomyosin. *J Biochem* **1965,** 58, (1), 107-8.

567. Wade, R.; Eddy, R.; Shows, T. B.; Kedes, L., cDNA sequence, tissue-specific expression, and chromosomal mapping of the human slow-twitch skeletal muscle isoform of troponin I. *Genomics* **1990,** 7, (3), 346-57.
568. Ohtsuki, I., Troponin: structure, function and dysfunction. *Adv Exp Med Biol* **2007,** 592, 21-36.
569. Xing, J.; Chinnaraj, M.; Zhang, Z.; Cheung, H. C.; Dong, W.-J., Structural studies of interactions between cardiac troponin I and actin in regulated thin filament using Förster resonance energy transfer. *Biochemistry* **2008,** 47, (50), 13383-13393.
570. Marston, S.; Zamora, J. E., Troponin structure and function: a view of recent progress. *Journal of muscle research and cell motility* **2020,** 41, (1), 71-89.
571. Katrukha, I. A., Human cardiac troponin complex. Structure and functions. *Biochemistry. Biokhimiia* **2013,** 78, (13), 1447-1465.
572. Ebashi, S.; Wakabayashi, T.; Ebashi, F., Troponin and its components. *J Biochem* **1971,** 69, (2), 441-5.
573. Drabikowski, W.; Dabrowska, R.; Barylko, B., Separation and characterization of the constituents of troponin. *FEBS Lett* **1971,** 12, (3), 148-152.
574. Greaser, M. L.; Gergely, J., Reconstitution of troponin activity from three protein components. *J Biol Chem* **1971,** 246, (13), 4226-33.
575. Murray, A. C.; Kay, C. M., Separation and characterization of the inhibitory factor of the troponin system. *Biochem Biophys Res Commun* **1971,** 44, (1), 237-44.
576. Tiso, N.; Rampoldi, L.; Pallavicini, A.; Zimbello, R.; Pandolfo, D.; Valle, G.; Lanfranchi, G.; Danieli, G. A., Fine mapping of five human skeletal muscle genes: alpha-tropomyosin, beta-tropomyosin, troponin-I slow-twitch, troponin-I fast-twitch, and troponin-C fast. *Biochem Biophys Res Commun* **1997,** 230, (2), 347-50.
577. Bhavsar, P. K.; Brand, N. J.; Yacoub, M. H.; Barton, P. J., Isolation and characterization of the human cardiac troponin I gene (TNNI3). *Genomics* **1996,** 35, (1), 11-23.
578. Wei, B.; Jin, J. P., TNNT1, TNNT2, and TNNT3: Isoform genes, regulation, and structure-function relationships. *Gene* **2016,** 582, (1), 1-13.
579. Samson, F.; Gilbert, J. R.; Koza-Taylor, P.; Speer, M. C.; Lee, J.; Roses, A. D., A PstI polymorphism detected by a genomic clone at the human slow troponin T (TNNT1) gene locus. *Nucleic Acids Res* **1991,** 19, (21), 6058.
580. Perry, S. V.; Cole, H. A., Phosphorylation of the "37000 component" of the troponin complex (troponin-t). *Biochem J* **1973,** 131, (2), 425-8.
581. Franklin, A. J.; Baxley, T.; Kobayashi, T.; Chalovich, J. M., The C-terminus of troponin T is essential for maintaining the inactive state of regulated actin. *Biophysical journal* **2012,** 102, (11), 2536-2544.
582. Mao, C.; Baumgartner, A. P.; Jha, P. K.; Huang, T. H.; Sarkar, S., Assignment of the human fast skeletal troponin T gene (TNNT3) to chromosome 11p15.5: evidence for the presence of 11pter in a monochromosome 9 somatic cell hybrid in NIGMS mapping panel 2. *Genomics* **1996,** 31, (3), 385-8.
583. Bermingham, N.; Hernandez, D.; Balfour, A.; Gilmour, F.; Martin, J. E.; Fisher, E. M., Mapping TNNC1, the gene that encodes cardiac troponin I in the human and the mouse. *Genomics* **1995,** 30, (3), 620-2.
584. Townsend, P. J.; Yacoub, M. H.; Barton, P. J., Assignment of the human fast skeletal muscle troponin C gene (TNNC2) between D20S721 and GCT10F11 on chromosome 20 by somatic cell hybrid analysis. *Ann Hum Genet* **1997,** 61, (Pt 5), 457-9.

585. Wilkins, J. A.; Risinger, M. A.; Lin, S., Studies on proteins that co-purify with smooth muscle vinculin: identification of immunologically related species in focal adhesions of nonmuscle and Z-lines of muscle cells. *J Cell Biol* **1986,** 103, (4), 1483-94.
586. Davis, S.; Lu, M. L.; Lo, S. H.; Lin, S.; Butler, J. A.; Druker, B. J.; Roberts, T. M.; An, Q.; Chen, L. B., Presence of an SH2 domain in the actin-binding protein tensin. *Science* **1991,** 252, (5006), 712-5.
587. Lo, S. H., Tensin. *The international journal of biochemistry & cell biology* **2004,** 36, (1), 31-34.
588. Lo, S. H.; Weisberg, E.; Chen, L. B., Tensin: a potential link between the cytoskeleton and signal transduction. *BioEssays : news and reviews in molecular, cellular and developmental biology* **1994,** 16, (11), 817-823.
589. Liao, Y.-C.; Lo, S. H., Tensins - emerging insights into their domain functions, biological roles and disease relevance. *Journal of cell science* **2021,** 134, (4).
590. Clark, K.; Howe, J. D.; Pullar, C. E.; Green, J. A.; Artym, V. V.; Yamada, K. M.; Critchley, D. R., Tensin 2 modulates cell contractility in 3D collagen gels through the RhoGAP DLC1. *Journal of cellular biochemistry* **2010,** 109, (4), 808-817.
591. Cui, Y.; Liao, Y.-C.; Lo, S. H., Epidermal growth factor modulates tyrosine phosphorylation of a novel tensin family member, tensin3. *Molecular cancer research : MCR* **2004,** 2, (4), 225-232.
592. Maruyama, K., Connectin, an elastic protein from myofibrils. *J Biochem* **1976,** 80, (2), 405-7.
593. Brown, S. S.; Malinoff, H. L.; Wicha, M. S., Connectin: cell surface protein that binds both laminin and actin. *Proceedings of the National Academy of Sciences of the United States of America* **1983,** 80, (19), 5927-5930.
594. Adewale, A. O.; Ahn, Y.-H., Titin N2A Domain and Its Interactions at the Sarcomere. *International journal of molecular sciences* **2021,** 22, (14).
595. LeWinter, M. M.; Wu, Y.; Labeit, S.; Granzier, H., Cardiac titin: structure, functions and role in disease. *Clinica chimica acta; international journal of clinical chemistry* **2007,** 375, (1-2), 1-9.
596. Ottenheijm, C. A. C.; Granzier, H., Role of titin in skeletal muscle function and disease. *Advances in experimental medicine and biology* **2010,** 682, 105-122.
597. Cai, G.; Wu, D.; Wang, Z.; Xu, Z.; Wong, K. B.; Ng, C. F.; Chan, F. L.; Yu, S., Collapsin response mediator protein-1 (CRMP1) acts as an invasion and metastasis suppressor of prostate cancer via its suppression of epithelial-mesenchymal transition and remodeling of actin cytoskeleton organization. *Oncogene* **2017,** 36, (4), 546-558.
598. Arimura, N.; Ménager, C.; Kawano, Y.; Yoshimura, T.; Kawabata, S.; Hattori, A.; Fukata, Y.; Amano, M.; Goshima, Y.; Inagaki, M.; Morone, N.; Usukura, J.; Kaibuchi, K., Phosphorylation by Rho kinase regulates CRMP-2 activity in growth cones. *Molecular and cellular biology* **2005,** 25, (22), 9973-9984.
599. Rosslenbroich, V.; Dai, L.; Baader, S. L.; Noegel, A. A.; Gieselmann, V.; Kappler, J., Collapsin response mediator protein-4 regulates F-actin bundling. *Experimental cell research* **2005,** 310, (2), 434-444.
600. Cha, C.; Zhang, J.; Ji, Z.; Tan, M.; Li, S.; Wu, F.; Chen, K.; Gong, S.; Guo, G.; Lin, H., CRMP4 regulates dendritic growth and maturation via the interaction with actin cytoskeleton in cultured hippocampal neurons. *Brain research bulletin* **2016,** 124, 286-294.

601. Gong, X.; Tan, M.; Gao, Y.; Chen, K.; Guo, G., CRMP-5 interacts with actin to regulate neurite outgrowth. *Molecular medicine reports* **2016,** 13, (2), 1179-1185.
602. Hoffmann, C.; Mao, X.; Dieterle, M.; Moreau, F.; Al Absi, A.; Steinmetz, A.; Oudin, A.; Berchem, G.; Janji, B.; Thomas, C., CRP2, a new invadopodia actin bundling factor critically promotes breast cancer cell invasion and metastasis. *Oncotarget* **2016,** 7, (12), 13688-13705.
603. den Hartigh, J. C.; van Bergen en Henegouwen, P. M.; Verkleij, A. J.; Boonstra, J., The EGF receptor is an actin-binding protein. *The Journal of cell biology* **1992,** 119, (2), 349-355.
604. Song, W.; Wu, J.; Ge, G.; Lin, Q., Two domains of the epidermal growth factor receptor are involved in cytoskeletal interactions. *Biochemical and biophysical research communications* **2008,** 370, (4), 589-593.
605. Bae, J.; Sung, B. H.; Cho, I. H.; Kim, S.-M.; Song, W. K., NESH regulates dendritic spine morphology and synapse formation. *PloS one* **2012,** 7, (4), e34677.
606. Bae, J.; Sung, B. H.; Cho, I. H.; Song, W. K., F-actin-dependent regulation of NESH dynamics in rat hippocampal neurons. *PloS one* **2012,** 7, (4), e34514.
607. Haase, H.; Pagel, I.; Khalina, Y.; Zacharzowsky, U.; Person, V.; Lutsch, G.; Petzhold, D.; Kott, M.; Schaper, J.; Morano, I., The carboxyl-terminal ahnak domain induces actin bundling and stabilizes muscle contraction. *FASEB journal : official publication of the Federation of American Societies for Experimental Biology* **2004,** 18, (7), 839-841.
608. Marie, H.; Pratt, S. J.; Betson, M.; Epple, H.; Kittler, J. T.; Meek, L.; Moss, S. J.; Troyanovsky, S.; Attwell, D.; Longmore, G. D.; Braga, V. M., The LIM protein Ajuba is recruited to cadherin-dependent cell junctions through an association with alpha-catenin. *J Biol Chem* **2003,** 278, (2), 1220-8.
609. McCormack, J. J.; Bruche, S.; Ouadda, A. B. D.; Ishii, H.; Lu, H.; Garcia-Cattaneo, A.; Chávez-Olórtegui, C.; Lamarche-Vane, N.; Braga, V. M. M., The scaffold protein Ajuba suppresses CdGAP activity in epithelia to maintain stable cell-cell contacts. *Scientific reports* **2017,** 7, (1), 9249.
610. Schleicher, K.; Schramek, D., AJUBA: A regulator of epidermal homeostasis and cancer. *Exp Dermatol* **2021,** 30, (4), 546-559.
611. Diring, J.; Mouilleron, S.; McDonald, N. Q.; Treisman, R., RPEL-family rhoGAPs link Rac/Cdc42 GTP loading to G-actin availability. *Nature cell biology* **2019,** 21, (7), 845-855.
612. Mitin, N.; Rossman, K. L.; Der, C. J., Identification of a novel actin-binding domain within the Rho guanine nucleotide exchange factor TEM4. *PloS one* **2012,** 7, (7), e41876.
613. Ke, H.; Parron, V. I.; Reece, J.; Zhang, J. Y.; Akiyama, S. K.; French, J. E., BCL2 inhibits cell adhesion, spreading, and motility by enhancing actin polymerization. *Cell research* **2010,** 20, (4), 458-469.
614. Shen, K.; Teruel, M. N.; Subramanian, K.; Meyer, T., CaMKIIbeta functions as an F-actin targeting module that localizes CaMKIIalpha/beta heterooligomers to dendritic spines. *Neuron* **1998,** 21, (3), 593-606.
615. Borgesius, N. Z.; van Woerden, G. M.; Buitendijk, G. H. S.; Keijzer, N.; Jaarsma, D.; Hoogenraad, C. C.; Elgersma, Y., βCaMKII plays a nonenzymatic role in hippocampal synaptic plasticity and learning by targeting αCaMKII to synapses. *The Journal of neuroscience : the official journal of the Society for Neuroscience* **2011,** 31, (28), 10141-10148.

616. Neirynck, K.; Waterschoot, D.; Vandekerckhove, J.; Ampe, C.; Rommelaere, H., Actin interacts with CCT via discrete binding sites: a binding transition-release model for CCT-mediated actin folding. *Journal of molecular biology* **2006,** 355, (1), 124-138.
617. Hansen, W. J.; Cowan, N. J.; Welch, W. J., Prefoldin-nascent chain complexes in the folding of cytoskeletal proteins. *The Journal of cell biology* **1999,** 145, (2), 265-277.
618. Rommelaere, H.; De Neve, M.; Neirynck, K.; Peelaers, D.; Waterschoot, D.; Goethals, M.; Fraeyman, N.; Vandekerckhove, J.; Ampe, C., Prefoldin recognition motifs in the nonhomologous proteins of the actin and tubulin families. *The Journal of biological chemistry* **2001,** 276, (44), 41023-41028.
619. Lu, R.; Niesen, M. J.; Hu, W.; Vaidehi, N.; Shively, J. E., Interaction of actin with carcinoembryonic antigen-related cell adhesion molecule 1 (CEACAM1) receptor in liposomes is Ca2+- and phospholipid-dependent. *J Biol Chem* **2011,** 286, (31), 27528-36.
620. D'Atri, F.; Citi, S., Cingulin interacts with F-actin in vitro. *FEBS letters* **2001,** 507, (1), 21-24.
621. Bennardini, F.; Wrzosek, A.; Chiesi, M., Alpha B-crystallin in cardiac tissue. Association with actin and desmin filaments. *Circ Res* **1992,** 71, (2), 288-94.
622. Wang, X.; Osinska, H.; Klevitsky, R.; Gerdes, A. M.; Nieman, M.; Lorenz, J.; Hewett, T.; Robbins, J., Expression of R120G-alphaB-crystallin causes aberrant desmin and alphaB-crystallin aggregation and cardiomyopathy in mice. *Circulation research* **2001,** 89, (1), 84-91.
623. Wojtowicz, I.; Jablonska, J.; Zmojdzian, M.; Taghli-Lamallem, O.; Renaud, Y.; Junion, G.; Daczewska, M.; Huelsmann, S.; Jagla, K.; Jagla, T., Drosophila small heat shock protein CryAB ensures structural integrity of developing muscles, and proper muscle and heart performance. *Development* **2015,** 142, (5), 994-1005.
624. Yin, B.; Tang, S.; Xu, J.; Sun, J.; Zhang, X.; Li, Y.; Bao, E., CRYAB protects cardiomyocytes against heat stress by preventing caspase-mediated apoptosis and reducing F-actin aggregation. *Cell stress & chaperones* **2019,** 24, (1), 59-68.
625. Marat, A. L.; Ioannou, M. S.; McPherson, P. S., Connecdenn 3/DENND1C binds actin linking Rab35 activation to the actin cytoskeleton. *Molecular biology of the cell* **2012,** 23, (1), 163-175.
626. Zhang, S.; Buder, K.; Burkhardt, C.; Schlott, B.; Görlach, M.; Grosse, F., Nuclear DNA helicase II/RNA helicase A binds to filamentous actin. *The Journal of biological chemistry* **2002,** 277, (1), 843-853.
627. Zhang, S.; Köhler, C.; Hemmerich, P.; Grosse, F., Nuclear DNA helicase II (RNA helicase A) binds to an F-actin containing shell that surrounds the nucleolus. *Experimental cell research* **2004,** 293, (2), 248-258.
628. Lee, T.; Pelletier, J., The biology of DHX9 and its potential as a therapeutic target. *Oncotarget* **2016,** 7, (27), 42716-42739.
629. Magri, E.; Zaccarini, M.; Grazi, E., The interaction of histone and protamine with actin. Possible involvement in the formation of the mitotic spindle. *Biochemical and biophysical research communications* **1978,** 82, (4), 1207-1210.
630. Doyle, A.; Crosby, S. R.; Burton, D. R.; Lilley, F.; Murphy, M. F., Actin bundling and polymerisation properties of eukaryotic elongation factor 1 alpha (eEF1A), histone H2A-H2B and lysozyme in vitro. *Journal of structural biology* **2011,** 176, (3), 370-378.
631. Blotnick, E.; Sol, A.; Muhlrad, A., Histones bundle F-actin filaments and affect actin structure. *PloS one* **2017,** 12, (8), e0183760.

632. Lazarides, E.; Lindberg, U., Actin is the naturally occurring inhibitor of deoxyribonuclease I. *Proc Natl Acad Sci U S A* **1974,** 71, (12), 4742-6.
633. Kabsch, W.; Mannherz, H. G.; Suck, D.; Pai, E. F.; Holmes, K. C., Atomic structure of the actin:DNase I complex. *Nature* **1990,** 347, (6288), 37-44.
634. Gu, C.; Yaddanapudi, S.; Weins, A.; Osborn, T.; Reiser, J.; Pollak, M.; Hartwig, J.; Sever, S., Direct dynamin-actin interactions regulate the actin cytoskeleton. *The EMBO journal* **2010,** 29, (21), 3593-3606.
635. Mooren, O. L.; Kotova, T. I.; Moore, A. J.; Schafer, D. A., Dynamin2 GTPase and cortactin remodel actin filaments. *The Journal of biological chemistry* **2009,** 284, (36), 23995-24005.
636. Menon, M.; Askinazi, O. L.; Schafer, D. A., Dynamin2 organizes lamellipodial actin networks to orchestrate lamellar actomyosin. *PloS one* **2014,** 9, (4), e94330.
637. Liu, G.; Grant, W. M.; Persky, D.; Latham, V. M., Jr.; Singer, R. H.; Condeelis, J., Interactions of elongation factor 1alpha with F-actin and beta-actin mRNA: implications for anchoring mRNA in cell protrusions. *Mol Biol Cell* **2002,** 13, (2), 579-92.
638. Owen, C. H.; DeRosier, D. J.; Condeelis, J., Actin crosslinking protein EF-1a of Dictyostelium discoideum has a unique bonding rule that allows square-packed bundles. *J Struct Biol* **1992,** 109, (3), 248-54.
639. Abbas, W.; Kumar, A.; Herbein, G., The eEF1A Proteins: At the Crossroads of Oncogenesis, Apoptosis, and Viral Infections. *Front Oncol* **2015,** 5, 75.
640. Novosylna, O.; Doyle, A.; Vlasenko, D.; Murphy, M.; Negrutskii, B.; El'skaya, A., Comparison of the ability of mammalian eEF1A1 and its oncogenic variant eEF1A2 to interact with actin and calmodulin. *Biological chemistry* **2017,** 398, (1), 113-124.
641. Bektas, M.; Nurten, R.; Gurel, Z.; Sayers, Z.; Bermek, E., Interactions of eukaryotic elongation factor 2 with actin: a possible link between protein synthetic machinery and cytoskeleton. *FEBS Lett* **1994,** 356, (1), 89-93.
642. Bektas, M.; Guncer, B.; Guven, C.; Nurten, R.; Bermek, E., Actin--an inhibitor of eukaryotic elongation factor activities. *Biochem Biophys Res Commun* **2004,** 317, (4), 1061-6.
643. Slater, P. G.; Cammarata, G. M.; Samuelson, A. G.; Magee, A.; Hu, Y.; Lowery, L. A., XMAP215 promotes microtubule-F-actin interactions to regulate growth cone microtubules during axon guidance in. *Journal of cell science* **2019,** 132, (9).
644. Holaska, J. M.; Kowalski, A. K.; Wilson, K. L., Emerin caps the pointed end of actin filaments: evidence for an actin cortical network at the nuclear inner membrane. *PLoS biology* **2004,** 2, (9), E231.
645. Teixeira, F. R.; Yokoo, S.; Gartner, C. A.; Manfiolli, A. O.; Baqui, M. M. A.; Assmann, E. M.; Maragno, A. L. G. C.; Yu, H.; de Lanerolle, P.; Kobarg, J.; Gygi, S. P.; Gomes, M. D., Identification of FBXO25-interacting proteins using an integrated proteomics approach. *Proteomics* **2010,** 10, (15), 2746-2757.
646. Obaishi, H.; Nakanishi, H.; Mandai, K.; Satoh, K.; Satoh, A.; Takahashi, K.; Miyahara, M.; Nishioka, H.; Takaishi, K.; Takai, Y., Frabin, a novel FGD1-related actin filament-binding protein capable of changing cell shape and activating c-Jun N-terminal kinase. *The Journal of biological chemistry* **1998,** 273, (30), 18697-18700.
647. Langhorst, M. F.; Solis, G. P.; Hannbeck, S.; Plattner, H.; Stuermer, C. A. O., Linking membrane microdomains to the cytoskeleton: regulation of the lateral mobility of reggie-1/flotillin-2 by interaction with actin. *FEBS letters* **2007,** 581, (24), 4697-4703.

648. Van Baelen, H.; Bouillon, R.; De Moor, P., Vitamin D-binding protein (Gc-globulin) binds actin. *The Journal of biological chemistry* **1980,** 255, (6), 2270-2272.
649. Bouillon, R.; Schuit, F.; Antonio, L.; Rastinejad, F., Vitamin D Binding Protein: A Historic Overview. *Frontiers in endocrinology* **2019,** 10, 910.
650. Wu, T.; Mu, Y.; Bogomolovas, J.; Fang, X.; Veevers, J.; Nowak, R. B.; Pappas, C. T.; Gregorio, C. C.; Evans, S. M.; Fowler, V. M.; Chen, J., HSPB7 is indispensable for heart development by modulating actin filament assembly. *Proceedings of the National Academy of Sciences of the United States of America* **2017,** 114, (45), 11956-11961.
651. Sattlegger, E.; Barbosa, J. A. R. G.; Moraes, M. C. S.; Martins, R. M.; Hinnebusch, A. G.; Castilho, B. A., Gcn1 and actin binding to Yih1: implications for activation of the eIF2 kinase GCN2. *The Journal of biological chemistry* **2011,** 286, (12), 10341-10355.
652. Pereira, C. M.; Sattlegger, E.; Jiang, H. Y.; Longo, B. M.; Jaqueta, C. B.; Hinnebusch, A. G.; Wek, R. C.; Mello, L. E.; Castilho, B. A., IMPACT, a protein preferentially expressed in the mouse brain, binds GCN1 and inhibits GCN2 activation. *J Biol Chem* **2005,** 280, (31), 28316-23.
653. Landino, J.; Ohi, R., The Timing of Midzone Stabilization during Cytokinesis Depends on Myosin II Activity and an Interaction between INCENP and Actin. *Current biology : CB* **2016,** 26, (5), 698-706.
654. Knoll, K. R.; Eustermann, S.; Niebauer, V.; Oberbeckmann, E.; Stoehr, G.; Schall, K.; Tosi, A.; Schwarz, M.; Buchfellner, A.; Korber, P.; Hopfner, K.-P., The nuclear actin-containing Arp8 module is a linker DNA sensor driving INO80 chromatin remodeling. *Nature structural & molecular biology* **2018,** 25, (9), 823-832.
655. Cao, T.; Sun, L.; Jiang, Y.; Huang, S.; Wang, J.; Chen, Z., Crystal structure of a nuclear actin ternary complex. *Proc Natl Acad Sci U S A* **2016,** 113, (32), 8985-90.
656. Bledzka, K.; Bialkowska, K.; Sossey-Alaoui, K.; Vaynberg, J.; Pluskota, E.; Qin, J.; Plow, E. F., Kindlin-2 directly binds actin and regulates integrin outside-in signaling. *The Journal of cell biology* **2016,** 213, (1).
657. Johnson, H. W.; Schell, M. J., Neuronal IP3 3-kinase is an F-actin-bundling protein: role in dendritic targeting and regulation of spine morphology. *Mol Biol Cell* **2009,** 20, (24), 5166-80.
658. Windhorst, S.; Song, K.; Gazdar, A. F., Inositol-1,4,5-trisphosphate 3-kinase-A (ITPKA) is frequently over-expressed and functions as an oncogene in several tumor types. *Biochemical pharmacology* **2017,** 137, 1-9.
659. Zou, S.; Jha, S.; Kim, E. Y.; Dryer, S. E., A novel actin-binding domain on Slo1 calcium-activated potassium channels is necessary for their expression in the plasma membrane. *Mol Pharmacol* **2008,** 73, (2), 359-68.
660. Zusev, M.; Benayahu, D., New insights on cellular distribution, microtubule interactions and post-translational modifications of MS-KIF18A. *Journal of cellular physiology* **2008,** 217, (3), 618-625.
661. Visser-Grieve, S.; Zhou, Z.; She, Y.-M.; Huang, H.; Cyr, T. D.; Xu, T.; Yang, X., LATS1 tumor suppressor is a novel actin-binding protein and negative regulator of actin polymerization. *Cell research* **2011,** 21, (10), 1513-1516.
662. Takeuchi, T.; Heng, H. H. Q.; Ye, C. J.; Liang, S.-B.; Iwata, J.; Sonobe, H.; Ohtsuki, Y., Down-regulation of a novel actin-binding molecule, skeletrophin, in malignant melanoma. *The American journal of pathology* **2003,** 163, (4), 1395-1404.

663. Moos, C.; Mason, C. M.; Besterman, J. M.; Feng, I. N.; Dubin, J. H., The binding of skeletal muscle C-protein to F-actin, and its relation to the interaction of actin with myosin subfragment-1. *J Mol Biol* **1978,** 124, (4), 571-86.
664. Burghardt, T. P., Demographic model for inheritable cardiac disease. *Archives of biochemistry and biophysics* **2019,** 672, 108056.
665. Sato, K.; Handa, H.; Kimura, M.; Okano, Y.; Nagaoka, H.; Nagase, T.; Sugiyama, T.; Kitade, Y.; Ueda, H., Identification of a Rho family specific guanine nucleotide exchange factor, FLJ00018, as a novel actin-binding protein. *Cellular signalling* **2013,** 25, (1), 41-49.
666. Torti, M.; Bertoni, A.; Canobbio, I.; Sinigaglia, F.; Lapetina, E. G.; Balduini, C., Interaction of the low-molecular-weight GTP-binding protein rap2 with the platelet cytoskeleton is mediated by direct binding to the actin filaments. *Journal of cellular biochemistry* **1999,** 75, (4), 675-685.
667. MacDonald, J. I. S.; Dietrich, A.; Gamble, S.; Hryciw, T.; Grant, R. I.; Meakin, S. O., Nesca, a novel neuronal adapter protein, links the molecular motor kinesin with the pre-synaptic membrane protein, syntaxin-1, in hippocampal neurons. *Journal of neurochemistry* **2012,** 121, (6), 861-880.
668. Kim, D. J.; Kim, S. H.; Kim, S. M.; Bae, J. I.; Ahnn, J.; Song, W. K., F-actin binding region of SPIN90 C-terminus is essential for actin polymerization and lamellipodia formation. *Cell Commun Adhes* **2007,** 14, (1), 33-43.
669. Iwata, Y.; Sampaolesi, M.; Shigekawa, M.; Wakabayashi, S., Syntrophin is an actin-binding protein the cellular localization of which is regulated through cytoskeletal reorganization in skeletal muscle cells. *European journal of cell biology* **2004,** 83, (10), 555-565.
670. Bhat, H. F.; Adams, M. E.; Khanday, F. A., Syntrophin proteins as Santa Claus: role(s) in cell signal transduction. *Cellular and molecular life sciences : CMLS* **2013,** 70, (14), 2533-2554.
671. Ali, R.; Mir, H. A.; Hamid, R.; Shah, R. A.; Khanday, F. A.; Bhat, S. S., Jasplakinolide Attenuates Cell Migration by Impeding Alpha-1-syntrophin Protein Phosphorylation in Breast Cancer Cells. *Protein J* **2021,** 40, (2), 234-244.
672. Buechler, C.; Boettcher, A.; Bared, S. M.; Probst, M. C. O.; Schmitz, G., The carboxyterminus of the ATP-binding cassette transporter A1 interacts with a beta2-syntrophin/utrophin complex. *Biochemical and biophysical research communications* **2002,** 293, (2), 759-765.
673. Kirchhof, M. G.; Chau, L. A.; Lemke, C. D.; Vardhana, S.; Darlington, P. J.; Márquez, M. E.; Taylor, R.; Rizkalla, K.; Blanca, I.; Dustin, M. L.; Madrenas, J., Modulation of T cell activation by stomatin-like protein 2. *Journal of immunology (Baltimore, Md. : 1950)* **2008,** 181, (3), 1927-1936.
674. Coutts, A. S.; MacKenzie, E.; Griffith, E.; Black, D. M., TES is a novel focal adhesion protein with a role in cell spreading. *Journal of cell science* **2003,** 116, (Pt 5), 897-906.
675. Rotter, B.; Bournier, O.; Nicolas, G.; Dhermy, D.; Lecomte, M.-C., AlphaII-spectrin interacts with Tes and EVL, two actin-binding proteins located at cell contacts. *The Biochemical journal* **2005,** 388, (Pt 2), 631-638.
676. Xi, Q.; Pauer, G. J. T.; Marmorstein, A. D.; Crabb, J. W.; Hagstrom, S. A., Tubby-like protein 1 (TULP1) interacts with F-actin in photoreceptor cells. *Investigative ophthalmology & visual science* **2005,** 46, (12), 4754-4761.

677. Peng, J.-M.; Hsieh, S.-Y.; Cheng, J.-H.; Luo, J.-W.; Su, Y.-L.; Luo, H.-L., Confirming whether KLHL23 deficiency potentiates migration in urothelial carcinoma. *The Chinese journal of physiology* **2021,** 64, (3), 142-149.
678. Peng, J.-M.; Bera, R.; Chiou, C.-Y.; Yu, M.-C.; Chen, T.-C.; Chen, C.-W.; Wang, T.-R.; Chiang, W.-L.; Chai, S.-P.; Wei, Y.; Wang, H.; Hung, M.-C.; Hsieh, S.-Y., Actin cytoskeleton remodeling drives epithelial-mesenchymal transition for hepatoma invasion and metastasis in mice. *Hepatology (Baltimore, Md.)* **2018,** 67, (6), 2226-2243.
679. Kang, M.-I.; Kobayashi, A.; Wakabayashi, N.; Kim, S.-G.; Yamamoto, M., Scaffolding of Keap1 to the actin cytoskeleton controls the function of Nrf2 as key regulator of cytoprotective phase 2 genes. *Proceedings of the National Academy of Sciences of the United States of America* **2004,** 101, (7), 2046-2051.
680. Wu, B.; Yang, S.; Sun, H.; Sun, T.; Ji, F.; Wang, Y.; Xu, L.; Zhou, D., Keap1 Inhibits Metastatic Properties of NSCLC Cells by Stabilizing Architectures of F-Actin and Focal Adhesions. *Molecular cancer research : MCR* **2018,** 16, (3), 508-516.
681. Itoh, K.; Wakabayashi, N.; Katoh, Y.; Ishii, T.; Igarashi, K.; Engel, J. D.; Yamamoto, M., Keap1 represses nuclear activation of antioxidant responsive elements by Nrf2 through binding to the amino-terminal Neh2 domain. *Genes & development* **1999,** 13, (1), 76-86.
682. Taguchi, K.; Yamamoto, M., The KEAP1-NRF2 System in Cancer. *Frontiers in oncology* **2017,** 7, 85.
683. Hernandez, M. C.; Andres-Barquin, P. J.; Martinez, S.; Bulfone, A.; Rubenstein, J. L.; Israel, M. A., ENC-1: a novel mammalian kelch-related gene specifically expressed in the nervous system encodes an actin-binding protein. *The Journal of neuroscience : the official journal of the Society for Neuroscience* **1997,** 17, (9), 3038-3051.
684. Zhao, L.; Gregoire, F.; Sul, H. S., Transient induction of ENC-1, a Kelch-related actin-binding protein, is required for adipocyte differentiation. *The Journal of biological chemistry* **2000,** 275, (22), 16845-16850.
685. Watanabe, Y.; Usada, N.; Minami, H.; Morita, T.; Tsugane, S.; Ishikawa, R.; Kohama, K.; Tomida, Y.; Hidaka, H., Calvasculin, as a factor affecting the microfilament assemblies in rat fibroblasts transfected by src gene. *FEBS letters* **1993,** 324, (1), 51-55.
686. Jurewicz, E.; Robaszkiewicz, K.; Moraczewska, J.; Filipek, A., Binding of S100A6 to actin and the actin-tropomyosin complex. *Scientific reports* **2020,** 10, (1), 12824.
687. Schreiber, J.; Végh, M. J.; Dawitz, J.; Kroon, T.; Loos, M.; Labonté, D.; Li, K. W.; Van Nierop, P.; Van Diepen, M. T.; De Zeeuw, C. I.; Kneussel, M.; Meredith, R. M.; Smit, A. B.; Van Kesteren, R. E., Ubiquitin ligase TRIM3 controls hippocampal plasticity and learning by regulating synaptic γ-actin levels. *The Journal of cell biology* **2015,** 211, (3), 569-586.
688. Dolat, L.; Hunyara, J. L.; Bowen, J. R.; Karasmanis, E. P.; Elgawly, M.; Galkin, V. E.; Spiliotis, E. T., Septins promote stress fiber-mediated maturation of focal adhesions and renal epithelial motility. *J Cell Biol* **2014,** 207, (2), 225-35.
689. Smith, C.; Dolat, L.; Angelis, D.; Forgacs, E.; Spiliotis, E. T.; Galkin, V. E., Septin 9 Exhibits Polymorphic Binding to F-Actin and Inhibits Myosin and Cofilin Activity. *J Mol Biol* **2015,** 427, (20), 3273-3284.
690. Sun, J.; Zheng, M.-Y.; Li, Y.-W.; Zhang, S.-W., Structure and function of Septin 9 and its role in human malignant tumors. *World J Gastrointest Oncol* **2020,** 12, (6), 619-631.
691. Shuster, C. B.; Lin, A. Y.; Nayak, R.; Herman, I. M., Beta cap73: a novel beta actin-specific binding protein. *Cell motility and the cytoskeleton* **1996,** 35, (3), 175-187.

692. Welch, A. Y.; Herman, I. M., Cloning and characterization of betaCAP73, a novel regulator of beta-actin assembly. *The international journal of biochemistry & cell biology* **2002,** 34, (7), 864-881.
693. Menice, C. B.; Hulvershorn, J.; Adam, L. P.; Wang, C. A.; Morgan, K. G., Calponin and mitogen-activated protein kinase signaling in differentiated vascular smooth muscle. *The Journal of biological chemistry* **1997,** 272, (40), 25157-25161.
694. Leinweber, B. D.; Leavis, P. C.; Grabarek, Z.; Wang, C. L.; Morgan, K. G., Extracellular regulated kinase (ERK) interaction with actin and the calponin homology (CH) domain of actin-binding proteins. *The Biochemical journal* **1999,** 344 Pt 1, 117-123.
695. Boëda, B.; El-Amraoui, A.; Bahloul, A.; Goodyear, R.; Daviet, L.; Blanchard, S.; Perfettini, I.; Fath, K. R.; Shorte, S.; Reiners, J.; Houdusse, A.; Legrain, P.; Wolfrum, U.; Richardson, G.; Petit, C., Myosin VIIa, harmonin and cadherin 23, three Usher I gene products that cooperate to shape the sensory hair cell bundle. *The EMBO journal* **2002,** 21, (24), 6689-6699.
696. Michalski, N.; Michel, V.; Caberlotto, E.; Lefevre, G. M.; van Aken, A. F.; Tinevez, J. Y.; Bizard, E.; Houbron, C.; Weil, D.; Hardelin, J. P.; Richardson, G. P.; Kros, C. J.; Martin, P.; Petit, C., Harmonin-b, an actin-binding scaffold protein, is involved in the adaptation of mechanoelectrical transduction by sensory hair cells. *Pflugers Arch* **2009,** 459, (1), 115-30.
697. Wagner, O.; Zinke, J.; Dancker, P.; Grill, W.; Bereiter-Hahn, J., Viscoelastic properties of f-actin, microtubules, f-actin/alpha-actinin, and f-actin/hexokinase determined in microliter volumes with a novel nondestructive method. *Biophysical journal* **1999,** 76, (5), 2784-2796.
698. Schneider, G. B.; Hamano, H.; Cooper, L. F., In vivo evaluation of hsp27 as an inhibitor of actin polymerization: hsp27 limits actin stress fiber and focal adhesion formation after heat shock. *J Cell Physiol* **1998,** 177, (4), 575-84.
699. Clarke, J. P.; Mearow, K. M., Cell stress promotes the association of phosphorylated HspB1 with F-actin. *PloS one* **2013,** 8, (7), e68978.
700. Barylko, B.; Dobrowolski, Z., Ca2+-calmodulin-dependent regulation of F-actin-myelin basic protein interaction. *Eur J Cell Biol* **1984,** 35, (2), 327-35.
701. Boggs, J. M.; Rangaraj, G., Interaction of lipid-bound myelin basic protein with actin filaments and calmodulin. *Biochemistry* **2000,** 39, (26), 7799-7806.
702. Mornet, D.; Bonet-Kerrache, A., Neurocalcin-actin interaction. *Biochimica et biophysica acta* **2001,** 1549, (2), 197-203.
703. Ivings, L.; Pennington, S. R.; Jenkins, R.; Weiss, J. L.; Burgoyne, R. D., Identification of Ca2+-dependent binding partners for the neuronal calcium sensor protein neurocalcin delta: interaction with actin, clathrin and tubulin. *The Biochemical journal* **2002,** 363, (Pt 3), 599-608.
704. Cau, J.; Faure, S.; Comps, M.; Delsert, C.; Morin, N., A novel p21-activated kinase binds the actin and microtubule networks and induces microtubule stabilization. *The Journal of cell biology* **2001,** 155, (6), 1029-1042.
705. Tamura, M.; Kai, T.; Tsunawaki, S.; Lambeth, J. D.; Kameda, K., Direct interaction of actin with p47(phox) of neutrophil NADPH oxidase. *Biochemical and biophysical research communications* **2000,** 276, (3), 1186-1190.

706. Metcalfe, S.; Weeds, A.; Okorokov, A. L.; Milner, J.; Cockman, M.; Pope, B., Wild-type p53 protein shows calcium-dependent binding to F-actin. *Oncogene* **1999,** 18, (14), 2351-2355.
707. Okorokov, A. L.; Rubbi, C. P.; Metcalfe, S.; Milner, J., The interaction of p53 with the nuclear matrix is mediated by F-actin and modulated by DNA damage. *Oncogene* **2002,** 21, (3), 356-367.
708. Bähler, M.; Greengard, P., Synapsin I bundles F-actin in a phosphorylation-dependent manner. *Nature* **1987,** 326, (6114), 704-707.
709. Valtorta, F.; Ceccaldi, P. E.; Grohovaz, F.; Chieregatti, E.; Fesce, R.; Benfenati, F., Fluorescence approaches to the study of the actin-nucleating and bundling activities of synapsin I. *J Physiol Paris* **1993,** 87, (2), 117-22.
710. Chilcote, T. J.; Siow, Y. L.; Schaeffer, E.; Greengard, P.; Thiel, G., Synapsin IIa bundles actin filaments. *Journal of neurochemistry* **1994,** 63, (4), 1568-1571.
711. Nielander, H. B.; Onofri, F.; Schaeffer, E.; Menegon, A.; Fesce, R.; Valtorta, F.; Greengard, P.; Benfenati, F., Phosphorylation-dependent effects of synapsin IIa on actin polymerization and network formation. *Eur J Neurosci* **1997,** 9, (12), 2712-22.
712. Castresana, J.; Saraste, M., Does Vav bind to F-actin through a CH domain? *FEBS letters* **1995,** 374, (2), 149-151.
713. Kranewitter, W. J.; Gimona, M., N-terminally truncated Vav induces the formation of depolymerization-resistant actin filaments in NIH 3T3 cells. *FEBS letters* **1999,** 455, (1-2), 123-129.
