# Supplementary material for: Actin-Associated Proteins and Small Molecules Targeting the Actin Cytoskeleton
Source: Int J Mol Sci. 2022 Feb 14;23(4):2118. doi: 10.3390/ijms23042118 (PMC8880164; doi:10.3390/ijms23042118)
Supplement: Supplementary file 1 [file ijms-23-02118-s001.zip › Table S2 Chemical molecules and antibodies acting on actin and AAPs.pdf]

Table S2. Chemical molecules and antibodies acting on actin and AAPs.

Name	MW	Target molecule	Target disease and function	Concentration used (IC_{50}) or Kd	Link to pubchem	Reference
Phalloidin	788.9	Actin	Stabilize F-actin.	Kd= 36 nM	https://pubchem.ncbi.nlm.nih.gov/compound/4752	[1-3]
(-)-Doliculide	616.5	Actin	Stabilize F-actin and nucleate actin.	IC_{50}=30~40 nM for growth inhibition of CA46 and PtK2 cells.	https://pubchem.ncbi.nlm.nih.gov/compound/10438804	[4]
Cytochalasins	B: 479.6<br>D: 507.6	Actin	Bind to the barbed end to inhibit polymerization of F-actin.	IC_{50}=0.5~3 μM for cytotoxicity against MCF7 and A549 cell lines.	B: https://pubchem.ncbi.nlm.nih.gov/compound/5311281<br>D: https://pubchem.ncbi.nlm.nih.gov/compound/5458428	[5-8]
Scytophycin	806.1	Actin	Depolymerize F-actin.	IC_{50} =50~90 nM for cytotoxicity against SKOV3 and SKVLB1 cells.	https://pubchem.ncbi.nlm.nih.gov/compound/101168554	[9]
Jasplakinolide	709.7	Actin	Stabilize F-actin and nucleate actin.	Kd= 15 nM. IC_{50}=35 nM for cytotoxicity against PC3 prostatecarcinoma cells.	https://pubchem.ncbi.nlm.nih.gov/compound/9831636	[10, 11]
Swinholide A	1389.9	Actin	Stabilize actin dimers and sever actin filaments.		https://pubchem.ncbi.nlm.nih.gov/compound/25077966	[12, 13]
Cucurbitacin E	556.7	Actin	Inhibit actin depolymerization. Also inhibit tubulin polymerization.	IC_{50} =7~50 nM for cytotoxicity against prostate carcinoma explants.	https://pubchem.ncbi.nlm.nih.gov/compound/5281319	[14, 15]
Misakinolide A/ Bistheonellide A	1337.8	Actin	Swinholide A family.<br>Barbed end capping.	Kd=50 nM	https://pubchem.ncbi.nlm.nih.gov/compound/6441172	[16]
Chondramide A	646.8	Actin	Stabilize F-actin.<br>Block invasion by *Toxoplasma gondii.*	IC_{50} =3~85 nM for cytotoxicity against SKOV3, U-937, and A-498 cells.	https://pubchem.ncbi.nlm.nih.gov/compound/46217451	[17] [18]
Latrunculin A/B	A: 421.6<br>B: 395.5	Actin	Stabilize G-actin and depolymerize F-actin.	IC_{50}=0.5~3 μM for cytotoxicity against MCF7 and A549 cell lines.	A: https://pubchem.ncbi.nlm.nih.gov/compound/445420<br>B: https://pubchem.ncbi.nlm.nih.gov/compound/6436219	[8, 19]
Dihydrohalichondramide	839.0	Actin	Trisoxazole family. Barbed end capping activity to inhibit polymerization.		https://pubchem.ncbi.nlm.nih.gov/compound/10259927	[20]

Jaspisamide A	850.4	Actin	Trisoxazole family. Severing and capping.		C: https://pubchem.ncbi.nlm.nih.gov/compound/6444281	[21, 22]
Kabiramide C	942.1	Actin	Trisoxazole family. Severing and barbed end capping.	Kd<100 nM	https://pubchem.ncbi.nlm.nih.gov/compound/155926072	[21, 22]
Amphidinolide H	562.7	Actin	Stabilize actin filament.	Binding constants to G-actin and F-actin are 0.43 and 0.21 μM	https://pubchem.ncbi.nlm.nih.gov/substance/103557492	[23, 24]
Reidispongiolide A/C	A: 958.3	Actin	Reidispongiolide sphinxolide family. Severing and capping.		A: https://pubchem.ncbi.nlm.nih.gov/compound/5289284	[25, 26]
Sphinxolide	B: 960.2	Actin	Reidispongiolide sphinxolide family. Severing and capping.		B: https://pubchem.ncbi.nlm.nih.gov/compound/46937020	[25]
Bistramide A	705.0	Actin	Inhibit actin polymerization, depolymerize F-actin, binds to G-actin in a 1:1 ratio.	Kd=7.0~9.0nM	https://pubchem.ncbi.nlm.nih.gov/compound/49864366	[27, 28]
Hurghadolide A	1363.8	Actin	Disrupt actin cytoskeleton.	IC_{50}=365 nM for cytotoxicity against HCT-116 cell.	https://pubchem.ncbi.nlm.nih.gov/compound/15957507	[29]
Aplyronine A	1076.4	Actin	Depolymerize F-actin and inhibit the polymerization of actin by forming a 1:1 complex with monomeric actin.	Kd=100 nM. IC_{50} =0.01 nM for cytotoxicity against HeLa S3 cells.	https://pubchem.ncbi.nlm.nih.gov/compound/11840920	[30, 31]
Swinholide I	1405.9	Actin	Stabilize actin dimers and disrupt actin cytoskeleton.	IC_{50}=5.6 nM for cytotoxicity against HCT-116 cell.	https://pubchem.ncbi.nlm.nih.gov/compound/15957506	[29]
Ulapualide A	882.5	Actin	Trisoxazole family. Severing and capping.	Kd<100 nM	B: https://pubchem.ncbi.nlm.nih.gov/compound/6442513	[32]
Rhizopodin	1469.8	Actin	Stabilize actin dimers and inhibit polymerization.		https://pubchem.ncbi.nlm.nih.gov/compound/25198043	[33]
Lobophorolide	766.5	Actin	Stabilize actin dimers and inhibit polymerization.		http://www.chemspider.com/Chemical-Structure.8503287.html	[34]
Halichondramide	837.0	Actin	Trisoxazole family. Severing and capping.	Kd<100 nM	https://pubchem.ncbi.nlm.nih.gov/compound/6443267	[35-37]
Mycalolide B	1027.2	Actin	Trisoxazole family. Inhibit polymerization of G-actin and severs F-actin. Suppress proliferation,	IC_{50} =21 nM for cytotoxicity against HeLa S3 cells.	https://pubchem.ncbi.nlm.nih.gov/compound/4267	[31, 38]

			migration, and invasion of breast and ovarian cancer cells.			
Prieurianin and prieurianin acetate	762.8	Actin	Stabilize F-actin by reducing severing and depolymerization.	IC_{50} =4.4 µg/ml for cytotoxicity against P388 cells.	https://pubchem.ncbi.nlm.nih.gov/compound/329486	[39]
Violaceol-I and -I	524.5	Actin	Inhibit polymerization. Induce F-actin aggregation.	Kd=14.4 and 2.52 nM	https://pubchem.ncbi.nlm.nih.gov/compound/156023402	[40]
seco-clavilactone B	314.3	Actin	Inhibit polymerization.	~ 10 µM	https://pubchem.ncbi.nlm.nih.gov/compound/132917657	[41]
Honokiol	266.3	Actin	Anticancer	EC_{50}(FRET) = 44 µM	https://pubchem.ncbi.nlm.nih.gov/compound/72303	[42-44]
Fluphenazine	437.5	Actin	Antipsychotic	EC_{50}(FRET) = 25 µM	https://pubchem.ncbi.nlm.nih.gov/compound/3372	[42-44]
Phenothiazine	199.3	Actin	Antipsychotic	EC_{50}(FRET) = 7 µM	https://pubchem.ncbi.nlm.nih.gov/compound/7108	[42-44]
Tegaserod	301.4	Actin	Irritable bowel syndrome	EC_{50}(FRET) = 29 µM	https://pubchem.ncbi.nlm.nih.gov/compound/135409453	[42-44]
Carvedilol	406.5	Actin	β Blocker, treat high blood pressure.	EC_{50}(FRET) = 48 µM	https://pubchem.ncbi.nlm.nih.gov/compound/2585	[42-44]
Mitoxantrone	444.5	Actin	Multiple sclerosis	EC_{50}(FRET) = 5.4 µM	https://pubchem.ncbi.nlm.nih.gov/compound/4212	[42-44]
Thioridazine	370.6	Actin	Antipsychotic	EC_{50}(FRET) = 21 µM	https://pubchem.ncbi.nlm.nih.gov/compound/5452	[42-44]
Flutamide	276.2	Actin	Antiandrogen, anticancer	EC_{50}(FRET) = 15 µM	https://pubchem.ncbi.nlm.nih.gov/compound/3397	[42-44]
Mefloquine	378.3	Actin	Anti-malaria	EC_{50}(FRET) = 21 µM	https://pubchem.ncbi.nlm.nih.gov/compound/4046	[42-44]
Dantrolene	314.3	Actin	Postsynaptic muscle relaxant	EC_{50}(FRET) = 48 µM	https://pubchem.ncbi.nlm.nih.gov/compound/6914273	[42-44]
Chivosazole A	866.1	Actin	Inhibit nucleation, polymerization, and severing of F-actin filaments. Inhibit interaction of actin with gelsolin, profilin, cofilin, and thymosin-beta4.		https://pubchem.ncbi.nlm.nih.gov/compound/21594703	[45]
Raltegravir	444.4	γ-Actin Fascin	Inhibit interaction between aldolase A and γ-actin. Inhibit actin-bundling activity of fascin. FDA-approved inhibitor of human immunodeficiency virus-1.	~100 µM	https://pubchem.ncbi.nlm.nih.gov/compound/54671008	[46, 47]
Pentachloropseudilin (PCIP)	331.4	Myosin I	Selectively inhibit myosin-1c.	1-5 µM	https://pubchem.ncbi.nlm.nih.gov/compound/3053233	[48] [49]
2,3-Butanedione monoxime (BDM)	101.1	Myosin II	ATPase inhibitor of the motor domain of	~5 µM	https://pubchem.ncbi.nlm.nih.gov/compound/6409633	[50, 51]

			skeletal muscle myosin-II.			
N-benzyl-*p*-toluene sulphonamide (BTS)	261.3	Myosin II	Inhibit myosin ATPase and actin-myosin interaction.	~5 µM	https://pubchem.ncbi.nlm.nih.gov/compound/95801	[52, 53]
Blebbistatin and its analogs	292.3	Myosin II	Inhibit motor activity.	0.5–5 µM	https://pubchem.ncbi.nlm.nih.gov/compound/3476986	[54-57]
(S)-3'-hydroxyblebbistatin and (S)-3'-aminoblebbistatin	308.3 and 307.3	Myosin II	Higher water solubility than (S)-blebbistatin. No interference in fluorescence readouts.	14~20 µM	https://pubchem.ncbi.nlm.nih.gov/compound/132850501 https://pubchem.ncbi.nlm.nih.gov/compound/132850502	[58]
Amphidinolide B	562.7	Actomyosin	Increase the ATPase activity of actomyosin. Enhance an interaction of actin and myosin and increase Ca2+ sensitivity of the contractile apparatus.	~10 µM	https://pubchem.ncbi.nlm.nih.gov/compound/101920315	[59]
Thymol	150.2	Myosin subfragment-1	Activate the S1 ATPase.	1.5-2 mM	https://pubchem.ncbi.nlm.nih.gov/compound/6989	[60]
Tachyplegin A-2	317.4	Class XIV unconventional myosin and its associated light chain	Decrease motor activity. Inhibit parasite motility and invasion.	25~ 100 µM	https://pubchem.ncbi.nlm.nih.gov/compound/1899566	[61]
CK-2018448 (CK-448)		Myosin	Inhibit smooth muscle myosin.		https://pubchem.ncbi.nlm.nih.gov/substance/144221115	[62]
CK-2018571		smooth muscle myosin	Inhibit smooth muscle myosin.	IC_{50}=9 nM for the inhibitory effect on smooth muscle myosin.		[63]
MYK-461 (Mavacamten)	273.3	Cardiac myosin heavy chain	Decrease the ATPase activity of the cardiac myosin heavy chain.	IC50=0.3 µM for the inhibitory effect on ATPase activity of mouse cardiac myofibrils.	https://pubchem.ncbi.nlm.nih.gov/compound/117763040 https://pubchem.ncbi.nlm.nih.gov/compound/117761397	[64-66]
Suramin, NF023, and aurintricarboxylic acid	1297.3, 422.3	Cardiac myosin-binding protein C	Inhibit interaction between cardiac myosin-binding protein C and actin.	6~16 µM	https://pubchem.ncbi.nlm.nih.gov/compound/5361 https://pubchem.ncbi.nlm.nih.gov/compound/2259	[67]
Myokinasib	402.5	Myosin light chain kinase 1	Reduce phosphorylated myosin II light chain.	IC_{50}=7.9 µM for inhibition of myosin light chain kinase 1.		[68]
NSC 95397	310.4	S100A4	Disrupt the S100A4/myosin-IIA interaction and inhibit S100A4-	Form covalent adducts with Cys81 and	https://pubchem.ncbi.nlm.nih.gov/compound/262093	[69]

			mediated depolymerization of myosin-IIA filaments.	Cys86 of S100A4.		
TR100<br>ATM-1001<br>ATM-3507	422.6, 611.8	Tropomyosin	Abolish the inhibitory effect of tropomyosin on actin depolymerization.	~50 μM	https://pubchem.ncbi.nlm.nih.gov/compound/99941738<br>https://pubchem.ncbi.nlm.nih.gov/compound/118666864	[70-73]
ORM-3819	397.4	Troponin C	Potent positive inotropic agent.		https://pubchem.ncbi.nlm.nih.gov/compound/136448063	[74]
Levosimendan, (-)-OR-1259, UNII-C6T4514L4E	280.3	Troponin C	Anti-arrhythmia		https://pubchem.ncbi.nlm.nih.gov/compound/3033825	[75]
Golodirsen, antisense oligonucleotide Vyondys 53 Exondys 51 (eteplirsen) Spinraza (nusinersen)<br>SRP-5051.		Dystrophin gene	Treatment of Duchenne muscular dystrophy amenable to exon 53 skipping.		https://pubchem.ncbi.nlm.nih.gov/substance/405226573<br>https://pubchem.ncbi.nlm.nih.gov/substance/187051835<br>https://pubchem.ncbi.nlm.nih.gov/substance/315431340	[76-78]
c-Abl inhibitors (GNF-5 and imatinib	418.4 and 493.6	c-Abl	Treatment of contracted ASM cells with c-Abl inhibitors and β-agonist cooperatively increase actin disassembly.	10 μM	https://pubchem.ncbi.nlm.nih.gov/compound/44129660<br>https://pubchem.ncbi.nlm.nih.gov/compound/5291	[79]
CK-0944636 CK-0993548 (CK-636/548/666/869)	284.4	Arp2/3	Arp2/3 complex inhibitor. Inhibit its ability to nucleate actin filaments.	4 μM	https://pubchem.ncbi.nlm.nih.gov/compound/588963<br>https://pubchem.ncbi.nlm.nih.gov/substance/162248989	[80-83]
CDDO-Im	541.7	Arp2/3	Arp2/3 complex inhibitor.	~ 1 μM	https://pubchem.ncbi.nlm.nih.gov/compound/9958995	[83, 84]
CDDO-Me	505.7	Arp2/3	Arp2/3 complex inhibitor.	~ 1 μM	https://pubchem.ncbi.nlm.nih.gov/compound/400769	[83, 84]
187-1	1784.1	N-WASP	Stabilize the autoinhibited conformation of N-WASP and prevent activation of the Arp2/3 complex.	IC_{50}=~2 μM for inhibition of PIP2-stimulated actin assembly.	https://pubchem.ncbi.nlm.nih.gov/compound/44405676	[85]
Wiskostatin	426.1	N-WASP	Stabilize the autoinhibited conformation of N-WASP.	IC_{50}=~140 μM for inhibition of actin polymerization.	https://pubchem.ncbi.nlm.nih.gov/compound/2775510	[86, 87]
Small molecule compound #13		WASP	Bind WASP and promote its degradation.	Kd=~30.8 nM to WASP.		[88]
Enoxacin	320.3	B2 subunit of vacuolar H(+)-ATPase (V-ATPase)	Block binding between purified B2 and F-actin. Inhibit osteoclast bone resorption.	IC_{50}= 10 μM for inhibition of interaction between B2 and F-actin.	https://pubchem.ncbi.nlm.nih.gov/compound/3229	[89]
Proline-derived modules		Ena/VASP	Reduce extravasation of invasive breast cancer cells in a zebrafish model.	Kd= 0.12 μM. IC_{50}= 0.67 μM for inhibition of the interaction of		[90, 91]

				ENAH EVH1 domain with RAPH1, RIAM, and Zyxin.		
NSC305787 and NSC668394	445. 4 and 452. 1	ERM (Ezrin, Radixin, Moesin)	Inhibit ERM phosphorylation and reduce cell motility to prevent tumor metastasis.	NSC305787: IC_{50}= 8.3, 55, 9.4 μM for inhibition of ezrin, radixin, and moexin phosphorylation by PKCI, respectively. NSC668394: IC_{50}= 8.1, 35.3, 59.5 μM for inhibition of ezrin, radixin, and moexin phosphorylation by PKCI, respectively.	https://pubchem.ncbi.nlm.nih.gov/compound/470998 https://pubchem.ncbi.nlm.nih.gov/compound/381594	[92]
DX-52-1	357. 4	Radixin	Inhibit epithelial cell migration. Disrupt radixin's ability to interact with both actin and the cell adhesion molecule CD44.	IC_{50}= 140 nM for inhibition of wound closure.	https://pubchem.ncbi.nlm.nih.gov/compound/122737	[93]
MMV667492 MMV020549 MMV666069	313. 4, 398. 5, 418. 6	Ezrin	Inhibit ezrin-mediated cell motility of osteosarcoma cells.	Kd= 2~30 μM	https://pubchem.ncbi.nlm.nih.gov/compound/4267688 https://pubchem.ncbi.nlm.nih.gov/compound/20925640 https://pubchem.ncbi.nlm.nih.gov/compound/26325235	[94]
Migrastatin	489. 6	Fascin	Inhibit actin-bundling activity and block tumor cell migration, invasion, and metastasis.	IC_{50}=0.023~29 μM for inhibition of cell migration.	https://pubchem.ncbi.nlm.nih.gov/compound/11016431	[95-98]
G2: N-(1-(4-(trifluoromethyl)benzyl)-1H-indazol-3-yl)furan-2-carboxamide	385. 3	Fascin	Inhibit actin-bundling activity and block tumor cell migration and invasion.	IC_{50}= 5~8 μM for inhibition of actin-bundling function of fascin.	https://pubchem.ncbi.nlm.nih.gov/compound/73331364	[99]
NP-G2-044 NP-G2-029	399. 4	Fascin	Inhibit actin-bundling activity and block tumor metastasis.	IC_{50}=~0.18 μM for inhibition of actin-bundling function of fascin.	https://pubchem.ncbi.nlm.nih.gov/compound/91844684	[98, 100]
BDP-13176	497. 4	Fascin	Disrupt actin binding.	IC_{50}= 0.24 μM for inhibition of actin-bundling function of fascin.	https://pubchem.ncbi.nlm.nih.gov/compound/137333984	[101]

Imipramine	280.4	Fascin	Diminish migration and invasion of cancer cells.	~20 μM	https://pubchem.ncbi.nlm.nih.gov/#query=Imipramine	[102]
Flightless I neutralizing antibodies (FnAb)		Flightless I	Reduce flightless I expression. Enhance wound repair and reduce scar formation.			[103, 104]
Kartogenin	317.3	FLNA	Disrupt interaction between FLNA and transcription factor core-binding factor β subunit (CBFβ), and induce chondrogenesis.	~ 100 nM	https://pubchem.ncbi.nlm.nih.gov/compound/2826191	[105]
PTI-125/Simufilam	259.4	FLNA	Reduce amyloid-related Alzheimer's disease.	~ 1 nM	https://pubchem.ncbi.nlm.nih.gov/compound/46195331	[106-108]
SMIFH2	377.2	Formin homology 2 domains	Prevent both formin nucleation and processive barbed end elongation and decrease formin's affinity for the barbed end.	IC_{50}= 5 to 15 μM for inhibition of morphological change.	https://pubchem.ncbi.nlm.nih.gov/compound/2258538	[109, 110]
Intramimics (IMM-01 and -02)	267.4 and 301.4	mDia	Disrupt the interaction between the DID and DAD autoregulatory domains to activate mDia, triggering actin assembly and microtubule stabilization.	IC_{50}=140 nM (IMM-01) and 99 nM (IMM-02) for inhibition of DID–DAD binding.	https://pubchem.ncbi.nlm.nih.gov/compound/135445526 https://pubchem.ncbi.nlm.nih.gov/compound/136251852	[110, 111]
Nanobodies		L-plastin	Inhibit actin-bundling activity of L-plastin.			[112]
Peptidomimetics of L-plastin		L-plastin	Inhibit bone resorption by osteoclasts but not bone formation by osteoblasts.			[113]
Oroxylin A	284.3	L-plastin	Inhibit actin-bundling activity of L-plastin. Induce osteoporosis and accelerate bone fracture healing in mice.		https://pubchem.ncbi.nlm.nih.gov/compound/5320315	[114]
TAT- MARGSVSDEE, denoted as an inhibitory LPL peptide		L-plastin	Block osteoclast function without impairing the function of osteoblasts.	Subcutaneous injection (~1.5 mg/kg body weight) was given in 36 weeks old		[115]

				mice for 14 weeks.		
compound 1 (C1, 8-(3-hydroxyphenyl)-10-(4-methylphenyl)-2,4,5,6,7,11,12-heptaazatricyclo[7.4.0.$0^{3,7}$]trideca-1(13),3,5,9,11-pentaen-13-ol) and compound 2 (C2, 8-(3-hydroxyphenyl)-10-phenyl-2,4,5,6,7,11,12-heptaazatricyclo[7.4.0.$0^{3,7}$]trideca-1(13),3,5,9,11-pentaen-13-ol))		Profilin 1	Inhibit profilin 1/actin interaction. Slow migration and proliferation of endothelial cells.	~50~100 µM for *in vitro* assay.		[116]
Sanguinarine	332.3	SWAP70	Block phagocytosis and F-actin polymerization.	0.8~2 µM	https://pubchem.ncbi.nlm.nih.gov/compound/5154	[117, 118]

References for Table-S2

1. Lengsfeld, A. M.; Low, I.; Wieland, T.; Dancker, P.; Hasselbach, W., Interaction of phalloidin with actin. *Proc Natl Acad Sci U S A* **1974,** 71, (7), 2803-7.
2. Faulstich, H.; Schafer, A. J.; Weckauf, M., The dissociation of the phalloidin-actin complex. *Hoppe Seylers Z Physiol Chem* **1977,** 358, (2), 181-4.
3. Kumari, A.; Kesarwani, S.; Javoor, M. G.; Vinothkumar, K. R.; Sirajuddin, M., Structural insights into actin filament recognition by commonly used cellular actin markers. *EMBO J* **2020,** 39, (14), e104006.
4. Bai, R.; Covell, D. G.; Liu, C.; Ghosh, A. K.; Hamel, E., (-)-Doliculide, a new macrocyclic depsipeptide enhancer of actin assembly. *J Biol Chem* **2002,** 277, (35), 32165-71.
5. Natori, S., Cytochalasins-actin filament modifiers as a group of mycotoxins. *Dev Toxicol Environ Sci* **1986,** 12, 291-9.
6. Johnson, D. H., The effect of cytochalasin D on outflow facility and the trabecular meshwork of the human eye in perfusion organ culture. *Invest Ophthalmol Vis Sci* **1997,** 38, (13), 2790-9.
7. Salu, K. J.; Huang, Y.; Bosmans, J. M.; Liu, X.; Li, S.; Wang, L.; Verbeken, E.; Bult, H.; Vrints, C. J.; De Scheerder, I. K., Addition of cytochalasin D to a biocompatible oil stent coating inhibits intimal hyperplasia in a porcine coronary model. *Coron Artery Dis* **2003,** 14, (8), 545-55.
8. Hayot, C.; Debeir, O.; Van Ham, P.; Van Damme, M.; Kiss, R.; Decaestecker, C., Characterization of the activities of actin-affecting drugs on tumor cell migration. *Toxicol Appl Pharmacol* **2006,** 211, (1), 30-40.
9. Smith, C. D.; Carmeli, S.; Moore, R. E.; Patterson, G. M., Scytophycins, novel microfilament-depolymerizing agents which circumvent P-glycoprotein-mediated multidrug resistance. *Cancer Res* **1993,** 53, (6), 1343-7.
10. Bubb, M. R.; Senderowicz, A. M.; Sausville, E. A.; Duncan, K. L.; Korn, E. D., Jasplakinolide, a cytotoxic natural product, induces actin polymerization and competitively inhibits the binding of phalloidin to F-actin. *J Biol Chem* **1994,** 269, (21), 14869-71.

11. Pospich, S.; Merino, F.; Raunser, S., Structural Effects and Functional Implications of Phalloidin and Jasplakinolide Binding to Actin Filaments. *Structure* **2020,** 28, (4), 437-449 e5.
12. Bubb, M. R.; Spector, I.; Bershadsky, A. D.; Korn, E. D., Swinholide A is a microfilament disrupting marine toxin that stabilizes actin dimers and severs actin filaments. *J Biol Chem* **1995,** 270, (8), 3463-6.
13. Klenchin, V. A.; King, R.; Tanaka, J.; Marriott, G.; Rayment, I., Structural basis of swinholide A binding to actin. *Chem Biol* **2005,** 12, (3), 287-91.
14. Duncan, K. L.; Duncan, M. D.; Alley, M. C.; Sausville, E. A., Cucurbitacin E-induced disruption of the actin and vimentin cytoskeleton in prostate carcinoma cells. *Biochem Pharmacol* **1996,** 52, (10), 1553-60.
15. Sorensen, P. M.; Iacob, R. E.; Fritzsche, M.; Engen, J. R.; Brieher, W. M.; Charras, G.; Eggert, U. S., The natural product cucurbitacin E inhibits depolymerization of actin filaments. *ACS Chem Biol* **2012,** 7, (9), 1502-8.
16. Terry, D. R.; Spector, I.; Higa, T.; Bubb, M. R., Misakinolide A is a marine macrolide that caps but does not sever filamentous actin. *J Biol Chem* **1997,** 272, (12), 7841-5.
17. Sasse, F.; Kunze, B.; Gronewold, T. M.; Reichenbach, H., The chondramides: cytostatic agents from myxobacteria acting on the actin cytoskeleton. *J Natl Cancer Inst* **1998,** 90, (20), 1559-63.
18. Ma, C. I.; Diraviyam, K.; Maier, M. E.; Sept, D.; Sibley, L. D., Synthetic chondramide A analogues stabilize filamentous actin and block invasion by Toxoplasma gondii. *J Nat Prod* **2013,** 76, (9), 1565-72.
19. Morton, W. M.; Ayscough, K. R.; McLaughlin, P. J., Latrunculin alters the actin-monomer subunit interface to prevent polymerization. *Nat Cell Biol* **2000,** 2, (6), 376-8.
20. Braet, F.; Spector, I.; Shochet, N.; Crews, P.; Higa, T.; Menu, E.; de Zanger, R.; Wisse, E., The new anti-actin agent dihydrohalichondramide reveals fenestrae-forming centers in hepatic endothelial cells. *BMC Cell Biol* **2002,** 3, 7.
21. Klenchin, V. A.; Allingham, J. S.; King, R.; Tanaka, J.; Marriott, G.; Rayment, I., Trisoxazole macrolide toxins mimic the binding of actin-capping proteins to actin. *Nat Struct Biol* **2003,** 10, (12), 1058-63.
22. Tanaka, J.; Yan, Y.; Choi, J.; Bai, J.; Klenchin, V. A.; Rayment, I.; Marriott, G., Biomolecular mimicry in the actin cytoskeleton: mechanisms underlying the cytotoxicity of kabiramide C and related macrolides. *Proc Natl Acad Sci U S A* **2003,** 100, (24), 13851-6.
23. Saito, S. Y.; Feng, J.; Kira, A.; Kobayashi, J.; Ohizumi, Y., Amphidinolide H, a novel type of actin-stabilizing agent isolated from dinoflagellate. *Biochem Biophys Res Commun* **2004,** 320, (3), 961-5.
24. Usui, T.; Kazami, S.; Dohmae, N.; Mashimo, Y.; Kondo, H.; Tsuda, M.; Terasaki, A. G.; Ohashi, K.; Kobayashi, J.; Osada, H., Amphidinolide h, a potent cytotoxic macrolide, covalently binds on actin subdomain 4 and stabilizes actin filament. *Chem Biol* **2004,** 11, (9), 1269-77.
25. Allingham, J. S.; Zampella, A.; D'Auria, M. V.; Rayment, I., Structures of microfilament destabilizing toxins bound to actin provide insight into toxin design and activity. *Proc Natl Acad Sci U S A* **2005,** 102, (41), 14527-32.

26. Pereira, J. H.; Petchprayoon, C.; Hoepker, A. C.; Moriarty, N. W.; Fink, S. J.; Cecere, G.; Paterson, I.; Adams, P. D.; Marriott, G., Structural and biochemical studies of actin in complex with synthetic macrolide tail analogues. *ChemMedChem* **2014,** 9, (10), 2286-93.
27. Statsuk, A. V.; Bai, R.; Baryza, J. L.; Verma, V. A.; Hamel, E.; Wender, P. A.; Kozmin, S. A., Actin is the primary cellular receptor of bistramide A. *Nat Chem Biol* **2005,** 1, (7), 383-8.
28. Rizvi, S. A.; Tereshko, V.; Kossiakoff, A. A.; Kozmin, S. A., Structure of bistramide A-actin complex at a 1.35 angstroms resolution. *J Am Chem Soc* **2006,** 128, (12), 3882-3.
29. Youssef, D. T.; Mooberry, S. L., Hurghadolide A and swinholide I, potent actin-microfilament disrupters from the Red Sea sponge Theonella swinhoei. *J Nat Prod* **2006,** 69, (1), 154-7.
30. Hirata, K.; Muraoka, S.; Suenaga, K.; Kuroda, T.; Kato, K.; Tanaka, H.; Yamamoto, M.; Takata, M.; Yamada, K.; Kigoshi, H., Structure basis for antitumor effect of aplyronine a. *J Mol Biol* **2006,** 356, (4), 945-54.
31. Kita, M.; Yoneda, K.; Hirayama, Y.; Yamagishi, K.; Saito, Y.; Sugiyama, Y.; Miwa, Y.; Ohno, O.; Morita, M.; Suenaga, K.; Kigoshi, H., Fluorescent aplyronine a: intracellular accumulation and disassembly of actin cytoskeleton in tumor cells. *Chembiochem* **2012,** 13, (12), 1754-8, 1702.
32. Vincent, E.; Saxton, J.; Baker-Glenn, C.; Moal, I.; Hirst, J. D.; Pattenden, G.; Shaw, P. E., Effects of ulapualide A and synthetic macrolide analogues on actin dynamics and gene regulation. *Cell Mol Life Sci* **2007,** 64, (4), 487-97.
33. Hagelueken, G.; Albrecht, S. C.; Steinmetz, H.; Jansen, R.; Heinz, D. W.; Kalesse, M.; Schubert, W. D., The absolute configuration of rhizopodin and its inhibition of actin polymerization by dimerization. *Angew Chem Int Ed Engl* **2009,** 48, (3), 595-8.
34. Blain, J. C.; Mok, Y. F.; Kubanek, J.; Allingham, J. S., Two molecules of lobophorolide cooperate to stabilize an actin dimer using both their "ring" and "tail" region. *Chem Biol* **2010,** 17, (8), 802-7.
35. Chung, S. C.; Lee, S. H.; Jang, K. H.; Park, W.; Jeon, J. E.; Oh, H.; Shin, J.; Oh, K. B., Actin depolymerizing effect of trisoxazole-containing macrolides. *Bioorg Med Chem Lett* **2011,** 21, (11), 3198-201.
36. Shin, Y.; Kim, G. D.; Jeon, J. E.; Shin, J.; Lee, S. K., Antimetastatic effect of halichondramide, a trisoxazole macrolide from the marine sponge Chondrosia corticata, on human prostate cancer cells via modulation of epithelial-to-mesenchymal transition. *Mar Drugs* **2013,** 11, (7), 2472-85.
37. Bae, S. Y.; Kim, G. D.; Jeon, J. E.; Shin, J.; Lee, S. K., Anti-proliferative effect of (19Z)-halichondramide, a novel marine macrolide isolated from the sponge Chondrosia corticata, is associated with G2/M cell cycle arrest and suppression of mTOR signaling in human lung cancer cells. *Toxicol In Vitro* **2013,** 27, (2), 694-9.
38. Pipaliya, B. V.; Trofimova, D. N.; Grange, R. L.; Aeluri, M.; Deng, X.; Shah, K.; Craig, A. W.; Allingham, J. S.; Evans, P. A., Truncated Actin-Targeting Macrolide Derivative Blocks Cancer Cell Motility and Invasion of Extracellular Matrix. *J Am Chem Soc* **2021,** 143, (18), 6847-6854.
39. Toth, R.; Gerding-Reimers, C.; Deeks, M. J.; Menninger, S.; Gallegos, R. M.; Tonaco, I. A.; Hubel, K.; Hussey, P. J.; Waldmann, H.; Coupland, G., Prieurianin/endosidin 1 is an actin-stabilizing small molecule identified from a chemical genetic screen for circadian clock effectors in Arabidopsis thaliana. *Plant J* **2012,** 71, (2), 338-52.

40. Asami, Y.; Jang, J. H.; Oh, H.; Sohn, J. H.; Kim, J. W.; Moon, D. O.; Kwon, O.; Kawatani, M.; Osada, H.; Kim, B. Y.; Ahn, J. S., Violaceols function as actin inhibitors inducing cell shape elongation in fibroblast cells. *Biosci Biotechnol Biochem* **2012,** 76, (8), 1431-7.
41. Miyazaki, S.; Sasazawa, Y.; Mogi, T.; Suzuki, T.; Yoshida, K.; Dohmae, N.; Takao, K.; Simizu, S., Identification of seco-clavilactone B as a small-molecule actin polymerization inhibitor. *FEBS Lett* **2016,** 590, (8), 1163-73.
42. Guhathakurta, P.; Prochniewicz, E.; Grant, B. D.; Peterson, K. C.; Thomas, D. D., High-throughput screen, using time-resolved FRET, yields actin-binding compounds that modulate actin-myosin structure and function. *J Biol Chem* **2018,** 293, (31), 12288-12298.
43. Guhathakurta, P.; Phung, L. A.; Prochniewicz, E.; Lichtenberger, S.; Wilson, A.; Thomas, D. D., Actin-binding compounds, previously discovered by FRET-based high-throughput screening, differentially affect skeletal and cardiac muscle. *J Biol Chem* **2020,** 295, (41), 14100-14110.
44. Roopnarine, O.; Thomas, D. D., Mechanistic analysis of actin-binding compounds that affect the kinetics of cardiac myosin-actin interaction. *J Biol Chem* **2021,** 296, 100471.
45. Wang, S.; Gegenfurtner, F. A.; Crevenna, A. H.; Ziegenhain, C.; Kliesmete, Z.; Enard, W.; Muller, R.; Vollmar, A. M.; Schneider, S.; Zahler, S., Chivosazole A Modulates Protein-Protein Interactions of Actin. *J Nat Prod* **2019,** 82, (7), 1961-1970.
46. Chang, Y. C.; Chiou, J.; Yang, Y. F.; Su, C. Y.; Lin, Y. F.; Yang, C. N.; Lu, P. J.; Huang, M. S.; Yang, C. J.; Hsiao, M., Therapeutic Targeting of Aldolase A Interactions Inhibits Lung Cancer Metastasis and Prolongs Survival. *Cancer Res* **2019,** 79, (18), 4754-4766.
47. Alburquerque-Gonzalez, B.; Bernabe-Garcia, A.; Bernabe-Garcia, M.; Ruiz-Sanz, J.; Lopez-Calderon, F. F.; Gonnelli, L.; Banci, L.; Pena-Garcia, J.; Luque, I.; Nicolas, F. J.; Cayuela-Fuentes, M. L.; Luchinat, E.; Perez-Sanchez, H.; Montoro-Garcia, S.; Conesa-Zamora, P., The FDA-Approved Antiviral Raltegravir Inhibits Fascin1-Dependent Invasion of Colorectal Tumor Cells In Vitro and In Vivo. *Cancers (Basel)* **2021,** 13, (4).
48. Chinthalapudi, K.; Taft, M. H.; Martin, R.; Heissler, S. M.; Preller, M.; Hartmann, F. K.; Brandstaetter, H.; Kendrick-Jones, J.; Tsiavaliaris, G.; Gutzeit, H. O.; Fedorov, R.; Buss, F.; Knolker, H. J.; Coluccio, L. M.; Manstein, D. J., Mechanism and specificity of pentachloropseudilin-mediated inhibition of myosin motor activity. *J Biol Chem* **2011,** 286, (34), 29700-8.
49. Bond, L. M.; Tumbarello, D. A.; Kendrick-Jones, J.; Buss, F., Small-molecule inhibitors of myosin proteins. *Future Med Chem* **2013,** 5, (1), 41-52.
50. Higuchi, H.; Takemori, S., Butanedione monoxime suppresses contraction and ATPase activity of rabbit skeletal muscle. *J Biochem* **1989,** 105, (4), 638-43.
51. Ostap, E. M., 2,3-Butanedione monoxime (BDM) as a myosin inhibitor. *J Muscle Res Cell Motil* **2002,** 23, (4), 305-8.
52. Cheung, A.; Dantzig, J. A.; Hollingworth, S.; Baylor, S. M.; Goldman, Y. E.; Mitchison, T. J.; Straight, A. F., A small-molecule inhibitor of skeletal muscle myosin II. *Nat Cell Biol* **2002,** 4, (1), 83-8.
53. Kagawa, M.; Sato, N.; Obinata, T., Effects of BTS (N-benzyl-p-toluene sulphonamide), an inhibitor for myosin-actin interaction, on myofibrillogenesis in skeletal muscle cells in culture. *Zoolog Sci* **2006,** 23, (11), 969-75.

54. Straight, A. F.; Cheung, A.; Limouze, J.; Chen, I.; Westwood, N. J.; Sellers, J. R.; Mitchison, T. J., Dissecting temporal and spatial control of cytokinesis with a myosin II Inhibitor. *Science* **2003,** 299, (5613), 1743-7.
55. Limouze, J.; Straight, A. F.; Mitchison, T.; Sellers, J. R., Specificity of blebbistatin, an inhibitor of myosin II. *J Muscle Res Cell Motil* **2004,** 25, (4-5), 337-41.
56. Allingham, J. S.; Smith, R.; Rayment, I., The structural basis of blebbistatin inhibition and specificity for myosin II. *Nat Struct Mol Biol* **2005,** 12, (4), 378-9.
57. Verhasselt, S.; Roman, B. I.; Bracke, M. E.; Stevens, C. V., Improved synthesis and comparative analysis of the tool properties of new and existing D-ring modified (S)-blebbistatin analogs. *Eur J Med Chem* **2017,** 136, 85-103.
58. Verhasselt, S.; Roman, B. I.; De Wever, O.; Van Hecke, K.; Van Deun, R.; Bracke, M. E.; Stevens, C. V., Discovery of (S)-3'-hydroxyblebbistatin and (S)-3'-aminoblebbistatin: polar myosin II inhibitors with superior research tool properties. *Org Biomol Chem* **2017,** 15, (9), 2104-2118.
59. Matsunaga, K.; Nakatani, K.; Ishibashi, M.; Kobayashi, J.; Ohizumi, Y., Amphidinolide B, a powerful activator of actomyosin ATPase enhances skeletal muscle contraction. *Biochim Biophys Acta* **1999,** 1427, (1), 24-32.
60. Tamura, T.; Iwamoto, H., Thymol: a classical small-molecule compound that has a dual effect (potentiating and inhibitory) on myosin. *Biochem Biophys Res Commun* **2004,** 318, (3), 786-91.
61. Heaslip, A. T.; Leung, J. M.; Carey, K. L.; Catti, F.; Warshaw, D. M.; Westwood, N. J.; Ballif, B. A.; Ward, G. E., A small-molecule inhibitor of T. gondii motility induces the posttranslational modification of myosin light chain-1 and inhibits myosin motor activity. *PLoS Pathog* **2010,** 6, (1), e1000720.
62. Zhao, X.; Ho, D.; Abarzua, P.; Dhar, S. K.; Wang, X.; Jia, Z.; Pannirselvam, M.; Morgans, D. J.; Malik, F. I.; Vatner, S. F., Inhibition of smooth muscle myosin as a novel therapeutic target for hypertension. *J Pharmacol Exp Ther* **2011,** 339, (1), 307-12.
63. Sirigu, S.; Hartman, J. J.; Planelles-Herrero, V. J.; Ropars, V.; Clancy, S.; Wang, X.; Chuang, G.; Qian, X.; Lu, P. P.; Barrett, E.; Rudolph, K.; Royer, C.; Morgan, B. P.; Stura, E. A.; Malik, F. I.; Houdusse, A. M., Highly selective inhibition of myosin motors provides the basis of potential therapeutic application. *Proc Natl Acad Sci U S A* **2016,** 113, (47), E7448-E7455.
64. Green, E. M.; Wakimoto, H.; Anderson, R. L.; Evanchik, M. J.; Gorham, J. M.; Harrison, B. C.; Henze, M.; Kawas, R.; Oslob, J. D.; Rodriguez, H. M.; Song, Y.; Wan, W.; Leinwand, L. A.; Spudich, J. A.; McDowell, R. S.; Seidman, J. G.; Seidman, C. E., A small-molecule inhibitor of sarcomere contractility suppresses hypertrophic cardiomyopathy in mice. *Science* **2016,** 351, (6273), 617-21.
65. Gollapudi, S. K.; Ma, W.; Chakravarthy, S.; Combs, A. C.; Sa, N.; Langer, S.; Irving, T. C.; Nag, S., Two Classes of Myosin Inhibitors, Para-nitroblebbistatin and Mavacamten, Stabilize beta-Cardiac Myosin in Different Structural and Functional States. *J Mol Biol* **2021,** 433, (23), 167295.
66. Zampieri, M.; Argiro, A.; Marchi, A.; Berteotti, M.; Targetti, M.; Fornaro, A.; Tomberli, A.; Stefano, P.; Marchionni, N.; Olivotto, I., Mavacamten, a Novel Therapeutic Strategy for Obstructive Hypertrophic Cardiomyopathy. *Curr Cardiol Rep* **2021,** 23, (7), 79.
67. Bunch, T. A.; Guhathakurta, P.; Lepak, V. C.; Thompson, A. R.; Kanassatega, R. S.; Wilson, A.; Thomas, D. D.; Colson, B. A., Cardiac myosin-binding protein C interaction

with actin is inhibited by compounds identified in a high-throughput fluorescence lifetime screen. *J Biol Chem* **2021,** 297, (1), 100840.
68. Schneidewind, T.; Kapoor, S.; Garivet, G.; Karageorgis, G.; Narayan, R.; Vendrell-Navarro, G.; Antonchick, A. P.; Ziegler, S.; Waldmann, H., The Pseudo Natural Product Myokinasib Is a Myosin Light Chain Kinase 1 Inhibitor with Unprecedented Chemotype. *Cell Chem Biol* **2019,** 26, (4), 512-523 e5.
69. Dulyaninova, N. G.; Hite, K. M.; Zencheck, W. D.; Scudiero, D. A.; Almo, S. C.; Shoemaker, R. H.; Bresnick, A. R., Cysteine 81 is critical for the interaction of S100A4 and myosin-IIA. *Biochemistry* **2011,** 50, (33), 7218-27.
70. Bonello, T. T.; Janco, M.; Hook, J.; Byun, A.; Appaduray, M.; Dedova, I.; Hitchcock-DeGregori, S.; Hardeman, E. C.; Stehn, J. R.; Bocking, T.; Gunning, P. W., A small molecule inhibitor of tropomyosin dissociates actin binding from tropomyosin-directed regulation of actin dynamics. *Sci Rep* **2016,** 6, 19816.
71. Currier, M. A.; Stehn, J. R.; Swain, A.; Chen, D.; Hook, J.; Eiffe, E.; Heaton, A.; Brown, D.; Nartker, B. A.; Eaves, D. W.; Kloss, N.; Treutlein, H.; Zeng, J.; Alieva, I. B.; Dugina, V. B.; Hardeman, E. C.; Gunning, P. W.; Cripe, T. P., Identification of Cancer-Targeted Tropomyosin Inhibitors and Their Synergy with Microtubule Drugs. *Mol Cancer Ther* **2017,** 16, (8), 1555-1565.
72. Mitchell, C. B.; Stehn, J. R.; O'Neill, G. M., Small molecule targeting of the actin associating protein tropomyosin Tpm3.1 increases neuroblastoma cell response to inhibition of Rac-mediated multicellular invasion. *Cytoskeleton (Hoboken)* **2018,** 75, (7), 307-317.
73. Kee, A. J.; Chagan, J.; Chan, J. Y.; Bryce, N. S.; Lucas, C. A.; Zeng, J.; Hook, J.; Treutlein, H.; Laybutt, D. R.; Stehn, J. R.; Gunning, P. W.; Hardeman, E. C., On-target action of anti-tropomyosin drugs regulates glucose metabolism. *Sci Rep* **2018,** 8, (1), 4604.
74. Nagy, L.; Pollesello, P.; Haikala, H.; Vegh, A.; Sorsa, T.; Levijoki, J.; Szilagyi, S.; Edes, I.; Toth, A.; Papp, Z.; Papp, J. G., ORM-3819 promotes cardiac contractility through Ca(2+) sensitization in combination with selective PDE III inhibition, a novel approach to inotropy. *Eur J Pharmacol* **2016,** 775, 120-9.
75. Pollesello, P.; Ben Gal, T.; Bettex, D.; Cerny, V.; Comin-Colet, J.; Eremenko, A. A.; Farmakis, D.; Fedele, F.; Fonseca, C.; Harjola, V. P.; Herpain, A.; Heringlake, M.; Heunks, L.; Husebye, T.; Ivancan, V.; Karason, K.; Kaul, S.; Kubica, J.; Mebazaa, A.; Molgaard, H.; Parissis, J.; Parkhomenko, A.; Poder, P.; Polzl, G.; Vrtovec, B.; Yilmaz, M. B.; Papp, Z., Short-Term Therapies for Treatment of Acute and Advanced Heart Failure-Why so Few Drugs Available in Clinical Use, Why Even Fewer in the Pipeline? *J Clin Med* **2019,** 8, (11).
76. Frank, D. E.; Schnell, F. J.; Akana, C.; El-Husayni, S. H.; Desjardins, C. A.; Morgan, J.; Charleston, J. S.; Sardone, V.; Domingos, J.; Dickson, G.; Straub, V.; Guglieri, M.; Mercuri, E.; Servais, L.; Muntoni, F.; Group, S.-N. S., Increased dystrophin production with golodirsen in patients with Duchenne muscular dystrophy. *Neurology* **2020,** 94, (21), e2270-e2282.
77. Sheikh, O.; Yokota, T., Pharmacology and toxicology of eteplirsen and SRP-5051 for DMD exon 51 skipping: an update. *Arch Toxicol* **2022,** 96, (1), 1-9.
78. Lefeuvre, C.; Brisset, M.; Sarlon, M.; Petit, N.; Orlikowski, D.; Clair, B.; Thiry, T.; Carlier, R. Y.; Prigent, H.; Nicolas, G.; Annane, D.; Laforet, P.; Pouplin, S., Nusinersen

treatment in adults with severe spinal muscular atrophy: A real-life retrospective observational cohort study. *Rev Neurol (Paris)* **2022**.
79. Nayak, A. P.; Lim, J. M.; Arbel, E.; Wang, R.; Villalba, D. R.; Nguyen, T. L.; Schaible, N.; Krishnan, R.; Tang, D. D.; Penn, R. B., Cooperativity between beta-agonists and c-Abl inhibitors in regulating airway smooth muscle relaxation. *FASEB J* **2021,** 35, (7), e21674.
80. Nolen, B. J.; Tomasevic, N.; Russell, A.; Pierce, D. W.; Jia, Z.; McCormick, C. D.; Hartman, J.; Sakowicz, R.; Pollard, T. D., Characterization of two classes of small molecule inhibitors of Arp2/3 complex. *Nature* **2009,** 460, (7258), 1031-4.
81. Baggett, A. W.; Cournia, Z.; Han, M. S.; Patargias, G.; Glass, A. C.; Liu, S. Y.; Nolen, B. J., Structural characterization and computer-aided optimization of a small-molecule inhibitor of the Arp2/3 complex, a key regulator of the actin cytoskeleton. *ChemMedChem* **2012,** 7, (7), 1286-94.
82. Hetrick, B.; Han, M. S.; Helgeson, L. A.; Nolen, B. J., Small molecules CK-666 and CK-869 inhibit actin-related protein 2/3 complex by blocking an activating conformational change. *Chem Biol* **2013,** 20, (5), 701-12.
83. Nurnberg, A.; Kollmannsperger, A.; Grosse, R., Pharmacological inhibition of actin assembly to target tumor cell motility. *Rev Physiol Biochem Pharmacol* **2014,** 166, 23-42.
84. To, C.; Shilton, B. H.; Di Guglielmo, G. M., Synthetic triterpenoids target the Arp2/3 complex and inhibit branched actin polymerization. *J Biol Chem* **2010,** 285, (36), 27944-57.
85. Peterson, J. R.; Lokey, R. S.; Mitchison, T. J.; Kirschner, M. W., A chemical inhibitor of N-WASP reveals a new mechanism for targeting protein interactions. *Proc Natl Acad Sci U S A* **2001,** 98, (19), 10624-9.
86. Peterson, J. R.; Bickford, L. C.; Morgan, D.; Kim, A. S.; Ouerfelli, O.; Kirschner, M. W.; Rosen, M. K., Chemical inhibition of N-WASP by stabilization of a native autoinhibited conformation. *Nat Struct Mol Biol* **2004,** 11, (8), 747-55.
87. Guerriero, C. J.; Weisz, O. A., N-WASP inhibitor wiskostatin nonselectively perturbs membrane transport by decreasing cellular ATP levels. *Am J Physiol Cell Physiol* **2007,** 292, (4), C1562-6.
88. Biber, G.; Ben-Shmuel, A.; Noy, E.; Joseph, N.; Puthenveetil, A.; Reiss, N.; Levy, O.; Lazar, I.; Feiglin, A.; Ofran, Y.; Kedmi, M.; Avigdor, A.; Fried, S.; Barda-Saad, M., Targeting the actin nucleation promoting factor WASp provides a therapeutic approach for hematopoietic malignancies. *Nat Commun* **2021,** 12, (1), 5581.
89. Ostrov, D. A.; Magis, A. T.; Wronski, T. J.; Chan, E. K.; Toro, E. J.; Donatelli, R. E.; Sajek, K.; Haroun, I. N.; Nagib, M. I.; Piedrahita, A.; Harris, A.; Holliday, L. S., Identification of enoxacin as an inhibitor of osteoclast formation and bone resorption by structure-based virtual screening. *J Med Chem* **2009,** 52, (16), 5144-51.
90. Opitz, R.; Muller, M.; Reuter, C.; Barone, M.; Soicke, A.; Roske, Y.; Piotukh, K.; Huy, P.; Beerbaum, M.; Wiesner, B.; Beyermann, M.; Schmieder, P.; Freund, C.; Volkmer, R.; Oschkinat, H.; Schmalz, H. G.; Kuhne, R., A modular toolkit to inhibit proline-rich motif-mediated protein-protein interactions. *Proc Natl Acad Sci U S A* **2015,** 112, (16), 5011-6.
91. Barone, M.; Muller, M.; Chiha, S.; Ren, J.; Albat, D.; Soicke, A.; Dohmen, S.; Klein, M.; Bruns, J.; van Dinther, M.; Opitz, R.; Lindemann, P.; Beerbaum, M.; Motzny, K.; Roske,

Y.; Schmieder, P.; Volkmer, R.; Nazare, M.; Heinemann, U.; Oschkinat, H.; Ten Dijke, P.; Schmalz, H. G.; Kuhne, R., Designed nanomolar small-molecule inhibitors of Ena/VASP EVH1 interaction impair invasion and extravasation of breast cancer cells. *Proc Natl Acad Sci U S A* **2020,** 117, (47), 29684-29690.

92. Bulut, G.; Hong, S. H.; Chen, K.; Beauchamp, E. M.; Rahim, S.; Kosturko, G. W.; Glasgow, E.; Dakshanamurthy, S.; Lee, H. S.; Daar, I.; Toretsky, J. A.; Khanna, C.; Uren, A., Small molecule inhibitors of ezrin inhibit the invasive phenotype of osteosarcoma cells. *Oncogene* **2012,** 31, (3), 269-81.

93. Kahsai, A. W.; Zhu, S.; Wardrop, D. J.; Lane, W. S.; Fenteany, G., Quinocarmycin analog DX-52-1 inhibits cell migration and targets radixin, disrupting interactions of radixin with actin and CD44. *Chem Biol* **2006,** 13, (9), 973-83.

94. Celik, H.; Hong, S. H.; Colon-Lopez, D. D.; Han, J.; Kont, Y. S.; Minas, T. Z.; Swift, M.; Paige, M.; Glasgow, E.; Toretsky, J. A.; Bosch, J.; Uren, A., Identification of Novel Ezrin Inhibitors Targeting Metastatic Osteosarcoma by Screening Open Access Malaria Box. *Mol Cancer Ther* **2015,** 14, (11), 2497-507.

95. Chen, L.; Yang, S.; Jakoncic, J.; Zhang, J. J.; Huang, X. Y., Migrastatin analogues target fascin to block tumour metastasis. *Nature* **2010,** 464, (7291), 1062-6.

96. Lecomte, N.; Njardarson, J. T.; Nagorny, P.; Yang, G.; Downey, R.; Ouerfelli, O.; Moore, M. A.; Danishefsky, S. J., Emergence of potent inhibitors of metastasis in lung cancer via syntheses based on migrastatin. *Proc Natl Acad Sci U S A* **2011,** 108, (37), 15074-8.

97. Kraft, R.; Kahn, A.; Medina-Franco, J. L.; Orlowski, M. L.; Baynes, C.; Lopez-Vallejo, F.; Barnard, K.; Maggiora, G. M.; Restifo, L. L., A cell-based fascin bioassay identifies compounds with potential anti-metastasis or cognition-enhancing functions. *Dis Model Mech* **2013,** 6, (1), 217-35.

98. Huang, J.; Dey, R.; Wang, Y.; Jakoncic, J.; Kurinov, I.; Huang, X. Y., Structural Insights into the Induced-fit Inhibition of Fascin by a Small-Molecule Inhibitor. *J Mol Biol* **2018,** 430, (9), 1324-1335.

99. Huang, F. K.; Han, S.; Xing, B.; Huang, J.; Liu, B.; Bordeleau, F.; Reinhart-King, C. A.; Zhang, J. J.; Huang, X. Y., Targeted inhibition of fascin function blocks tumour invasion and metastatic colonization. *Nat Commun* **2015,** 6, 7465.

100. Lin, L.; Lin, K.; Wu, X.; Liu, J.; Cheng, Y.; Xu, L. Y.; Li, E. M.; Dong, G., Potential Inhibitors of Fascin From A Database of Marine Natural Products: A Virtual Screening and Molecular Dynamics Study. *Front Chem* **2021,** 9, 719949.

101. Francis, S.; Croft, D.; Schuttelkopf, A. W.; Parry, C.; Pugliese, A.; Cameron, K.; Claydon, S.; Drysdale, M.; Gardner, C.; Gohlke, A.; Goodwin, G.; Gray, C. H.; Konczal, J.; McDonald, L.; Mezna, M.; Pannifer, A.; Paul, N. R.; Machesky, L.; McKinnon, H.; Bower, J., Structure-based design, synthesis and biological evaluation of a novel series of isoquinolone and pyrazolo[4,3-c]pyridine inhibitors of fascin 1 as potential anti-metastatic agents. *Bioorg Med Chem Lett* **2019,** 29, (8), 1023-1029.

102. Alburquerque-Gonzalez, B.; Bernabe-Garcia, M.; Montoro-Garcia, S.; Bernabe-Garcia, A.; Rodrigues, P. C.; Ruiz Sanz, J.; Lopez-Calderon, F. F.; Luque, I.; Nicolas, F. J.; Cayuela, M. L.; Salo, T.; Perez-Sanchez, H.; Conesa-Zamora, P., New role of the antidepressant imipramine as a Fascin1 inhibitor in colorectal cancer cells. *Exp Mol Med* **2020,** 52, (2), 281-292.

103. Jackson, J. E.; Kopecki, Z.; Adams, D. H.; Cowin, A. J., Flii neutralizing antibodies improve wound healing in porcine preclinical studies. *Wound Repair Regen* **2012,** 20, (4), 523-36.
104. Haidari, H.; Zhang, Q.; Melville, E.; Kopecki, Z.; Song, Y.; Cowin, A. J.; Garg, S., Development of Topical Delivery Systems for Flightless Neutralizing Antibody. *J Pharm Sci* **2017,** 106, (7), 1795-1804.
105. Johnson, K.; Zhu, S.; Tremblay, M. S.; Payette, J. N.; Wang, J.; Bouchez, L. C.; Meeusen, S.; Althage, A.; Cho, C. Y.; Wu, X.; Schultz, P. G., A stem cell-based approach to cartilage repair. *Science* **2012,** 336, (6082), 717-21.
106. Wang, H. Y.; Bakshi, K.; Frankfurt, M.; Stucky, A.; Goberdhan, M.; Shah, S. M.; Burns, L. H., Reducing amyloid-related Alzheimer's disease pathogenesis by a small molecule targeting filamin A. *J Neurosci* **2012,** 32, (29), 9773-84.
107. Wang, H. Y.; Pei, Z.; Lee, K. C.; Lopez-Brignoni, E.; Nikolov, B.; Crowley, C. A.; Marsman, M. R.; Barbier, R.; Friedmann, N.; Burns, L. H., PTI-125 Reduces Biomarkers of Alzheimer's Disease in Patients. *J Prev Alzheimers Dis* **2020,** 7, (4), 256-264.
108. Zhang, L.; Huang, T.; Teaw, S.; Nguyen, L. H.; Hsieh, L. S.; Gong, X.; Burns, L. H.; Bordey, A., Filamin A inhibition reduces seizure activity in a mouse model of focal cortical malformations. *Sci Transl Med* **2020,** 12, (531).
109. Rizvi, S. A.; Neidt, E. M.; Cui, J.; Feiger, Z.; Skau, C. T.; Gardel, M. L.; Kozmin, S. A.; Kovar, D. R., Identification and characterization of a small molecule inhibitor of formin-mediated actin assembly. *Chem Biol* **2009,** 16, (11), 1158-68.
110. Arden, J. D.; Lavik, K. I.; Rubinic, K. A.; Chiaia, N.; Khuder, S. A.; Howard, M. J.; Nestor-Kalinoski, A. L.; Alberts, A. S.; Eisenmann, K. M., Small-molecule agonists of mammalian Diaphanous-related (mDia) formins reveal an effective glioblastoma anti-invasion strategy. *Mol Biol Cell* **2015,** 26, (21), 3704-18.
111. Lash, L. L.; Wallar, B. J.; Turner, J. D.; Vroegop, S. M.; Kilkuskie, R. E.; Kitchen-Goosen, S. M.; Xu, H. E.; Alberts, A. S., Small-molecule intramimics of formin autoinhibition: a new strategy to target the cytoskeletal remodeling machinery in cancer cells. *Cancer Res* **2013,** 73, (22), 6793-803.
112. Delanote, V.; Vanloo, B.; Catillon, M.; Friederich, E.; Vandekerckhove, J.; Gettemans, J., An alpaca single-domain antibody blocks filopodia formation by obstructing L-plastin-mediated F-actin bundling. *FASEB J* **2010,** 24, (1), 105-18.
113. Chellaiah, M. A.; Majumdar, S.; Aljohani, H., Peptidomimetic inhibitors of L-plastin reduce the resorptive activity of osteoclast but not the bone forming activity of osteoblasts in vitro. *PLoS One* **2018,** 13, (9), e0204209.
114. Li, X.; Wang, L.; Huang, B.; Gu, Y.; Luo, Y.; Zhi, X.; Hu, Y.; Zhang, H.; Gu, Z.; Cui, J.; Cao, L.; Guo, J.; Wang, Y.; Zhou, Q.; Jiang, H.; Fang, C.; Weng, W.; Chen, X.; Chen, X.; Su, J., Targeting actin-bundling protein L-plastin as an anabolic therapy for bone loss. *Sci Adv* **2020,** 6, (47).
115. Aljohani, H.; Stains, J. P.; Majumdar, S.; Srinivasan, D.; Senbanjo, L.; Chellaiah, M. A., Peptidomimetic inhibitor of L-plastin reduces osteoclastic bone resorption in aging female mice. *Bone Res* **2021,** 9, (1), 22.
116. Gau, D.; Lewis, T.; McDermott, L.; Wipf, P.; Koes, D.; Roy, P., Structure-based virtual screening identifies a small-molecule inhibitor of the profilin 1-actin interaction. *J Biol Chem* **2018,** 293, (7), 2606-2616.

117. Fukui, Y.; Ihara, S., A mutant of SWAP-70, a phosphatidylinositoltrisphosphate binding protein, transforms mouse embryo fibroblasts, which is inhibited by sanguinarine. *PLoS One* **2010,** 5, (12), e14180.

118. Baranov, M. V.; Revelo, N. H.; Verboogen, D. R. J.; Ter Beest, M.; van den Bogaart, G., SWAP70 is a universal GEF-like adaptor for tethering actin to phagosomes. *Small GTPases* **2019,** 10, (4), 311-323.
